# Amino Alcohols as Potential Antibiotic and Antifungal Leads

**DOI:** 10.3390/molecules27072050

**Published:** 2022-03-22

**Authors:** Jennifer R. Baker, Peter J. Cossar, Mark A. T. Blaskovich, Alysha G. Elliott, Johannes Zuegg, Matthew A. Cooper, Peter J. Lewis, Adam McCluskey

**Affiliations:** 1 Chemistry, School of Environmental & Life Sciences, The University of Newcastle, University Drive Callaghan, Newcastle, NSW 2308, Australia; jennifer.r.baker@newcastle.edu.au (J.R.B.); p.cossar@tue.nl (P.J.C.); 2 Community for Open Antimicrobial Drug Discovery, Centre for Superbug Solutions, Institute for Molecular Bioscience, The University of Queensland, Brisbane, QLD 4072, Australia; m.blaskovich@imb.uq.edu.au (M.A.T.B.); a.elliott@imb.uq.edu.au (A.G.E.); j.zuegg@imb.uq.edu.au (J.Z.); m.cooper@uq.edu.au (M.A.C.); 3 Biology, School of Environmental & Life Sciences, The University of Newcastle, University Drive Callaghan, Newcastle, NSW 2308, Australia; peter.lewis@newcastle.edu.au; 4 Molecular Horizons, School of Chemistry and Molecular Bioscience, University of Wollongong, Wollongong, NSW 2522, Australia

**Keywords:** antibiotic, antifungal, acrylonitriles, focused libraries

## Abstract

Five focused compound libraries (forty-nine compounds), based on prior studies in our laboratory were synthesized and screened for antibiotic and anti-fungal activity against *S.* *aureus*, *E.* *coli*, *K.* *pneumoniae*, *P. aeruginosa*, *A. baumannii,* *C. albicans* and *C. neoformans*. Low levels of activity, at the initial screening concentration of 32 μg/mL, were noted with analogues of (*Z*)-2-(3,4-dichlorophenyl)-3-phenylacrylonitriles which made up the first two focused libraries produced. The most promising analogues possessing additional substituents on the terminal aromatic ring of the synthesised acrylonitriles. Modifications of the terminal aromatic moiety were explored through epoxide installation flowed by flow chemistry mediated ring opening aminolysis with discreet sets of amines to the corresponding amino alcohols. Three new focused libraries were developed from substituted anilines, cyclic amines, and phenyl linked heterocyclic amines. The aniline-based compounds were inactive against the bacterial and fungal lines screened. The introduction of a cyclic, such as piperidine, piperazine, or morpholine, showed >50% inhibition when evaluated at 32 μg/mL compound concentration against methicillin-resistant *Staphylococcus* *aureus*. Examination of the terminal aromatic substituent via oxirane aminolysis allowed for the synthesis of three new focused libraries of afforded amino alcohols. Aromatic substituted piperidine or piperazine switched library activity from antibacterial to anti-fungal activity with ((*Z*)-2-(3,4-dichlorophenyl)-3-(4-(2-hydroxy-3-(4-methylpiperazin-1-yl)propoxy)phenyl)acrylonitrile), ((*Z*)-2-(3,4-dichlorophenyl)-3-(4-(2-hydroxy-3-(4-(4-hydroxyphenyl)piperazin-1-yl)propoxy)-phenyl)acrylonitrile) and ((*Z*)-3-(4-(3-(4-cyclohexylpiperazin-1-yl)-2-hydroxypropoxy)-phenyl)-2-(3,4-dichlorophenyl)-acrylonitrile) showing >95% inhibition of *Cryptococcus neoformans var. grubii* *H99* growth at 32 μg/mL. While (*Z*)-3-(4-(3-(cyclohexylamino)-2-hydroxypropoxy)phenyl)-2-(3,4-dichlorophenyl)-acrylonitrile, (*S,Z*)-2-(3,4-dichlorophenyl)-3-(4-(2-hydroxy-3-(piperidin-1-yl)propoxy)phenyl)acrylonitrile, (*R,Z*)-2-(3,4-dichlorophenyl)-3-(4-(2-hydroxy-3-(piperidin-1-yl)propoxy)phenyl)acrylonitrile, (*Z*)-2-(3,4-dichlorophenyl)-3-(4-(2-hydroxy-3-(D-11-piperidin-1-yl)propoxy)phenyl)-acrylonitrile, and (*Z*)-3-(4-(3-(4-cyclohexylpiperazin-1-yl)-2-hydroxypropoxy)-phenyl)-2-(3,4-dichlorophenyl)-acrylonitrile 32 μg/mL against *Staphylococcus* *aureus*.

## 1. Introduction

The recent report detailing bacterial isolates resistant to polymyxin removes the last effective class of antibiotics against multi-drug resistant (MDR) bacteria [1]. With this loss of the last line of defenses against bacteria, the health community believes that this places us on the brink of a “post-antibiotic era” [2]. In the USA alone, >2 million people are subject to serious antibiotic resistant bacterial infections each year [3]. Across the USA and Europe, antibiotic resistant and fungal infections are responsible for annual deaths of >20,000 and 25,000, respectively [3,4]. Most concerning is the lack of efficacious compounds which treat Gram-negative bacteria [5,6,7]. There are only a handful of potential drugs which target Gram-negative bacteria in clinical trials offering significant benefits over current clinically used antibiotics [5,8,9].

Currently most of the antibiotic development is focused on improvements to existing antibiotics. This lack of structural novelty progressing to the clinic positions generational compound development as a critical strategy for the near future, but it is not known if this approach is sustainable or can addresses the growing problem of antibiotic resistance. Governments are starting to react through initiatives such as the Combating Antibiotic Resistant Bacteria Biopharmaceutical Accelerator (CARB-X) initiative, but a significant number of pharmaceutical companies are no longer pursuing antibiotic development [10,11,12]. Despite a recent resurgence in antibiotic drug discovery, to date there has been limited progress in developing novel mode of action antibiotics [5,8,9,13,14].

**Figure 1:**
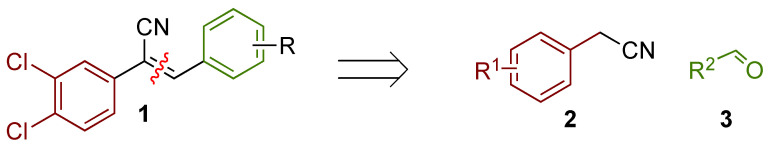
An illustrative example of an AhR active ligand (**1**), with the proposed retrosynthesis, and potential analogue development from substituted phenyacetonitriles (**2**) and aldehydes (**3**).

As part of a series of studies we have developed a number of classes of biologically active molecules [15,16,17,18,19,20]. With our in-house compound library, we sought to examine one of our compound series, that were originally designed to target the aryl hydrocarbon receptor (AhR) (Figure 1) [15,21,22,23], for activity against methicillin-resistant *Staphylococcus aureus* (MRSA), *E. coli*, *Klebsiella pneumoniae*, *Acinetobacter baumannii* and *Pseudomonas aeruginosa*; and the yeasts *Candida albicans* and *Cryptococcus neoformans*. We rationalized that the ease of synthetic access and further modification to amino alcohols, a class of compounds that spans the amphenicol and key macrolide antibiotics, positioned these compounds as logical development leads [24,25,26].

## 2. Results

The acrylonitrile analogues **1a**–**1m** (*Library A*) were generated as previously described from the Knoevenagel condensation of 3,4-dichlorophenylacetonitrile (**2a**) and substituted benzaldehydes (**3a**–**m**) (Supporting Information for detail) [15,21]. Screening of *Library A*, against the *S. aureus*, *E. coli*, *K. pneumoniae*, *P. aeruginosa*, *A. baumannii* bacterial and *C. albicans*, and *C. neoformans* fungal lines at an initial compound test concentration of 32 μg/mL gave the data presented in Table 1.

Analysis of the *Library A* data from (Table 1) revealed no broad-spectrum antibiotic activity 32 μg/mL with only low levels of bacteria and fungi death noted. Of note the 4-nitroanilino **1e** showed 28% inhibition of *P. aeruginosa*; 4-anilino **1g**, 37% inhibition of *A. baumannii*; 4-propyldimethyl amino ether **1j**, 28% inhibition of *A. baumannii* and 3-nitrovanillin **1m**, 64% inhibition of *Candida albicans* growth. While poor, all of the most active species retain a 4-substituent on the aromatic ring originating from the aldehydes (**2**).

**Scheme 1:**
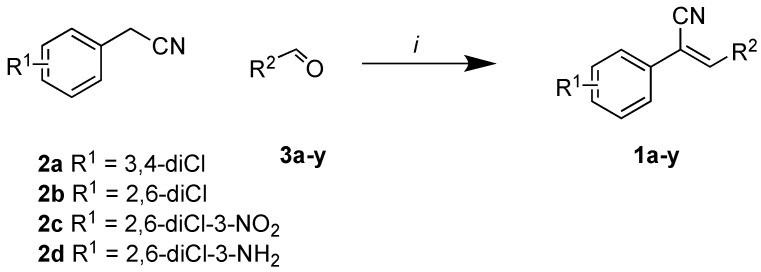
*Reagents and Conditions.* (*i*) PhCH_2_NMe_3_(OH), H_2_O, 50 °C, 5 h.

The second library, *Library B*, saw the use of phenylacetonitriles (**2b**–**d**; Scheme 1) and selected aldehydes spanning pyrrole carboxaldehydes (**3n**–**q**), 1*H*-benzo[*d*]imidazole carboxaldehyde (**3r**); and nitro-benzaldehydes (**3s**–**w**) and amino-benzaldehydes (**3x**, **3y**) (Supporting Information) [21]. These compounds were subjected to bacterial and fungal screening as before and this data is presented in Table 2 [27].

Examination of the data presented in Table 2 revealed a modest increase in activity of *Library B* analogues at the initial screening concentration (32 μg/mL) with 2-bromopyrrole **1o** showing 37% inhibition of *C. albicans* growth; 2,3-dibromopyrrole **1p** 32% inhibition of *S. aureus* growth; 1*H*-benzo[*d*]imidazole (**1r**), 27% inhibition of *P. aeruginosa* growth. With the 2,6-dichlorophenylacrylonitrile (**2b**) based 3′-nitrophenyl (**1u**), 26% inhibition of *C. neoformans* growth; the 2,6-dichloro-3-nitrophenylacetonitrile (**2c**) based 2′-nitrophenyl (**1s**) 29% inhibition of *C. neoformans* growth. The corresponding diamino analogue to (**1v**), compound (**1y**), demonstrated the best activity of any in *Library B*, exhibiting 48% inhibition against *A. baumannii growth.* These data support retention of a nitrogen substituent, with the enhanced activity possibly due to electronic effects. The activity increases were limited, and with this in mind we revisited *Library A* where the most active analogues suggested a preference for a 4-substituted aromatic moiety. As amino alcohol containing macrolides are known to have antibiotic properties, we rationalized that introducing such a moiety might confer enhanced activity to our lead compounds described above [24,25,26]. Access to the desired amino alcohol analogues was via introduction, and subsequent ring opening of an oxirane moiety with an amine.

**Scheme 2:**
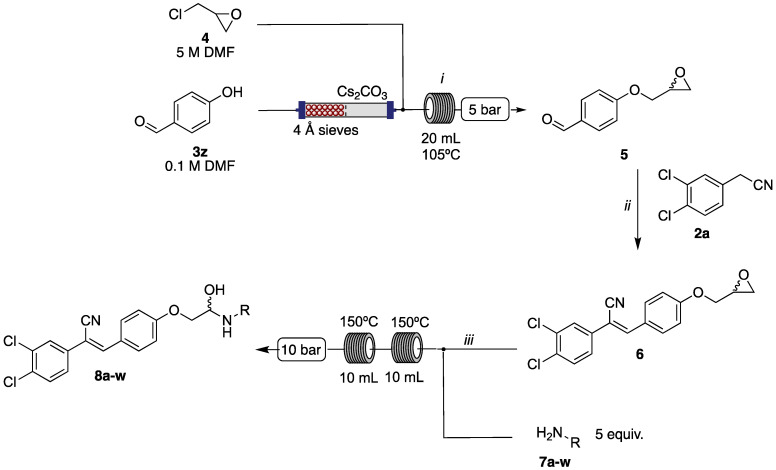
Reagents and conditions: (*i*) Vapourtec Easy-MedChem, 5 M **4** (anhy. DMF), 0.1 M **3z** (anhy. DMF), 105 °C, 5 bar, 1.0 mL·min^−1^ (t_r_ = 20 min); (*ii*) **1a**, PhCH_2_NMe_3_(OH), H_2_O, 50 °C, 5h; (*iii*) RNH_2_ (**7a**–**w**) 0.05 M (EtOH), **6** 0.01 M (EtOH), 150 °C, 10 bar, 1.0 mL·min^−1^ (t_r_ = 20 min).

In a typical experiment, epichlorohydrin (**4**) was coupled to the 4-hydroxybenzaldehyde (**3z**), yielding epoxyether (**5**) (Supporting Information) [28]. Subsequent Knoevenagel condensation with **2a** under batch conditions gave the key intermediate (**6**). The desired libraries of amino alcohols were accessed through an amine (**7a**–**w**; see Table 3, Table 4 and Table 5 for details) mediated ring opening of **6**. This protocol afforded a diverse array of amino alcohols which categorized as three libraries in total (**8a**–**w**) [28]. *Library C* (**6**, **8a**–**8d**), from simple anilines; *Library D* (**8e**–**8l**) from cyclic aliphatic amines; and *Library E* (**8m**–**8w**) from aromatic piperidines and piperazines (see Table 3, Table 4 and Table 5 for details and Scheme 2).

*Library C* retained the 3,4-dichlorophenylacetonitrile (**2a**), with the introduction of simple anilines via epoxide ring opening of **6** (Scheme 2). Screening of this library as before and analysis of this date revealed that no *Library C* analogue displayed broad spectrum activity. Analogues **6**, **8a** and **8d** showed >20% inhibition against *P. aeruginosa* (Table 3).

*Library D* (**8e**–**8l**) saw introduction of simple cyclic aliphatic and small heterocyclic rings. This minor modification had a major effect on the degree and number of active compounds (Table 4). Screening of these analogues at 32 μg/mL, and analysis of the data acquired revealed modest levels of activity with cyclohexyl **8e** displaying 88% inhibition of *S. aureus* growth, and cyclohexyl piperidine (**8f**) 55% inhibition of *S. aureus*; piperidine (**8g**) 67 and 50% inhibition of *S. aureus* and *C. albicans* growth, respectively.

The introduction of the amino alcohol moiety also introduced a stereocenter with the added -OH moiety. To examine the effect of the two possible stereoisomers, optically pure (**8h**), (**8i**) and racemic (**8g**) where prepared. Both (**8h**) and (**8i**) returned essentially identical inhibition of *S. aureus* at 92 and 90% inhibition, respectively, suggesting no stereochemical preference for the -OH moiety. However, racemic (**8g**) displayed 20% lower *S. aureus* at 67% inhibition.

With other *Library D* analogues, per-deuteration of the piperidine moiety giving (**8j**) retained 90% inhibition of *S. aureus* growth. The piperazine acetamide (**8k**) and morpholino (**8l**), lost antibiotic activity but showed 53% and 35% growth inhibition of *C. albicans*, respectively. These latter compounds suggest that the presence of polar moieties on the heterocyclic ring are detrimental to antibiotic activity but may be favourable for fungal activity.

Having established the preferential installation of small cyclic aliphatics and heterocyclic moieties, *Library E* extended the substituents of these rings through the addition of a terminal aromatic moiety. The inclusion of the additional aromatic moiety saw modest activity, at the initial 32 μg/mL screening concentration, with acetophenone (**8u**) inhibiting *P. aeruginosa* growth (28%); 4-bromophenyl (**8r**) inhibited *K. pneumoniae* (28%); 4-(*p*-tolyl)piperazine (**8p**) inhibited *C. neoformans* (96%); and 4-methylpiperazinylphenol (**8s**) inhibited the *C. albicans* (65%) and *C. neoformans* (97%) fungal lines.

Having completed the initial screening of these focused libraries at 32 μg/mL, analogues showing >85% activity against at least one of the bacteria or fungi lines proceeded to full minimum inhibitory concentration (MIC) determination by broth micro-dilution dose response assay. These data are presented in Table 6.

As can be seen from the data presented in Table 6, it is clear that the MIC values across the analogues evaluated was generally 32 μg/mL. Analogues (**8e**), (**8h**), (**8i**) and (**8j**) were all confirmed to be active with an MIC of 32 μg/mL against *S. aureus*, which is equipotent with the clinically used colistin, but less active than amoxicillin. These analogues did not show broad-spectrum antimicrobial activity with no activity at the highest tested concentration against the four Gram-negative strains, nor against the two yeasts tested. While this is modest, it does support the screening of biologically active scaffolds against alternative targets.

## 3. Discussion

Within the pool of 3,4-dichlorophenyl analogues, *Library A*, 4-NO_2_ (**1e**) was more active against *P. aeruginosa* than the corresponding 2-NO_2_ (**1c**) and 3-NO_2_ (**1d**). Modification of the 4-NO_2_ moiety to 4-NH_2_ with (**1f**) saw a loss of *P. aeruginosa* activity, but modest activity against *A. baumannii*. This was the sole aniline analogue to display activity. Incorporation of the *N,N*-dimethylamino moiety (**1g**) resulted in a slight potency reduction, whereas nitro-vanillin (**1m**) showed no antibiotic activity, but modest effects against *C. albicans*. Caution is required in ascribing definitive SAR outcomes in these series as they involve analysis of a single datapoint.

Modification of either the parent phenylacrylonitrile via the 2,6-dichloro or 2,6-dichloro-3-nitro or 2,6-dichloro-3-amino phenylacrylonitrile or parent aldehyde with pyrrole, imidazole and nitrophenyl substituents all displayed lower levels of activity than *Library A* analogues. Excepting 3′-amino (**1y**), with modest activity against *A. baumannii*. However, within this Library general trends were evident, with the di-Br (**1p**) the most active against *S. aureus*; mono-Br (**1o**) against *C. albicans* and benzimidazole (**1r**) against *P. aeruginosa*.

The development of analogues with the inclusion of the β-aminoalcohol moiety, while retaining the 3,4-dichlorophenyl moiety was readily accomplished in a series of simple flow chemistry mediated steps. However, the retention of the terminal aromatic moieties with *Library C* showed essentially no activity. The use of cyclic and heterocyclic amines in the oxiraine aminolysis step, gave analogues **8e**–**8l**. This removal of the terminal aromatic moiety was rewarded with a significant increase in antibiotic activity, especially against *S. aureus* (**8e**–**8j**, >50% inhibition at 32 μg/mL). Individual enantiomers (**8h**) and (**8i**) were equipotent (>90%), yet the racemate (**8g**) showed only partial inhibition of MRSA at 32 μg/mL (67%), supporting a negative cooperability of these isomers. This stereochemical preference will be examined and reported on in a future study. Alternatively, as the deuterated racemate (**8j**) showed the same activity as the pure isomers (**8h**) and (**8i**), incorporation of such moieties may negate the need to isolate single pure stereoisomers. This supported further development of this series with the introduction of an aromatic moiety appended to a saturated six-membered ring (i.e., piperazine and piperidine). Incorporation of polar moieties in the terminus of the heterocyclic, viz *N*-Ac (**8k**) and morpholino (**18l**), were detrimental to *S. aureus* activity, but these analogues showed modest activity against *C. albicans*.

Within *Library E* switching the ether linker position from 4- (**8o**) to 3- (**8n**) and 2- (**8m**) was detrimental to activity. These analogues were essentially devoid of antibacterial activity. The switch to antifungal activity was unexpected, but combined with the observed antibiotic activity strongly supports the on-going development of this class of compounds as novel antibacterial/antifungal agents. Of the analogues with antifungal activity, the piperidine (**8g**, **8x**) is more prevalent than piperazine (**8p**). This is fortuitous as phenylpiperazines are known to form highly reactive iminoquinone moieties (a potential toxicophore) capable of reacting with glutathione [29,30,31,32]. Fungal active compounds show higher specificity towards the yeasts with both (**8p**) and (**8s**) inhibiting >95% *C. neoformans* growth at 32 μg/mL. Analogue (**8s**) shows promising activity against both yeasts, *C. albicans* and *C. neoformans* with 65 and 97% inhibition at 32 μg/mL, respectively. These compounds were not antibacterial.

Examination of the most active analogues across all five libraries (**8e**, **8h**, **8i**, **8j**, **8s** and **8x**) in a broth microdilution dose response assay revealed (**8e**), and (8**h**–**i**) as having MIC values of 32 μg/mL (which is equivalent to an IC_50_ value of approximately 40 μM), which represents a significant enhancement relative to lead **1**. To further explore the efficacy of these compounds, cytotoxicity screening was conducted in HEK393 cells and all evaluated analogues returned growth inhibition values >20 μM.

## 4. Conclusions

Five focused libraries totaling 49 compounds (plus the oxirane intermediate, **6**) were rapidly assembled. With the exception of *Library C*, active compounds were observed across all focused libraries. The most promising antibiotic activity was observed with (**8e**–**8i**) against *S. aureus*. A negative cooperativity between isomers in the racemic (**8e**) was noted (with single enantiomers (**8g**) and (**8i**) ca 25% more active). This effect was ameliorated through the introduction of a per-deuterated piperidine moiety (**8j**). The introduction of aromatic substituents to the piperidine or piperazine moiety resulted in a transition from antibiotic to antifungal activity with (**8p**), (**8s**) and (**8x**) showing excellent activity at >95% inhibition of *C. neoformans* at 32 μg/mL (which is equivalent to an IC_50_ value of approximately 40 μM).

From this study we have identified a novel scaffold that is active against *S. aureus* (**8e**, **8h**–**8j**), and with structural manipulation shows differential activity against *C. albicans* (**8p**, **8s** and **8x**).

## 5. Experimental

### 5.1. Biology

All bacteria and yeast strains were sourced from the American Type Culture Collection (ATCC) and antimicrobial assays were conducted by the Community for Open Antimicrobial Drug Discovery (CO-ADD) [33,34,35]. Relevant positive inhibitor controls for each species were included in all assays.

Compounds for inhibitory assays were prepared in DMSO and water to a final testing concentration of (i) 32 μg/mL or 10 μM for single point concentration screening, or (ii) serially diluted two-fold down the wells for 8 times with a top testing concentration of 32 μg/mL or 10 μM for minimum inhibitory concentration (MIC) dose response assays, in 384-well, non-binding surface plate (NBS, Corning 3460) for each bacterial/yeast strain to be tested, and in duplicate (n = 2), Final maximum DMSO concentration was 1%. All of the sample-preparations were carried out using liquid handling robots.

### 5.2. Antibacterial Assays

For both single concentration screening and dose response assays, all bacteria were cultured in Cation-adjusted Mueller Hinton broth (CAMHB) at 37 °C overnight. A sample of each culture was then diluted 40-fold in fresh broth and incubated at 37 °C for 1.5–3 h. The resultant mid-log phase cultures were diluted (CFU/mL measured by OD_600_), then added to each well of the compound containing plates, giving a cell density of 5 × 10^5^ CFU/mL and a total volume of 50 μL in 384-well non-binding surface plates (NBS, Corning 3460). All of the plates were covered and incubated at 37 °C for 18 h without shaking.

Inhibition of bacterial growth was determined measuring absorbance at 600 nm (OD_600_), using a Tecan M1000 Pro monochromator plate reader. The percentage of growth inhibition was calculated for each well, using the negative control (media only) and positive control (bacteria without inhibitors) on the same plate as references.

The percentage of growth inhibition was calculated for each well, using the negative control (media only) and positive control (bacteria without inhibitors) on the same plate. For single point concentration assays the final recorded readout was the percentage inhibition at the concentration tested (i.e., % at 32 μg/mL or 10 μM). For the dose response MIC assay, the MIC was determined as the lowest concentration at which the growth was fully inhibited, defined by an inhibition ≥ 80% by spectrophotometric read, the equivalent 100% inhibition by eye.

### 5.3. Antifungal Assay

For both single concentration screening and dose response assays, fungi/yeast strains were cultured for three days on yeast extract-peptone dextrose (YPD) agar at 30 °C. A yeast suspension of 1 × 10^6^ to 5 × 10^6^ CFU/mL (as determined by OD_530_) was prepared from five colonies. The suspension was subsequently diluted and added to each well of the compound-containing plates giving a final cell density of fungi suspension of 2.5 × 10^3^ CFU/mL and a total volume of 50 μL in 384-well non-binding surface plates (NBS, Corning 3460). All plates were covered and incubated at 35 °C for 36 h without shaking.

Growth inhibition of *C. albicans* was determined measuring absorbance at 630 nm (OD_630_), while the growth inhibition of *C. neoformans* was determined measuring the difference in absorbance between 600 and 570 nm (OD_600–570_), after the addition of resazurin (0.001% final concentration) and incubation at 35 °C for 2 h. The absorbance was measured using a Biotek Multiflo Synergy HTX plate reader.

For both yeasts, the percentage of growth inhibition was calculated for each well, using the negative control (media only) and positive control (fungi without inhibitors) on the same plate. For single point concentration assays the final recorded readout was the percentage inhibition at the concentration tested (i.e., % at 32 μg/mL or 10 μM). For the dose response MIC assay, the MIC was determined as the lowest concentration at which the growth was fully inhibited, defined by an inhibition ≥80% for *C. albicans* and an inhibition ≥70% for *C. neoformans*. Due to a higher variance in growth and inhibition, a lower threshold was applied to the data for *C. neoformans*.

### 5.4. Chemistry

#### General Methods

All reactions were performed using standard laboratory equipment and glassware. Solvents and reagents were purchased from Sigma Aldrich, Alfa Aesar or AK Scientific (Australia) and used as received. Organic solvents were of bulk quality. Organic solvent extracts were dried with magnesium sulfate (MgSO_4_), and dried under reduced pressure with either Büchi or Heidolph rotary evaporators. Melting points were recorded in open capillaries on a Stuart SMP11 Melting Point Apparatus. Where available, literature values are provided and appropriately referenced. Electrospray mass spectra were recorded using HPLC-grade 10% acetonitrile/H_2_O or 100% acetonitrile (with 0.1% formic acid) as carrier solvents on an Agilent Technologies 1260 Infinity UPLC system with a 6120 Quadrupole LC/MS in electrospray ionization (ESI) positive and negative modes. TLC was performed on Merck silica gel 60 F254 pre-coated aluminum plates with a thickness of 0.2 mm. Column chromatography was performed under ‘flash’ conditions on Merck silica gel 60 (230–400 mesh).

Nuclear magnetic resonance (NMR) spectroscopy was performed on a Bruker Avance III 400 MHz spectrometer, where proton NMR (^1^H NMR) spectra and carbon NMR (^13^C NMR) spectra were acquired at 400 and 100 MHz, respectively, or a Bruker Avance III 600 MHz spectrometer, where proton NMR (^1^H NMR) spectra and carbon NMR (^13^C NMR) spectra were acquired at 600 and 150 MHz, respectively. All spectra were recorded in deuterated dimethyl sulfoxide (DMSO-*d_6_*) or deuterated acetone (acetone-*d_6_*) obtained from Cambridge Isotope Laboratories Inc. Chemical shifts (δ) were measured in parts per million (ppm) and referenced against the internal reference peaks. Coupling constants (*J*) were measured in Hertz (Hz). NMR assignments were determined through the interpretation of one- and two-dimensional spectra. Multiplicities are denoted as singlet (s), broad singlet (bs), doublet (d), doublet of doublets (dd), doublet of doublet of doublets (ddd), triplet (t), quartet (q), triplet of doublets (td), doublet of triplets (dt) and multiplet (m). Peaks are listed in decreasing chemical shift in the following format: chemical shift (integration (^1^H), multiplicity (^1^H), coupling constant (^1^H). The Biotage^®^ initiator+ was used to perform microwave reactions.

Experimental details for all previously published compounds can be found in the Supplementary Material. Spectral data for all compounds can be found in the Supplementary material.

General Procedure 1: - The aldehyde (1.05 eq., 1.05 mmol), was added to a vigorously stirred solution of water (10 mL) and heated to 50 °C. The dichlorophenylacetonitrile (1 mmol) was then slowly added forming a suspension. After 5–10 min of stirring, 40% PhCH_2_NMe_3_(OH) (7 mL) was added dropwise. After complete addition, the reaction vessel stirred at 50 °C for 5 h. After this period, the solution was filtered hot, washed with warm water and purified by either recrystallisation or column chromatography.

General Procedure 2: - (*Z*)-2-(3,4-dichlorophenyl)-3-(4-(oxiran-2-ylmethoxy)phenyl)acrylonitrile (**15**, 1 eq.) was combined with the required amine (either 1.5 or 2 eq., as stated) and 20 mL ethanol. The solution was irradiated at 120 °C for 20 min. Upon chilling, the desired product was isolated via vacuum filtration.

General Procedure 3: - Using a Vapourtec RS-400 equipped with fraction collection kit and auto-sampler, a 2.0 mL sample loop was charged with a 0.4 M solution of epoxide (**15**) in toluene. An additional 2 mL sample loop was charged with a 2.8 M amine solution in ethanol (as stated). The solutions were flowed together and the resulting stream was then passed through two PFA coil reactors in series at 150 °C, 10 bar back pressure and 0.5 mL·min^−1^ (residence time 40 min). The resulting reaction mixture was collected, concentrated in vacuo and purified as described.

***(Z)-4-(2-cyano-2-(3,4-dichlorophenyl)vinyl)-N-methylbenzamide (1a)***.

4-Formylbenzoic acid (0.300 g, 2 mmol) and oxalyl chloride (1.3 eq., 0.330 g, 2.6 mmol (1.3 mL 2.0 M DCM solution)) were combined with 15 mL DCM. DMF (1.5 mL) was added dropwise, the flask was stoppered and the reaction left to stir at ambient temperature until TLC analysis indicated reaction was complete (1 h). The solvent was removed in vacuo and the resultant brown oil was carried directly through to the next step.

4-Formylbenzoyl chloride (0.336 g, 2 mmol), K_2_CO_3_ (2 eq., 0.553 g, 4 mmol) and methylamine (1.2 eq., 210 µL 40 wt.% aqueous solution, 2.4 mmol) were combined with 5 mL diethyl ether and stirred vigorously at RT overnight. The reaction was quenched with 1 M HCl (10 mL), diluted with diethyl ether (30 mL), washed with sat. NaHCO_3_ (3 × 20 mL), brine (20 mL), dried over MgSO_4_ and the solvent removed in vacuo.

The desired product ((*Z*)-4-(2-cyano-2-(3,4-dichlorophenyl)vinyl)-*N*-methylbenzamide, **1a**) was subsequently prepared according to general procedure 1, from the freshly prepared 4-formyl-*N*-methylbenzamide (1.05 eq., 95 mg, 0.56 mmol) and 3,4-dichlorophenyl acetonitrile (103 mg, 0.55 mmol). The crude solid was taken up in 40 mL 2:1 diethyl ether:EtOAc, washed with water (3 × 20 mL), 1 M NaOH (3 × 10 mL) and brine (2 × 20 mL). The organic extract was dried over MgSO_4_ and the solvent removed in vacuo to afford the desired product as a tan solid (148 mg, 81%), m.p.: 222–225 °C. ^1^H NMR (600 MHz, DMSO-*d_6_*) δ 8.58 (q, *J* = 4.2 Hz, NH), 8.25 (s, 1H), 8.08 (d, *J* = 2.2 Hz, 1H), 8.00 (d, *J* = 8.5 Hz, 2H), 7.97 (d, *J* = 8.5 Hz, 2H), 7.82 (d, *J* = 8.5 Hz, 1H), 7.75 (dd, *J* = 8.5, 2.3 Hz, 1H), 2.81 (d, *J* = 4.5 Hz, 3H); ^13^C NMR (151 MHz, DMSO-*d_6_*) δ 165.8, 143.9, 136.3, 135.7, 134.3, 132.1, 132.0, 131.3, 129.2 (2C), 127.6 (2C), 127.5, 126.3, 117.2, 109.3, 26.3; IR υ_max_/cm^−1^: 3314 (NH), 2931 (C=C), 2216 (CN), 1634 (C=O), 816 (C-Cl); LRMS (ESI^+^) *m*/*z*: 331 (C_13_H_13_Cl_2_N_2_O_2_) [M+H]; HRMS: Exact mass calculated for C_13_H_13_Cl_2_N_2_O_2_ [M+H], 331.0339.

***(Z)-3-(2-aminophenyl)-2-(3,4-dichlorophenyl)acrylonitrile (1f).***

(*Z*)-2-(3,4-Dichlorophenyl)-3-(2-nitrophenyl)acrylonitrile (**2c**, 1.239 g, 3.88 mmol) and iron powder (9 equiv, 1.517 g, 27.16 mmol) were combined with a mixture of glacial acetic acid (4 mL), ethanol (4 mL), and water (2 mL) and exposed to ultrasonic irradiation at 30 °C for 7 h. The reaction mixture was diluted with ethanol (30 mL) and filtered through a pad of Celite, before concentration under reduced pressure to afford the desired product as an off-white solid (650 mg, 58%), m.p.: 157–159 °C. ^1^H NMR (400 MHz, acetone-*d_6_*): δ 7.92 (s, 1H), 7.77 (d, *J* = 1.2 Hz, 1H), 7.73 (d, *J* = 5.2 Hz, 1H), 7.70 (d, *J* = 5.6 Hz, 1H), 7.60 (d, *J* = 5.6 Hz, 1H), 7.56 (m, 2H), 7.26 (td, *J* = 9.2, 4.8, 0.4 Hz, 1H); ^13^C NMR (101 MHz, acetone-*d_6_*): δ 173.0, 156.5, 148.3, 139.4, 138.5, 133.2, 132.3, 132.0, 132.0, 130.7, 130.1, 128.7, 124.6, 123.7, 123.2; IR υ_max_/cm^−1^: 3073 (NH_2_), 2970 (NH_2_), 2219 (CN), 821 (C-Cl); LRMS (ESI^−^) *m*/*z*: 288 (C_15_H_9_Cl_2_N_2_) [M-H]; HRMS: Exact mass calculated for C_15_H_11_Cl_2_N_2_ [M+H], 289.0924.

***(Z)-2-(3,4-Dichlorophenyl)-3-(4-(oxiran-2-ylmethoxy)phenyl)-acrylonitrile (6)*** [21].

4-Hydroxybenzaldehyde (**3z**) (305 mg, 2.5 mmol) in 25 mL acetonitrile was passed through a series of Omnifit columns, the first containing molecular sieves, the second contained cesium carbonate and sand (50%:50% *w/w*) at a flow rate of 0.5 mL/min. The reactant stream was then met by a secondary stream containing epichlorohydrin (17 mL, with 20 mL DMF and 63 mL acetonitrile, flow rate 0.5 mL/min), and together the reactant streams were passed through a Vapourtec static mixer reactor (20mL) heated at 105 °C and held at 5 bar back pressure. At the completion of the reaction, the product solution was evaporated under reduced pressure, diluted with ethyl acetate (50 mL), washed with water (3 × 50mL), and then brine (1 × 50 mL), dried over MgSO_4_ and the solvent removed under reduced pressure to afford 4-(oxiran-2-ylmethoxy)benzaldehyde (**5**) as a light brown oil (364 mg, 82%).

The desired product ((*Z*)-2-(3,4-dichlorophenyl)-3-(4-(oxiran-2-ylmethoxy)phenyl)-acrylonitrile (**6**)) was then prepared according to general procedure 1 from the freshly prepared (4-(oxiran-2-ylmethoxy)benzaldehyde (**5**, 1.05 eq., 12.177 g, 68 mmol) and 3,4-dichlorophenylacetonitrile (**1a**, 12.109 g, 65 mmol) The crude solid was recrystallized from ethanol to afford the desired product (**6**) as a yellow solid (15.805 g, 70%), m.p.: 121–124 °C. ^1^H NMR (400 MHz, acetone-*d_6_*) δ 8.02 (d, *J* = 8.8 Hz, 2H), 7.97 (s, 1H), 7.94 (d, *J* = 1.4 Hz, 1H), 7.73–7.68 (m, 2H), 7.15 (d, *J* = 8.8 Hz, 2H), 4.47 (dd, *J* = 11.4, 2.6 Hz, 1H), 4.00 (dd, *J* = 11.4, 6.4 Hz, 1H), 3.36 (td, *J* = 6.6, 2.6 Hz, 1H), 2.87 (dd, *J* = 4.8, 4.4 Hz, 1H), 2.75 (dd, *J* = 5.2, 2.4 Hz, 1H); ^13^C NMR (101 MHz, acetone-*d_6_*) δ 162.0, 144.6, 136.4, 133.5, 132.8, 132.5 (2C), 132.0, 128.2, 127.5, 126.5, 118.5, 116.0 (2C), 106.6, 70.4, 50.4, 44.4; IR υ_max_/cm^−1^: 3063 (C=C), 2210 (CN), 1255 (arom. C-O), 811 (C-Cl); LRMS: (ESI^−^) *m*/*z*: 368 (C_18_H_12_Cl_2_NNaO_2_) [M+Na-H].

***(S,Z)-2-(3,4-Dichlorophenyl)-3-(4-(2-hydroxy-3-(piperidin-1-yl)propoxy)phenyl)acrylonitrile (8h)***.

4-Hydroxybenzaldehyde (**3z**, 1.071 g, 8.77 mmol), cesium carbonate (1.5 eq., 4.287 g, 13.2 mmol) and [*S*]-(+)-glycidyl-3-nitrobenzene sulfonate (1.1 eq., 2.500 g, 9.64 mmol) were combined with 30 mL DMF. Heated to 80 °C for 4 h. Reaction was diluted with water (50 mL) and EtOAc (30 mL), washed with water (5 × 30 mL) and brine (30 mL). The aqueous layer was extracted with EtOAc (3 × 30 mL). Organic extracts combined, dried over MgSO_4_ and concentrated under reduced pressure to afford the desired product as a pale brown oil. Yield not calculated and material carried directly through.

(*S*)-4-(oxiran-2-ylmethoxy)benzaldehyde (1.1 eq., 1.603 g, 8.77 mmol) was combined with 15 mL water and heated to 50 °C for 10 min while stirring. 3,4-Dichlorophenylacetonitrile (**2a**, 1.483 g, 7.97 mmol) was then added and the reaction stirred for a further 10 min. 10 mL Benzyltrimethylammonium hydroxide (40 wt % aq. solution) was added dropwise and the reaction left to stir at 50 °C for 6 h. Filtered under vacuum, washed with water (2 × 10 mL) and recrystallized from ethanol to afford the desired product as a yellow solid (1.7198 g, 62% over 2 steps).

The desired product (**8h**) was then prepared according to general procedure 3 from (*S,Z*)-2-(3,4-dichlorophenyl)-3-(4-(oxiran-2-ylmethoxy)phenyl)acrylonitrile (346 mg, 1 mmol) and piperidine (1.5 eq., 148 μL, 1.5 mmol) to afford the desired compound as a yellow solid (305 mg, 74%), m.p.: 100–102 °C. ^1^H NMR (600 MHz, DMSO*-d_6_*) δ 8.09 (s, 1H), 8.00 (d, *J* = 1.9 Hz, 1H), 7.96 (d, *J* = 8.7 Hz, 2H), 7.76 (d, *J* = 8.5 Hz, 1H), 7.68 (dd, *J* = 8.5, 1.9 Hz, 1H), 7.13 (d, *J* = 8.8 Hz, 2H), 4.86 (s, 1H), 4.10–4.07 (m, 1H), 4.00–3.95 (m, 2H), 2.41–2.31 (m, 5H), 1.50–1.47 (m, 4H), 1.37 (d, *J* = 4.8 Hz, 2H); ^13^C NMR (151 MHz, DMSO*-d_6_*) δ 161.2, 144.3, 134.9, 132.0, 131.5 (2C), 131.2, 131.1, 127.0, 125.76, 125.74, 117.9, 115.1 (2C), 104.3, 71.5, 66.4, 61.7, 54.8 (2C), 25.7 (2C), 24.0; IR υ_max_/cm^−1^: 3300 cm^−1^ (OH), 2933 cm^−1^ (N-CH_2_), 2216 cm^−1^ (CN), 1182 cm^−1^ (C-O), 813 cm^−1^ (C-Cl); LRMS: (ESI^+^) *m*/*z*: 431 (C_23_H_25_Cl_2_N_2_O_2_) [M+H]; HRMS: Exact mass calculated for C_23_H_25_Cl_2_N_2_O_2_ [M+H], 431.1288.

***(R,Z)-2-(3,4-Dichlorophenyl)-3-(4-(2-hydroxy-3-(piperidin-1-yl)propoxy)phenyl)acrylonitrile (8i).***

4-Hydroxybenzaldehyde (**3z**, 0.978 g, 8.00 mmol), cesium carbonate (1.5 eq., 3.910 g, 12.0 mmol) and [*R*]-(−)-glycidyl-3-nitrobenzene sulfonate (1.1 eq., 2.282 g, 8.80 mmol) were combined with 30 mL DMF. Heated to 80 °C for 4 h. Reaction was diluted with water (50 mL) and EtOAc (30 mL), washed with water (5 × 30 mL) and brine (30 mL). Aqueous back-extracted with EtOAc (3 × 30 mL). Organic extracts combined, dried over MgSO_4_ and concentrated under reduced pressure to afford the desired product as a pale brown oil (1.279 g, 90%).

(*R*)-4-(oxiran-2-ylmethoxy)benzaldehyde (1.05 eq., 1.511 g, 6.46 mmol) was combined with 15 mL water and heated to 50 °C for 10 min while stirring. 3,4-Dichlorophenylacetonitrile (**2a**, 1.144 g, 6.15 mmol) was then added, and the reaction stirred for a further 10 min. 10 mL benzyltrimethylammonium hydroxide (40 wt% aq. solution) was then added dropwise and the reaction left to stir at 50 °C for 6 h. Filtered under vacuum, washed with water (2 × 10 mL) and recrystallized from ethanol to afford the desired product as a yellow solid (1.5043 g, 71%).

The desired product (**8i**) was then prepared according to general procedure 3 from (R,*Z*)-2-(3,4-dichlorophenyl)-3-(4-(oxiran-2-ylmethoxy)phenyl)acrylonitrile (346 mg, 1 mmol) and piperidine (1.5 eq., 148 µL, 1.5 mmol) to afford the desired compound as a yellow solid (264 mg, 64%), m.p.: 101–103 °C. ^1^H NMR (600 MHz, DMSO*-d_6_*) δ 8.10 (s, 1H), 8.01 (d, *J* = 2.1 Hz, 1H), 7.96 (d, *J* = 8.8 Hz, 2H), 7.77 (d, *J* = 8.5 Hz, 1H), 7.69 (dd, *J* = 8.5, 2.1 Hz, 1H), 7.13 (d, *J* = 8.8 Hz, 2H), 4.86 (d, *J* = 3.3 Hz, 1H), 4.10–4.05 (m, 1H), 4.00–3.95 (m, 2H), 2.41–2.31 (m, 5H), 1.50–1.47 (m, 4H), 1.37 (d, *J* = 4.7 Hz, 2H); ^13^C NMR (151 MHz, DMSO*-d_6_*) δ 161.2, 144.3, 135.0, 132.0, 131.5 (2C), 131.2, 131.1, 127.0, 125.77, 125.75, 117.9, 115.1 (2C), 104.3, 71.5, 66.4, 61.7, 54.8 (2C), 25.7 (2C), 24.0; IR υ_max_/cm^−1^: 3366 cm^−1^ (OH), 2932 cm^−1^ (N-CH_2_), 2211 cm^−1^ (CN), 1182 cm^−1^ (C-O), 813 cm^−1^ (C-Cl); LRMS: (ESI^+^) *m*/*z*: 431 (C_23_H_25_Cl_2_N_2_O_2_) [M+H]; HRMS: Exact mass calculated for C_23_H_25_Cl_2_N_2_O_2_ [M+H], 431.1288.

***(Z)-2-(3,4-Dichlorophenyl)-3-(4-(2-hydroxy-3-(4-methylpiperazin-1-yl)propoxy)phenyl)acrylonitrile (8p)*** [15]**.**

Prepared according to general procedure 3 from (*Z*)-2-(3,4-dichlorophenyl)-3-(4-(oxiran-2-ylmethoxy)phenyl)acrylonitrile (**6**, 346 mg, 1 mmol) and 1-methyl-4-phenylpiperazine (1.5 eq., 264 mg, 1.5 mmol) to afford the desired compound as a bright yellow solid (448 mg, 89%), m.p.: 147–150 °C. ^1^H NMR (400 MHz, DMSO-*d_6_*) δ 8.10 (s, 1H), 8.00 (d, *J* = 1.9 Hz, 1H), 7.96 (d, *J* = 8.7 Hz, 2H), 7.76 (d, *J* = 8.5 Hz, 1H), 7.68 (dd, *J* = 8.5, 1.9 Hz, 1H), 7.14 (d, *J* = 8.7 Hz, 2H), 7.01 (d, *J* = 8.2 Hz, 2H), 6.81 (d, *J* = 8.4 Hz, 2H), 4.97 (d, *J* = 4.2 Hz, 1H), 4.12 (d, *J* = 6.7 Hz, 1H), 4.00 (d, *J* = 8.1 Hz, 2H), 3.06–3.05 (m, 4H), 2.64–2.56 (m, 4H), 2.43 (dd, *J* = 12.7, 5.6 Hz, 1H), 2.19 (s, 3H); ^13^C NMR (101 MHz, DMSO-*d_6_*) δ 161.2, 149.0, 144.3, 135.0, 132.0, 131.5 (2C), 131.2, 131.1, 129.4 (2C), 127.5, 127.0, 125.81, 125.78, 117.9, 115.6 (2C), 115.2 (C2), 104.4, 71.4, 66.5, 60.9, 53.5 (2C), 48.8 (2C), 20.0; IR υ_max_/cm^−1^: 3333 (OH), 2816 (N-CH_2_), 2208 (CN), 1182 (C-O), 817 (C-Cl); LRMS (ESI^+^) *m*/*z*: 522 (C_29_H_30_Cl_2_N_3_O_2_) [M+H]; HRMS: Exact mass calculated for C_29_H_30_Cl_2_N_3_O_2_ [M+H], 522.1710. Found 522.1713.

***(Z)-3-(4-(3-(4-(4-Chlorophenyl)piperazin-1-yl)-2-hydroxypropoxy)phenyl)-2-(3,4-dichlorophenyl)-acrylonitrile (8q)*** [15].

Prepared according to general procedure 3 from (*Z*)-2-(3,4-dichlorophenyl)-3-(4-(oxiran-2-ylmethoxy)phenyl)acrylonitrile (**6**, 200 mg, 0.58 mmol) and 1-(4-chlorophenyl)piperazine (2 eq., 227 mg, 1.16 mmol) to afford the desired compound as a cream colored solid (250 mg, 82%), m.p.: 159–162 °C. ^1^H NMR (400 MHz, DMSO-*d_6_*) δ 8.10 (s, 1H), 8.01 (d, *J* = 2.0 Hz, 1H), 7.96 (d, *J* = 8.8 Hz, 2H), 7.77 (d, *J* = 8.5 Hz, 1H), 7.69 (dd, *J* = 8.5, 2.1 Hz, 1H), 7.21 (d, *J* = 8.9 Hz, 2H), 7.14 (d, *J* = 8.8 Hz, 2H), 6.93 (d, *J* = 9.0 Hz, 2H), 4.99 (d, *J* = 2.6 Hz, 1H), 4.12–4.11 (m, 1H), 4.05–4.00 (m, 2H), 3.12 (t, *J* = 4.3 Hz, 4H), 2.65–2.57 (m, 4H), 2.44 (dd, *J* = 13.1, 4.4 Hz, 1H); ^13^C NMR (101 MHz, DMSO-*d_6_*) δ 161.2, 149.9, 144.3, 135.0, 132.1, 131.6 (2C), 131.24, 131.15, 128.6 (2C), 127.0, 125.83, 125.81, 122.3, 117.9, 116.8 (2C), 115.2 (2C), 104.4, 71.4, 66.5, 60.9, 53.3 (2C), 48.1 (2C); IR υ_max_/cm^−1^: 3355 (br, OH), 2830 (N-CH_2_), 2216 (CN), 1260 (C-O), 815 (C-Cl); LRMS: (ESI^+^) *m*/*z*: 542 (C_28_H_27_Cl_3_N_3_O_2_) [M+H); HRMS: Exact mass calculated for (C_28_H_27_Cl_3_N_3_O_2_ [M+H], 542.1163. Found 542.1147.

***(Z)-3-(4-(3-(4-(4-Bromophenyl)piperazin-1-yl)-2-hydroxypropoxy)phenyl)-2-(3,4-dichlorophenyl)acrylonitrile (8r)*** [15].

Prepared according to general procedure 3 from (*Z*)-2-(3,4-dichlorophenyl)-3-(4-(oxiran-2-ylmethoxy)phenyl)acrylonitrile (**6**, 200 mg, 0.58 mmol) and 1-(4-bromophenyl)piperazine (2 eq., 279 mg, 1.16 mmol) to afford the desired compound as a cream coloured solid (326 mg, 96%), m.p.: 162–165 °C. ^1^H NMR (400 MHz, DMSO-*d_6_*) δ 8.10 (s, 1H), 8.00 (s, 1H), 7.96 (d, *J* = 8.6 Hz, 2H), 7.76 (d, *J* = 8.6 Hz, 1H), 7.68 (d, *J* = 6.8 Hz, 1H), 7.33 (d, *J* = 8.8 Hz, 2H), 7.14 (d, *J* = 8.6 Hz, 2H), 6.88 (d, *J* = 8.8 Hz, 2H), 4.99 (s, 1H), 4.11 (d, *J* = 6.5 Hz, 1H), 4.01–3.99 (m, 2H), 3.12 (s, 4H), 2.63–2.56 (m, 4H), 2.46–2.41 (m, 1H); ^13^C NMR (101 MHz, DMSO-*d_6_*) δ 161.2, 150.2, 144.3, 134.9, 132.0, 131.5 (2C), 131.4 (2C), 131.2, 131.1, 127.0, 125.80, 125.77, 117.9, 117.2 (2C), 115.1 (2C), 109.9, 104.4, 71.4, 66.5, 60.9, 53.3 (2C), 47.9 (2C); IR υ_max_/cm^−1^: 3300 (OH), 2830 cm (N-CH_2_), 2216 (CN), 1259 cm (C-O), 814 cm (C-Cl); LRMS: (ESI^+^) *m*/*z*: 588 (C_28_H_27_BrCl_2_N_3_O_2_) [M+H]; HRMS: Exact mass calculated for (C_28_H_27_BrCl_2_N_3_O_2_) [M+H], 588.0638. Found 588.0617.

***(Z)-2-(3,4-Dichlorophenyl)-3-(4-(2-hydroxy-3-(4-(4-hydroxyphenyl)piperazin-1-yl)propoxy)-phenyl)acrylonitrile (8s).***

Prepared according to general procedure 3 from (*Z*)-2-(3,4-dichlorophenyl)-3-(4-(oxiran-2-ylmethoxy)phenyl)acrylonitrile (**6**, 346 mg, 1.0 mmol) and 1-(4-hydroxyphenyl)piperazine (1.5 eq., 267 mg, 1.5 mmol). The resultant residue was recrystallised from methanol to afford the desired compound as a brown solid (330 mg, 63%), m.p.: 162–164 °C. ^1^H NMR (600 MHz, DMSO-*d_6_*) δ 8.81 (bs, OH), 8.09 (s, 1H), 8.00 (d, *J* = 2.3 Hz, 1H), 7.96 (d, *J* = 8.9 Hz, 2H), 7.76 (d, *J* = 8.5 Hz, 1H), 7.68 (dd, *J* = 8.5, 2.3 Hz, 1H), 7.14 (d, *J* = 8.9 Hz, 2H), 6.77–6.76 (m, 2H), 6.65–6.63 (m, 2H), 4.97 (s, 1H), 4.12–4.10 (m, 1H), 4.02–3.98 (m, 2H), 2.95 (t, *J* = 4.7 Hz, 4H), 2.60 (ddd, *J* = 18.1, 10.4, 5.0 Hz, 4H), 2.42 (dd, *J* = 12.6, 6.1 Hz, 1H); ^13^C NMR (151 MHz, DMSO-*d_6_*) δ 161.2, 150.8, 144.3, 144.2, 134.9, 132.0, 132.5 (2C), 131.2, 131.1, 127.0, 125.79, 125.77, 117.9, 117.7 (2C), 115.4 (2C), 115.1 (2C), 104.4, 71.4, 66.5, 60.9, 53.7 (2C), 50.1 (2C); IR υ_max_/cm^−1^: 3366 (OH), 2212 (CN), 1370 (OH), 1182 (C-O), 815 (C-Cl); LRMS (ESI+) *m*/*z*: 524 (C_29_H_28_Cl_2_N_3_O_3_) [M+H]; HRMS: Exact mass calculated for (C_28_H_28_Cl_2_N_3_O_3_) [M+H], 524.1403. 

***(Z)-2-(3,4-Dichlorophenyl)-3-(4-(2-hydroxy-3-(4-(4-(trifluoromethyl)phenyl)piperazin-1-yl)propoxy)-phenyl)acrylonitrile (8t)*** [15].

Prepared according to general proceure 3 from (Z)-2-(3,4-dichlorophenyl)-3-(4-(oxiran-2-ylmethoxy)phenyl)acrylonitrile (6, 346 mg, 1 mmol) and 1-(4-trifluoromethylphenyl)piperazine (2 eq., 461 mg, 2 mmol) to afford the desired compound as a pale yellow solid (486 mg, 84%), m.p.: 159–161 °C. ^1^H NMR (400 MHz, DMSO-d_6_) δ 8.09 (s, 1H), 8.00 (d, J = 1.8 Hz, 1H), 7.96 (d, J = 8.7 Hz, 2H), 7.75 (d, J = 8.5 Hz, 1H), 7.68 (dd, J = 8.5, 1.8 Hz, 1H), 7.48 (d, J = 8.6 Hz, 2H), 7.14 (d, J = 8.7 Hz, 2H), 7.04 (d, J = 8.6 Hz, 2H), 5.00 (d, J = 3.3 Hz, 1H), 4.12–4.11 (m, 1H), 4.02–3.99 (m, 2H), 3.27 (s, 4H), 2.64–2.55 (m, 4H), 2.47–2.42 (m, 1H); ^13^C NMR (101 MHz, DMSO-d_6_) δ 161.2, 153.3, 144.3, 134.9, 132.1, 131.5, 131.2, 131.1, 127.0, 126.1 (q, J = 3.4 Hz, 2C), 125.83, 125.78, 125.0 (q, J = 272.7 Hz), 117.9, 117.8 (q, J = 32.2 Hz), 115.2 (2C), 114.1 (2C), 104.4, 71.4, 66.6, 60.9, 53.2 (2C), 47.1 (2C); ^19^F NMR (376 MHz, DMSO-d_6_, CF_3_CO_2_H) δ -56.1; IR υ_max_/cm^−1^: 3300 (OH), 2208 (CN), 1224 (C-F), 1179 (C-F), 1103 (C-O), 819 (C-Cl); LRMS (ESI+) *m*/*z*: 576 (C_29_H_27_Cl_2_F_3_N_3_O_2_) [M+H]; HRMS: Exact mass calculated for (C_29_H_27_Cl_2_F_3_N_3_O_2_) [M+H], 576.4442. Found 576.4311.

***(Z)-2-(3,4-Dichlorophenyl)-3-(4-(3-(4-(4-acetamidophenyl)piperazin-1-yl)-2-hydroxypropoxy)phenyl)-acrylonitrile (8u)*** [15]**.**

Prepared according to general procedure 3 from (*Z*)-2-(3,4-dichlorophenyl)-3-(4-(oxiran-2-ylmethoxy)phenyl)acrylonitrile (**6**, 193 mg, 0.56 mmol) and 4′-piperazineacetophenone (2 eq., 227 mg, 1.11 mmol) to afford the desired compound as an orange solid (240 mg, 78%), m.p.: 149–153 °C. ^1^H NMR (400 MHz, acetone-*d_6_*) δ 8.02 (dd, *J* = 8.8, 1.6 Hz, 2H), 7.98 (s, 1H), 7.94 (d, *J* = 1.4 Hz, 1H), 7.86 (dd, *J* = 8.8, 1.6 Hz, 1H), 7.75–7.69 (m, 2H), 7.15 (dd, *J* = 8.8, 1.6 Hz, 2H), 6.99 (dd, *J* = 8.8, 1.6 Hz, 2H) 4.24 (dd, *J* = 8.8, 3.6 Hz, 1H), 4.20–4.16 (m, 1H), 4.13 (dd, *J* = 8.9, 5.2 Hz, 1H), 3.41 (t, *J* = 5.1 Hz, 4H), 2.78–2.58 (m, 6H), 2.45 (s, 3H); ^13^C NMR (101 MHz, acetone-*d_6_*) δ 195.8, 162.5, 155.2, 144.7, 136.5, 133.5, 132.7, 132.5 (2C), 132.0, 130.9 (2C), 128.4, 128.2, 127.2, 126.5, 118.5, 116.0 (2C), 114.2 (2C), 106.3, 72.1, 67.6, 61.7, 54.3 (2C), 48.1 (2C), 26.1; IR υ_max_/cm^−1^: 3100 (br, OH), 2981 cm-1 (CH_3_), 2890 (N-CH_2_), 2210 (CN) 1660 (C=O), 1272 (C-O), 818 (C-Cl); LRMS: (ESI+) *m*/*z*: 550 (C_30_H_30_Cl_2_N_3_O_3_) [M+H]; HRMS: Exact mass calculated for C_30_H_30_Cl_2_N_3_O_3_ [M+H], 550.1659. Found 550.1671.

***(Z)-3-(4-(3-(4-(4-Nitrophenyl)piperazin-1-yl)-2-hydroxypropoxy)phenyl)-2-(3,4-dichlorophenyl)acrylonitrile (8v)*** [15].

Prepared according to general procedure 3 from (*Z*)-2-(3,4-dichlorophenyl)-3-(4-(oxiran-2-ylmethoxy)phenyl)acrylonitrile (**6**, 346 mg, 1 mmol) and 1-(4-nitrophenyl)piperazine (1.5 eq., 308 mg, 1.5 mmol) to afford the desired compound as a bright yellow solid (437 mg, 79%), m.p.: 90–92 °C. ^1^H NMR (600 MHz, DMSO-*d_6_*) δ 8.10 (s, 1H), 8.04 (d, *J* = 9.2 Hz, 2H), 8.00 (s, 1H), 7.96 (d, *J* = 8.5 Hz, 2H), 7.76 (d, *J* = 8.3 Hz, 1H), 7.68 (d, *J* = 8.3 Hz, 1H), 7.14 (d, *J* = 8.5 Hz, 2H), 7.02 (d, *J* = 9.2 Hz, 2H), 5.02 (d, *J* = 4.1 Hz, 1H), 4.12 (d, *J* = 6.8 Hz, 1H), 4.01 (dd, *J* = 16.0, 6.8 Hz, 2H), 3.45 (s, 4H), 2.63–2.54 (m, 5H), 2.45 (dd, *J* = 12.6, 6.0 Hz, 1H); ^13^C NMR (101 MHz, DMSO-*d_6_*) δ 161.2, 154.7, 144.3, 136.8, 134.9, 132.0, 131.5 (2C), 131.2, 131.1, 127.0, 125.8, 125.7 (2C), 117.9, 115.1 (2C), 112.6 (2C), 104.4, 71.3, 66.6, 60.7, 53.0 (2C), 46.4 (2C); IR υ_max_/cm^−1^: 3337 (OH), 2835 (N-CH_2_), 2212 (CN), 1589 (NO_2_), 1332 (NO_2_), 1244 (C-O), 827 (C-Cl); LRMS: (ESI^+^) *m*/*z*: 553 (C_28_H_27_Cl_2_N_4_O_4_) [M+H]; HRMS: Exact mass calculated for (C_28_H_27_Cl_2_N_4_O_4_) [M+H], 553.1404. Found 553.1408.

***(Z)-2-(3,4-Dichlorophenyl)-3-(4-(3-(4-(2-fluorophenyl)piperazin-1-yl)-2-hydroxypropoxy)phenyl)-acrylonitrile (8w)***[15].

Prepared according to general procedure 3 from (*Z*)-2-(3,4-dichlorophenyl)-3-(4-(oxiran-2-ylmethoxy)phenyl)acrylonitrile (**6**, 200 mg, 0.58 mmol) and 1-(2-fluorophenyl)piperazine (2 eq., 208 mg, 1.16 mmol) to afford the desired compound as a pale yellow solid (225 mg, 74%), m.p.: 119–121 °C. ^1^H NMR (400 MHz, acetone-*d_6_*) δ 8.02 (d, *J* = 8.8 Hz, 2H), 7.98 (s, 1H), 7.94 (d, *J* = 1.6 Hz, 1H), 7.73–7.67 (m, 2H), 7.15 (d, *J* = 8.8, Hz, 2H), 7.11–7.01 (m, 3H), 6.98–6.93 (m, 1H) 4.23 (dd, *J* = 9.1, 3.4 Hz, 1H), 4.21–4.16 (m, 1H), 4.12 (dd, *J* = 9.1, 5.3 Hz, 1H), 3.12 (t, *J* = 4.8 Hz, 4H), 2.82–2.59 (m, 6H); ^13^C NMR (101 MHz, acetone-*d_6_*) δ 162.6, 156.5 (d, *J* = 245.4 Hz), 144.7, 141.23 (d, *J* = 8.5 Hz), 136.5, 133.5, 132.7, 132.5 (2C), 132.0, 128.2, 127.2, 126.5, 125.5 (d, *J* = 3.0 Hz), 123.1 (d, *J* = 8.1 Hz), 120.0 (d, *J* = 3.0 Hz), 118.5, 116.7 (d, *J* = 21.2 Hz), 116.0 (2C), 106.3, 72.2, 67.5, 61.8, 54.6 (2C), 51.36 (d, *J* = 3.3 Hz, 2C); ^19^F NMR (376 MHz, acetone-*d_6_*, CF_3_CO_2_H) δ -124.4; IR υ_max_/cm^−1^: 3200 (br, OH), 2825 (N-CH_2_), 2214 (CN), 1262 (C-O), 818 (C-Cl); LRMS: (ESI^+^) *m*/*z*: 526 (C_28_H_27_Cl_2_FN_3_O_2_) [M+H]; HRMS: Exact mass calculated for C_28_H_27_Cl_2_FN_3_O_2_ [M+H], 526.1459. Found 526.1465.

***(Z)-3-(4-(3-(4-Cyclohexylpiperazin-1-yl)-2-hydroxypropoxy)-phenyl)-2-(3,4-dichlorophenyl)-acrylonitrile (8x)* [15].**

Prepared according to general procedure 3 from (*Z*)-2-(3,4-dichlorophenyl)-3-(4-(oxiran-2-ylmethoxy)phenyl)acrylonitrile (**6**, 346 mg, 1 mmol) and 4-phenylpiperidine (2 eq., 340 mg, 2 mmol) to afford the desired compound as a pale yellow solid (448 mg, 87%), m.p.: 170–172 °C. ^1^H NMR (400 MHz, DMSO-*d_6_*) δ 8.10 (s, 1H), 8.01 (d, *J* = 2.2 Hz, 1H), 7.97 (d, *J* = 8.8 Hz, 2H), 7.76 (d, *J* = 8.5 Hz, 1H), 7.68 (dd, *J* = 8.5, 2.2 Hz, 1H), 7.30–7.22 (m, 4H), 7.18–7.14 (m, 3H), 4.91 (d, *J* = 4.1 Hz, 1H), 4.13–4.11 (m, 1H), 4.00–3.98 (m, 2H), 3.00 (dd, *J* = 26.9, 10.9 Hz, 2H), 2.49–2.35 (m, 3H), 2.12 (dt, *J* = 11.3, 8.4 Hz, 2H), 1.70–1.60 (m, 4H); ^13^C NMR (101 MHz, DMSO-*d_6_*) δ 161.2, 146.3, 144.3, 135.0, 132.0 (2 overlapping signals), 131.5 (2C), 131.2, 131.1, 128.3 (2C), 127.0, 126.7 (2C), 126.0, 125.8, 117.9, 115.2 (2C), 104.3, 71.5, 66.6, 61.3, 54.6 (d, *J* = 20.8 Hz, 2C), 41.8, 33.2 (d, *J* = 3.0 Hz, 2C); IR υ_max_/cm^−1^: 3422 (OH), 2808 (N-CH_2_), 2207 (CN), 1263 (C-O), 810 (C-Cl); LRMS: (ESI^+^) *m*/*z*: 507 (C_29_H_29_Cl_2_N_2_O_2_) [M+H]; HRMS: Exact mass calculated for C_29_H_29_Cl_2_N_2_O_2_ [M+H], 507.1601. Found 507.1620.

## Figures and Tables

**Table 1:** Percentage inhibition of bacteria and yeast at 32 μg/mL on treatment with *Library A* compounds **1a**–**1m**.

**Structure** 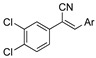	*S. aureus* ^a^	*E. coli* ^b^	*K. pneumoniae* ^c^	*P. aeruginosa* ^d^	*A. baumannii* ^e^	*C. albicans* ^f^	*C. neoformans* ^g^
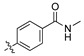 **1a**	14.2	<10	<10	22.3	<10	14.7	<10
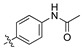 **1b**	16.2	<10	<10	11.4	13.9	11.8	10.7
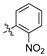 **1c**	13.2	<10	10.5	16.2	<10	<10	<10
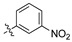 **1d**	13.5	<10	13.6	19.0	15.0	<10	<10
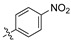 **1e**	15.9	<10	12.6	27.8	<10	11.8	<10
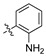 **1f**	<10	<10	17.1	14.6	13.2	10	22.3
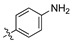 **1g**	<10	<10	11.9	10.4	37.0	17.2	<10
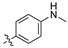 **1h**	13.6	<10	<10	23.4	10	<10	<10
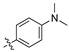 **1i**	<10	<10	<10	16.6	<10	12.2	<10
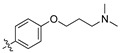 **1j**	<10	<10	10.2	18.5	27.5	<10	<10
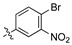 **1k**	12.7	<10	17.5	17.7	10.3	<10	10.3
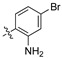 **1l**	10	<10	<10	21.6	<10	<10	<10
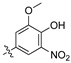 **1m**	14.9	<10	<10	13.0	<10	63.6	10

^a^ *S. aureus* MRSA (ATCC 43300); ^b^ *E. coli* FDA control (ATCC 25922); ^c^ *K. pneumoniae* K6/ESBL (ATCC 700603); ^d^ *P. aeruginosa* (ATCC 27853); ^e^ *A. baumannii* (ATCC 19606); ^f^ *C. albicans* CLSI reference (ATCC 90028); ^g^ *C. neoformans var. grubii H99* type strain (ATCC 208821).

**Table 2:** Percentage inhibition of bacteria and yeast growth at 32 μg/mL on treatment with *Library B* compounds **1n**–**1y**.

**Structure** 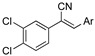	*S. aureus* ^a^	*E. coli* ^b^	*K. pneumoniae* ^c^	*P. aeruginosa* ^d^	*A. baumannii* ^e^	*C. albicans* ^f^	*C. neoformans* ^g^
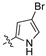 **1n**	<10	<10	<10	17.5	<10	10	<10
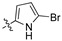 **1o**	<10	<10	10.1	17.6	<10	37.1	<10
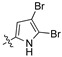 **1p**	32.0	<10	19.0	22.8	<10	10.3	<10
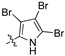 **1q**	24.4	<10	14.0	20.9	<10	10	<10
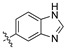 **1r**	13.8	<10	<10	26.8	14.5	16.6	<10
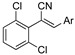							
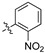 **1s**	<10	<10	<10	11.1	15.9	<10	23.6
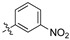 **1t**	<10	<10	<10	11.3	19.7	<10	28.6
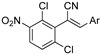							
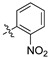 **1u**	12.9	<10	<10	13.2	14.3	<10	25.9
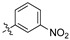 **1v**	16.1	<10	16.3	20.1	12.0	10.1	<10
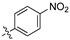 **1w**	22.7	<10	<10	<10	<10	<10	21.8
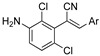							
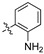 **1x**	<10	<10	11.01	10.4	<10	<10	18.5
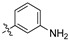 **1y**	<10	<10	21.3	17.3	48.2	<10	23.8

^a^ *S. aureus* MRSA (ATCC 43300); ^b^ *E. coli* FDA control (ATCC 25922); ^c^ *K. pneumoniae* K6/ESBL (ATCC 700603); ^d^ *P. aeruginosa* (ATCC 27853); ^e^ *A. baumannii* (ATCC 19606); ^f^ *C. albicans* CLSI reference (ATCC 90028); ^g^ *C. neoformans var. grubii H99* type strain (ATCC 208821).

**Table 3:** Percentage inhibition of bacteria and yeast at 32 μg/mL on treatment with *Library C* compounds **6**, **8a**–**8d**.

**Structure** 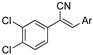	*S. aureus* ^a^	*E. coli* ^b^	*K. pneumoniae* ^c^	*P. aeruginosa* ^d^	*A. baumannii* ^e^	*C. albicans* ^f^	*C. neoformans* ^g^
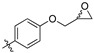 **6**	<10	<10	<10	24.5	<10	<10	<10
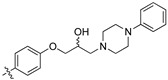 **8a**	14.5	<10	17.1	22.7	11.6	<10	<10
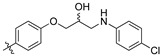 **8b**	<10	<10	<10	<10	<10	14.1	<10
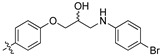 **8c**	<10	<10	<10	11.7	19.3	10	<10
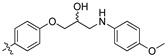 **8d**	<10	<10	10	23.2	<10	17.3	<10

^a^ *S. aureus* MRSA (ATCC 43300); ^b^ *E. coli* FDA control (ATCC 25922); ^c^ *K. pneumoniae* K6/ESBL (ATCC 700603); ^d^ *P. aeruginosa* (ATCC 27853); ^e^ *A. baumannii* (ATCC 19606); ^f^ *C. albicans* CLSI reference (ATCC 90028); ^g^ *C. neoformans var. grubii H99* type strain (ATCC 208821).

**Table 4:** Percentage inhibition of bacteria and yeast at 32 μg/mL on treatment with *Library D* compounds **8e**–**8l**.

**Structure** 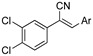	*S. aureus* ^a^	*E. coli* ^b^	*K. pneumoniae* ^c^	*P. aeruginosa* ^d^	*A. baumannii* ^e^	*C. albicans* ^f^	*C. neoformans* ^g^
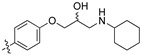 **8e**	88.3	<10	13.1	20.8	13.8	11.3	<10
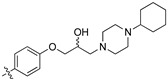 **8f**	54.9	<10	<10	<10	15.1	<10	<10
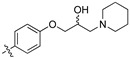 **8g**	67.2	<10	<10	22.3	<10	50.1	<10
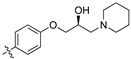 **8h**	92	<10	<10	13.2	<10	<10	<10
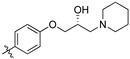 **8i**	90.3	<10	<10	26.5	11.8	13.3	<10
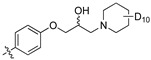 **8j**	90.1	<10	16.9	27.5	<10	19.1	<10
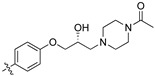 **8k**	16.2	<10	<10	24.4	<10	53.0	<10
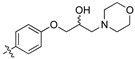 **8l**	15.9	<10	<10	21.5	<10	35.0	<10

^a^ *S. aureus* MRSA (ATCC 43300); ^b^ *E. coli* FDA control (ATCC 25922); ^c^ *K. pneumoniae* K6/ESBL (ATCC 700603); ^d^ *P. aeruginosa* (ATCC 27853); ^e^ *A. baumannii* (ATCC 19606); ^f^ *C. albicans* CLSI reference (ATCC 90028); ^g^ *C. neoformans var. grubii H99* type strain (ATCC 208821).

**Table 5:** Percentage inhibition of bacteria and yeast at 32 μg/mL on treatment with *Library E* compounds **8m**–**8x**.

**Structure** 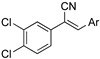	*S. aureus* ^a^	*E. coli* ^b^	*K. pneumoniae* ^c^	*P. aeruginosa* ^d^	*A. baumannii* ^e^	*C. albicans* ^f^	*C. neoformans* ^g^
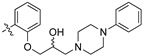 **8m**	<10	<10	<10	11.2	<10	<10	<10
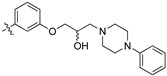 **8n**	<10	<10	<10	19.0	<10	<10	<10
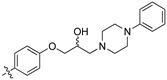 **8o**	11.6	<10	10.8	23.6	<10	12.5	<10
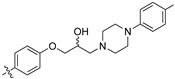 **8p**	12.8	<10	<10	16.1	<10	12.5	96.3
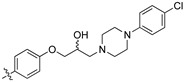 **8q**	<10	<10	<10	15.2	<10	<10	-<10
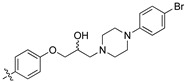 **8r**	15.3	<10	28.4	22.8	10	<10	<10
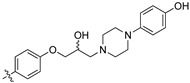 **8s**	13.9	<10	12.3	20.6	<10	64.6	97.1
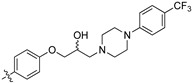 **8t**	12.8	<10	<10	10	<10	<10	<10
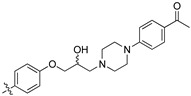 **8u**	10	<10	15.1	27.7	11.2	10.6	<10
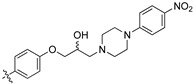 **8v**	12.6	<10	13.1	23.1	<10	<10	<10
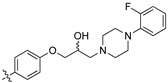 **8w**	<10	<10	<10	18.8	<10	11.7	<10
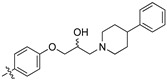 **8x**	<10	<10	10.3	16.0	<10	<10	98.4

^a^ *S. aureus* MRSA (ATCC 43300); ^b^ *E. coli* FDA control (ATCC 25922); ^c^ *K. pneumoniae* K6/ESBL (ATCC 700603); ^d^ *P. aeruginosa* (ATCC 27853); ^e^ *A. baumannii* (ATCC 19606); ^f^ *C. albicans* CLSI reference (ATCC 90028); ^g^ *C. neoformans var. grubii H99* type strain (ATCC 208821).

**Table 6:** Minimum inhibitory concentration (MIC) μg/mL of analogues **8e**, **h**, **i**, **j**, **q**, **s** and **x**.

**Structure** 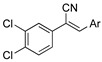	*S. aureus* ^a^	*E. coli* ^b^	*K. pneumoniae* ^c^	*P. aeruginosa* ^d^	*A. baumannii* ^e^	*C. albicans* ^f^	*C. neoformans* ^g^
Amoxicillin	16	8	>32	>32	>32	- ^h^	-
Colistin	32	8	>32	>32	>32	-	-
Amphotericin B	-	-	-	-	-	1.56	1.56
**8e**	32	-	-	-	-	-	-
**8h**	32	-	-	-	-	-	-
**8i**	32	-	-	-	-	-	-
**8j**	32	-	-	-	-	-	-
**8q**	-	-	-	-	-	-	-
**8s**	-	-	-	-	-	-	-
**8x**	-	-	-	-	-	-	32

^a^ *S. aureus* MRSA (ATCC 43300); ^b^ *E.coli* FDA control (ATCC 25922); ^c^ *K. pneumoniae* K6/ESBL (ATCC 700603); ^d^ *P. aeruginosa* (ATCC 27853); ^e^ *A. baumannii* (ATCC 19606); ^f^ *C. albicans* CLSI reference (ATCC 90028); ^g^ *C. neoformans var. grubii H99* type strain (ATCC 208821); ^h^ MIC > 32 μg/mL.

## Data Availability

All pertinent data has been supplied in the Supporting Information that accompanies this article.

## Supplementary Materials

The following supporting information can be downloaded at: https://www.mdpi.com/article/10.3390/molecules27072050/s1, synthesis detail for compounds generated; chiral HPLC chromatograms for **8g**, **8h** and **8i** and ^1^H and 13C NMR spectra for all compounds [36,37,38,39].

## Sample Availability

Samples of the compounds are available from the authors.
