# Supplementary material for: Amino Alcohols as Potential Antibiotic and Antifungal Leads
Source: Molecules. 2022 Mar 22;27(7):2050. doi: 10.3390/molecules27072050 (PMC9000800; doi:10.3390/molecules27072050)
Supplement: Supplementary file 1 [file molecules-27-02050-s001.zip › molecules-1550228-supplementary.pdf]

Amino alcohols as potential antibiotic and antifungal leads

Jennifer R. Baker,[1] Peter J. Cossar,[1] Mark A. T. Blaskovich,[2] Alysha G. Elliott,[2] Johannes Zuegg,[2] Matthew A. Cooper,[2] Peter J. Lewis,[3] Adam McCluskey[1]*

[1] Chemistry, School of Environmental & Life Sciences, The University of Newcastle, University Drive Callaghan NSW 2308, Australia. E-mail: Adam.McCluskey@newcastle.edu.au

[2] Community for Open Antimicrobial Drug Discovery, Centre for Superbug Solutions, Institute for Molecular Bioscience, The University of Queensland, Brisbane, Queensland 4072, Australia.

[3] Biology, School of Environmental & Life Sciences, The University of Newcastle, University Drive Callaghan NSW 2308, Australia.

[1] Br JR Baker (0000-0002-9560-301X), Dr PJ Cossar (0000-0002-8260-5710), Prof A McCluskey (0000-0001-7125-863X)

[2] Prof P Lewis (0000-0002-1992-062X)

[3] Dr M A T Blaskovich (0000-0001-9447-2292), Dr A G Elliott (0000-0002-2983-0484), Dr J Zuegg (0000-0001-6240-6020), Prof MA Cooper 0000-0003-3147-3460)

Experimental details for synthesised compounds [1–5]:

General Procedure 1: - The aldehyde (1.05 eq., 1.05 mmol), was added to a vigorously stirred solution of water (10 mL) and heated to 50 °C. The dichlorophenylacetonitrile (1 mmol) was then slowly added forming a suspension. After 5-10 minutes of stirring, 40% $PhCH_2NMe_3(OH)$ (7 mL) was added dropwise. After complete addition, the reaction vessel stirred at 50 °C for 5 hours. After this period, the solution was filtered hot, washed with warm water and purified by either recrystallisation or column chromatography.

General Procedure 2: - (*Z*)-2-(3,4-dichlorophenyl)-3-(4-(oxiran-2-ylmethoxy)phenyl)acrylonitrile (**15**, 1 eq.) was combined with the required amine (either 1.5 or 2 eq., as stated) and 20 mL ethanol. The solution was irradiated at 120 °C for 20 min. Upon chilling, the desired product was isolated via vacuum filtration.

General Procedure 3: -: Using a Vapourtec RS-400 equipped with fraction collection kit and auto-sampler, a 2.0 mL sample loop was charged with a 0.4 M solution of epoxide (**15**) in toluene. An additional 2 mL sample loop was charged with a 2.8 M amine solution in ethanol (as stated). The solutions were flowed together and the resulting stream was then passed through two PFA coil reactors in series at 150 °C, 10 bar back pressure and 0.5 $mL.min^{-1}$ (residence time 40 min). The resulting reaction mixture was collected, concentrated in vacuo and purified as described.

(Z)-N-(4-(2-cyano-2-(3,4-dichlorophenyl)vinyl)phenyl)acetamide (1b) Prepared according to general procedure 1 as previously described,[2] m.p.: 275-277 °C. ^{1}H NMR (600 MHz, DMSO-*d$_6$*) δ 10.30 (s, 1H), 8.09 (s, 1H), 8.02 (s, 1H), 7.93 (d, *J* = 7.9 Hz, 2H), 7.78 – 7.74 (m, 3H), 7.70 (d, *J* = 7.8 Hz, 1H), 2.09 (s, 3H); ^{13}C NMR (151 MHz, DMSO-*d$_6$*) δ 168.9, 144.2, 142.0, 134.8, 132.1, 131.3, 131.2, 130.5 (2C), 127.8, 127.1, 125.9 (2C), 118.8, 117.8, 105.3, 24.2; IR ν_{max}/cm^{-1}: 3335 (NH), 3033 (C=C), 2221 (CN), 1686 (C=O), 815 (C-Cl); LRMS (ESI$^+$) m/z: 331 [M+H].

(Z)-2-(3,4-dichlorophenyl)-3-(2-nitrophenyl)acrylonitrile (1c) Prepared according to general procedure 1 as previously described,[2] m.p.: 172-174 °C. ^{1}H NMR (600 MHz, acetone-*d$_6$*) δ 8.43 (s, 1H), 8.32 (d, *J* = 8.2 Hz, 1H), 8.03 (d, *J* = 7.6 Hz, 1H), 7.99 (s, 1H), 7.97 (d, *J* = 7.5 Hz, 1H), 7.83 (t, *J* = 7.8 Hz, 1H), 7.78 (s, 2H); ^{13}C NMR (151 MHz, acetone-*d$_6$*) δ 148.7, 142.9, 135.3, 134.9, 134.1, 133.8, 132.3, 132.2, 131.9, 130.8, 128.9, 127.1, 126.1, 116.7, 114.2; IR ν_{max}/cm^{-1}: 3073 (C=C), 2223 (CN), 1521 (NO_2), 1341 (NO_2), 721 (C-Cl); LRMS (ESI$^+$) m/z: 341 [M+Na].

(Z)-2-(3,4-dichlorophenyl)-3-(3-nitrophenyl)acrylonitrile (1d) Prepared according to general procedure 1 as previously described,[2] m.p.: 217-218 °C. ^{1}H NMR (600 MHz, acetone-*d$_6$*) δ 8.85 (s, 1H), 8.44 (d, *J* = 7.8 Hz, 1H), 8.39 (dd, *J* = 8.2, 1.9 Hz, 1H), 8.28 (s, 1H), 8.06 (d, *J* = 2.2 Hz, 1H), 7.89 (t, *J* = 8.0 Hz, 1H), 7.81 (dd, *J* = 8.5, 2.2 Hz, 1H), 7.77 (d, *J* = 8.5 Hz, 1H); ^{13}C NMR (101 MHz, DMSO-*d$_6$*) δ 148.0, 142.3, 135.0, 134.9, 133.9, 132.4, 132.2, 131.4, 130.7, 127.6, 126.4, 125.2, 124.0, 116.8, 110.6; IR ν_{max}/cm^{1}: 3086 (C=C), 2200 (CN), 1526 (NO_2), 1352 (NO_2), 734 (C-Cl); LRMS (ESI$^+$) m/z: 319 [M+H].

(Z)-2-(3,4-dichlorophenyl)-3-(4-nitrophenyl)acrylonitrile (1e) Prepared according to general procedure 1 as previously described,[3] m.p.: 158-161 °C. ^{1}H NMR (400 MHz, acetone-*d$_6$*) δ 8.42 (dt, *J* = 8.8, 2.0 Hz, 2H), 8.25 – 8.22 (m, 3H), 8.05 (d, *J* = 2.1 Hz, 1H), 7.80 (m, 2H); ^{13}C NMR (101 MHz, acetone-*d$_6$*) δ 148.8, 140.3, 139.1, 134.8, 134.0, 133.5, 131.4, 130.3 (2C), 128.2, 125.6, 124.4 (2C), 116.5, 113.9; IR ν_{max}/cm^{1}: 3086 (C=C), 2210 (CN), 1592 (NO_2), 1345 (NO_2), 747 (C-Cl); LRMS (ESI$^+$) m/z: 319 [M+H].

(Z)-3-(4-aminophenyl)-2-(3,4-dichlorophenyl)acrylonitrile (1g) Prepared according to general procedure 1 as previously described,[2] m.p.: 177-180 °C. ^{1}H NMR (400 MHz, DMSO-*d$_6$*) δ 7.91 (d, *J* = 1.9 Hz, 1H), 7.87 (s, 1H), 7.76 (d, *J* = 8.6 Hz, 2H), 7.70 (d, *J* = 8.5 Hz, 1H), 7.61 (dd, *J* = 8.6, 2.0 Hz, 1H), 6.65 (d, *J* = 8.6 Hz, 2H), 6.17 (s, NH_2); ^{13}C NMR (101 MHz, DMSO-*d$_6$*) δ 152.5, 144.9, 135.9, 132.0 (2C), 131.9, 131.1, 129.9, 126.3, 125.1, 120.4, 118.9, 113.4 (2C), 98.7; IR ν_{max}/cm^{-1}: 3489 (NH_2), 3373 (NH_2), 2206 (CN), 1619 (NH) 821 (C-Cl); LRMS (ESI$^-$) m/z: 288 [M-H].

(Z)-2-(3,4-dichlorophenyl)-3-(4-(methylamino)phenyl)acrylonitrile (1h) Prepared according to general procedure 1 as previously described,[2] m.p.: 140-143 °C. ^{1}H NMR (400 MHz, DMSO-*d$_6$*) δ 7.91 (d, *J* = 2.0 Hz, 1H), 7.90 (s, 1H), 7.83 (d, *J* = 8.8 Hz, 2H), 7.70 (d, *J* = 8.5 Hz, 1H), 7.61 (dd, *J* = 8.5, 2.1 Hz, 1H), 6.74 (q, *J* = 4.7 Hz, NH), 6.65 (d, *J* = 8.8 Hz, 2H), 2.77 (d, *J* = 5.0 Hz, 3H); ^{13}C NMR (101 MHz, DMSO-*d$_6$*) δ 152.7, 144.9, 135.9, 131.9, 131.1 (2C), 129.9, 126.3, 125.1, 120.3, 118.9 (2C), 98.6, 29.2; IR ν_{max}/cm^{-1}: 3389 (NH), 2200 (CN), 1611 (NH) 807 (C-Cl); LRMS (ESI$^+$) m/z: 303 [M+H].

(Z)-2-(3,4-dichlorophenyl)-3-(4-(dimethylamino)phenyl)acrylonitrile (1i) Prepared according to general procedure 1 as previously described,[2] m.p.: 205-208 °C. ^{1}H NMR (400 MHz, DMSO-*d$_6$*) δ 7.96 (s, 1H), 7.94 (d, *J* = 2.2 Hz, 1H), 7.90 (d, *J* = 9.0 Hz, 2H), 7.72 (d, *J* = 8.5 Hz, 1H), 7.64 (dd, *J* = 8.5, 2.2 Hz, 1H), 6.83 (d, *J* = 9.0 Hz, 2H), 3.04 (s, 6H); ^{13}C NMR (101 MHz, DMSO-*d$_6$*) δ 152.1, 144.6, 135.8, 131.9, 131.6 (2C), 131.1, 130.0, 126.3, 125.2, 120.4, 118.8, 111.6 (2C), 99.4, 39.6 (2C); IR ν_{max}/cm^{-1}: 2910 (C=C), 2210 (CN), 806 (C-Cl); LRMS (ESI$^+$) m/z: 317 [M+H].

(Z)-2-(3,4-Dichlorophenyl)-3-(4-(3-(dimethylamino)propoxy)-phenyl)acrylonitrile (1j) Prepared according to general procedure 1 as previously described,[4] m.p.: 89-92 °C. ^{1}H NMR (400 MHz, acetone-*d$_6$*) δ 8.01 (d, *J* = 8.8 Hz, 2H), 7.97 (s,

1H), 7.94 (d, *J* = 1.6 Hz, 1H), 7.71 – 7.70 (m, 2H), 7.10 (d, *J* = 8.9 Hz, 2H), 4.16 (t, *J* = 6.4 Hz, 2H), 2.42 (t, *J* = 7.0 Hz, 2H), 2.18 (s, 6H), 1.93 (dd, *J* = 13.4, 6.7 Hz, 2H); ^{13}C NMR (101 MHz, acetone-*d$_6$*) δ 162.6, 144.8, 136.5, 133.9, 132.5 (2C), 132.0, 130.0, 128.2, 127.0, 126.5, 118.5, 115.8 (2C), 106.1, 67.2, 56.7, 45.7 (2C), 28.1; IR ν_{max}/cm^{-1}: 2953 (C=H), 2216 (CN), 1248 (C-O), 727 (C-Cl); LRMS: (ESI$^+$) m/z: 375 ($C_{20}H_{20}Cl_2N_2O$) [M+H].

(Z)-3-(4-bromo-3-nitrophenyl)-2-(3,4-dichlorophenyl)acrylonitrile (1k) Prepared according to general procedure 1 as previously described,[2] m.p.: 189-192 °C. ^{1}H NMR (400 MHz, acetone-*d$_6$*) δ 8.50 (d, *J* = 2.1 Hz, 1H), 8.22 (dd, *J* = 8.5, 2.1 Hz, 1H), 8.19 (s, 1H), 8.10 (d, *J* = 8.5 Hz, 1H), 8.03 (d, *J* = 2.0 Hz, 1H), 7.81 – 7.75 (m, 2H); ^{13}C NMR (101 MHz, acetone-*d$_6$*) δ 141.5, 136.6, 135.5, 135.1, 134.2, 134.1, 133.9, 133.8, 132.3, 128.8, 127.2, 127.1, 117.2, 116.3, 113.1; IR ν_{max}/cm^{-1}: 3096 (C=C), 2222 (CN), 1531 (NO_2), 1338 (NO_2), 734 (C-Cl); LRMS (ESI$^+$) m/z: 399 [M+H].

(Z)-3-(2-amino-4-bromophenyl)-2-(3,4-dichlorophenyl)acrylonitrile (1l) Prepared according to general procedure 1 as previously described,[2] m.p.: 215-217 °C. ^{1}H NMR (600 MHz, acetone-*d$_6$*) δ 7.91 (s, 1H), 7.76 (d, *J* = 2.0 Hz, 1H), 7.74 (d, *J* = 1.7 Hz, 1H), 7.71 (d, *J* = 8.2 Hz, 1H), 7.67 (d, *J* = 8.5 Hz, 1H), 7.55 (dd, *J* = 8.2, 2.0 Hz, 1H), 7.35 (dd, *J* = 8.5, 1.9 Hz, 1H), 5.94 (bs, NH_2); ^{13}C NMR (151 MHz, acetone-*d$_6$*) δ 157.2, 149.9, 139.3, 138.0, 133.3, 132.4, 132.0, 131.9, 130.3, 130.0, 128.6, 126.0, 124.1, 124.0, 123.4; IR ν_{max}/cm^{-1}: 3473 (NH_2), 3291 (C=C), 1644 (NH_2), 778 (C-Cl); LRMS (ESI+) m/z: 369 [M+H].

(Z)-2-(3,4-dichlorophenyl)-3-(4-hydroxy-3-methoxy-5-nitrophenyl)acrylonitrile (1m) Prepared according to general procedure 1 as previously described,[2] m.p.: decomp >260 °C. ^{1}H NMR (600 MHz, DMSO-*d$_6$*) δ 8.07 (s, 1H), 7.88 (d, *J* = 35.2 Hz, 2H), 7.66 (d, *J* = 8.1 Hz, 1H), 7.58 (d, *J* = 7.5 Hz, 1H), 7.53 (s, 1H), 3.67 (s, 3H); ^{13}C NMR (151 MHz, DMSO-*d$_6$*) δ 163.7, 155.3, 144.9, 136.6, 135.6, 131.8, 130.9, 128.9, 128.0, 125.6, 124.6, 119.7, 111.0, 107.2, 95.0, 55.1; IR ν_{max}/cm^{-1}: 2970 (C=C), 2204 (CN), 1539 (NO_2), 1355 (NO_2), 782 (C-Cl) LRMS (ESI$^-$) m/z: 363 [M-H].

(Z)-3-(5-bromo-1H-pyrrol-2-yl)-2-(3,4-dichlorophenyl)acrylonitrile (1n) Prepared as previously described,[2] m.p.: decomp >135 °C. ^{1}H NMR (600 MHz, $CDCl_3$) δ 9.61 (s, NH), 7.64 (s, 1H), 7.48 (d, *J* = 8.4 Hz, 1H), 7.39 (dd, *J* = 8.4, 1.6 Hz, 1H), 7.23 (s, 1H), 6.65 (s, 1H), 6.34 – 6.33 (m, 1H); ^{13}C NMR (151 MHz, $CDCl_3$) δ 133.8, 133.7, 132.5, 131.2, 130.7, 128.8, 126.8, 124.3, 121.4, 119.8, 107.5, 99.8; IR ν_{max}/cm^{-1}: 3263 (NH), 2205 (CN), 1596 (C=C), 767 (C-Cl); LRMS (ESI$^+$) m/z: 341 [M+H].

(Z)-3-(4-bromo-1H-pyrrol-2-yl)-2-(3,4-dichlorophenyl)acrylonitrile (1o) Prepared as previously described,[2] m.p.: decomp >125 °C. ^{1}H NMR (600 MHz, $CDCl_3$) δ 7.91 (s, 1H), 7.63 (d, *J* = 2.1 Hz, 1H), 7.58 (d, *J* = 8.3 Hz, 1H), 7.36 (dd, *J* = 8.3, 2.1 Hz, 1H), 7.01 (s, 1H), 6.33 – 6.31 (m, 1H), 6.19 (dd, *J* = 3.8, 2.4 Hz, 1H); ^{13}C NMR (151 MHz, $CDCl_3$) δ 134.4, 134.3, 133.2, 132.9, 131.8, 130.8, 128.04, 127.96, 120.1, 119.9, 113.6, 106.1, 102.8; IR ν_{max}/cm^{-1}: 3256 (NH), 2213 (CN), 1585 (C=C), 773 (C-Cl); LRMS (ESI$^+$) m/z: 341 [M+H].

(Z)-3-(4,5-dibromo-1H-pyrrol-2-yl)-2-(3,4-dichlorophenyl)acrylonitrile (1p) Prepared as previously described,[2] m.p.: decomp >145 °C. ^{1}H NMR (600 MHz, acetone-d$_6$) δ 7.81 (d, *J* = 2.3 Hz, 1H), 7.71 (s, 1H), 7.69 (d, *J* = 8.5 Hz, 1H), 7.61 (dd, *J* = 8.5, 2.3 Hz, 1H), 7.33 (s, 1H); ^{13}C NMR (151 MHz, acetone-d$_6$) δ 135.6, 133.7, 132.7, 132.2, 131.9, 130.3, 127.7, 126.0, 118.3, 116.2, 107.2, 103.6, 102.4; IR ν_{max}/cm^{-1}: 3184 (NH), 2224 (CN), 1601 (C=C), 797 (C-Cl); LRMS (ESI+) m/z: 421 [M+H].

(Z)-2-(3,4-dichlorophenyl)-3-(3,4,5-tribromo-1H-pyrrol-2-yl)acrylonitrile (1q) Prepared as previously described,[2] m.p.: decomp >185 °C. ^{1}H NMR (600 MHz, DMSO-*d$_6$*) δ 13.02 (bs, NH), 8.00 (d, *J* = 2.2 Hz, 1H), 7.75 (d, *J* = 8.5 Hz, 1H), 7.68 (dd, *J* = 8.5, 2.2 Hz, 1H), 7.63 (s, 1H); ^{13}C NMR (151 MHz, DMSO-*d$_6$*) δ 133.9, 132.2, 131.7, 131.4, 130.9, 127.10, 127.05, 125.8, 117.0, 108.0, 106.5, 106.2, 103.4; IR ν_{max}/cm^{-1}: 3218 cm^{-1} (NH), 2215 cm^{-1} (CN), 1603 cm^{-1} (NH), 815 cm^{-1} (C-Cl) 734 cm^{-1} (C-Br); LRMS (ESI$^+$) m/z: 499 (M+H).

(Z)-3-(1H-benzo[d]imidazol-6-yl)-2-(3,4-dichlorophenyl)acrylonitrile (1r) Prepared according to general procedure 1 as previously described,[2] m.p.: 270-273 °C. ^{1}H NMR (600 MHz, DMSO-*d$_6$*) δ 12.82 (bs, NH), 8.39 (s, 1H), 8.32 – 8.30 (m, 2H), 8.06 (d, *J* = 1.7 Hz, 1H), 7.86 (d, J = 8.1 Hz, 1H), 7.79 (d, J = 8.4 Hz, 1H), 7.75 – 7.73 (m, 2H); ^{13}C NMR (151 MHz, DMSO-*d$_6$*) δ 145.9, 144.58, 144.57, 135.0 (2 overlapping signals), 132.0, 131.23, 131.17, 127.2, 127.1, 125.9 (2 overlapping signals), 123.9, 118.0, 105.0; IR ν_{max}/cm^{-1}: 2725 cm^{-1} (NH), 2209 cm^{-1} (CN), 1583 cm^{-1} (NH), 809 cm^{-1} (C-Cl); LRMS (ESI$^+$) m/z: 314 [M+H].

(Z)-2-(2,6-dichlorophenyl)-3-(2-nitrophenyl)acrylonitrile (1s) Prepared as previously described,[2] m.p.: 144-146 °C. ^{1}H NMR (400 MHz, acetone-*d$_6$*) δ 8.34 (dd, *J* = 8.2, 0.8 Hz, 1H), 8.07 – 8.00 (m, 2H), 7.90 (s, 1H), 7.89 – 7.84 (m, 1H), 7.65 (d, *J* = 1.6 Hz, 1H), 7.63 (s, 1H), 7.57 (dd, *J* = 9.2, 6.9 Hz, 1H); ^{13}C NMR (101 MHz, acetone-*d$_6$*) δ 149.5, 148.4, 135.9, 135.5, 132.9 (2C), 132.53 (2C), 132.49, 131.9, 130.2, 129.7, 126.1, 116.0, 110.8; IR ν_{max}/cm^{-1}: 3049 (C=C), 2213 (CN), 1518 (NO_2), 1339 (NO_2), 779 (C-Cl); LRMS (ESI$^+$) m/z: 341 [M+Na].

(Z)-2-(2,6-dichlorophenyl)-3-(3-nitrophenyl)acrylonitrile (1t) Prepared as previously described,[2] m.p.: 167-169 °C. ^{1}H NMR (400 MHz, acetone-*d$_6$*) δ 8.85 (s, 1H), 8.43 (t, *J* = 6.8 Hz, 2H), 7.93 (t, *J* = 8.0 Hz, 1H), 7.70 (s, 1H), 7.66-7.64 (m,

2H), 7.59-7.55 (m, 1H); ^{13}C NMR (101 MHz, acetone-d_6) δ 149.6, 148.9, 135.8, 135.7, 135.4, 133.0 (2C), 132.9, 131.6, 129.8 (2C), 126.5, 124.5, 116.5, 109.2; IR ν_{max}/cm^{-1}: 3082 (C=C), 2214 (CN), 1525 (NO_2), 1347 (NO_2), 779 (C-Cl); LRMS (ESI$^+$) m/z: 341 [M+Na].

(Z)-2-(2,6-dichloro-3-nitrophenyl)-3-(2-nitrophenyl)acrylonitrile (1u) Prepared as previously described,[2] m.p.: 150-152 °C. ^{1}H NMR (400 MHz, $CDCl_3$) δ 8.33 (dd, J = 8.2, 1.0 Hz, 1H), 8.02 (d, J = 7.7 Hz, 1H), 7.91 (d, J = 8.8 Hz, 1H), 7.87 (td, J = 7.6, 0.9 Hz, 1H), 7.76 – 7.71 (m, 2H), 7.64 (d, J = 8.8 Hz, 1H); ^{13}C NMR (101 MHz, $CDCl_3$) δ 149.2, 147.7, 147.1, 139.8, 134.8, 134.3, 131.9, 131.2, 129.2, 129.0, 128.9, 126.9, 125.6, 114.6, 109.5; IR ν_{max}/cm^{-1}: 3066 (C=C), 2215 (CN), 1522 (NO_2), 1521 (NO_2), 1339 (NO_2), 1338 (NO_2), 797 (C-Cl); LRMS (ESI$^+$) m/z: 386 [M+Na-H].

(Z)-2-(2,6-dichloro-3-nitrophenyl)-3-(3-nitrophenyl)acrylonitrile (1v) Prepared as previously described,[2] m.p.: 151-152 °C. ^{1}H NMR (400 MHz, acetone-d_6) δ 8.87 – 8.86 (m, 1H), 8.48 – 8.42 (m, 2H), 8.20 (d, J = 8.8 Hz, 1H), 7.98 – 7.94 (m, 2H), 7.87 (s, 1H); ^{13}C NMR (101 MHz, acetone-d_6) δ 150.2, 149.7, 149.0*, 139.7, 135.8, 135.4, 135.1, 131.8, 130.8, 128.3, 128.0, 126.8, 124.7, 116.0, 108.1. *1 Quaternary carbon not observed in spectra, confirmed by 2D correlations (149.0); IR ν_{max}/cm^{-1}: 3085 (C=C), 2219 (CN), 1523 (NO_2), 1351 (NO_2), 758 (C-Cl); LRMS (ESI$^+$) m/z: 386 [M+Na-H].

(Z)-2-(2,6-dichloro-3-nitrophenyl)-3-(4-nitrophenyl)acrylonitrile (1w) Prepared as previously described,[2] m.p.: 154-156 °C. ^{1}H NMR (400 MHz, acetone-d_6) δ 8.48 (dd, J = 7.0, 1.8 Hz, 2H), 8.26 (d, J = 8.7 Hz, 2H), 8.20 (d, J = 8.8 Hz, 1H), 7.95 (d, J = 8.8 Hz, 1H), 7.86 (s, 1H); ^{13}C NMR (101 MHz, acetone-d_6) δ 150.3, 150.2, 149.0, 139.6, 139.4, 135.3, 131.3 (2C), 130.7, 128.3, 128.0, 125.1 (2C), 115.9, 109.1; IR ν_{max}/cm^{-1}: 3082 (C=C), 2223 (CN), 1514 (NO_2), 1339 (NO_2), 747 (C-Cl); LRMS (ESI$^+$) m/z: 364 [M+H].

(Z)-2-(3-amino-2,6-dichlorophenyl)-3-(2-aminophenyl)acrylonitrile (1x) Prepared as previously described,[2] m.p.: 226-228 °C. ^{1}H NMR (400 MHz, acetone-d_6) δ 7.76 (s, 1H), 7.71 (d, J = 8.0 Hz, 1H), 7.61 (d, J = 8.4 Hz, 1H), 7.54 (t, J = 7.6 Hz, 1H), 7.27 – 7.21 (m, 2H), 6.98 (d, J = 8.8 Hz, 1H), 5.60 (bs, NH_2), 5.25 (bs, NH_2); ^{13}C NMR (101 MHz, acetone-d_6) δ 156.3, 149.1, 145.4, 138.3, 135.7, 130.2, 129.3, 128.5, 126.6, 124.4, 122.7, 122.3, 122.2, 119.8, 116.9; IR ν_{max}/cm^{-1}: 3149 (C=C), 1635 (NH_2), 1615 (NH_2), 788 (C-Cl); LRMS (ESI$^+$) m/z: 305 [M+H].

(Z)-2-(3-amino-2,6-dichlorophenyl)-3-(3-aminophenyl)acrylonitrile (1y) Prepared as previously described,[2] m.p.: 49-52 °C. ^{1}H NMR (400 MHz, acetone-d_6) δ 7.26 – 7.17 (m, 4H), 7.15 (s, 1H), 6.98 (d, J = 8.8 Hz, 1H), 6.85 (ddd, J = 7.7, 2.2, 1.4 Hz, 1H), 5.34 (bs, NH_2), 4.97 (bs, NH_2); ^{13}C NMR (101 MHz, acetone-d_6) δ 151.1, 150.0, 145.5, 134.9, 134.0, 130.5, 129.4, 124.6, 123.3, 121.6, 120.5, 118.7, 118.1, 117.4, 114.9; IR ν_{max}/cm^{-1}: 3221 (C=C), 2209 (CN), 1615 (NH_2), 1581 (NH_2), 783 (C-Cl); LRMS (ESI$^+$) m/z: 305 [M+H].

(Z)-2-(3,4-Dichlorophenyl)-3-(4-(2-hydroxy-3-(phenylamino)-propoxy)phenyl)acrylonitrile (8a) Prepared according to general procedure 2 as previously described,[4] m.p.: 130-133 °C. ^{1}H NMR (400 MHz, acetone-d_6) δ 8.02 (d, J = 8.8 Hz, 2H), 7.98 (s, 1H), 7.94 (d, J = 1.6 Hz, 1H), 7.713 – 7.706 (m, 2H), 7.14 (d, J = 8.8 Hz, 2H), 7.10 (dd, J = 8.5, 7.4 Hz, 2H), 6.70 (d, J = 7.7 Hz, 2H), 6.60 (t, J = 7.6 Hz, 1H), 4.96 (s, 1H), 4.43 (d, J = 4.6 Hz, 1H), 4.27 – 4.21 (m, 2H), 4.21 – 4.15 (m, 1H), 3.50 – 3.40 (m, 1H), 3.28 (dd, J = 12.9, 5.7 Hz, 1H); ^{13}C NMR (101 MHz, acetone-d_6) δ 162.4, 149.9, 144.7, 136.5, 133.5, 132.8, 132.5 (2C), 132.0, 129.8 (2C), 128.2, 127.2, 126.5, 118.5, 117.4, 116.0 (2C), 113.5 (2C), 106.3, 71.7, 69.1, 47.3; IR ν_{max}/cm^{-1}: 3497 (NH), 3390 (br, OH), 3050 (C=C), 2210 (CN), 1598 (NH), 1253 (C-O-C), 815 cm^{-1} (C-Cl). LRMS: (ESI$^+$) m/z: 439 ($C_{24}H_{21}Cl_2N_2O_2$) [M+H].

(Z)-2-(3,4-Dichlorophenyl)-3-(4-(2-hydroxy-3-((4-chlorophenyl)-amino)propoxy)-phenyl)acrylonitrile (8b) Prepared according to general procedure 2 as previously described,[4] m.p.: 110-113 °C. ^{1}H NMR (400 MHz, acetone-d_6) δ 8.01 (d, J = 8.8 Hz, 2H), 7.97 (s, 1H), 7.94 (d, J = 1.6 Hz, 1H), 7.71 – 7.70 (m, 2H), 7.14 (d, J = 8.8 Hz, 2H), 7.09 (d, J = 8.8 Hz, 2H), 6.72 (d, J = 8.8 Hz, 2H), 5.19 (t, J = 5.6 Hz, NH), 4.49 (d, J = 4.4 Hz, 1H, OH), 4.23 – 4.17 (m, 3H), 3.45 (ddd, J = 13.1, 6.6, 4.6 Hz, 1H), 3.31 – 3.25 (m, 1H); ^{13}C NMR (101 MHz, acetone-d_6) δ 162.3, 148.8, 144.7, 136.5, 133.5, 132.8, 132.5 (2C), 132.0, 129.6 (2C), 128.2, 127.2, 126.5, 121.2, 118.5, 116.0 (2C), 114.7 (2C), 106.4, 71.5, 69.0, 47.4; IR ν_{max}/cm^{-1}: 3385 (br, OH), 2926 (C=C), 2210 (CN), 1586 (NH), 1257 (C-O-C), 828 (C-Cl); LRMS: (ESI$^+$) m/z: 473 ($C_{24}H_{20}Cl_3N_2O_2$) [M+H].

(Z)-2-(3,4-Dichlorophenyl)-3-(4-(2-hydroxy-3-((4-bromophenyl)amino)propoxy)-phenyl)acrylonitrile (8c) Prepared according to general procedure 2 as previously described,[4] m.p.: 121-124 °C. ^{1}H NMR (400 MHz, acetone-d_6) δ 8.00 (d, J = 7.6 Hz, 2H), 7.95 (d, J = 2.8 Hz, 1H), 7.92 (s, 1H), 7.69 – 7.68 (m, 2H), 7.21 (d, J = 8.8 Hz, 2H), 7.13 – 7.11 (m, 2H), 6.67 (d, J = 8.8 Hz, 2H), 5.21 (bs, NH), 4.49 (d, J = 4.5 Hz, 1H), 4.25 – 4.15 (m, 3H), 3.47 – 3.41 (m, 1H), 3.31 – 3.24 (m, 1H); ^{13}C NMR (101 MHz, acetone-d_6) δ 162.3, 149.1, 144.6, 136.4, 133.5, 132.7, 132.44, 132.40, 131.9, 128.1, 127.2, 126.5, 118.4, 115.9, 115.2, 108.1, 106.3, 71.5, 68.9, 47.2; IR ν_{max}/cm^{-1}: 3380 (br, OH), 3264 (C=C), 2878 (O-CH_3), 2210 (CN), 1594 (NH), 1246 (C-O-C), 812 (C-Cl); LRMS: (ESI$^+$) m/z: 517 ($C_{24}H_{20}BrCl_2N_2O_2$) [M+H].

(*Z)-2-(3,4-Dichlorophenyl)-3-(4-(2-hydroxy-3-((4-methoxyphenyl)amino)propoxy)-phenyl)acrylonitrile (8d)* Prepared according to general procedure 2 as previously described,[4] m.p.: 130-133 °C. ^{1}H NMR (400 MHz, acetone-d_6) δ 8.01 (d, J = 8.8 Hz, 2H), 7.97 (s, 1H), 7.94 (d, J = 1.5 Hz, 1H), 7.70 – 7.70 (m, 2H), 7.13 (d, J = 8.8 Hz, 2H), 6.76 – 6.73 (m, 2H), 6.68 – 6.66 (m, 2H), 4.59 (bs, NH), 4.41 (d, J = 4.5 Hz, OH), 4.25 – 4.14 (m, 3H), 3.68 (s, 3H), 3.39 (dd, J = 12.6, 3.6 Hz,

1H), 3.22 (dd, *J* = 12.6, 6.0 Hz, 1H); ^{13}C NMR (101 MHz, acetone-*d*$_6$) δ 162.4, 152.8, 144.7, 144.1, 136.5, 133.5, 132.7, 132.5 (2C), 132.0, 128.2, 127.2, 126.5, 118.5, 116.0 (2C), 115.5 (2C), 114.8 (2C), 106.3, 71.8, 69.2, 55.8, 48.3; IR ν_{max}/cm^{-1}: 3260 (br, OH), 3050 (C=C), 2829 (O-CH$_3$), 2210 (CN), 1596 (NH), 1272 (C-O-C), 820 (C-Cl); LRMS: (ESI+) m/z: 469 ($C_{25}H_{23}Cl_2N_2O_3$) [M+H]; (ESI$^-$) m/z: 513 ($C_{26}H_{24}Cl_2N_2O_5$) [M+FA-H].

(Z)-3-(4-(3-(Cyclohexylamino)-2-hydroxypropoxy)phenyl)-2-(3,4-dichlorophenyl)-acrylonitrile (8e) Prepared according to general procedure 2 as previously described,[4] m.p.: 112-114 °C. ^{1}H NMR (600 MHz, acetone-*d*$_6$) δ 8.01 (d, *J* = 8.8 Hz, 2H), 7.97 (s, 1H), 7.94 (d, *J* = 2.0 Hz, 1H), 7.73 – 7.68 (m, 2H), 7.14 – 7.10 (m, 2H), 4.16 (dd, *J* = 9.7, 4.5 Hz, 1H), 4.09 (dd, *J* = 9.7, 6.0 Hz, 1H), 4.03 (dd, *J* = 12.0, 5.4 Hz, 1H), 2.92 (dd, *J* = 11.9, 4.6 Hz, 1H), 2.77 (dd, *J* = 11.9, 7.1 Hz, 1H), 2.48 (ddd, *J* = 10.1, 7.0, 3.8 Hz, 1H), 1.91 – 1.90 (m, 2H), 1.73 – 1.70 (m, 2H), 1.60 – 1.57 (m, 1H), 1.28 (tt, *J* = 15.7, 3.3 Hz, 2H), 1.21 – 1.16 (m, 1H), 1.15 – 1.07 (m, 2H); ^{13}C NMR (151 MHz, acetone-*d*$_6$) δ 162.5, 144.7, 136.5, 133.5, 132.7, 132.5 (2C), 132.0, 128.2, 127.1, 126.5, 118.5, 116.0 (2C), 106.2, 72.0 (2C), 69.4, 57.5, 50.0 (2C), 34.1 (d, J = 21.1 Hz), 26.9, 25.6; IR ν_{max}/cm^{-1}: 3305 (NH), 3119 (br, OH), 2922 (C=C), 2853 (C-O-C), 2209 (CN), 1597 (NH), 1256 (C-O-C), 823 cm^{-1} (C-Cl).; LRMS: (ESI$^+$) m/z: 445 ($C_{24}H_{27}Cl_2N_2O_2$) [M+H].

(Z)-3-(4-(3-(4-Cyclohexylpiperazin-1-yl)-2-hydroxypropoxy)-phenyl)-2-(3,4-dichlorophenyl)-acrylonitrile (8f) Prepared according to general procedure 3 as previously described,[4] m.p.: 137-139 °C. ^{1}H NMR (400 MHz, DMSO-*d*$_6$) δ 8.10 (s, 1H), 8.00 (d, *J* = 2.1 Hz, 1H), 7.96 (d, *J* = 8.8 Hz, 2H), 7.76 (d, *J* = 8.5 Hz, 1H), 7.69 (d, *J* = 2.1 Hz, 1H), 7.13 (d, *J* = 8.8 Hz, 2H), 4.88 (d, *J* = 4.1 Hz, 1H), 4.10 – 4.06 (m, 1H), 3.95 (d, *J* = 6.2 Hz, 2H), 2.46 – 2.31 (m, 10H), 2.16 (s, 1H), 1.74 – 1.70 (m, 4H), 1.55 (d, *J* = 11.7 Hz, 1H), 1.23 – 1.04 (m, 5H); ^{13}C NMR (101 MHz, DMSO-*d*$_6$) δ 161.2, 144.3, 135.0, 132.0 (2 overlapping signals), 131.5 (2C), 131.2, 131.1, 127.0, 125.8, 117.9 (2C), 115.1, 104.3, 71.5, 66.4, 62.5, 61.1, 54.1 (2C), 48.5 (2C), 28.4, 25.9 (2C), 25.3 (2C); IR ν_{max}/cm^{-1}: 3498 (OH), 2812 (N-CH$_2$), 2212 (CN), 1269 (C-O-C), 813 (C-Cl); LRMS: (ESI$^+$) m/z: 514 ($C_{28}H_{34}Cl_2N_3O_2$) [M+H].

(Z)-2-(3,4-Dichlorophenyl)-3-(4-(2-hydroxy-3-(piperidin-1-yl)propoxy)phenyl)acrylonitrile (8g) Prepared according to general procedure 3 as previously described,[4] m.p.: 111-115 °C. ^{1}H NMR (400 MHz, acetone-*d*$_6$) δ 8.01 (d, *J* = 8.8 Hz, 2H), 7.97 (s, 1H), 7.94 (d, *J* = 1.6 Hz, 1H), 7.73 – 7.68 (m, 2H), 7.13 (d, *J* = 8.8 Hz, 2H), 4.18 – 4.15 (m, 1H), 4.12 – 4.05 (m, 2H), 2.53 – 2.44 (m, 6H), 1.57 (dt, *J* = 10.7, 5.4 Hz, 4H), 1.46 – 1.41 (m, 2H); ^{13}C NMR (101 MHz, acetone-*d*$_6$) δ 162.6, 144.8, 136.5, 133.5, 132.7, 132.5 (2C), 132.0, 128.2, 127.1, 126.5, 118.5, 116.0 (2C), 106.2, 72.3, 67.2, 62.4, 55.8 (2C), 26.89 (2C), 25.0; IR ν_{max}/cm^{-1}: 3300 (b, OH), 2853 (N-CH$_2$), 2214 (CN), 1264 (C-O-C), 816 (C-Cl); LRMS: (ESI$^+$) m/z: 431 ($C_{23}H_{25}Cl_2N_2O_2$) [M+H].

(Z)-2-(3,4-Dichlorophenyl)-3-(4-(2-hydroxy-3-(D-11-piperidin-1-yl)propoxy)phenyl)-acrylonitrile (8j) Prepared according to general procedure 3 as previously described,[4] m.p.: 103-105 °C. ^{1}H NMR (600 MHz, DMSO-*d*$_6$) δ 8.10 (s, 1H), 8.01 (d, *J* = 1.7 Hz, 1H), 7.96 (d, *J* = 8.7 Hz, 2H), 7.77 (d, *J* = 8.5 Hz, 1H), 7.69 (dd, *J* = 8.5, 1.9 Hz, 1H), 7.13 (d, *J* = 8.7 Hz, 2H), 4.85 (d, *J* = 3.0 Hz, 1H), 4.08 (t, *J* = 6.4 Hz, 1H), 3.97 – 3.95 (m, 2H), 2.39 (dd, *J* = 12.6, 5.8 Hz, 1H), 2.32 (dd, *J* = 12.6, 5.8 Hz, 1H); ^{13}C NMR (151 MHz, DMSO-*d*$_6$) δ 161.2, 144.3, 135.0, 132.0, 131.5 (2C), 131.2, 131.1, 127.0, 125.78, 125.75, 117.9, 115.1 (2C), 104.3, 71.5, 66.4, 61.6; IR ν_{max}/cm^{-1}: 3300 (OH), 2931 (N-CH$_2$), 2211 (CN), 1182 (C-O-C), 815 (C-Cl); LRMS (ESI$^+$) m/z: 441 ($C_{23}H_{15}D_{10}{}^{35}Cl_2N_2O_2$) [M+H].

(Z)-3-(4-(3-(4-Acetylpiperazin-1-yl)-2-hydroxypropoxy)phenyl)-2-(3,4-dichlorophenyl)-acrylonitrile (8k) Prepared according to general procedure 3 as previously described,[4] m.p.: 88-91 °C. ^{1}H NMR (400 MHz, DMSO-*d*$_6$) δ 8.10 (s, 1H), 8.01 (d, *J* = 2.2 Hz, 1H), 7.96 (d, *J* = 8.9 Hz, 2H), 7.77 (d, *J* = 8.5 Hz, 1H), 7.69 (dd, *J* = 8.5, 2.2 Hz, 1H), 7.14 (d, *J* = 8.9 Hz, 2H), 4.97 (d, *J* = 4.3 Hz, 1H), 4.10 – 4.07 (m, 1H), 4.00 – 3.96 (m, 2H), 3.42 – 3.39 (m, 4H), 2.48 – 2.37 (m, 10H), 1.97 (s, 3H); ^{13}C NMR (101 MHz, DMSO-*d*$_6$) δ 168.1, 161.2, 144.4, 135.0, 132.1, 131.5 (2C), 131.3, 131.2, 127.0, 125.84, 125.81, 117.9, 115.2 (2C), 104.4, 71.3, 66.5, 60.8, 53.7* (2C), 53.2* (2C), 45.8* (2C), 40.9* (2C), 21.2. *Isomerization confirmed by 2D correlations; IR ν_{max}/cm^{-1}: 3367 (br, OH), 2819 (N-CH$_2$), 2212 (CN), 1182 (C-O-C), 815 (C-Cl); LRMS (ESI$^+$) m/z: 474 ($C_{24}H_{26}Cl_2N_3O_3$) [M+H].

(Z)-2-(3,4-Dichlorophenyl)-3-(4-(2-hydroxy-3-morpholinopropoxy)phenyl)acrylonitrile (8l) Prepared according to general procedure 3 as previously described,[4] m.p.: 103-105 °C. ^{1}H NMR (400 MHz, acetone-*d*$_6$) δ 8.01 (d, *J* = 8.9 Hz, 2H), 7.97 (s, 1H), 7.94 (d, *J* = 1.6 Hz, 1H), 7.71 – 7.70 (m, 2H), 7.13 (d, *J* = 8.9 Hz, 2H), 4.20 (dd, *J* = 9.1, 3.4 Hz, 1H), 4.17 – 4.13 (m, 1H), 4.09 (dd, *J* = 9.0, 5.4 Hz, 1H), 3.64 (s, 4H), 2.61 – 2.51 (m, 6H); ^{13}C NMR (101 MHz, acetone-d$_6$) δ 162.5, 144.7, 136.5, 133.5, 132.7, 132.5 (2C), 132.0, 128.2, 127.1, 126.5, 118.5, 116.0 (2C), 106.3, 72.1, 67.4 (2C), 67.3, 62.3, 55.1 (2C); IR ν_{max}/cm^{-1}: 3360 (br, OH), 2861 (N-CH$_2$), 2214 (CN), 1270 (C-O-C), 814 (C-Cl); LRMS: (ESI$^+$) m/z: 433 ($C_{22}H_{23}Cl_2N_2O_3$) [M+H].

(Z)-2-(3,4-Dichlorophenyl)-3-(2-(2-hydroxy-3-(4-phenyl-piperazin-1-yl)propoxy)phenyl)-acrylonitrile (8m) Prepared according to general procedure 3 as previously described,[4] m.p.: 128-130 °C. ^{1}H NMR (400 MHz, acetone-*d*$_6$) δ 8.27 (s, 1H), 8.11 (dd, *J* = 7.8, 1.2 Hz, 1H), 7.92 (t, *J* = 1.2 Hz, 1H), 7.72 (d, *J* = 1.2 Hz, 2H), 7.53 – 7.49 (m, 1H), 7.23 – 7.19 (m, 3H), 7.12 (t, *J* = 7.6 Hz, 1H), 6.93 (d, *J* = 7.9 Hz, 2H), 6.77 (t, *J* = 7.3 Hz, 1H), 4.25 (d, *J* = 8.9 Hz, 1H), 4.22 – 4.14 (m, 2H), 3.17 (t, *J* = 5.0 Hz, 4H), 2.72 – 2.61 (m, 6H); ^{13}C NMR (101 MHz, acetone-*d*$_6$) δ 168.5, 162.2, 150.2, 146.0, 143.33,

143.29, 142.9, 141.8, 139.4 (2C), 138.8, 138.2, 136.4, 133.5, 131.4, 129.6, 127.7, 126.2 (2C), 123.5, 119.5, 82.6, 77.3, 71.6, 64.3 (2C), 59.5 (2C); IR ν_{max}/cm^{-1}: 3433 (OH), 2814 (N-CH_2), 2214 (CN), 1228 (C-O-C), 830 (C-Cl); LRMS: (ESI^+) m/z: 508 ($C_{28}H_{28}Cl_2N_3O_2$) [M+H].

(Z)-2-(3,4-Dichlorophenyl)-3-(3-(2-hydroxy-3-(4-phenyl-piperazin-1-yl)propoxy)phenyl)-acrylonitrile (8n) Prepared according to general procedure 3 as previously described,[4] m.p.: 132-134 °C. ^{1}H NMR (400 MHz, DMSO-d_6) δ 8.17 (s, 1H), 8.05 (s, 1H), 7.78 (d, *J* = 8.2 Hz, 1H), 7.71 (d, *J* = 8.1 Hz, 1H), 7.54 (d, *J* = 12.3 Hz, 2H), 7.48 (d, *J* = 7.7 Hz, 1H), 7.16 (dd, *J* = 16.9, 7.5 Hz, 3H), 6.90 (d, *J* = 7.7 Hz, 2H), 6.77 (d, *J* = 6.2 Hz, 1H), 4.99 (s, 1H), 4.08 (d, *J* = 8.3 Hz, 2H), 3.97 (s, 1H), 3.12 (s, 4H), 2.62 (s, 5H); ^{13}C NMR (101 MHz, DMSO-d_6) δ 158.9, 144.8, 134.6, 134.4, 132.1, 131.8, 131.3, 130.3, 130.2, 128.9, 127.4, 126.2, 121.8, 118.8, 117.8, 117.4, 115.4, 114.0, 108.1, 71.2, 69.2, 53.5, 49.6, 48.2; IR ν_{max}/cm^{-1}: 3190 (O-H), 2978 (C=C), 2216 (CN), 1274 (C-O-C), 819 (C-Cl); LRMS: (ESI^+) m/z: 508 ($C_{28}H_{28}Cl_2N_3O_2$) [M+H].

(Z)-2-(3,4-Dichlorophenyl)-3-(4-(2-hydroxy-3-(4-phenyl-piperazin-1-yl)propoxy)-phenyl)acrylonitrile (8o) Prepared according to general procedure 3 as previously described,[4] m.p.: 175-178 °C. ^{1}H NMR (600 MHz, DMSO-d_6) δ 8.10 (s, 1H), 8.01 (d, *J* = 2.2 Hz, 1H), 7.97 (d, *J* = 8.9 Hz, 2H), 7.77 (d, *J* = 8.5 Hz, 1H), 7.69 (dd, *J* = 8.5, 2.3 Hz, 1H), 7.20 (dd, *J* = 8.5, 7.4 Hz, 2H), 7.15 (d, *J* = 8.9 Hz, 2H), 6.92 (d, *J* = 8.0 Hz, 2H), 6.76 (t, *J* = 7.2 Hz, 1H), 4.98 (d, *J* = 4.7 Hz, 1H), 4.12 (dd, *J* = 9.3, 2.9 Hz, 1H), 4.03 – 4.00 (m, 2H), 3.12 (t, *J* = 4.9 Hz, 4H), 2.63 (dt, *J* = 10.0, 4.8 Hz, 2H), 2.63 – 2.58 (m, 2H), 2.54 (s, 1H), 2.44 (dd, *J* = 12.7, 6.1 Hz, 1H); ^{13}C NMR (151 MHz, DMSO-d_6) δ 161.2, 151.1, 144.3, 134.9, 132.0, 131.5 (2C), 131.2, 131.1, 128.89 (2C), 128.86, 127.0, 125.80, 125.78, 118.7, 117.9, 115.31 (2C), 115.27, 115.1 (2C), 104.4, 71.4, 66.5, 60.9, 53.5, 48.3; IR ν_{max}/cm^{-1}: 3392 (br, OH), 2825 (N-CH_2), 2210 (CN), 1245 (C-O-C), 818 (C-Cl); LRMS: (ESI^+) m/z: 508 ($C_{28}H_{28}Cl_2N_3O_2$) [M+H].

Chiral HPLC of Compound 8g

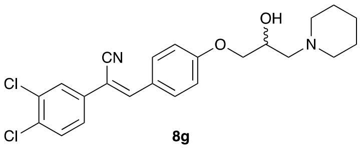

8g

Instrument:	LCMS	Location:	78
Injection date:	10/18/2018 8:17:18 AM	Injection:	1 of 1
Acq. method:	LCMS ISOCRATIC 20% _CHIRALB-1.5-12MINS.M	Injection volume:	5.000
Analysis method:	LCMS ISOCRATIC 20%_CHIRALB-1.5-12MINS.M	Acq. operator:	SYSTEM
Last changed:	10/18/2018 8:03:55 AM		

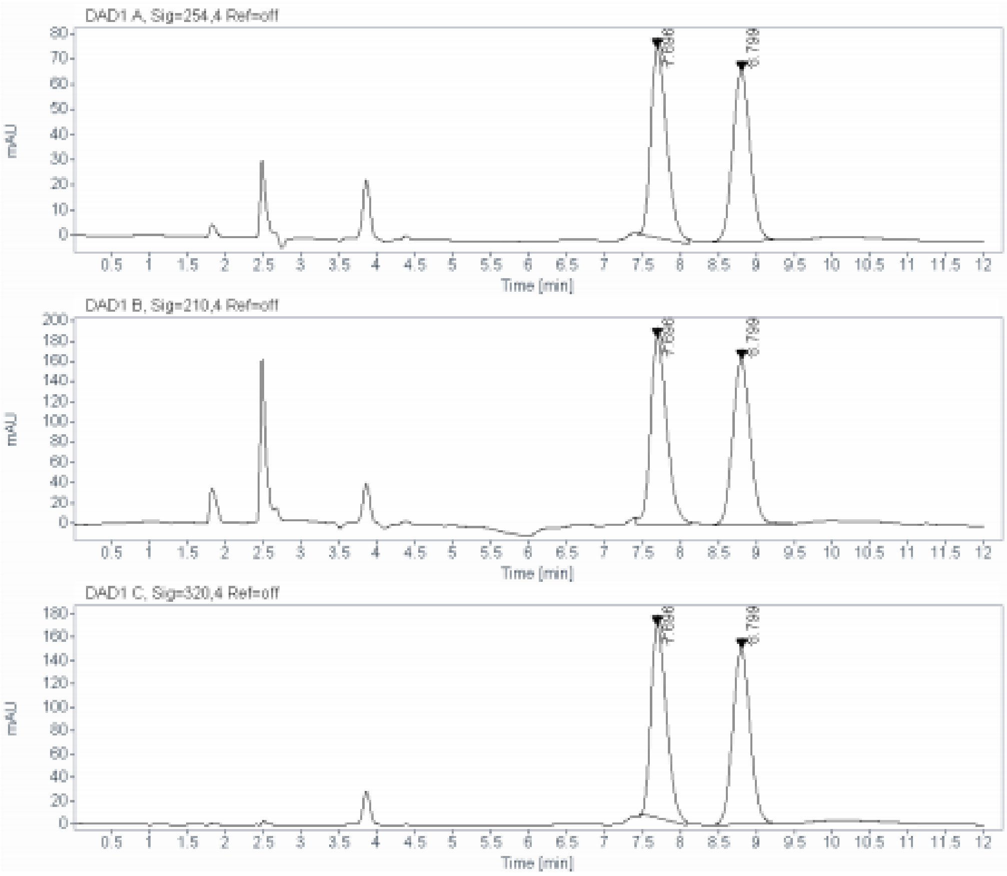

Chiral HPLC of Compound 8h

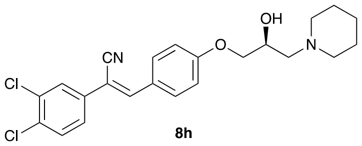

8h

**Instrument:**	LCMS	**Location:**	80
**Injection date:**	10/18/2018 8:31:02 AM	**Injection:**	1 of 1
**Acq. method:**	LCMS ISOCRATIC 20% _CHIRALB-1.5-12MINS.M	**Injection volume:**	5.000
**Analysis method:**	LCMS ISOCRATIC 20%_CHIRALB-1.5-12MINS.M	**Acq. operator:**	SYSTEM
**Last changed:**	10/18/2018 8:03:55 AM		

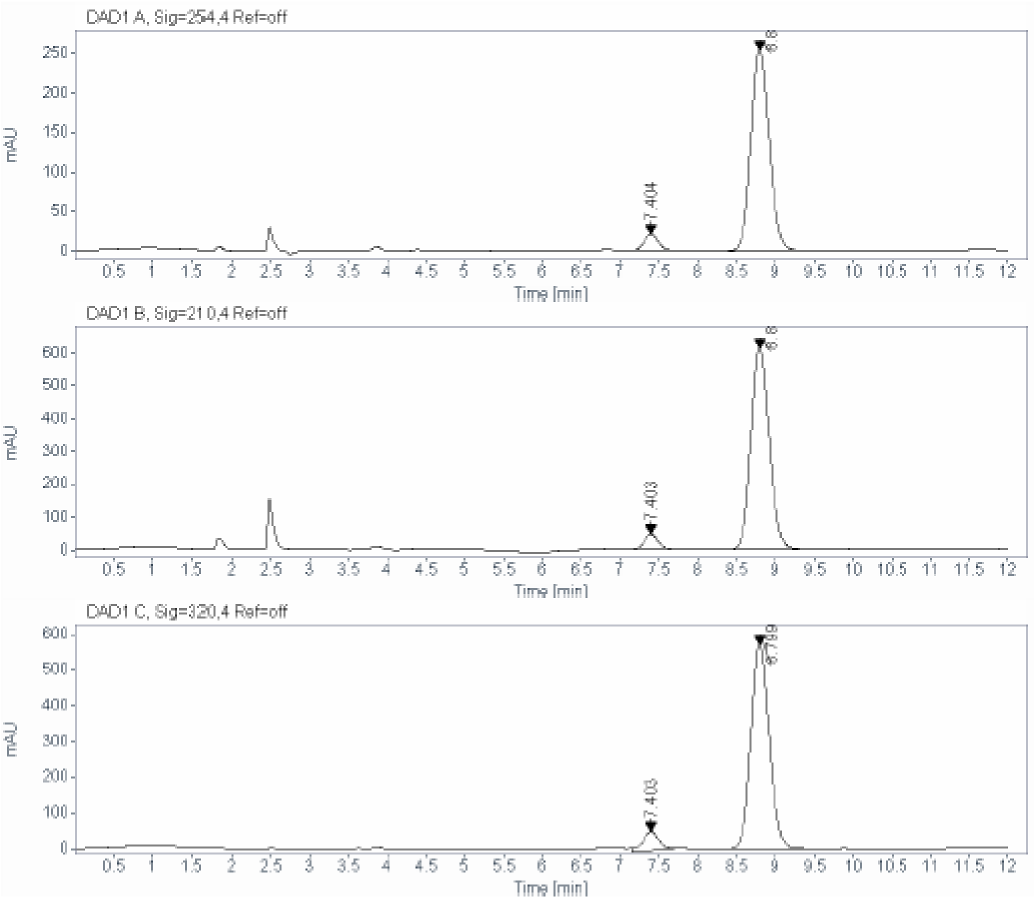

Chiral HPLC of Compound 8i

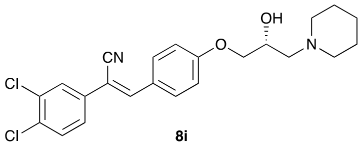

8i

Instrument:	LCMS	Location:	79
Injection date:	10/18/2018 8:44:54 AM	Injection:	1 of 1
Acq. method:	LCMS ISOCRATIC 20% _CHIRALB-1.5-12MINS.M	Injection volume:	5.000
Analysis method:	LCMS ISOCRATIC 20%_CHIRALB-1.5-12MINS.M	Acq. operator:	SYSTEM
Last changed:	10/18/2018 8:03:55 AM		

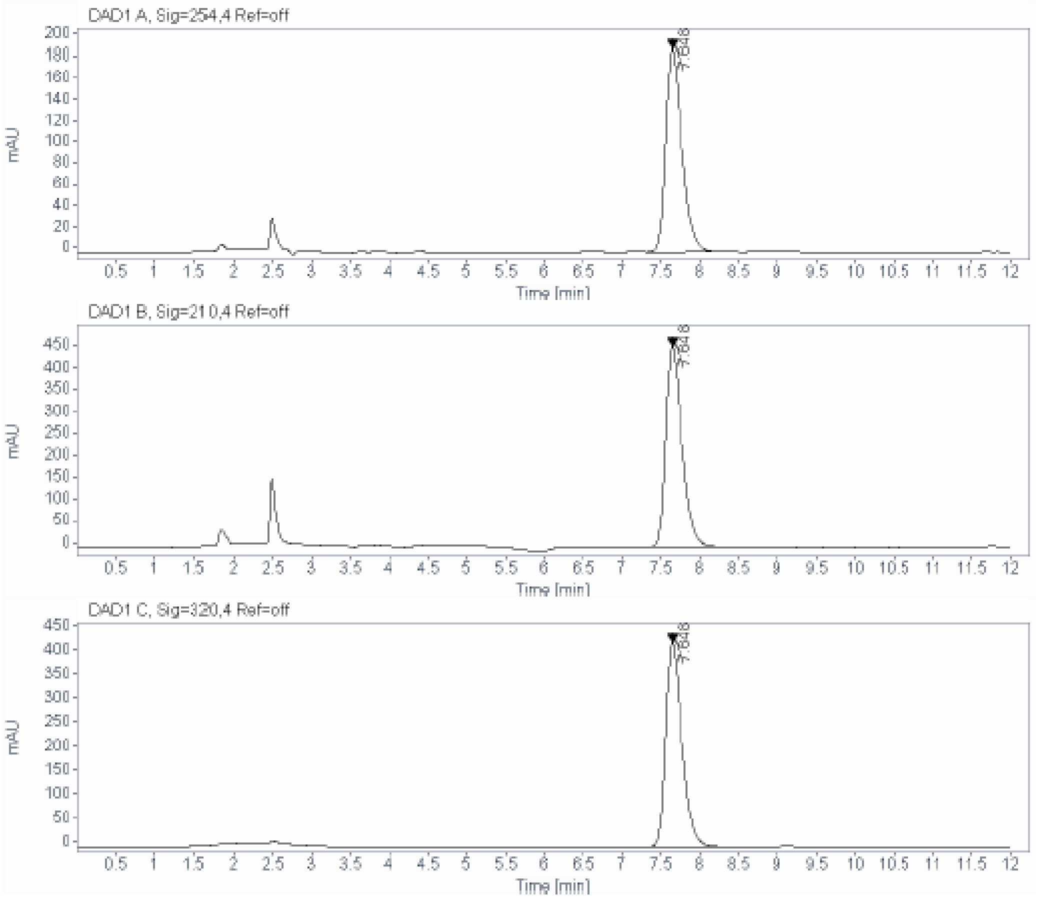

^{1}H and ^{13}C NMR spectra

(*Z*)-4-(2-cyano-2-(3,4-dichlorophenyl)vinyl)-*N*-methylbenzamide (**1a**)

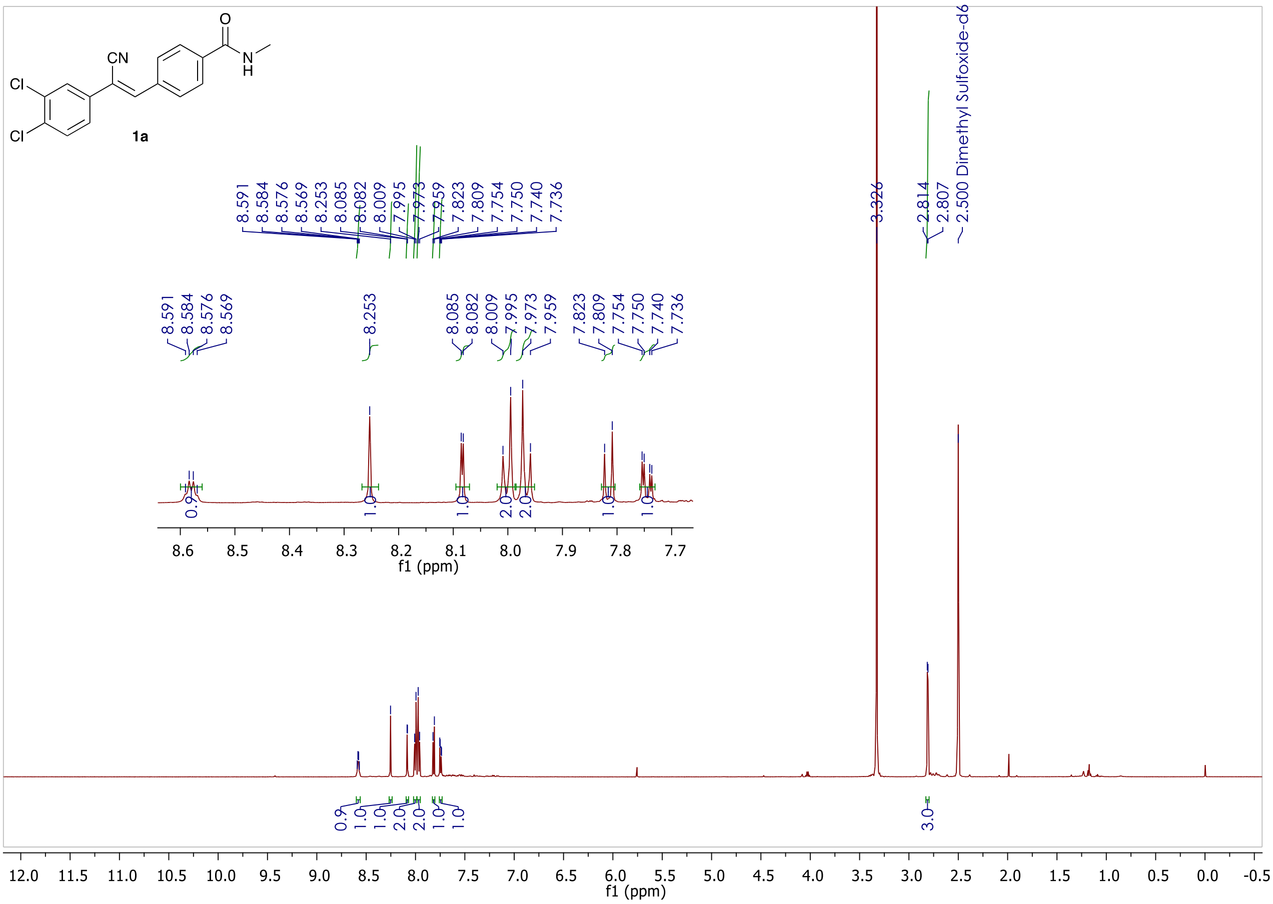

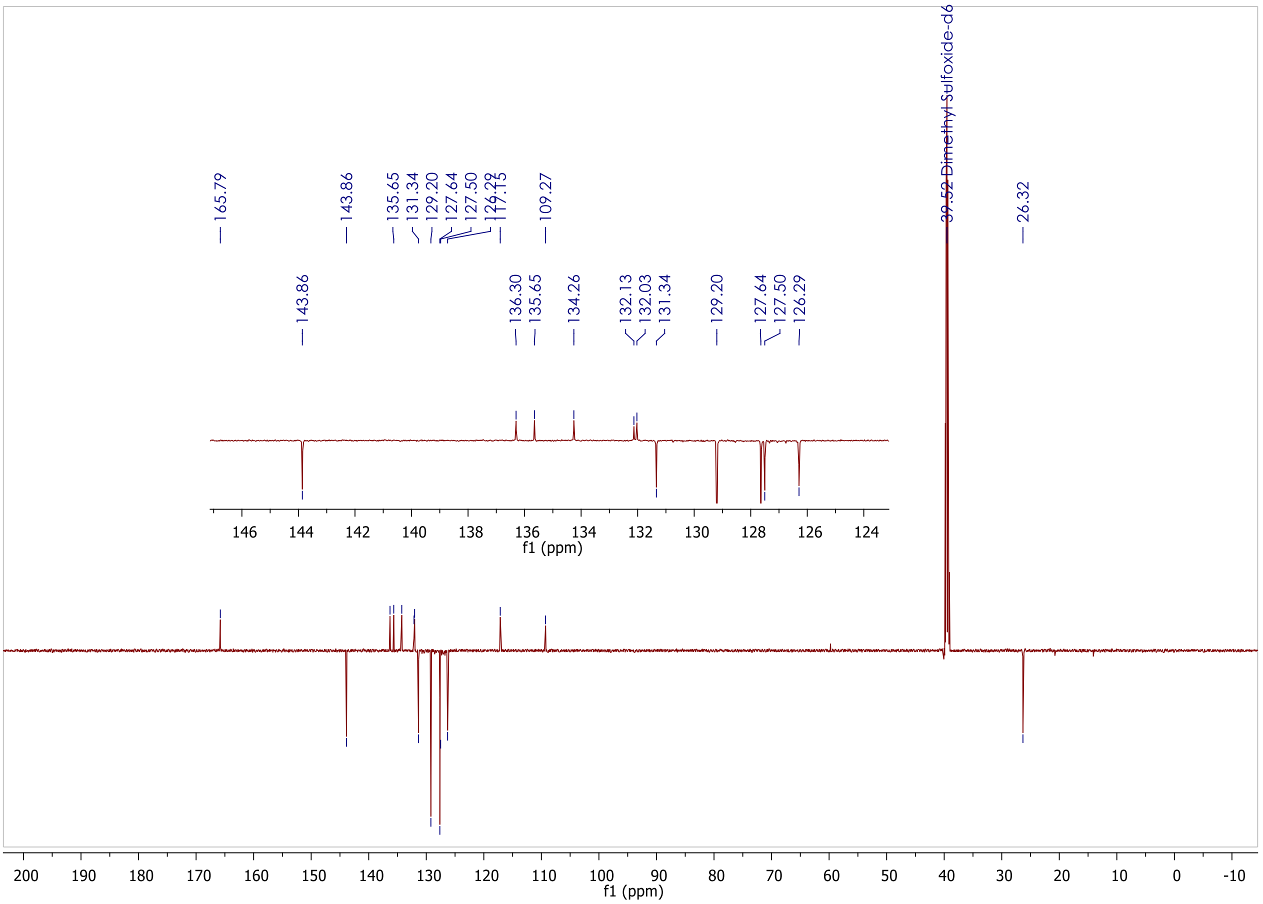

(*Z*)-*N*-(4-(2-cyano-2-(3,4-dichlorophenyl)vinyl)phenyl)acetamide (**1b**)

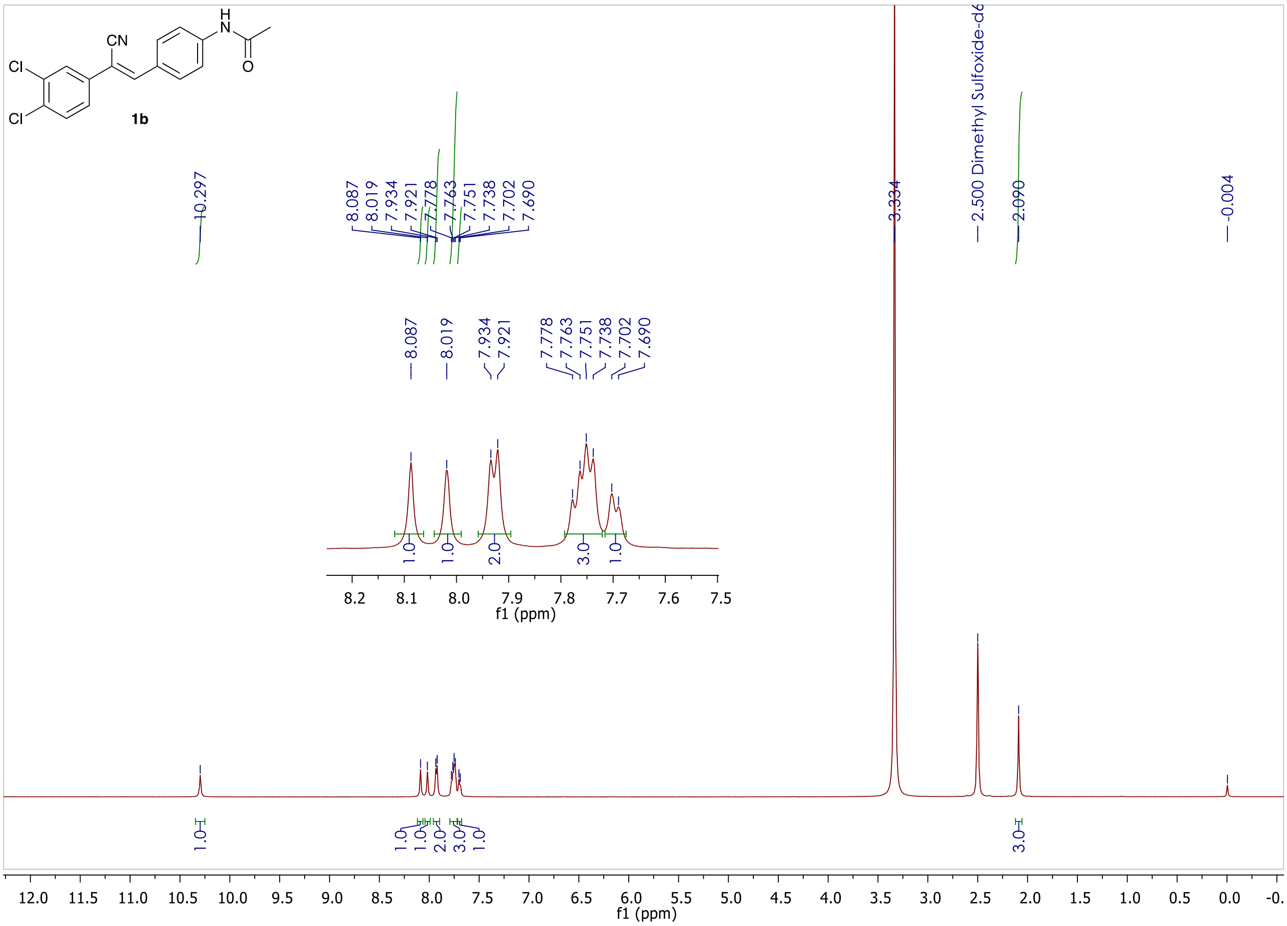

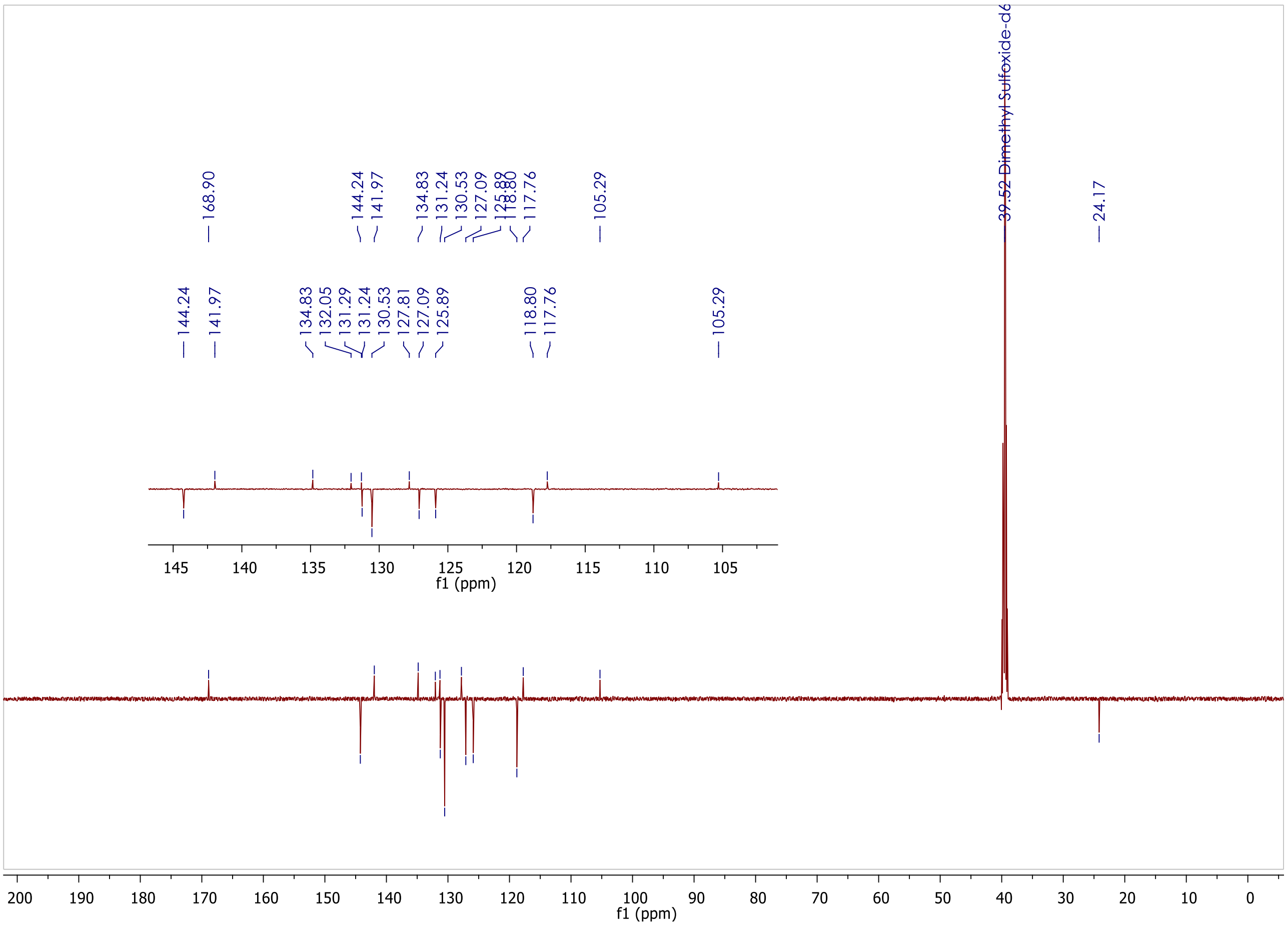

(*Z*)-2-(3,4-dichlorophenyl)-3-(2-nitrophenyl)acrylonitrile (**1c**)

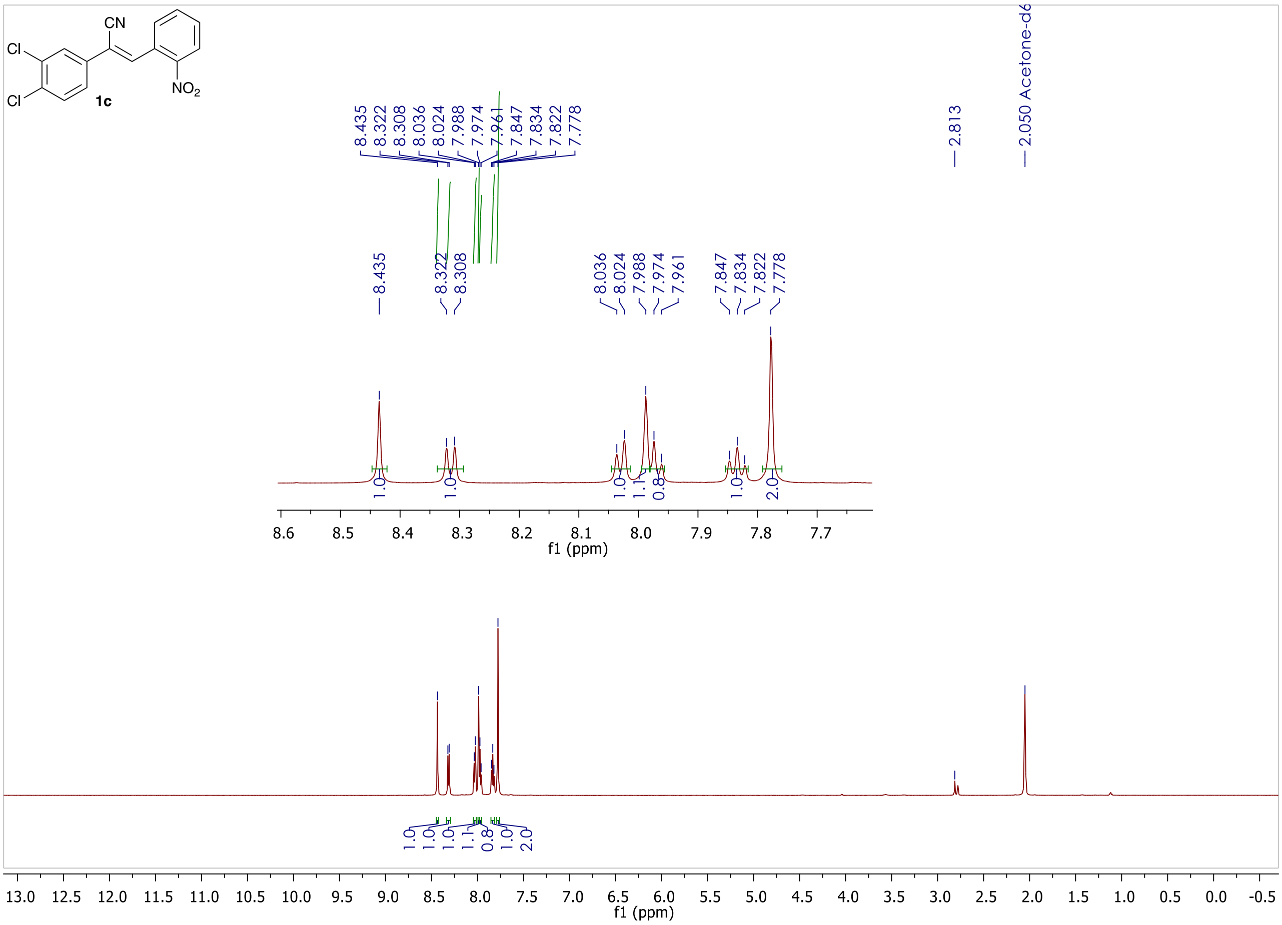

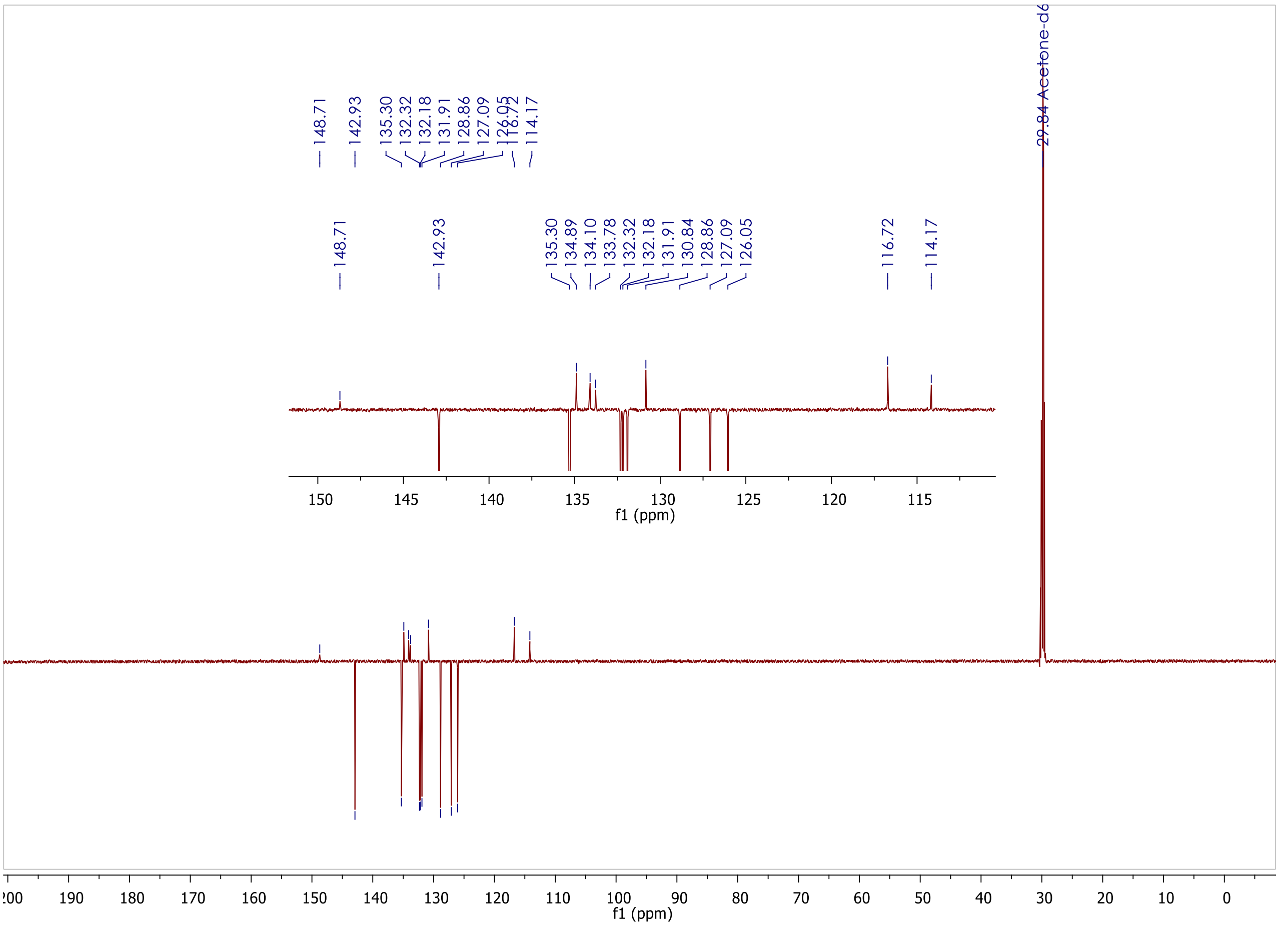

(*Z*)-2-(3,4-dichlorophenyl)-3-(3-nitrophenyl)acrylonitrile (**1d**)

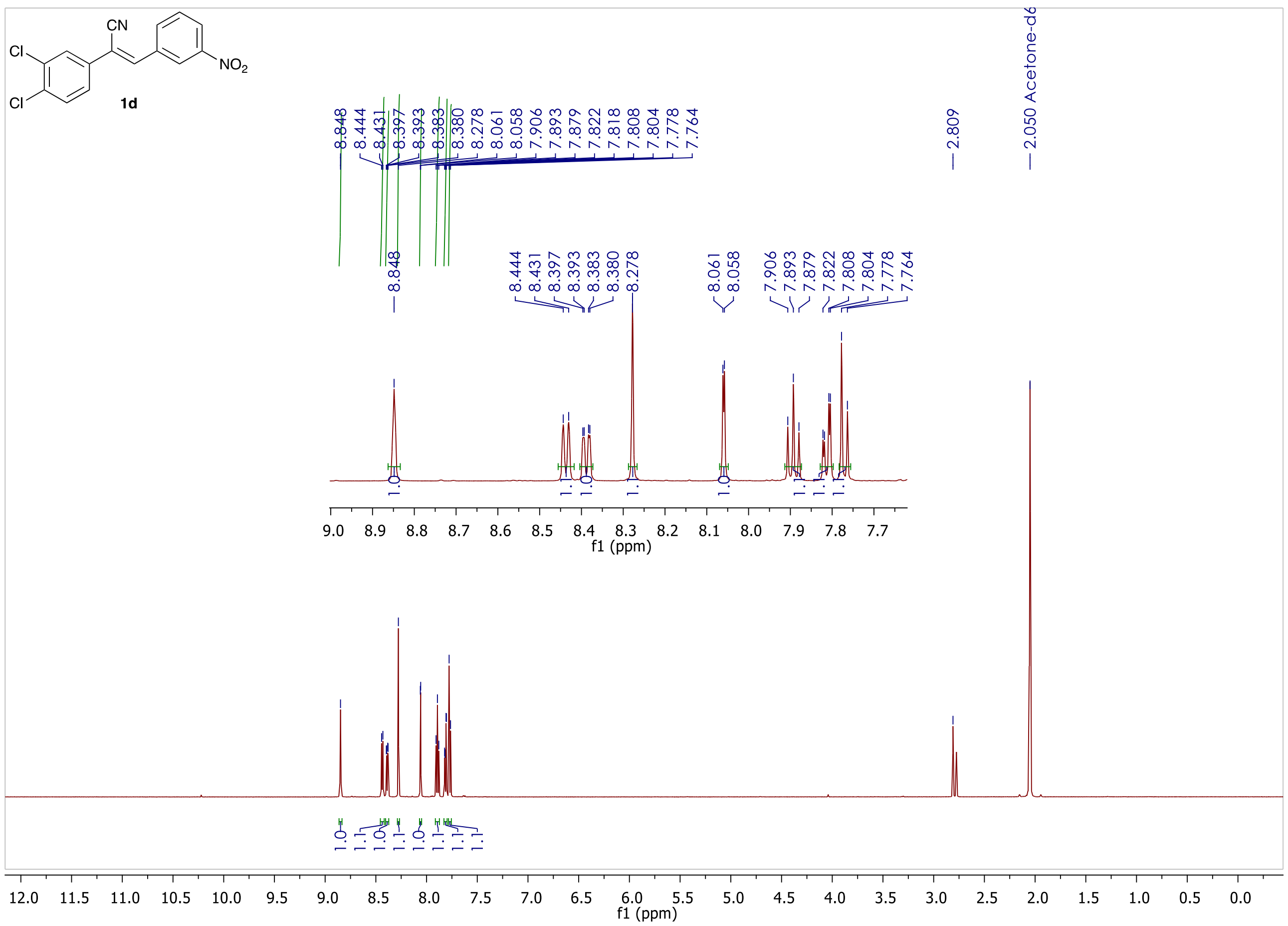

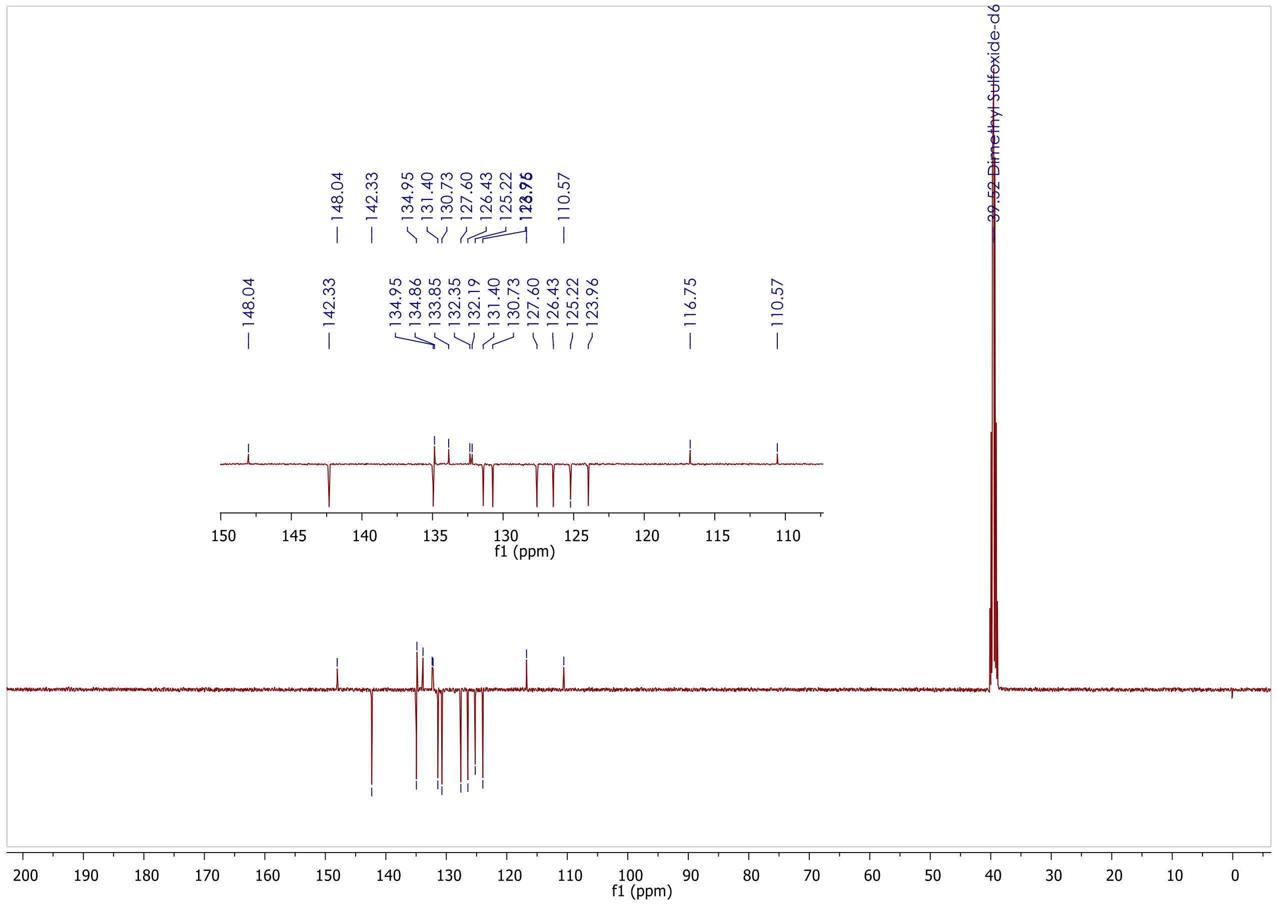

(*Z*)-2-(3,4-dichlorophenyl)-3-(4-nitrophenyl)acrylonitrilen (**1e**)

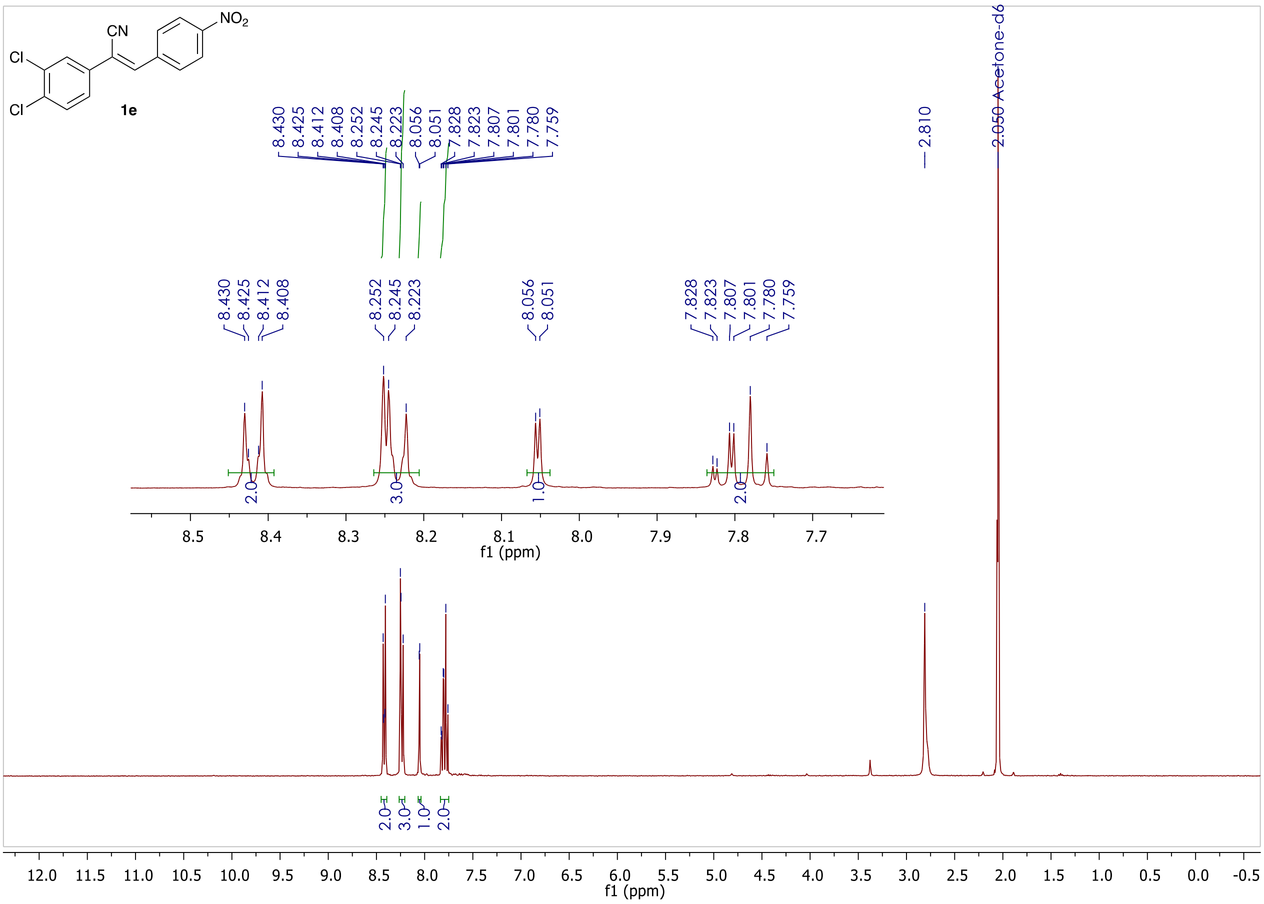

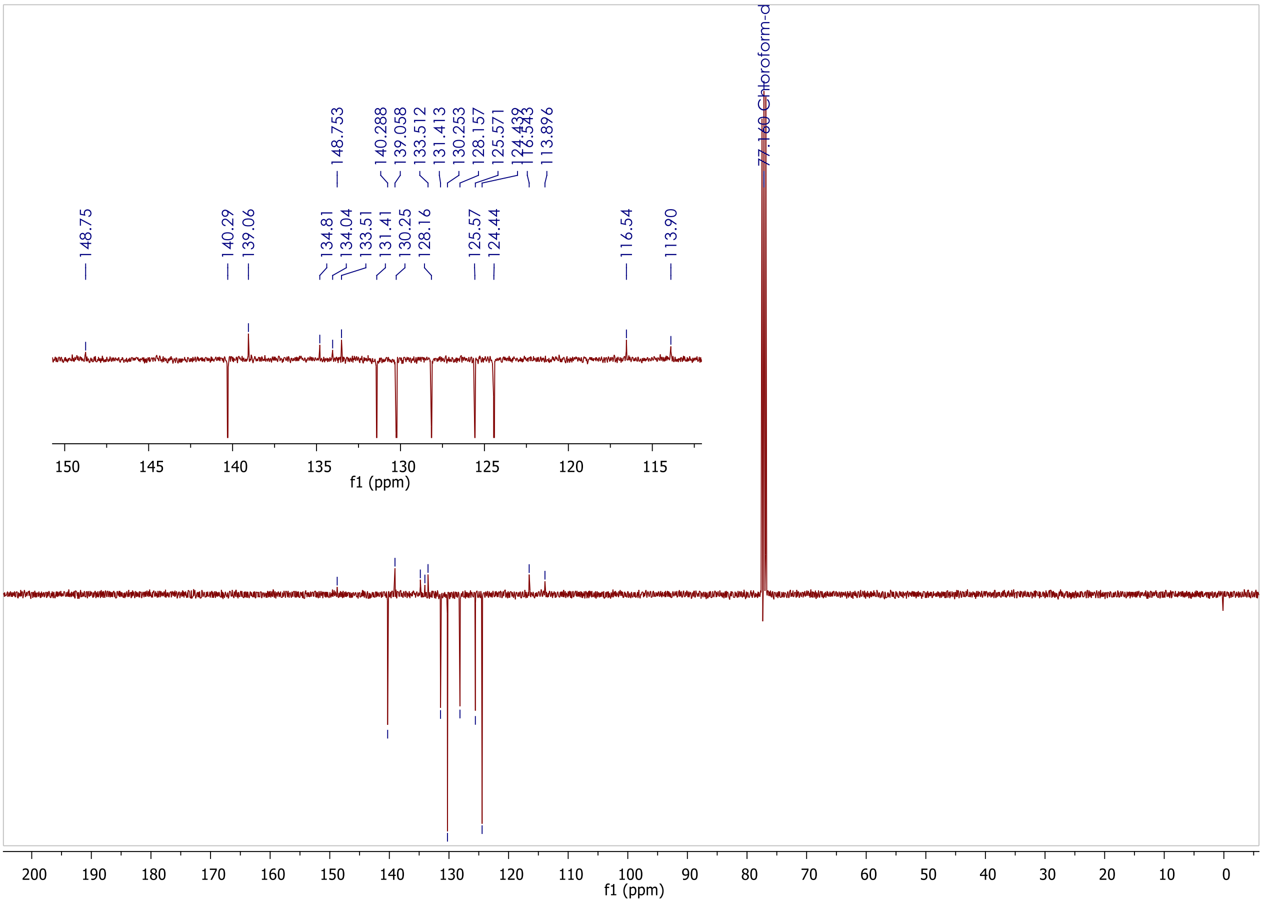

(*Z*)-3-(2-aminophenyl)-2-(3,4-dichlorophenyl)acrylonitrile (**1f**)

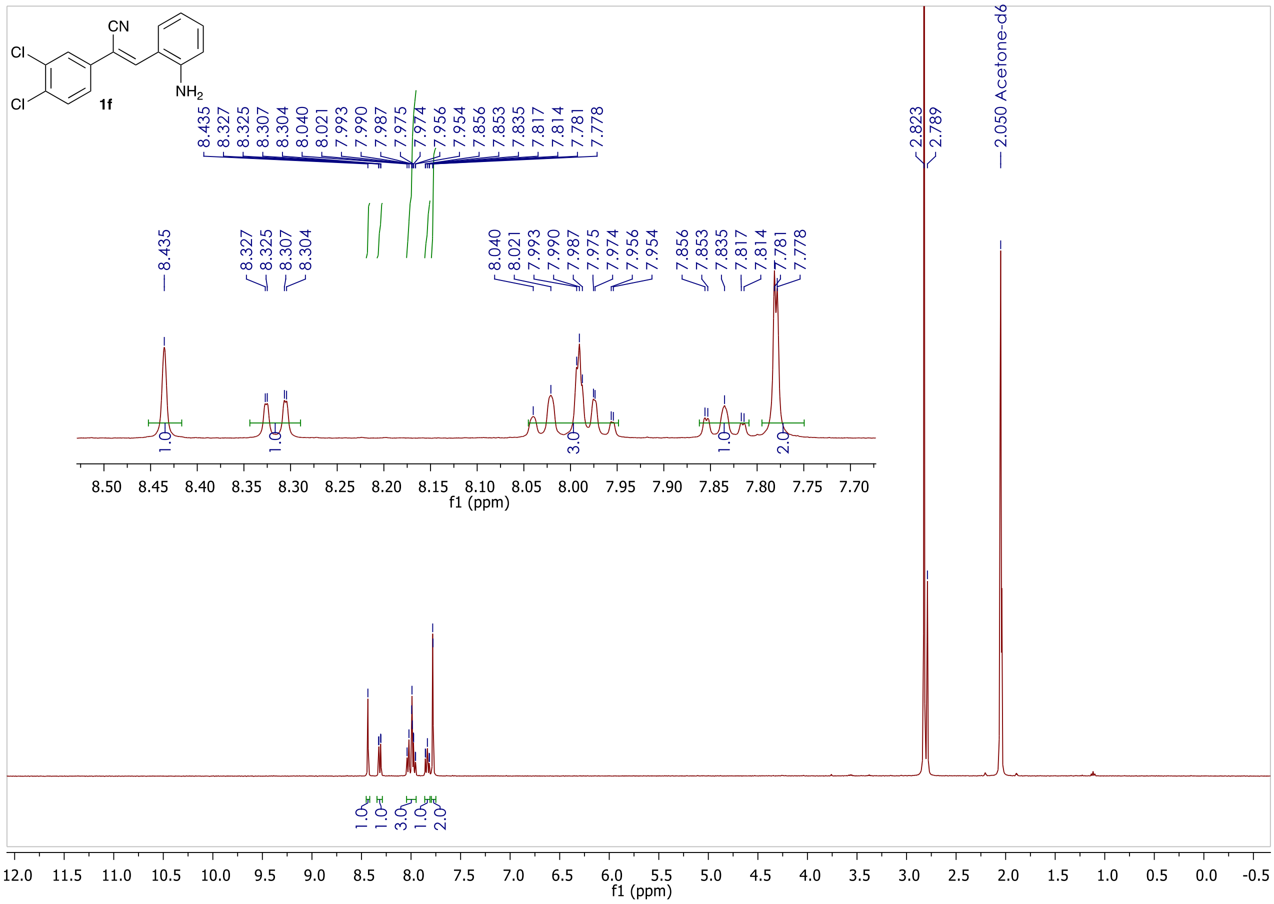

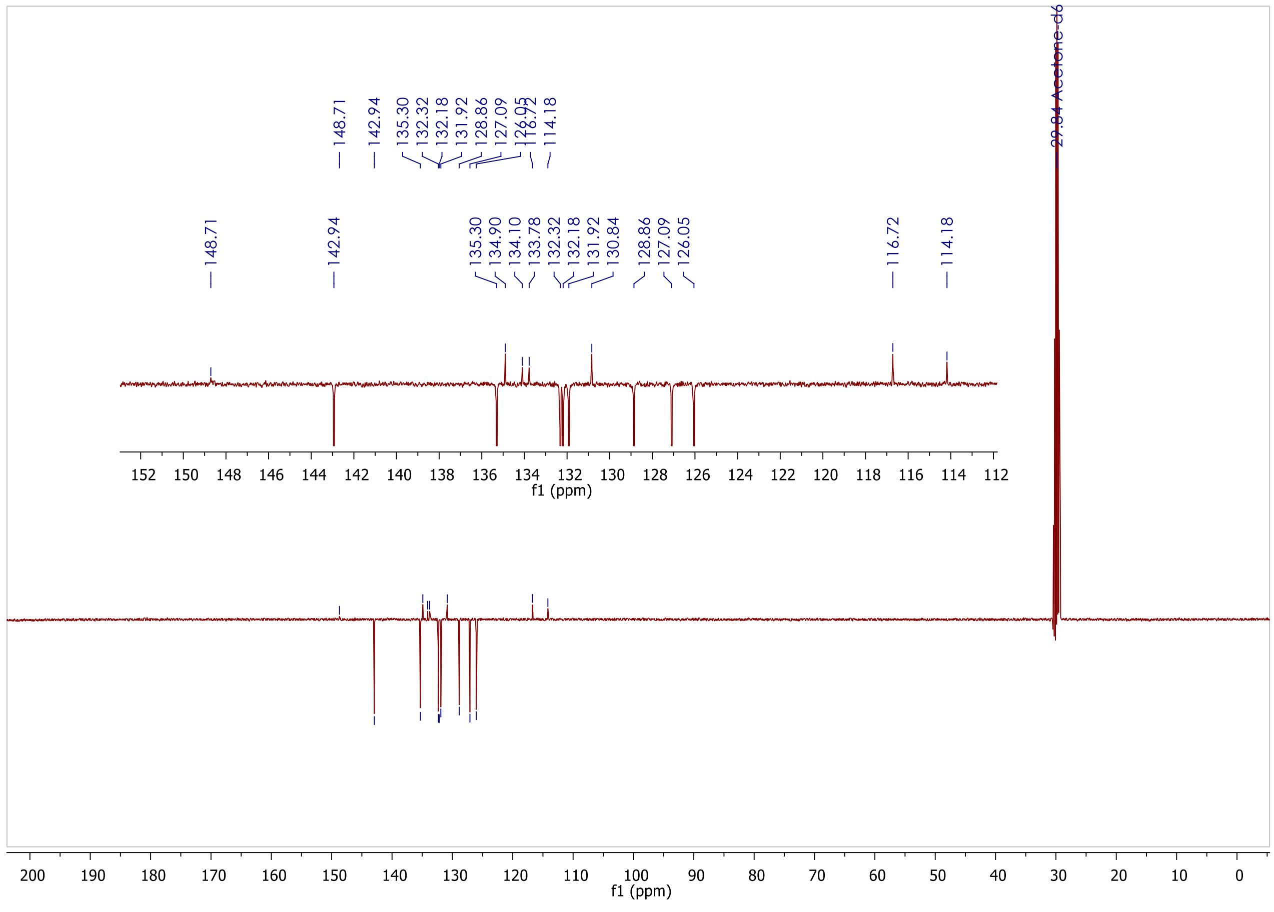

(*Z*)-3-(4-aminophenyl)-2-(3,4-dichlorophenyl)acrylonitrile (**1g**)

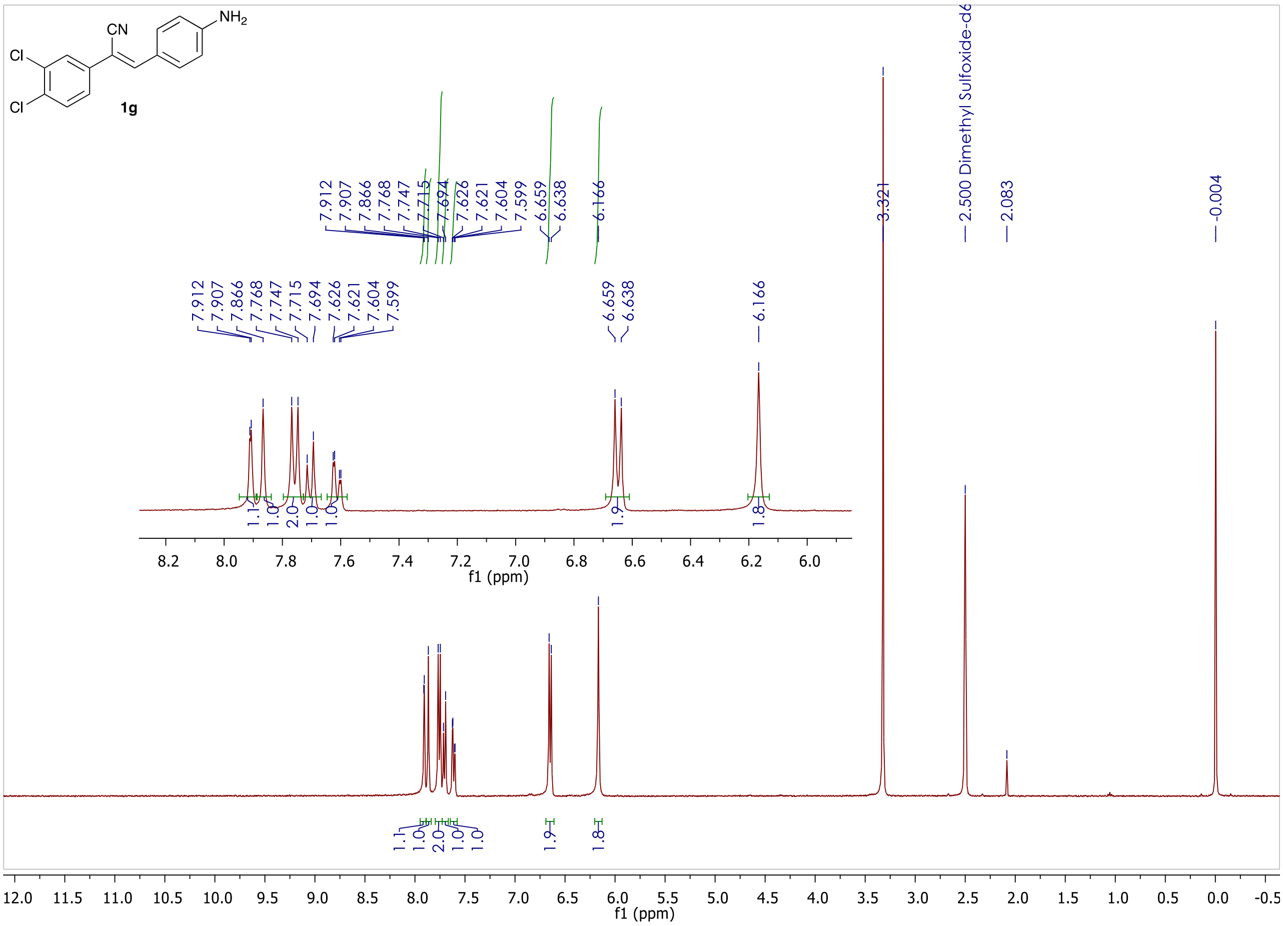

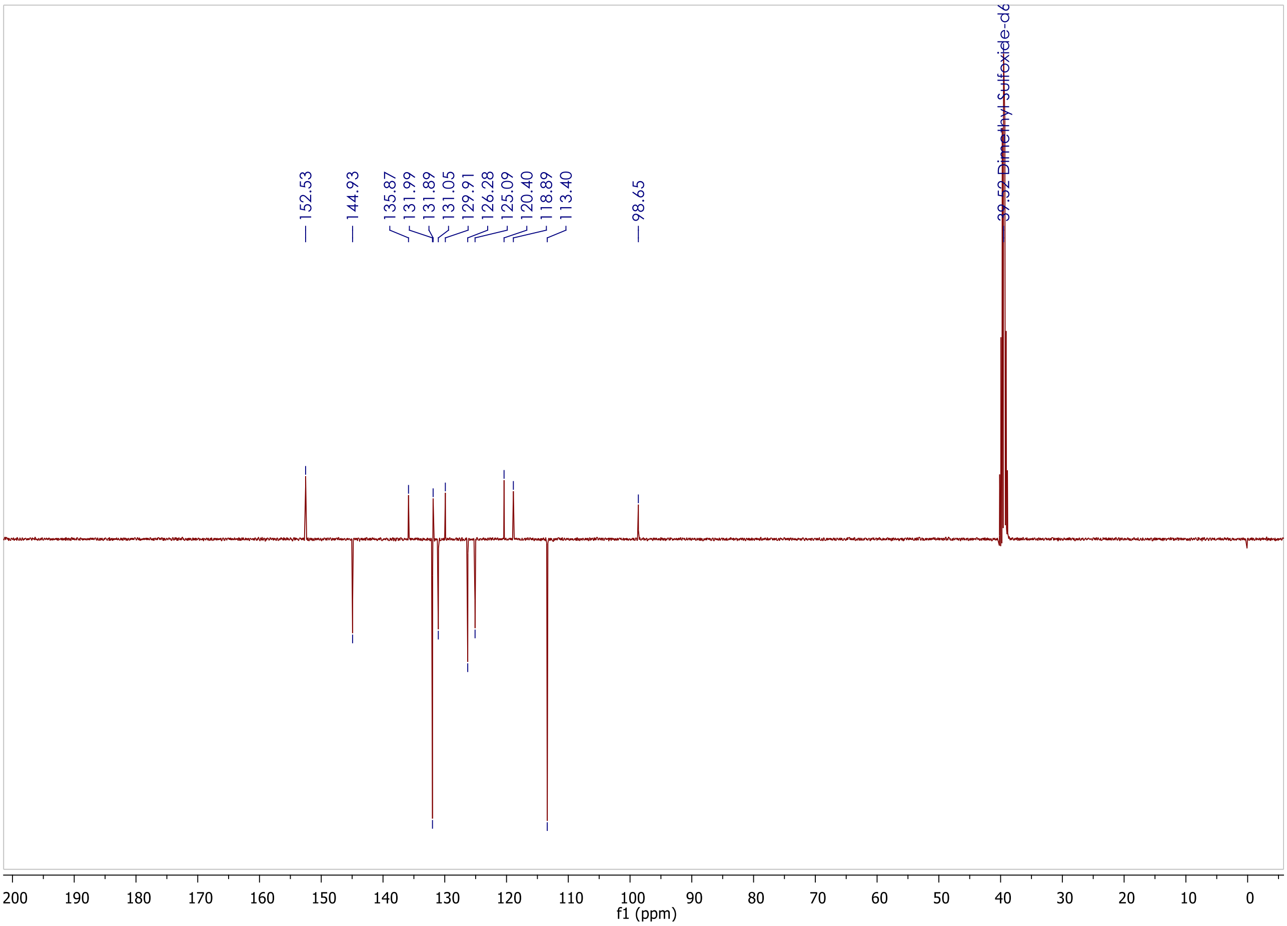

(*Z*)-2-(3,4-dichlorophenyl)-3-(4-(methylamino)phenyl)acrylonitrile (**1h**)

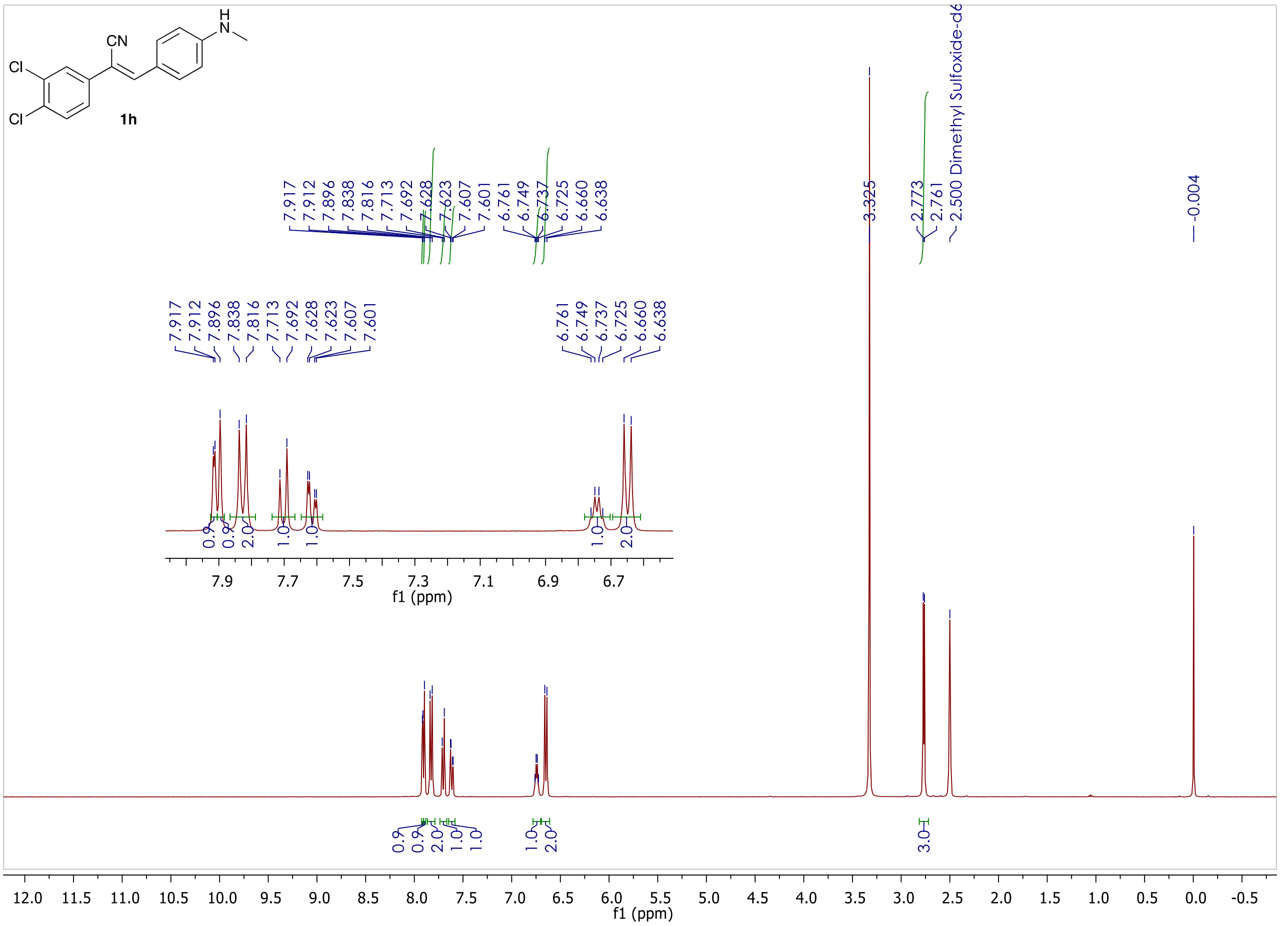

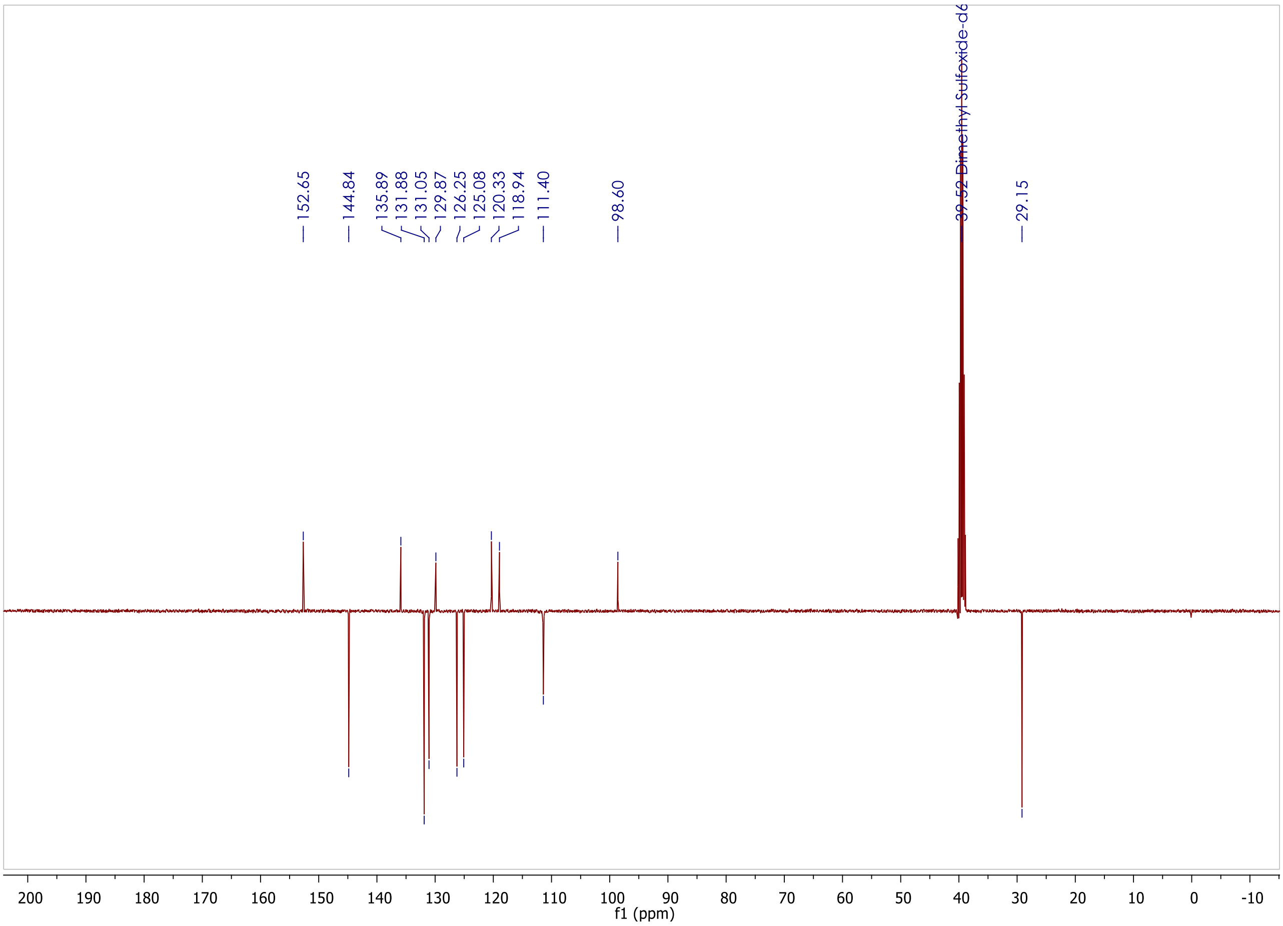

(*Z*)-2-(3,4-dichlorophenyl)-3-(4-(dimethylamino)phenyl)acrylonitrile (**1i**)

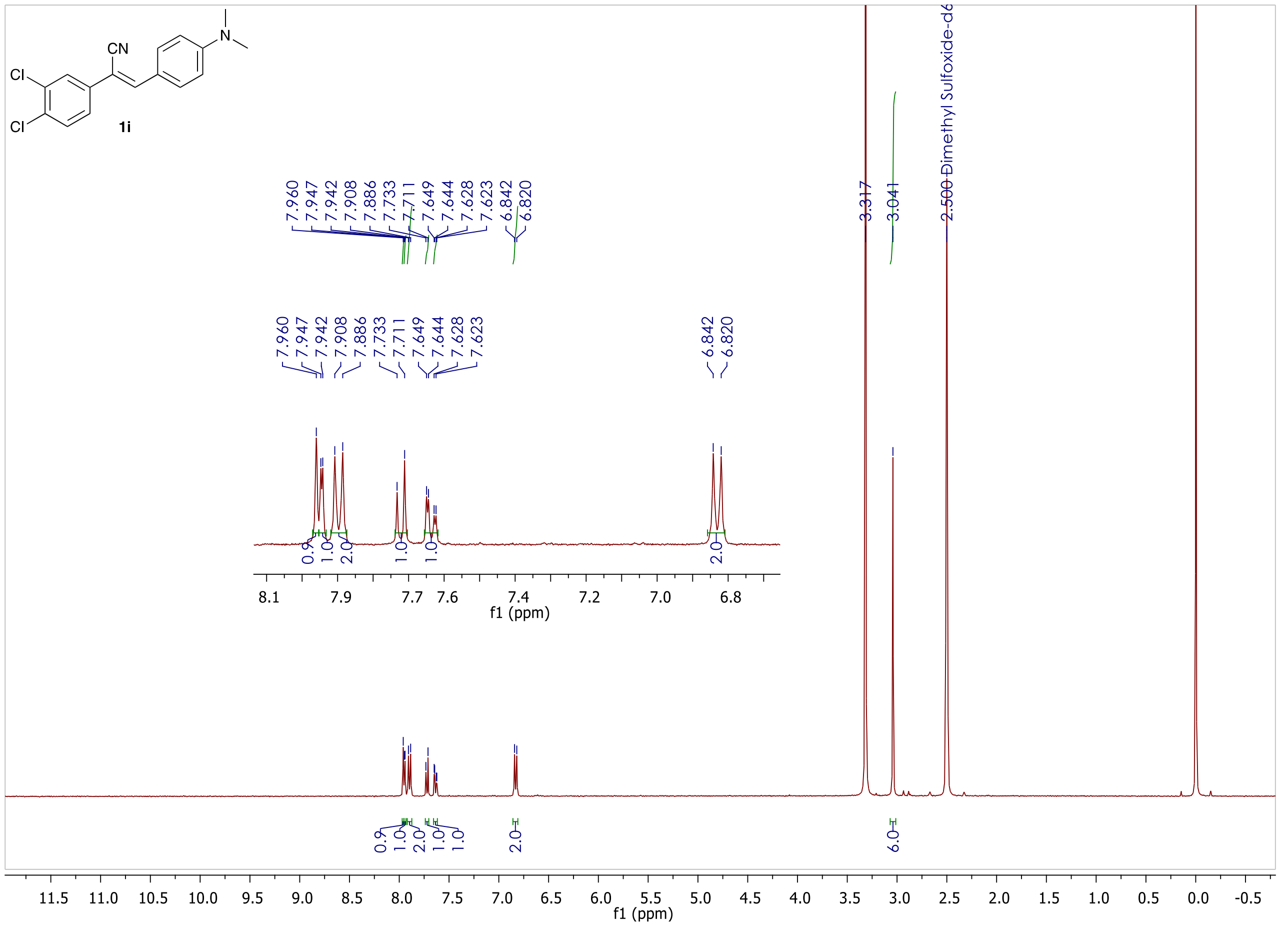

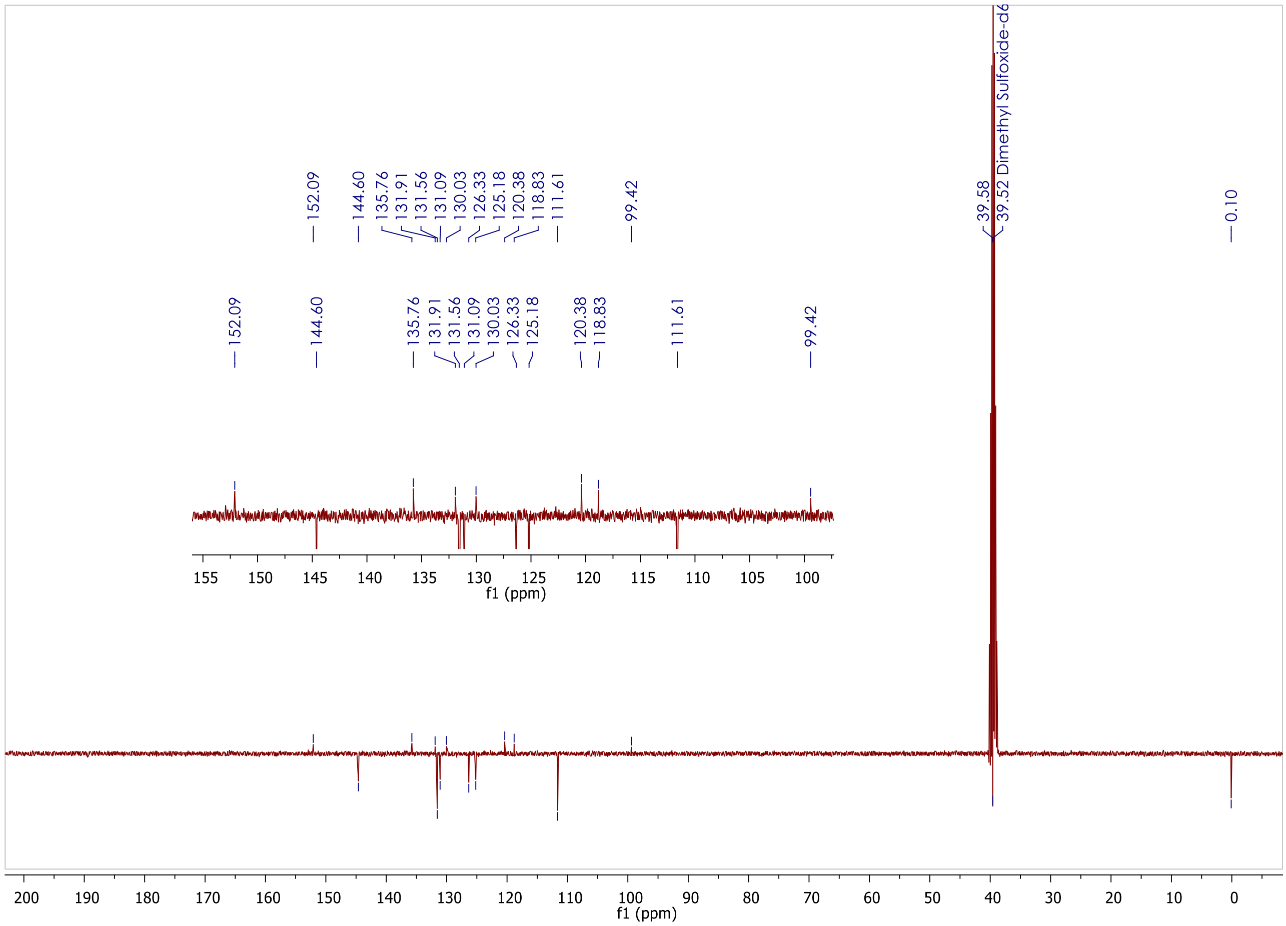

(*Z*)-2-(3,4-dichlorophenyl)-3-(4-(3-(dimethylamino)-2-hydroxypropoxy)phenyl)acrylonitrile (**1j**)

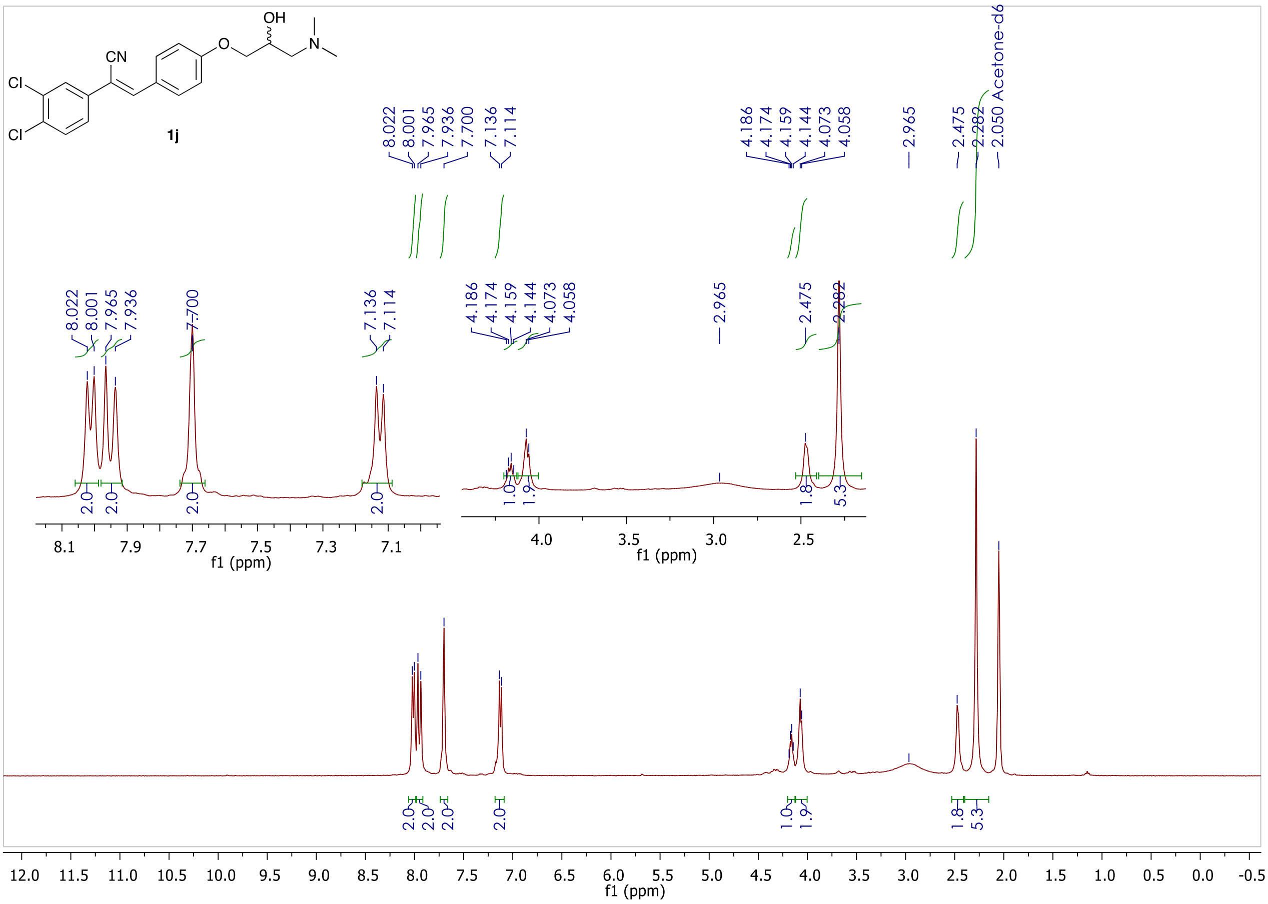

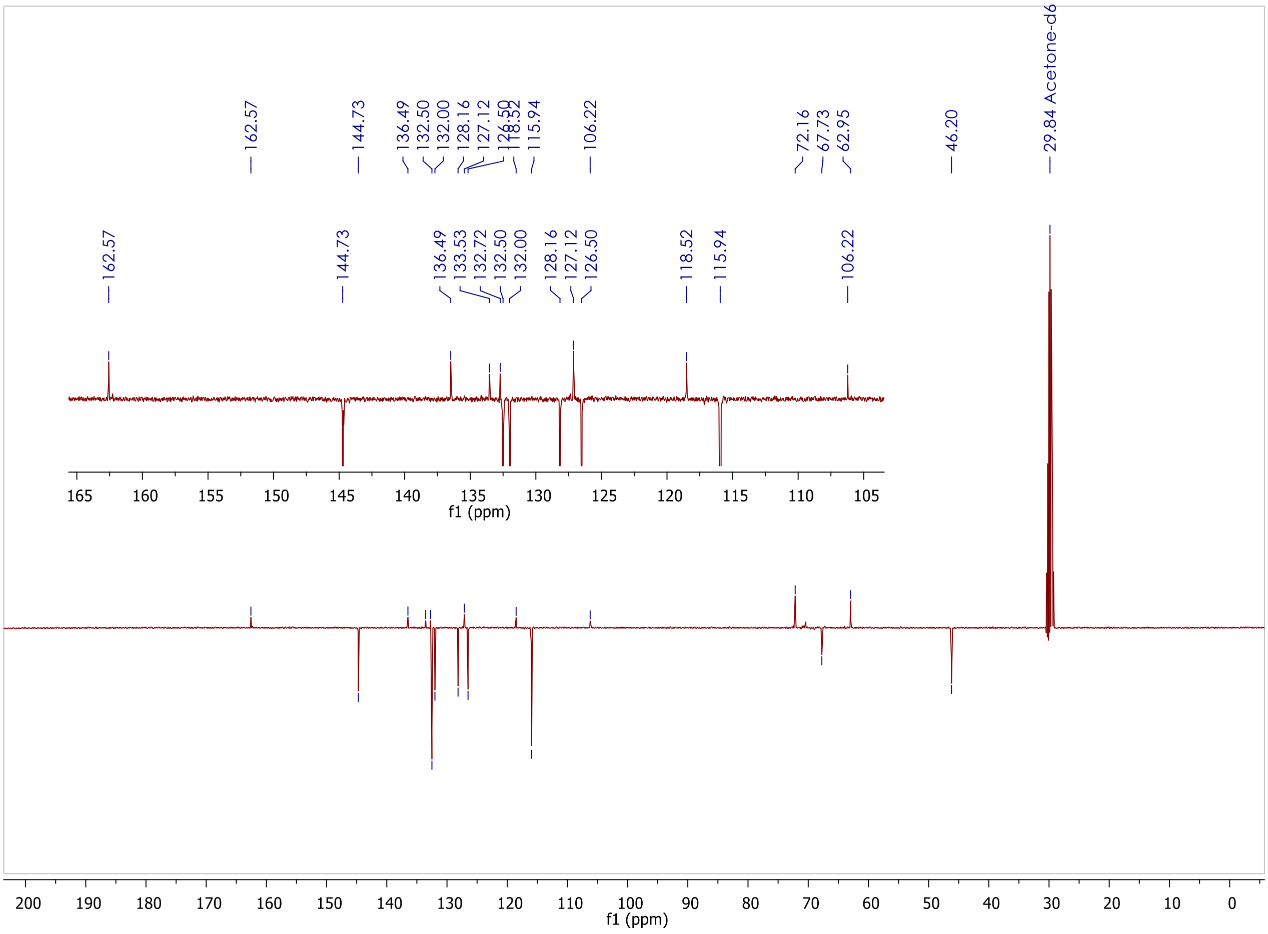

(*Z*)-3-(4-bromo-3-nitrophenyl)-2-(3,4-dichlorophenyl)acrylonitrile (**1k**)

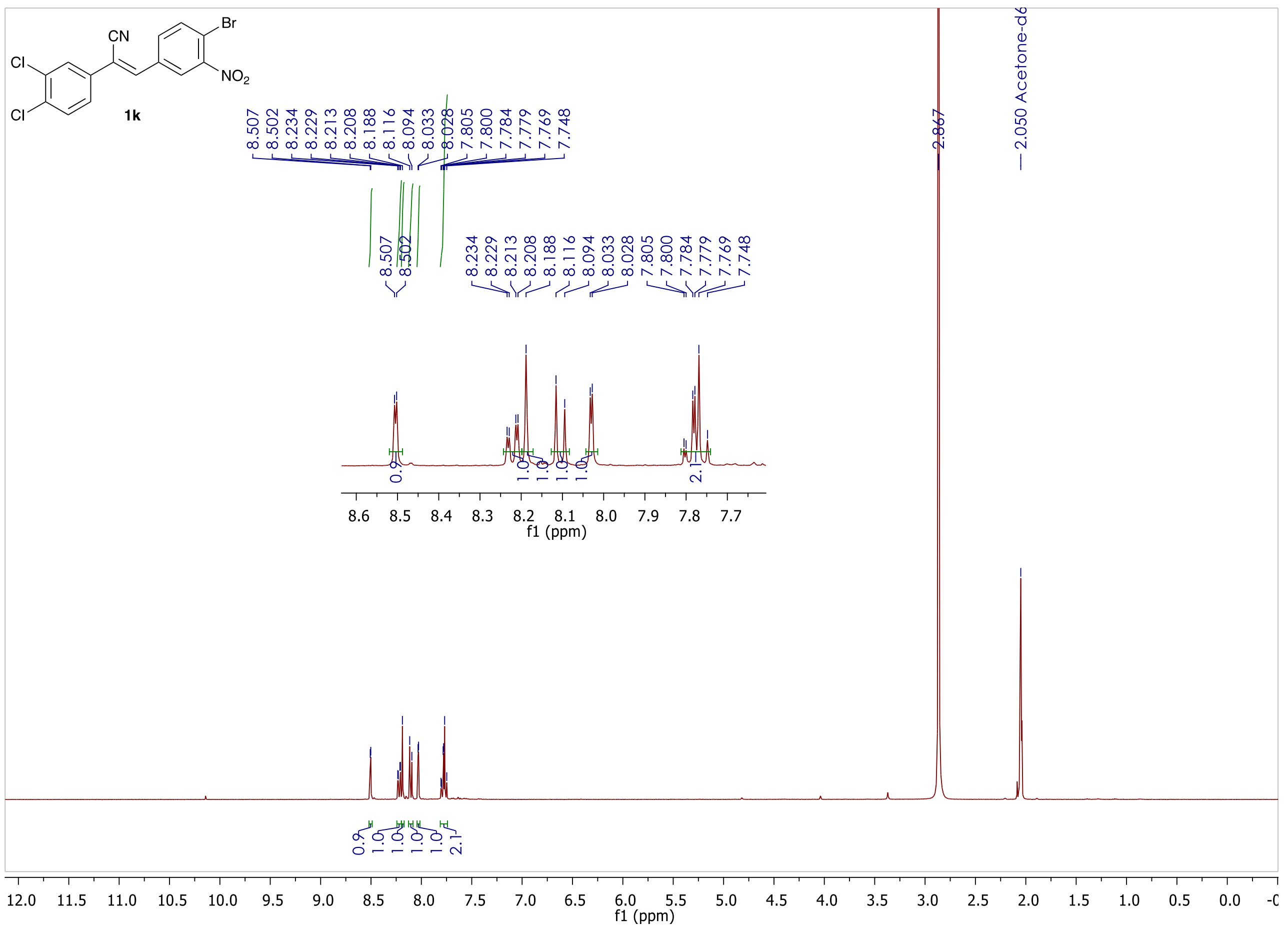

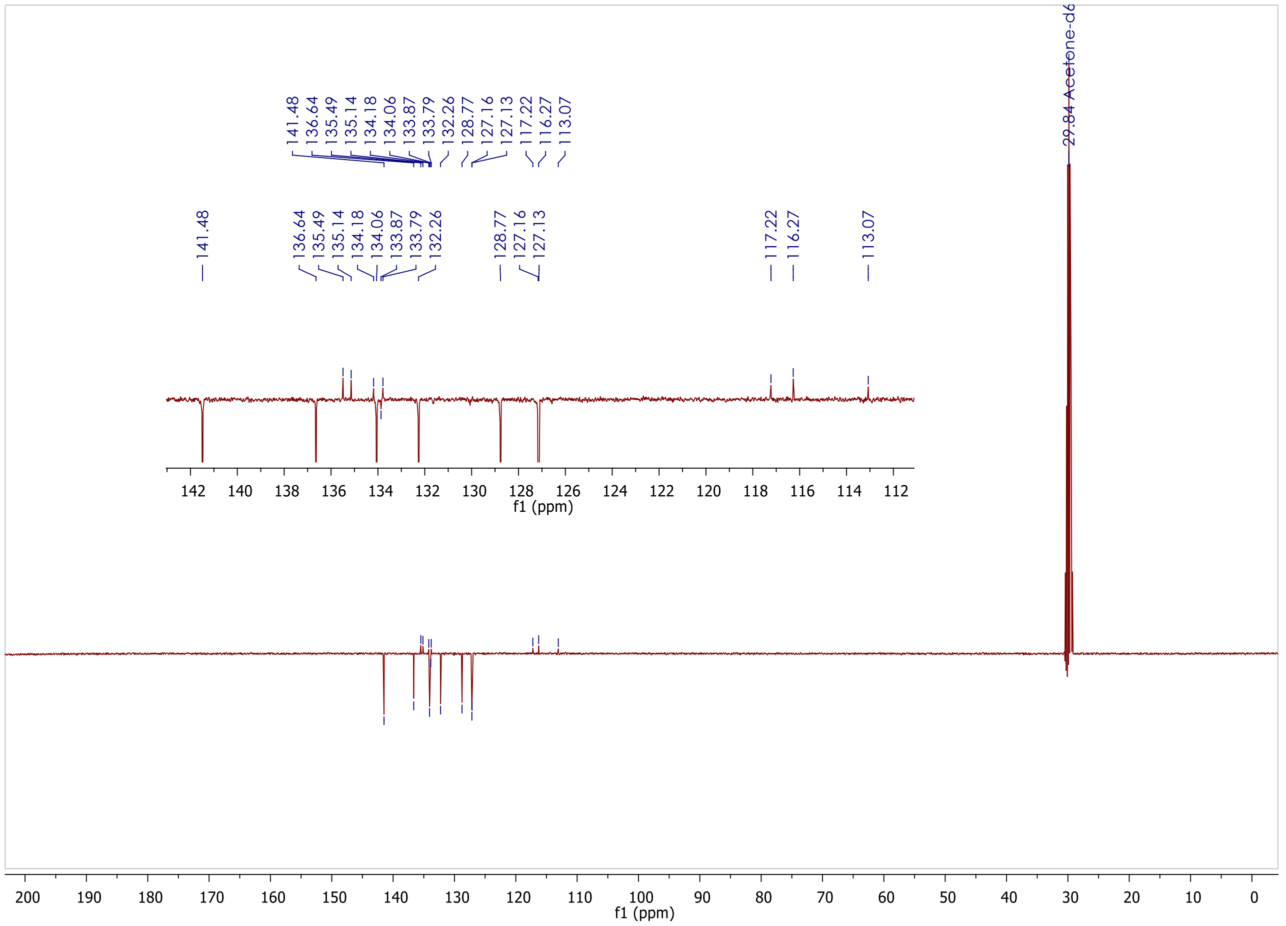

(*Z*)-3-(2-amino-4-bromophenyl)-2-(3,4-dichlorophenyl)acrylonitrile (**1l**)

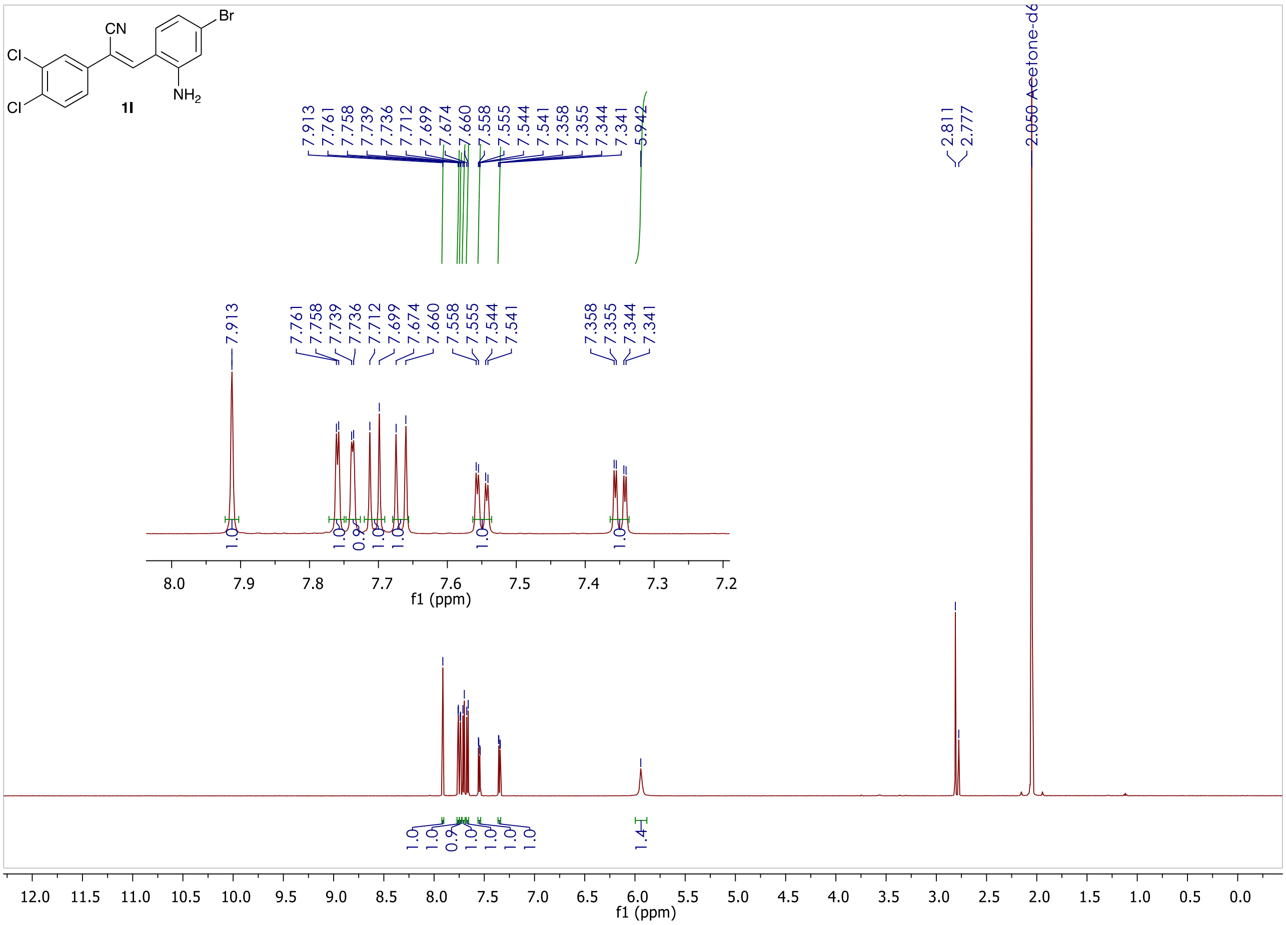

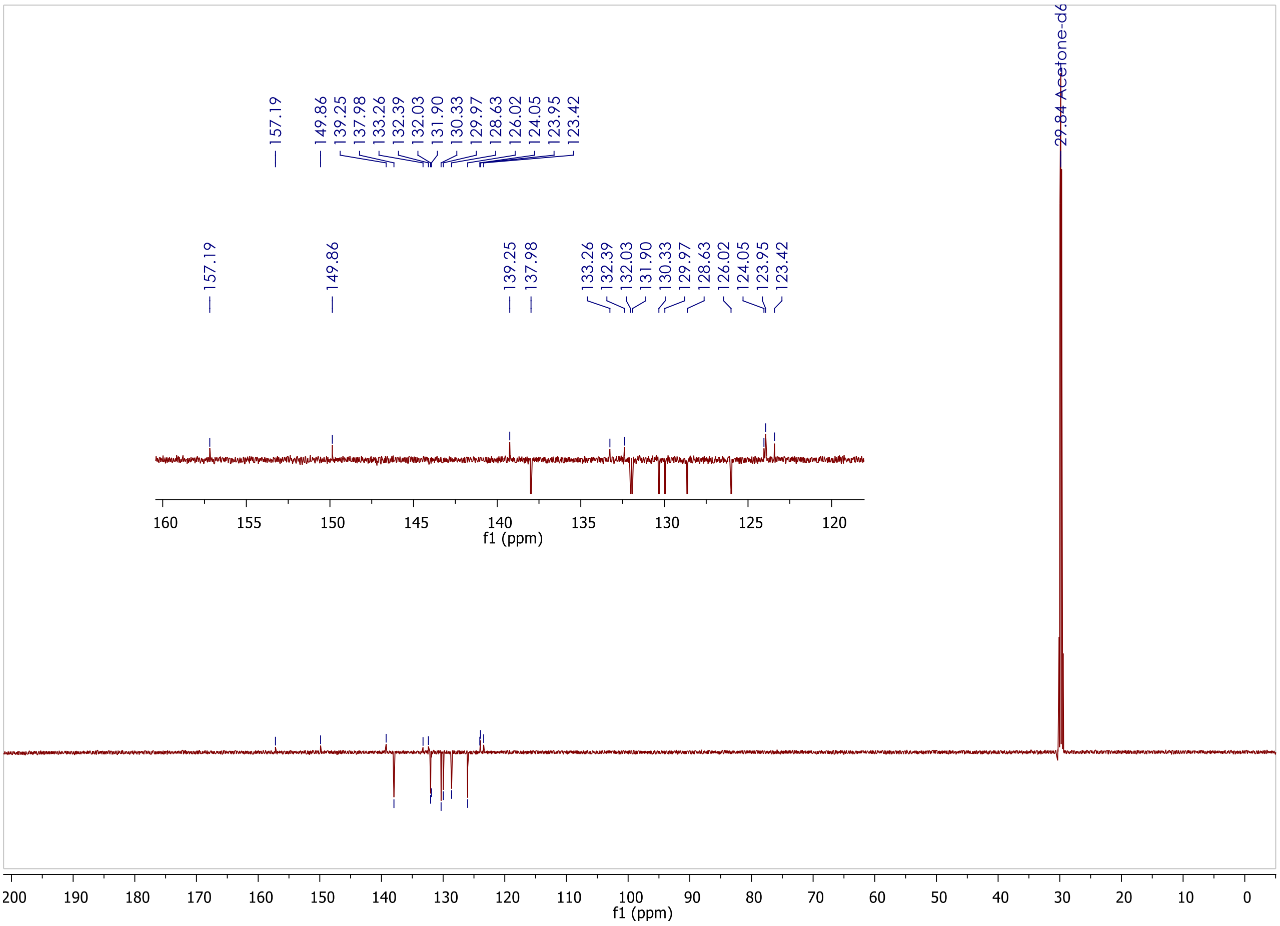

(*Z*)-2-(3,4-dichlorophenyl)-3-(4-hydroxy-3-methoxy-5-nitrophenyl)acrylonitrile (**1m**)

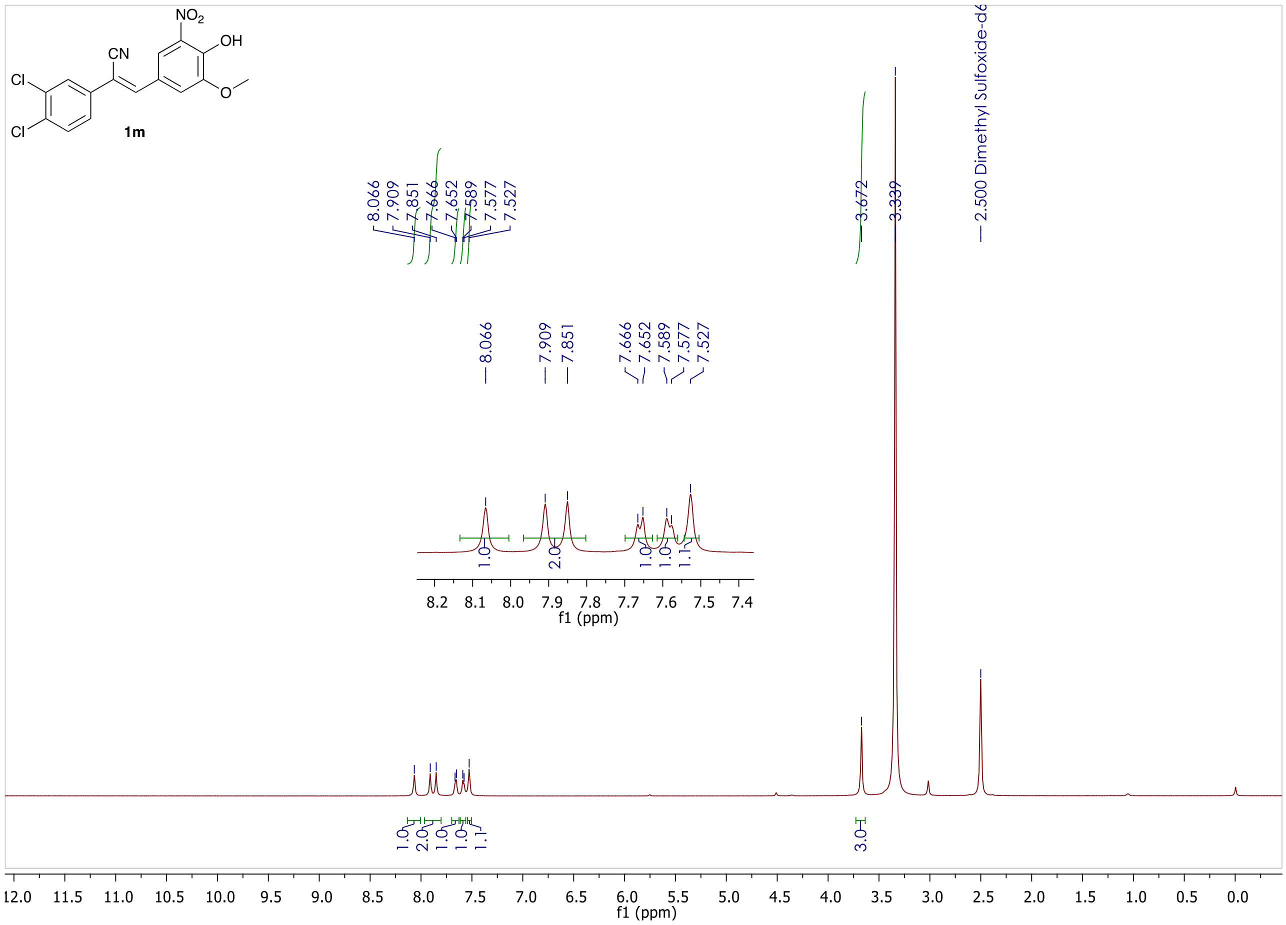

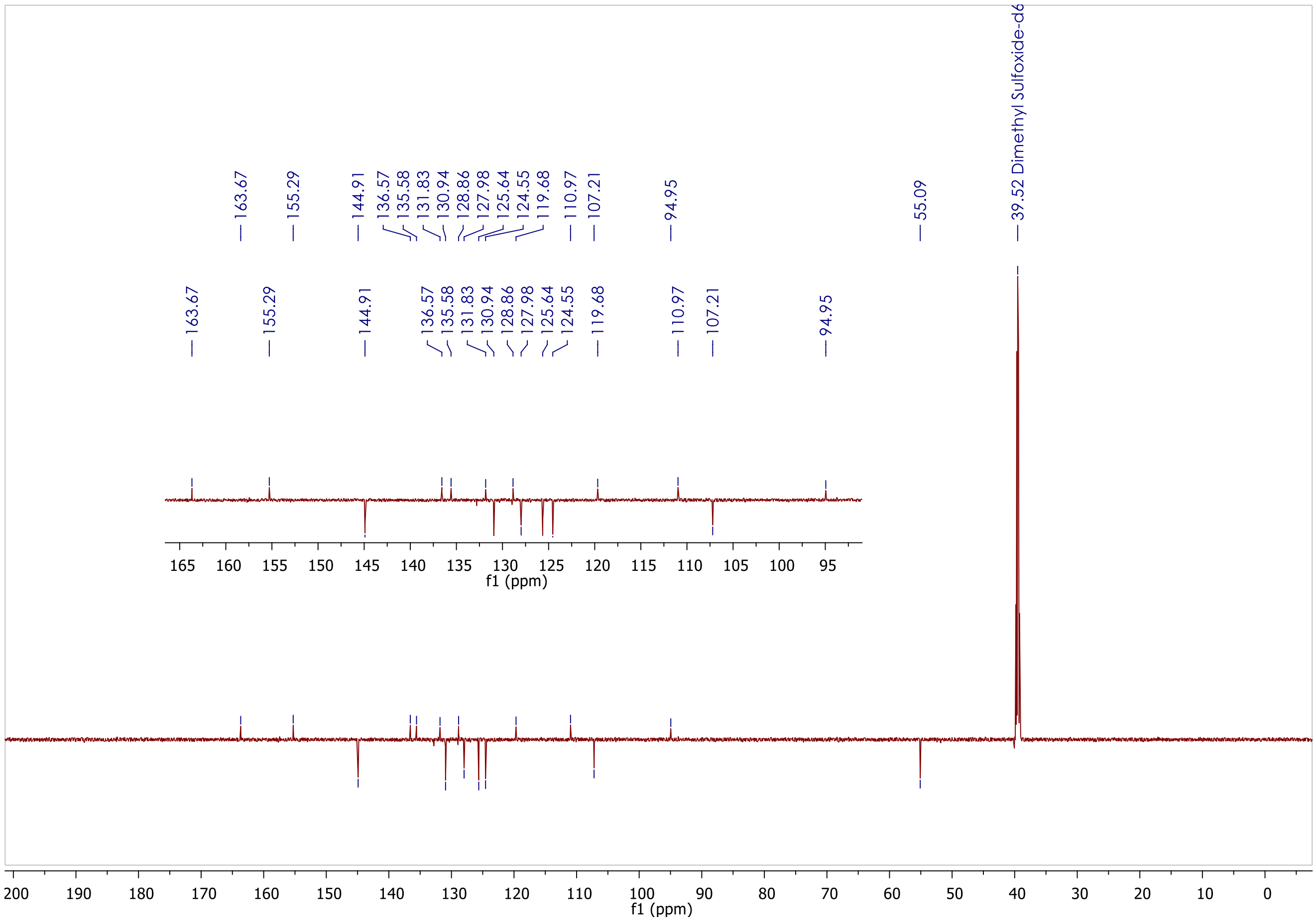

(*Z*)-3-(4-bromo-1*H*-pyrrol-2-yl)-2-(3,4-dichlorophenyl)acrylonitrile (**1n**)

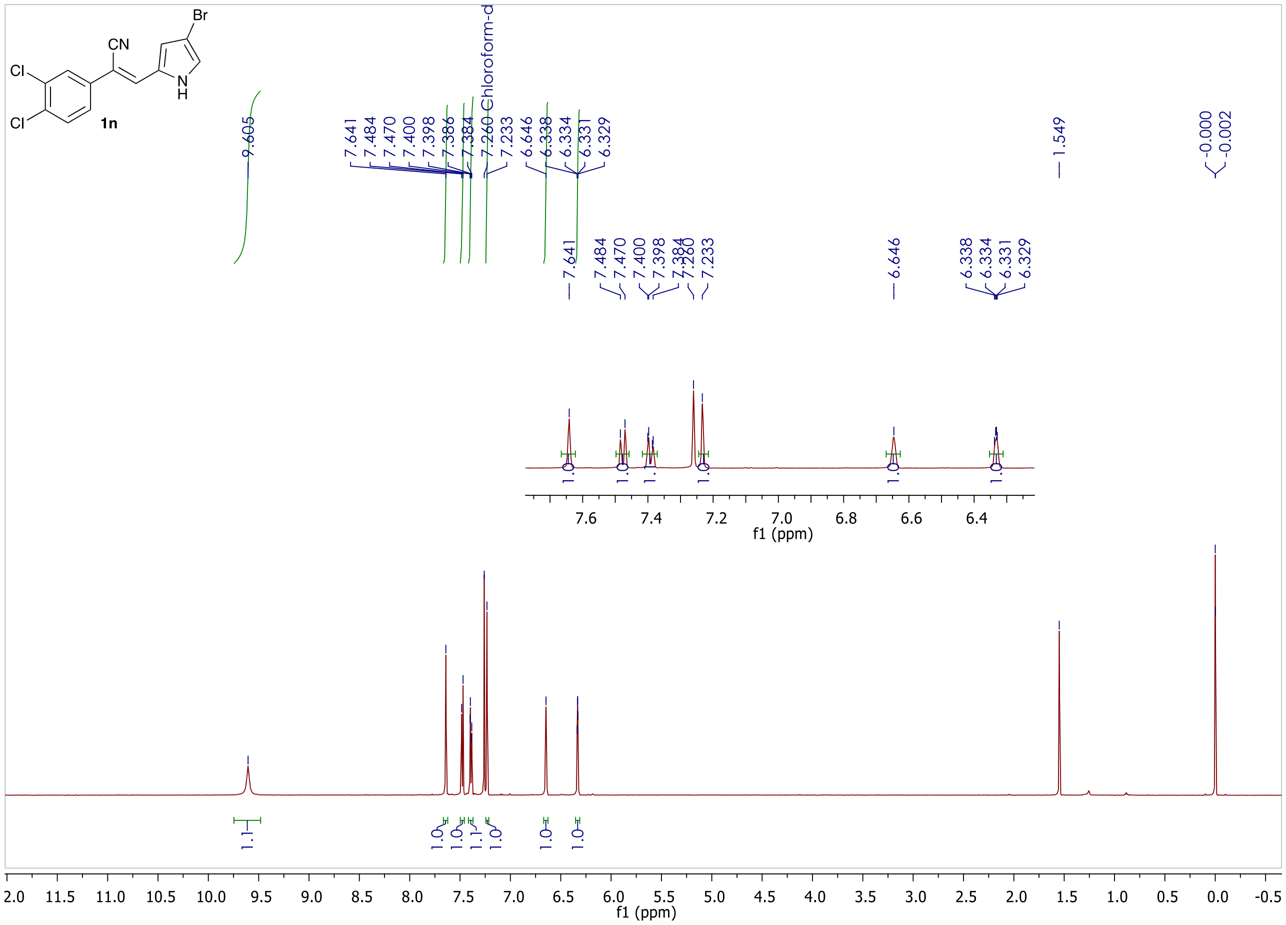

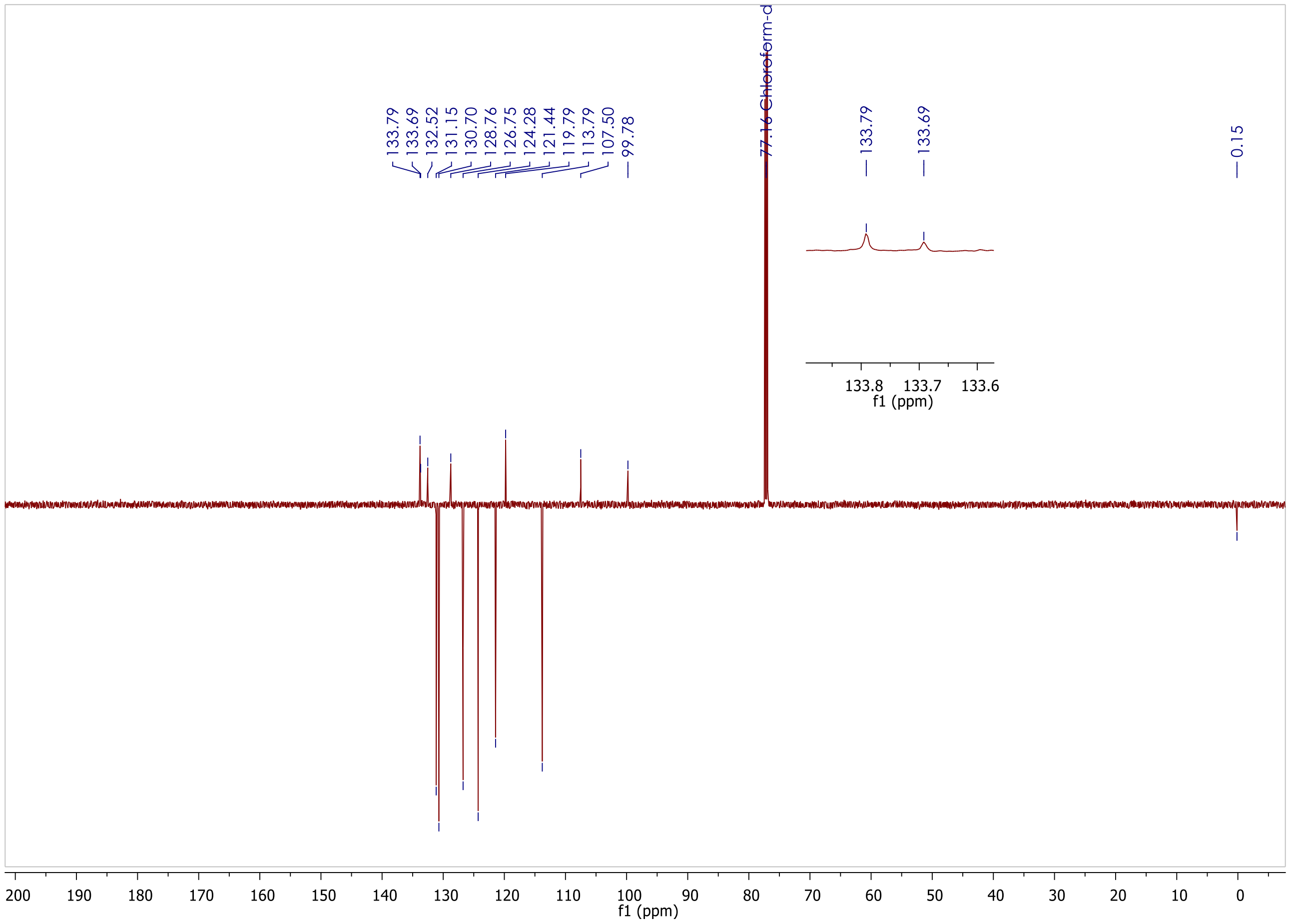

(*Z*)-3-(5-bromo-1*H*-pyrrol-2-yl)-2-(3,4-dichlorophenyl)acrylonitrile (**1o**)

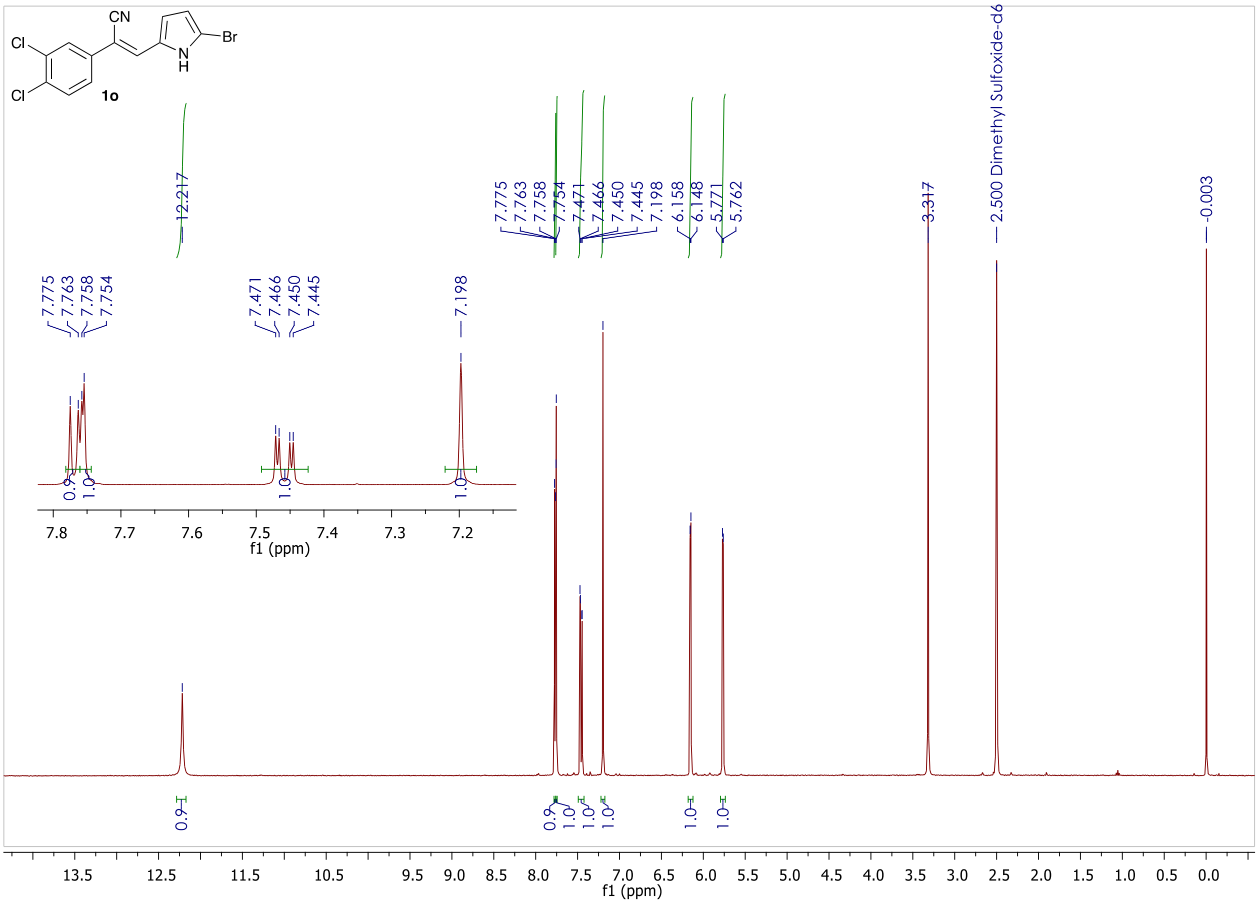

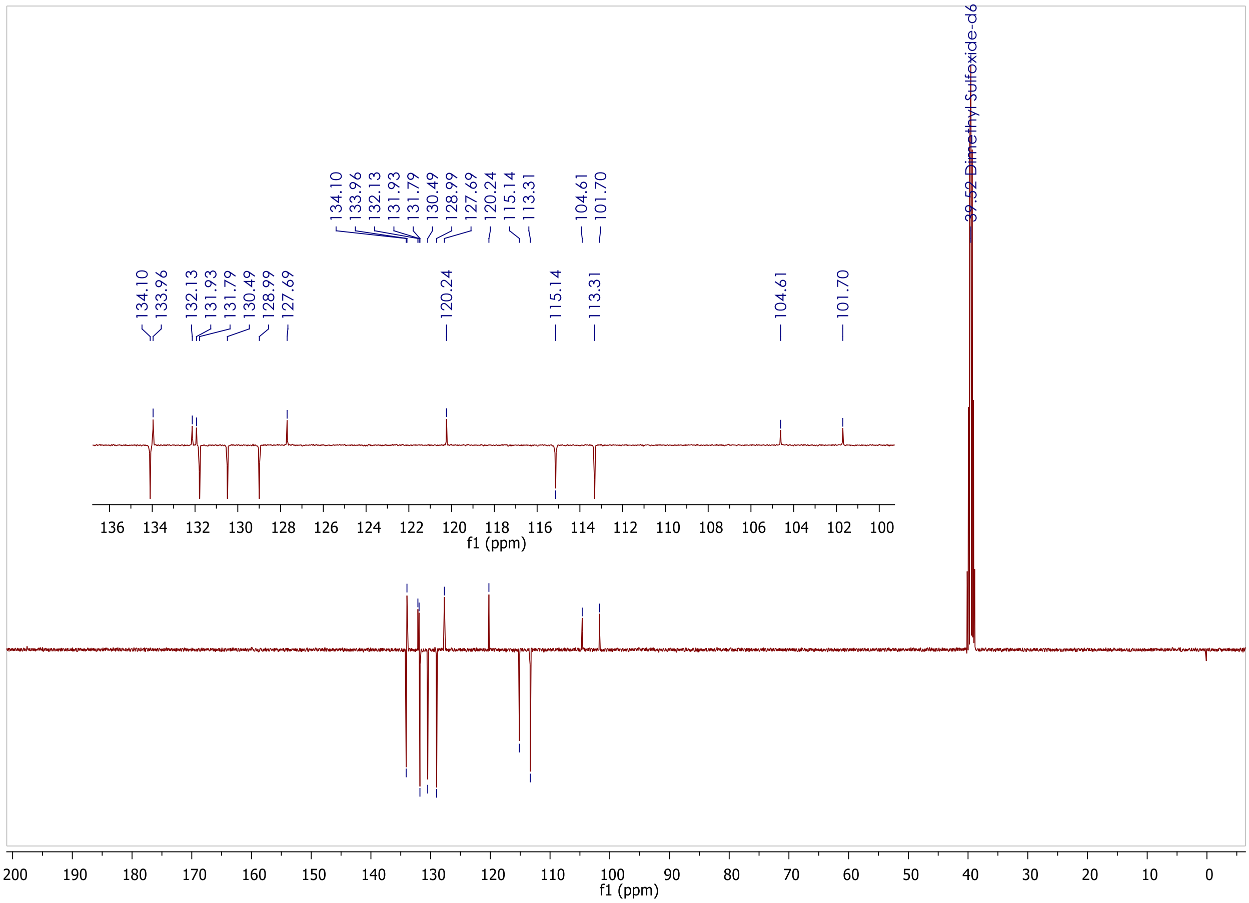

(*Z*)-3-(4,5-dibromo-1*H*-pyrrol-2-yl)-2-(3,4-dichlorophenyl)acrylonitrile (**1p**)

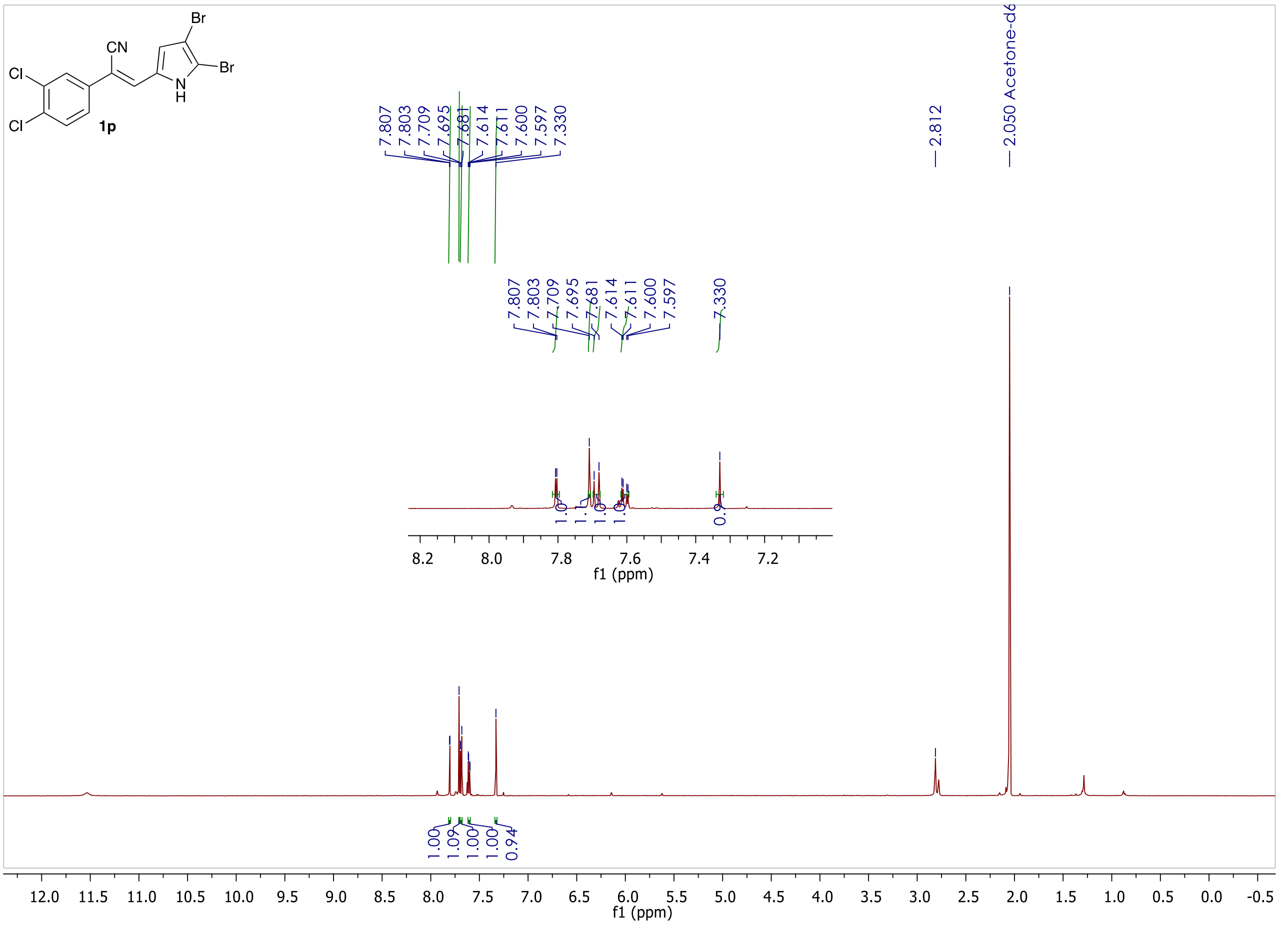

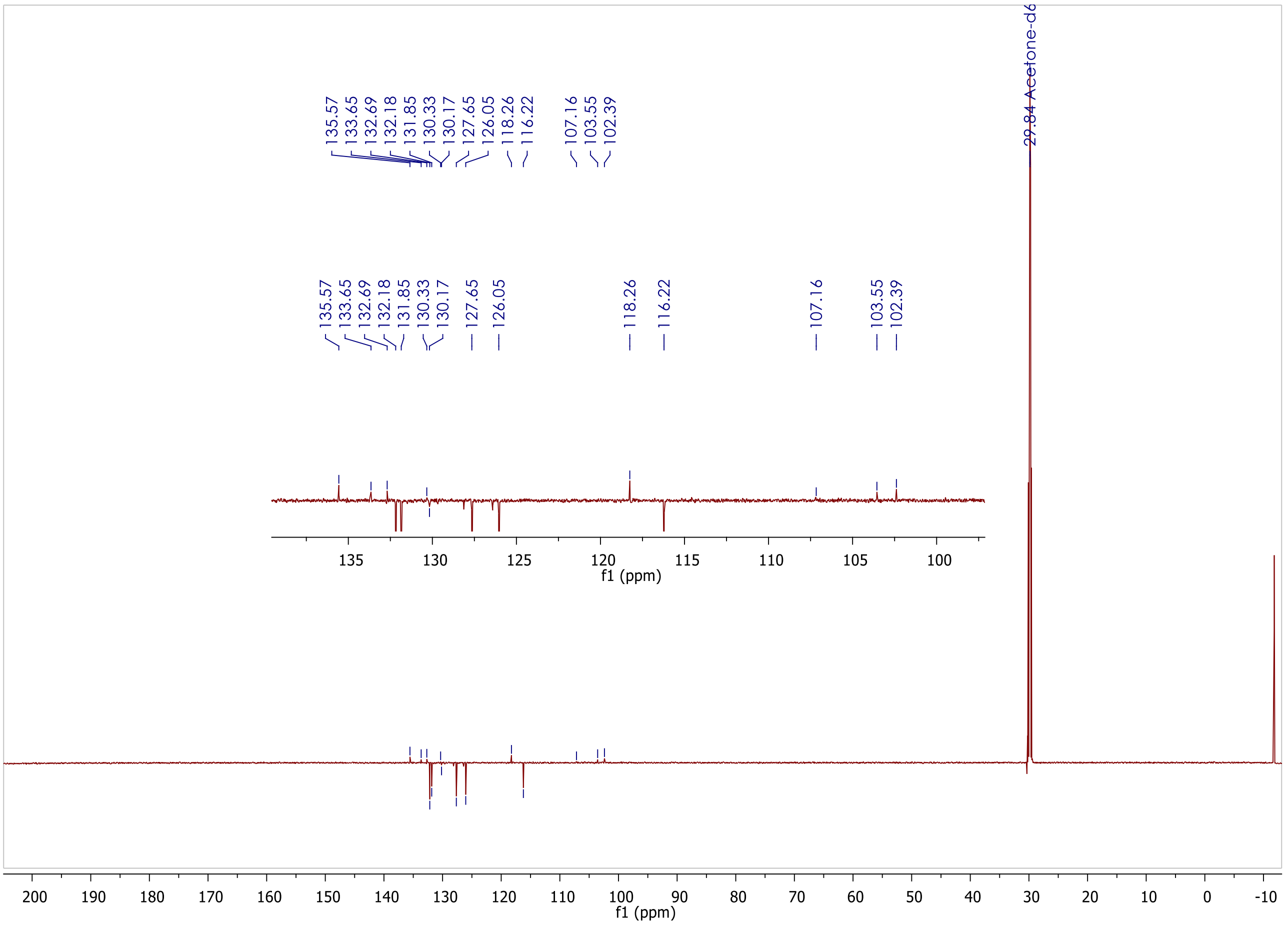

(*Z*)-2-(3,4-dichlorophenyl)-3-(3,4,5-tribromo-1*H*-pyrrol-2-yl)acrylonitrile (**1q**)

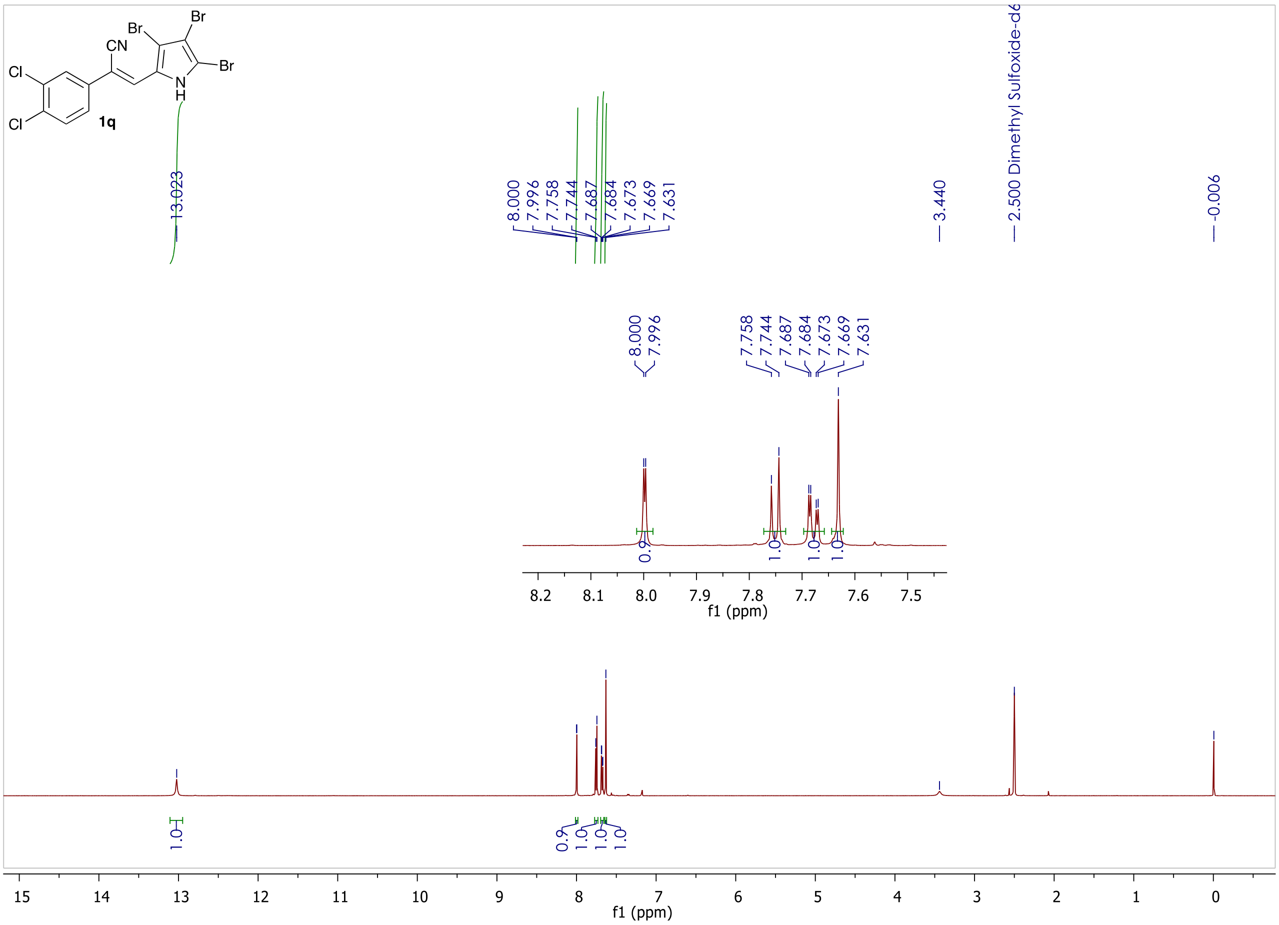

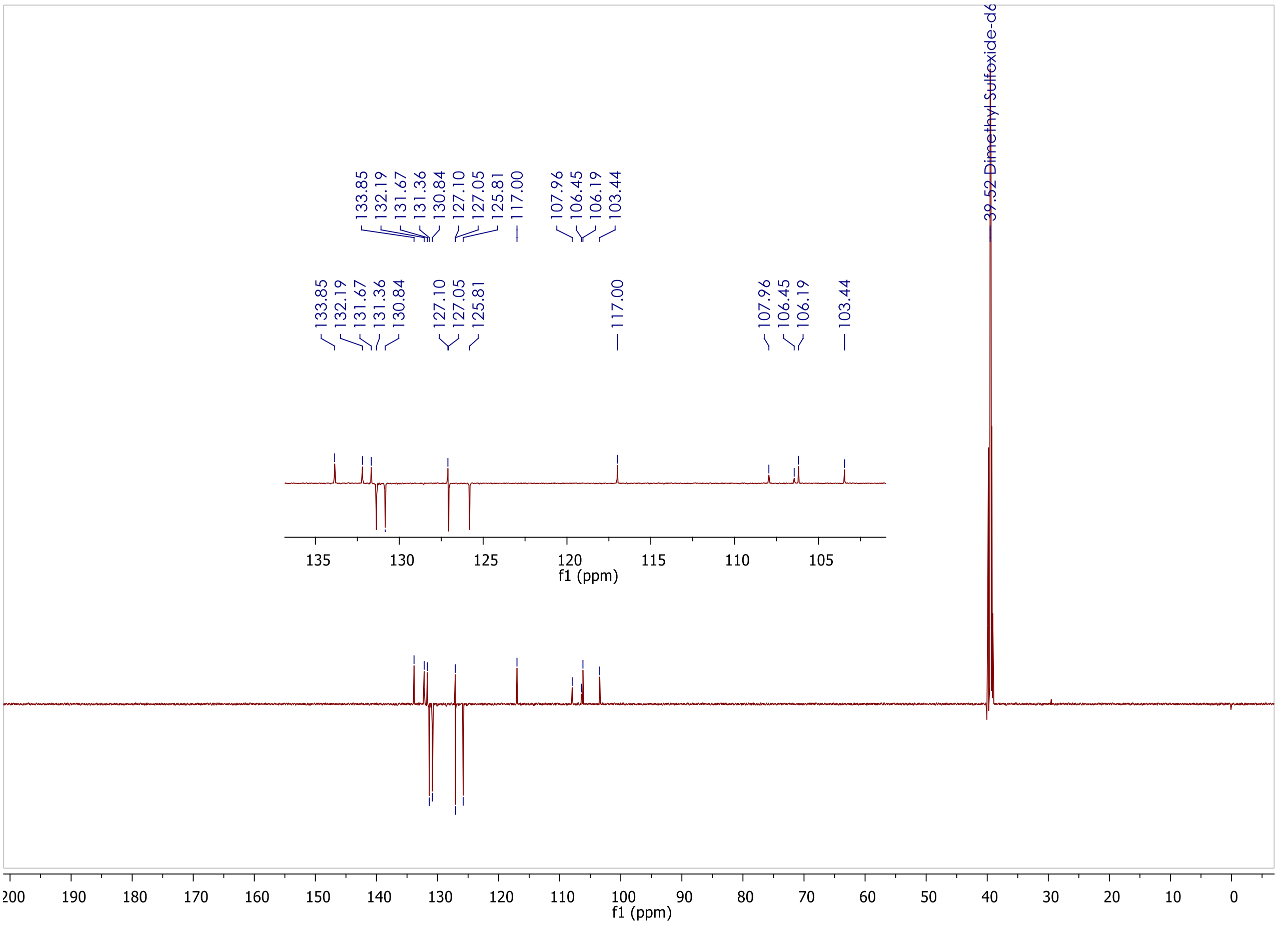

(*Z*)-3-(1*H*-benzo[*d*]imidazol-5-yl)-2-(3,4-dichlorophenyl)acrylonitrile (**1r**)

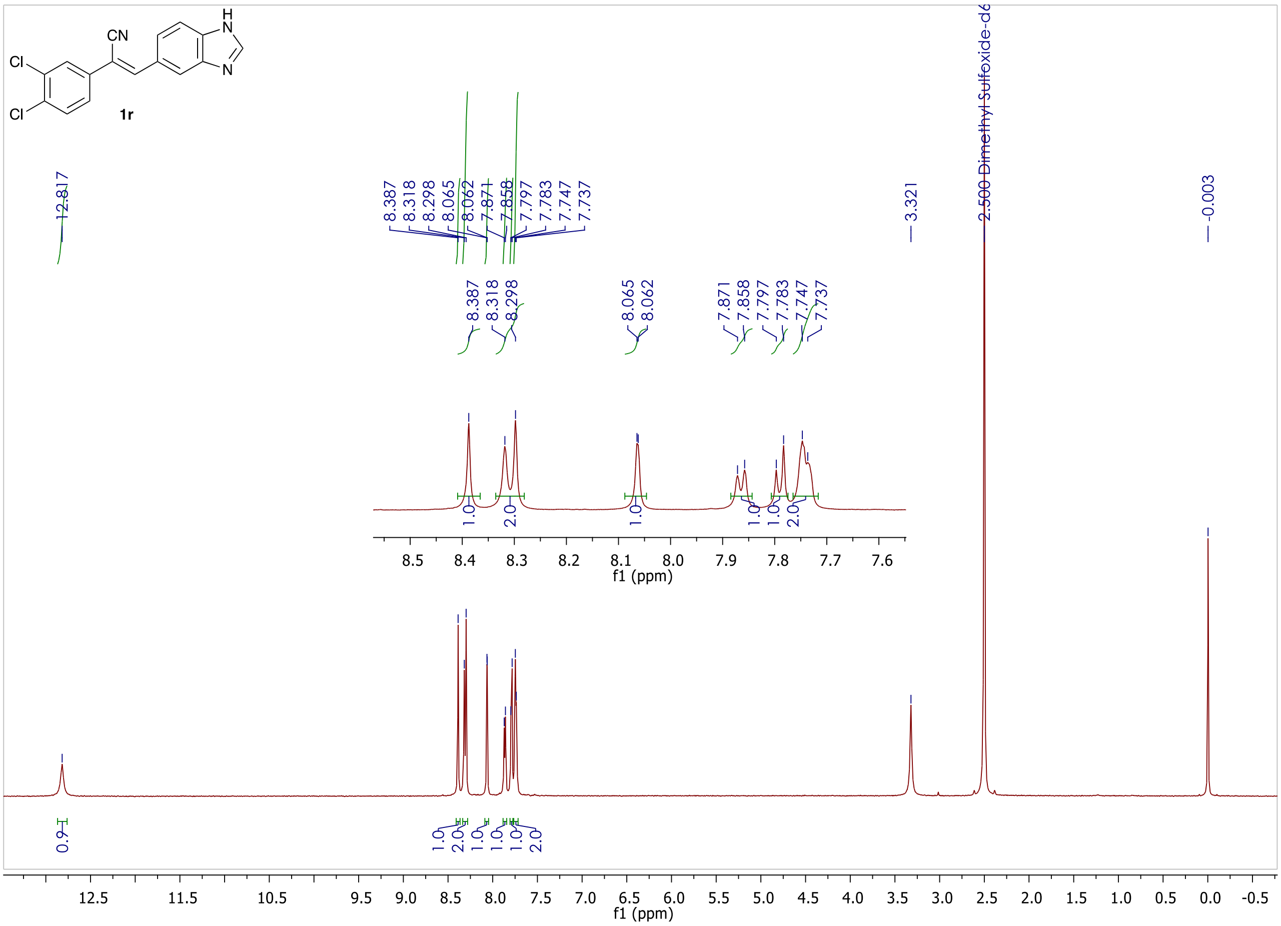

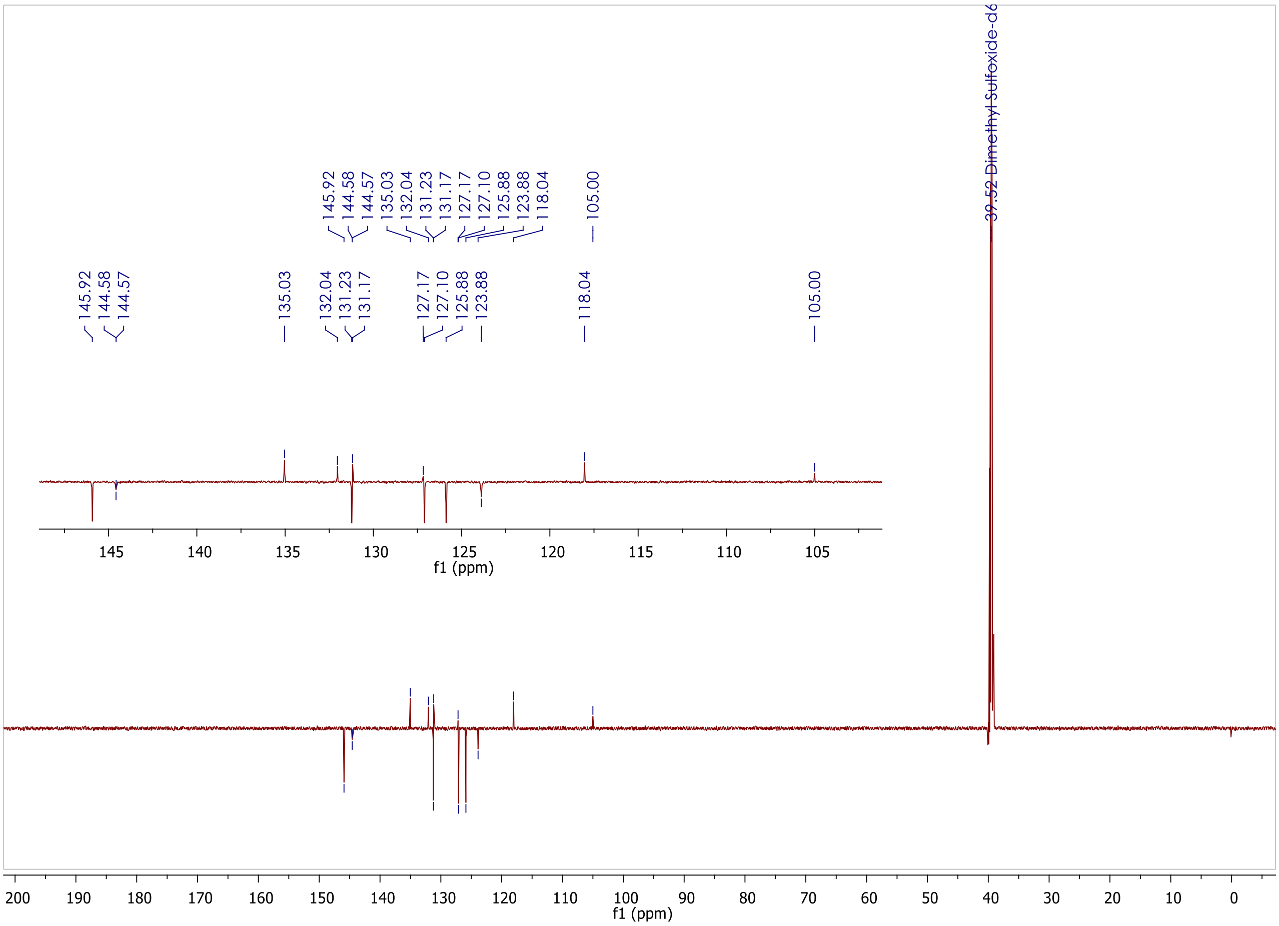

(*Z*)-2-(2,6-dichlorophenyl)-3-(2-nitrophenyl)acrylonitrile (**1s**)

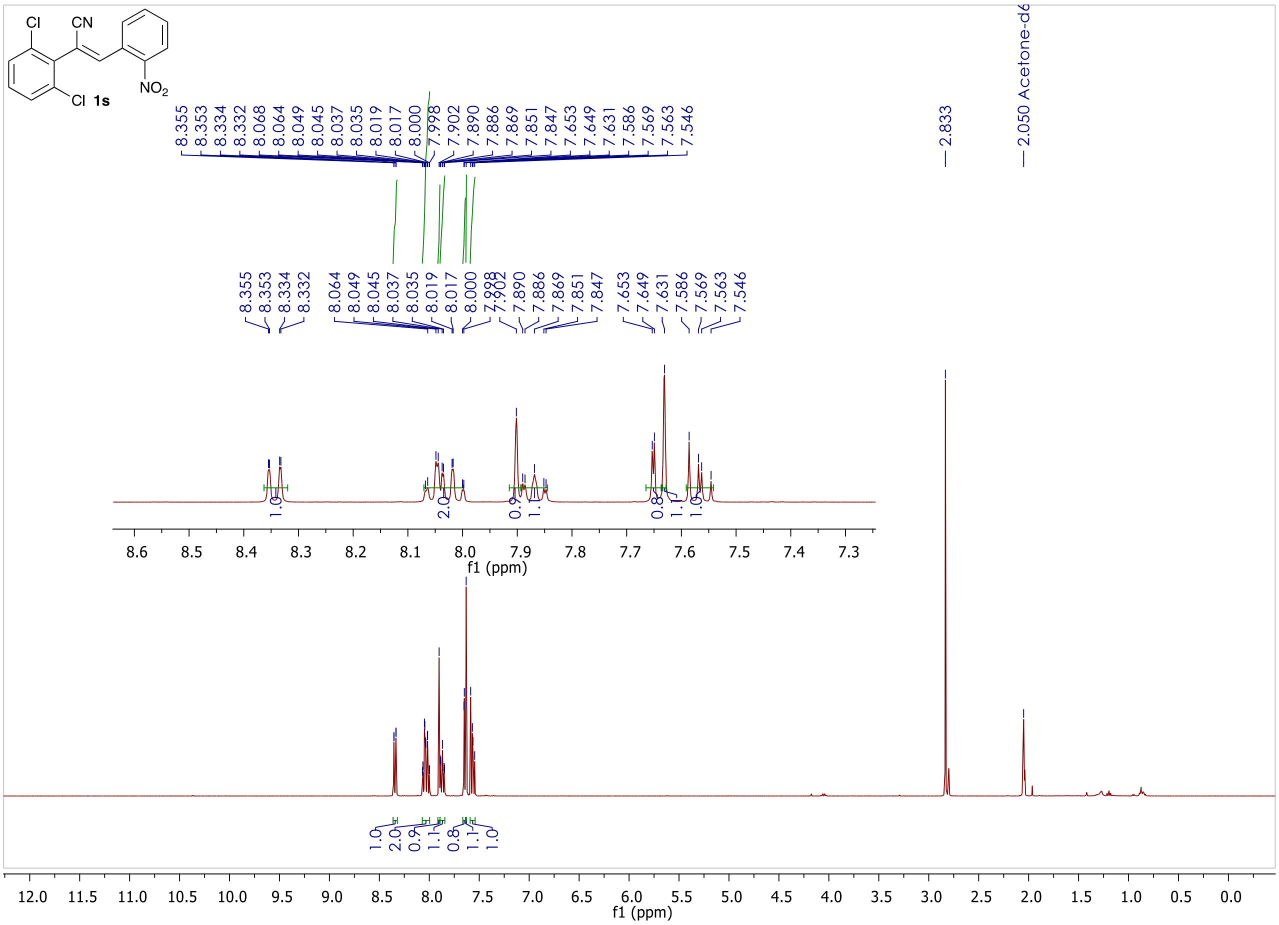

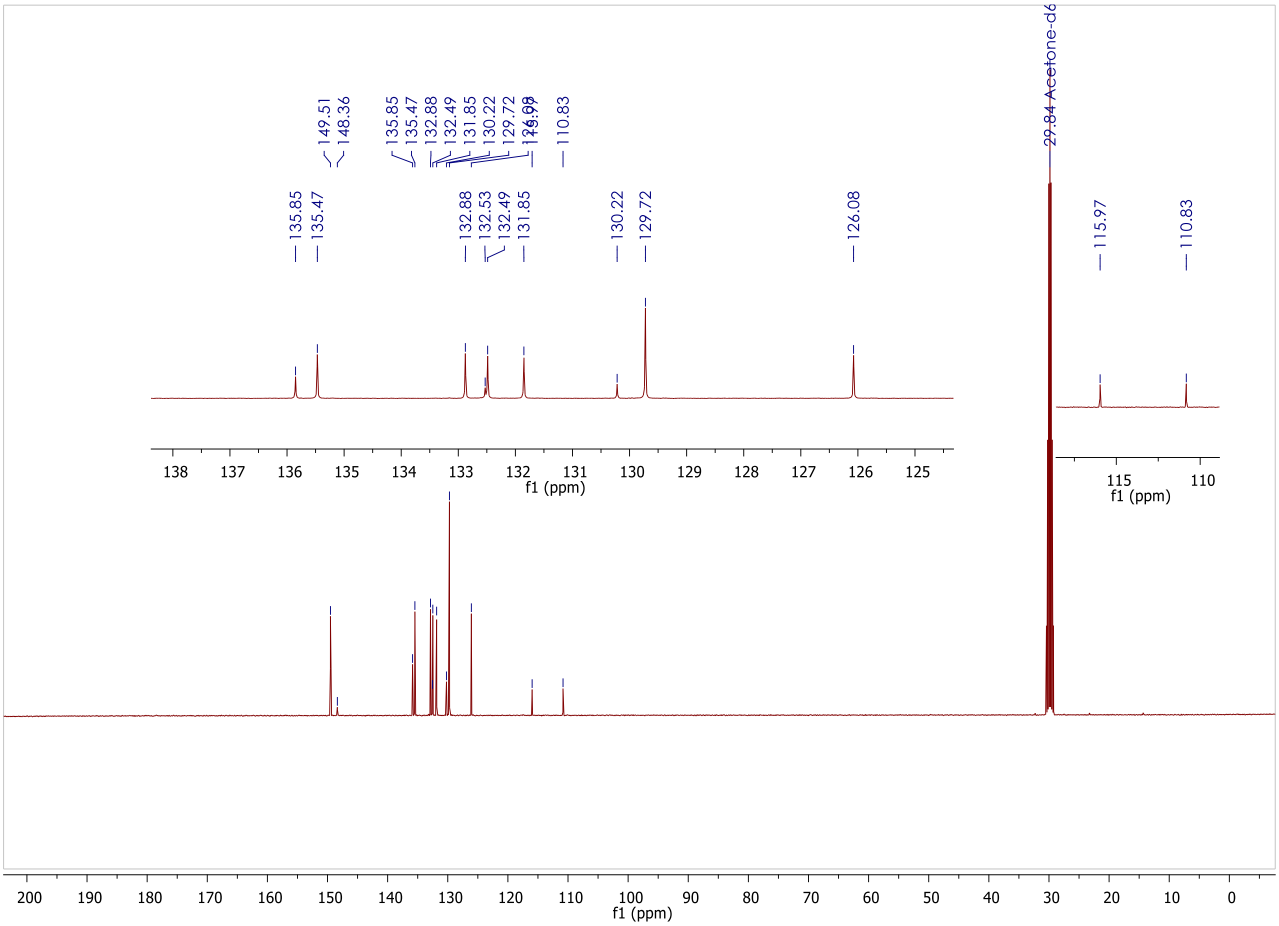

(*Z*)-2-(2,6-dichlorophenyl)-3-(3-nitrophenyl)acrylonitrile (**1t**)

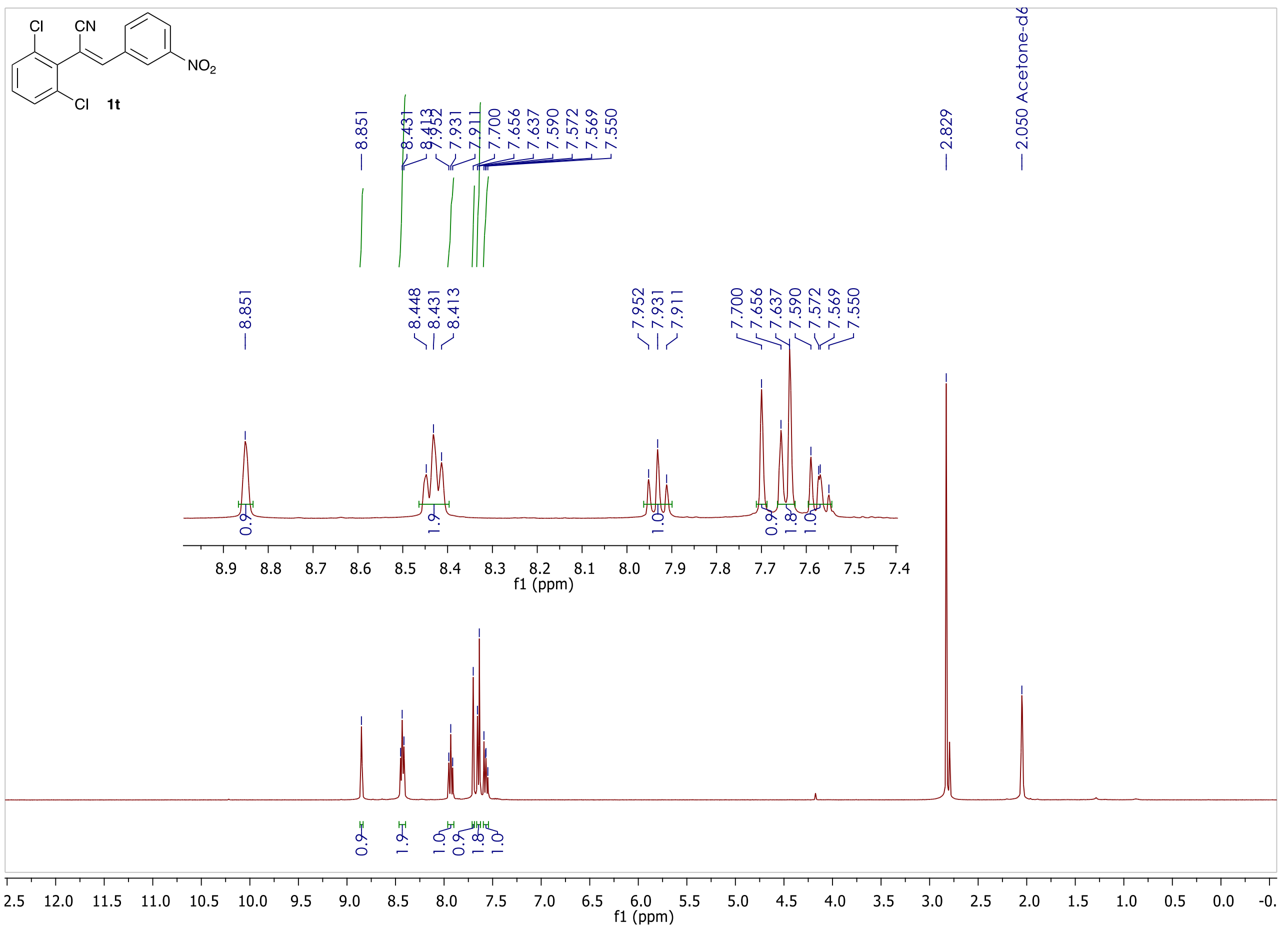

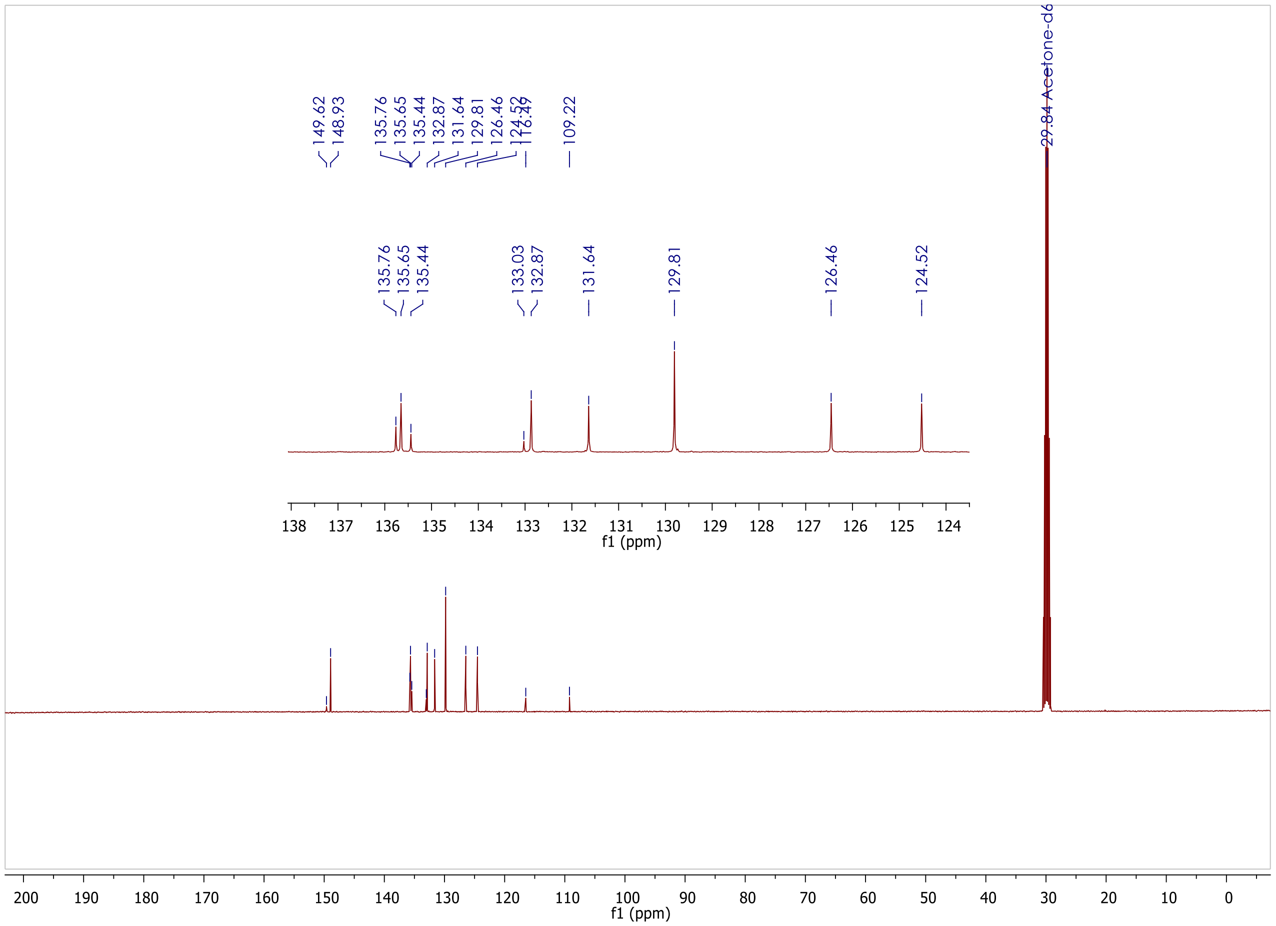

(*Z*)-2-(2,6-dichloro-3-nitrophenyl)-3-(2-nitrophenyl)acrylonitrile (**1u**)

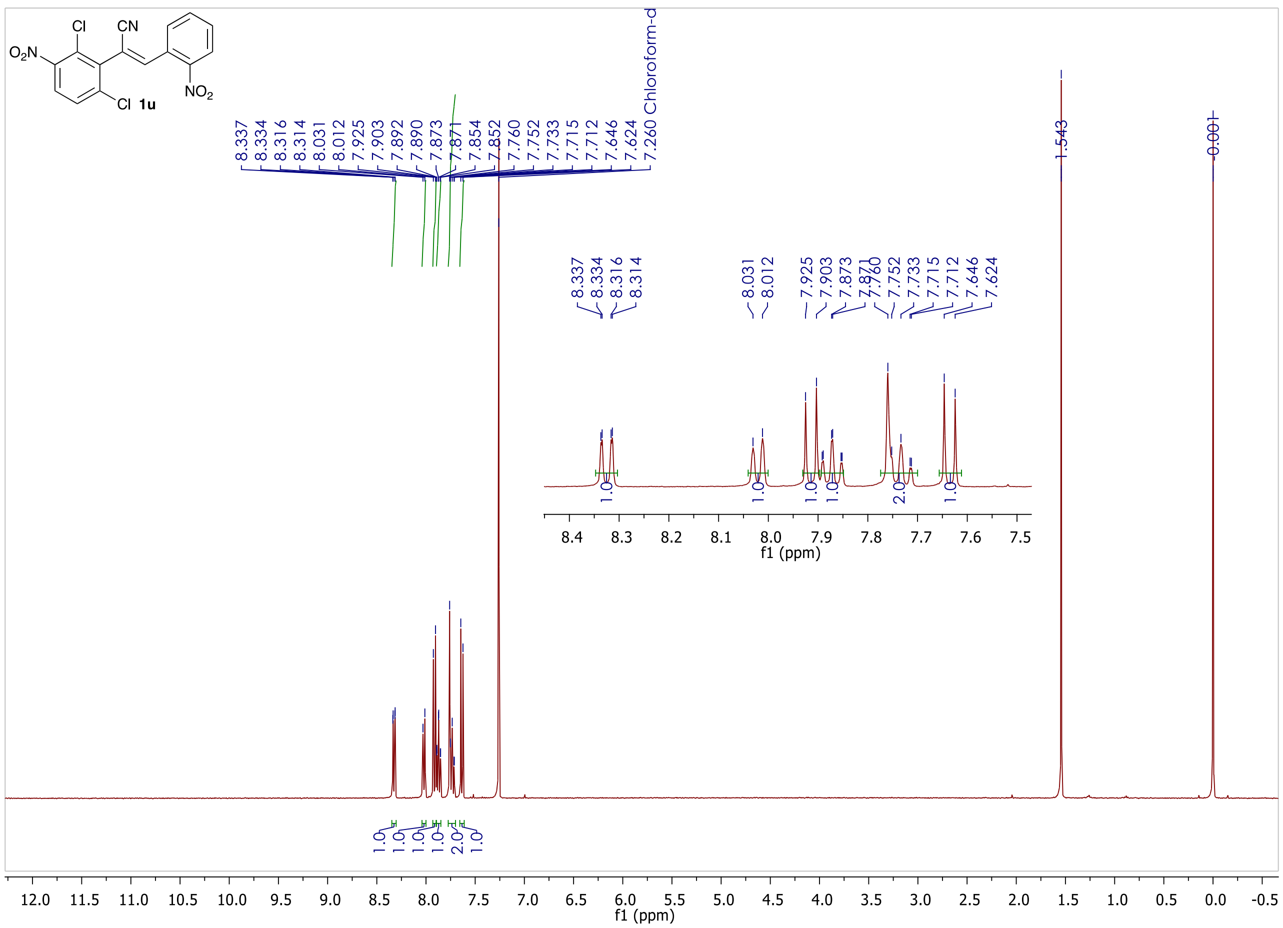

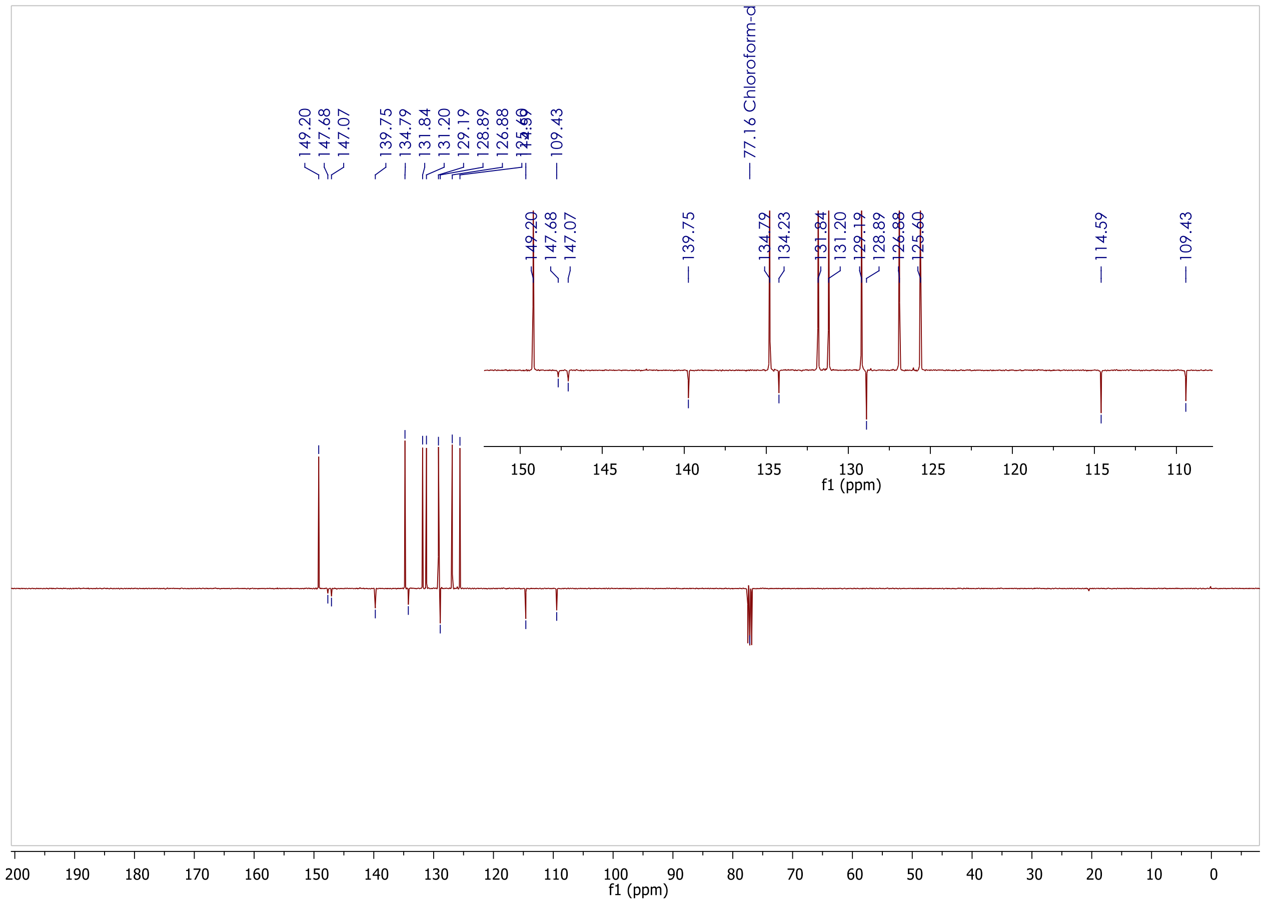

(*Z*)-2-(2,6-dichloro-3-nitrophenyl)-3-(3-nitrophenyl)acrylonitrile (**1v**)

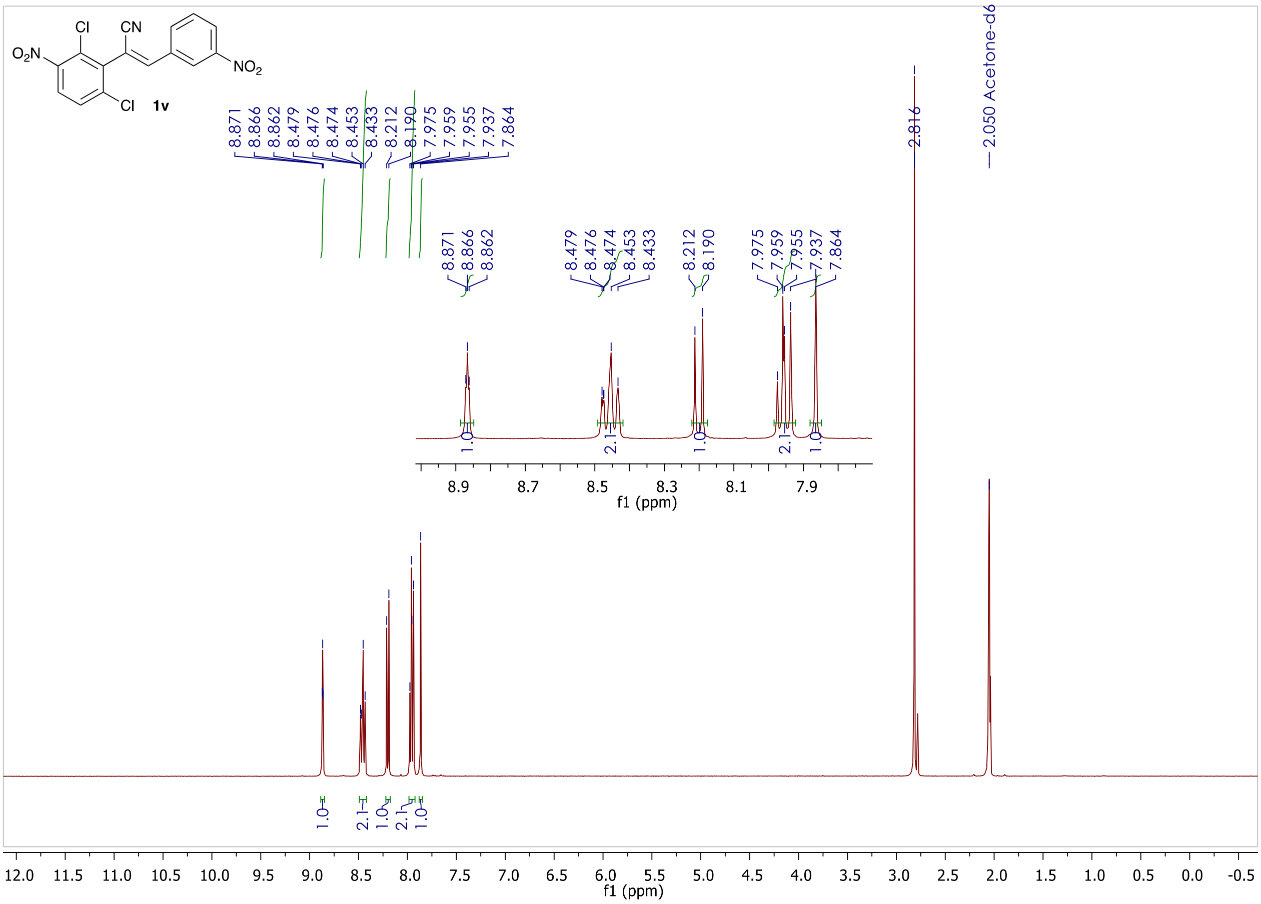

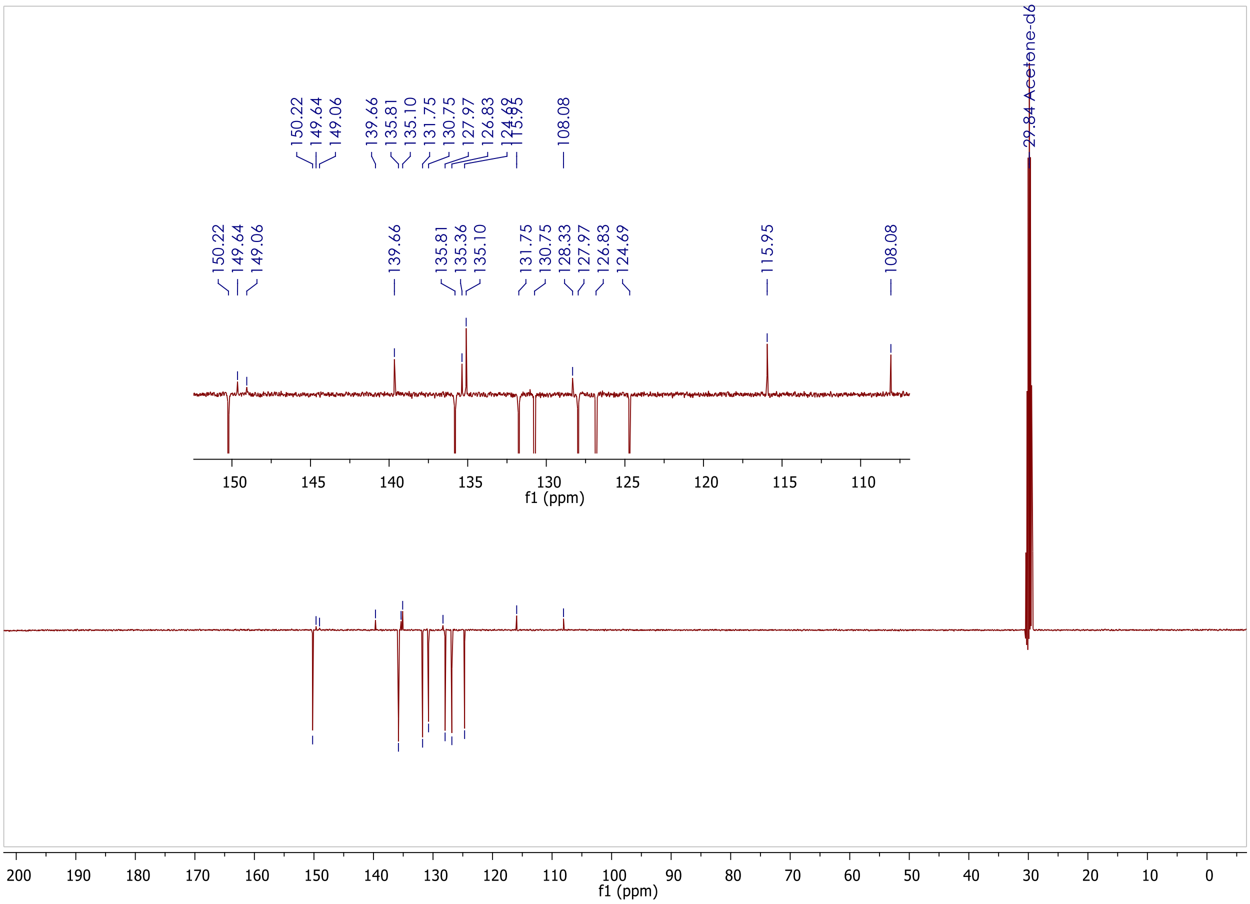

(*Z*)-2-(2,6-dichloro-3-nitrophenyl)-3-(4-nitrophenyl)acrylonitrile (**1w**)

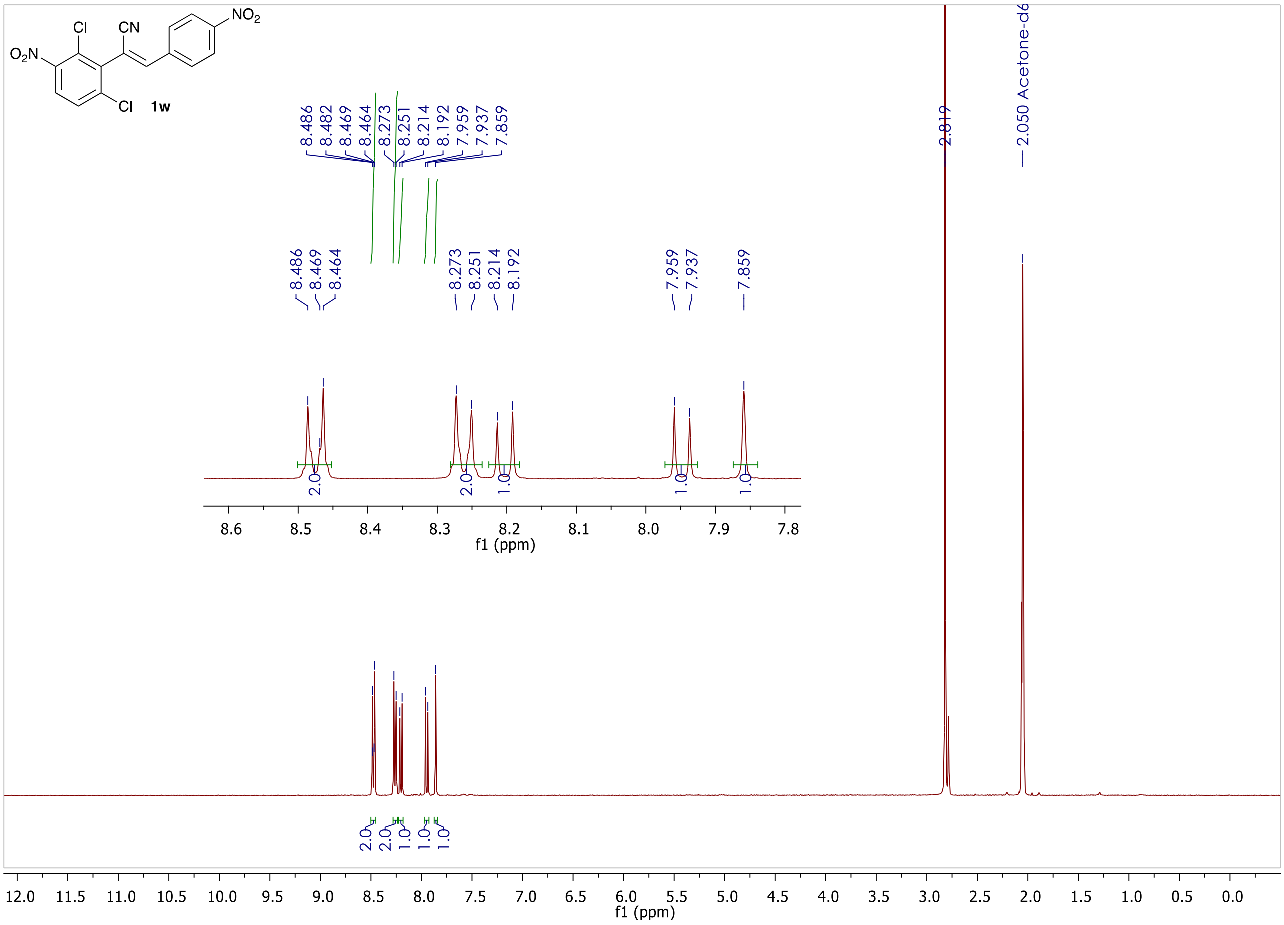

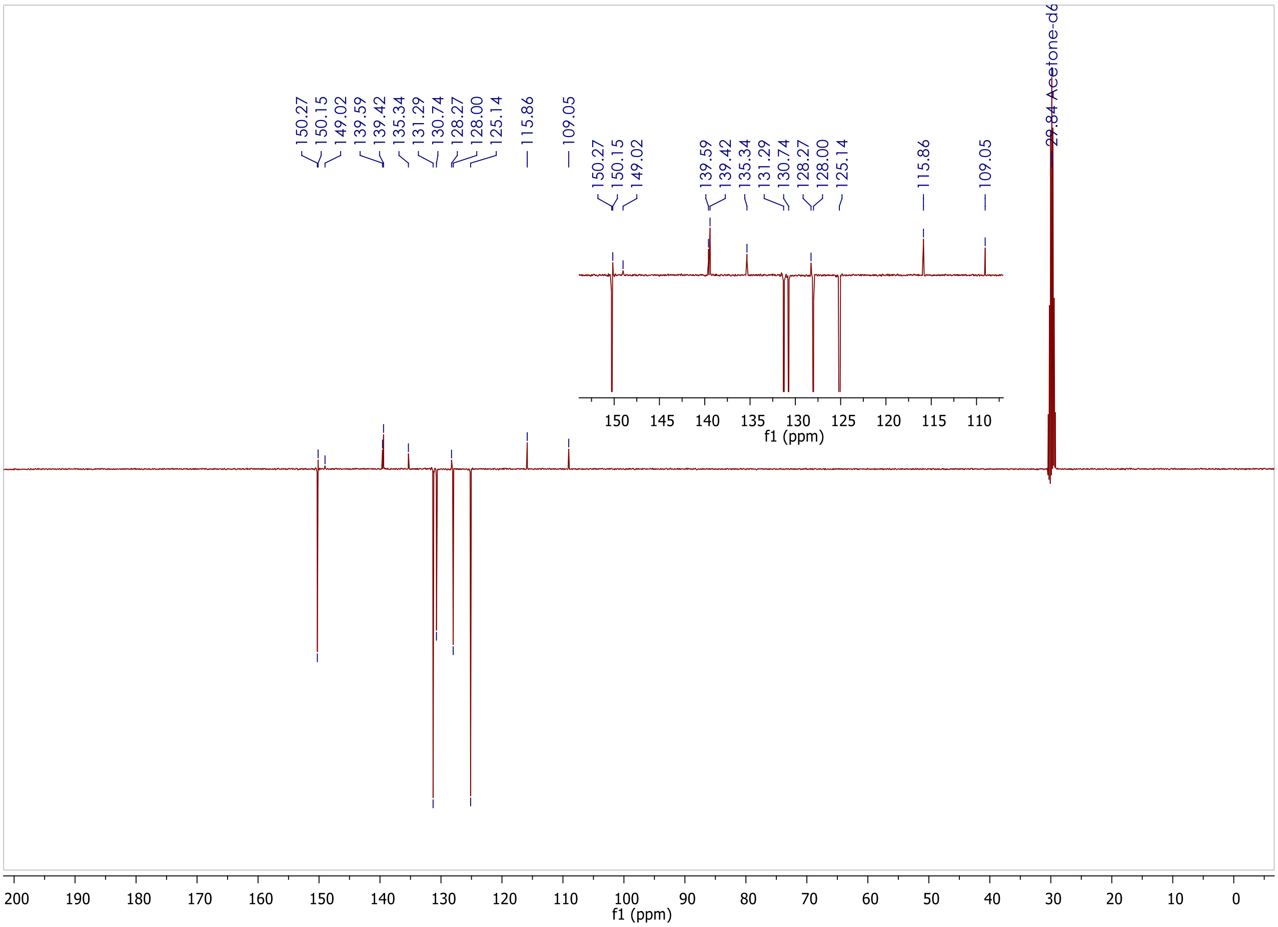

(*Z*)-2-(3-amino-2,6-dichlorophenyl)-3-(2-aminophenyl)acrylonitrile (**1x**)

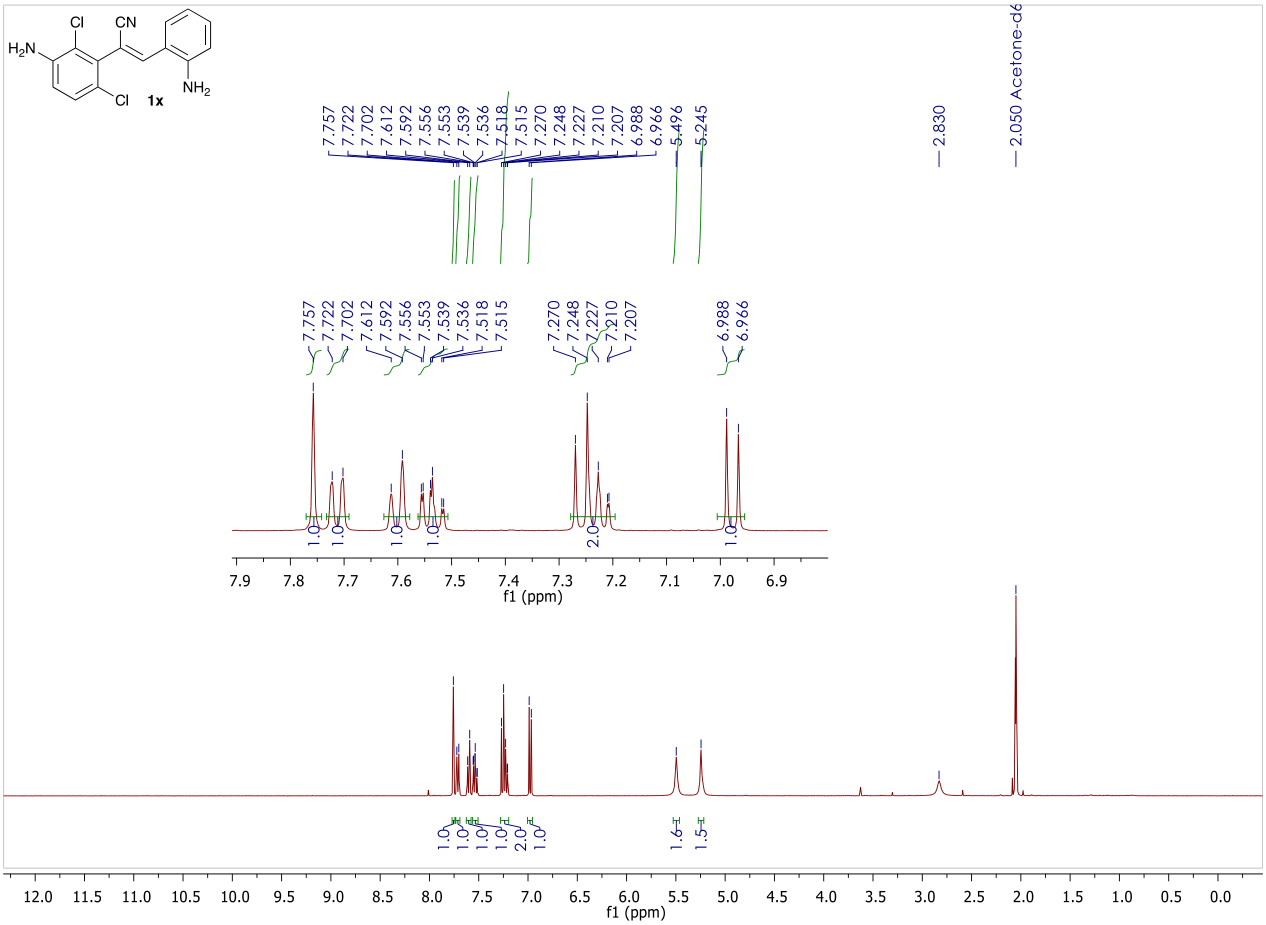

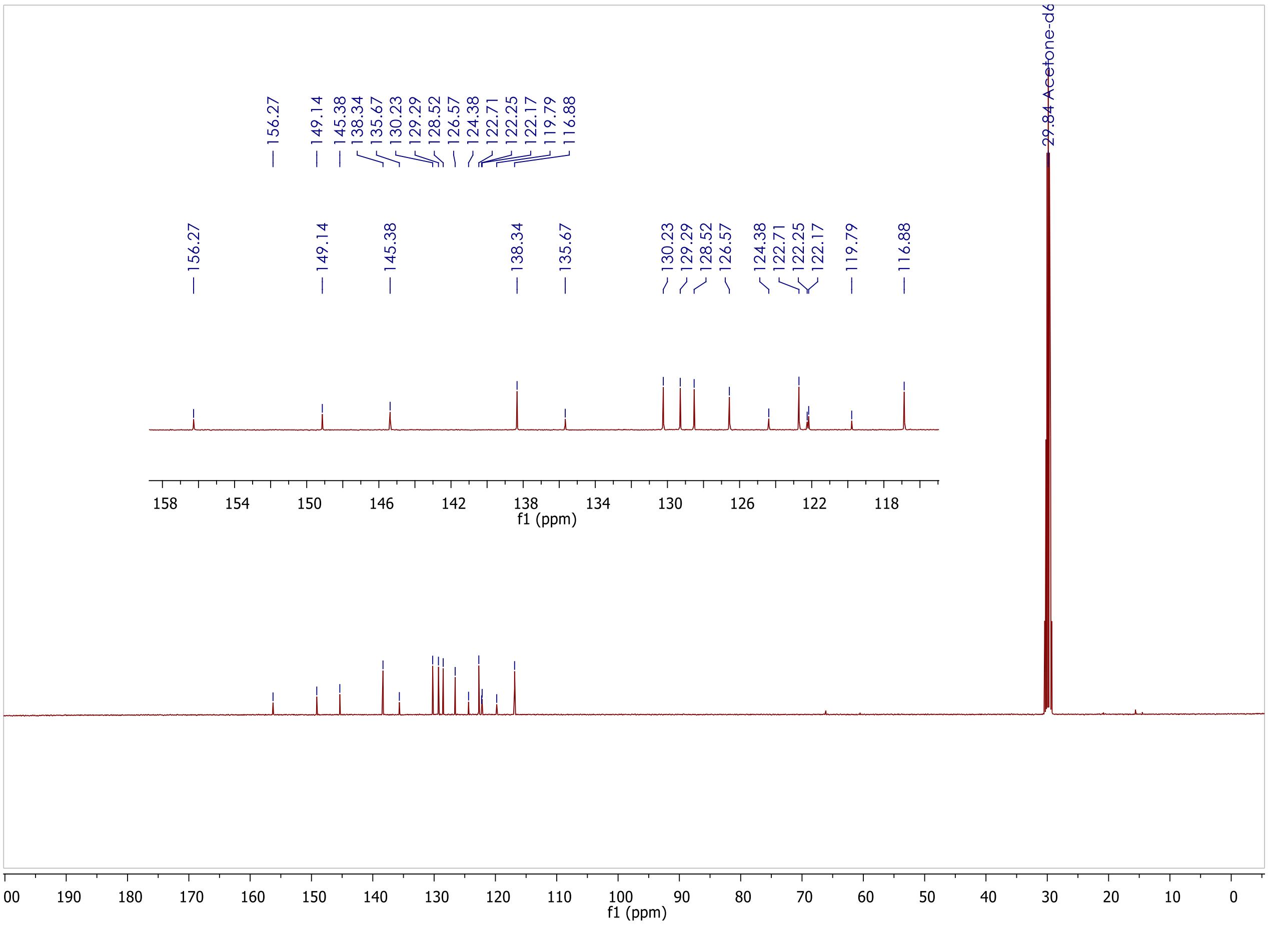

(*Z*)-2-(3-amino-2,6-dichlorophenyl)-3-(3-aminophenyl)acrylonitrile (**1y**)

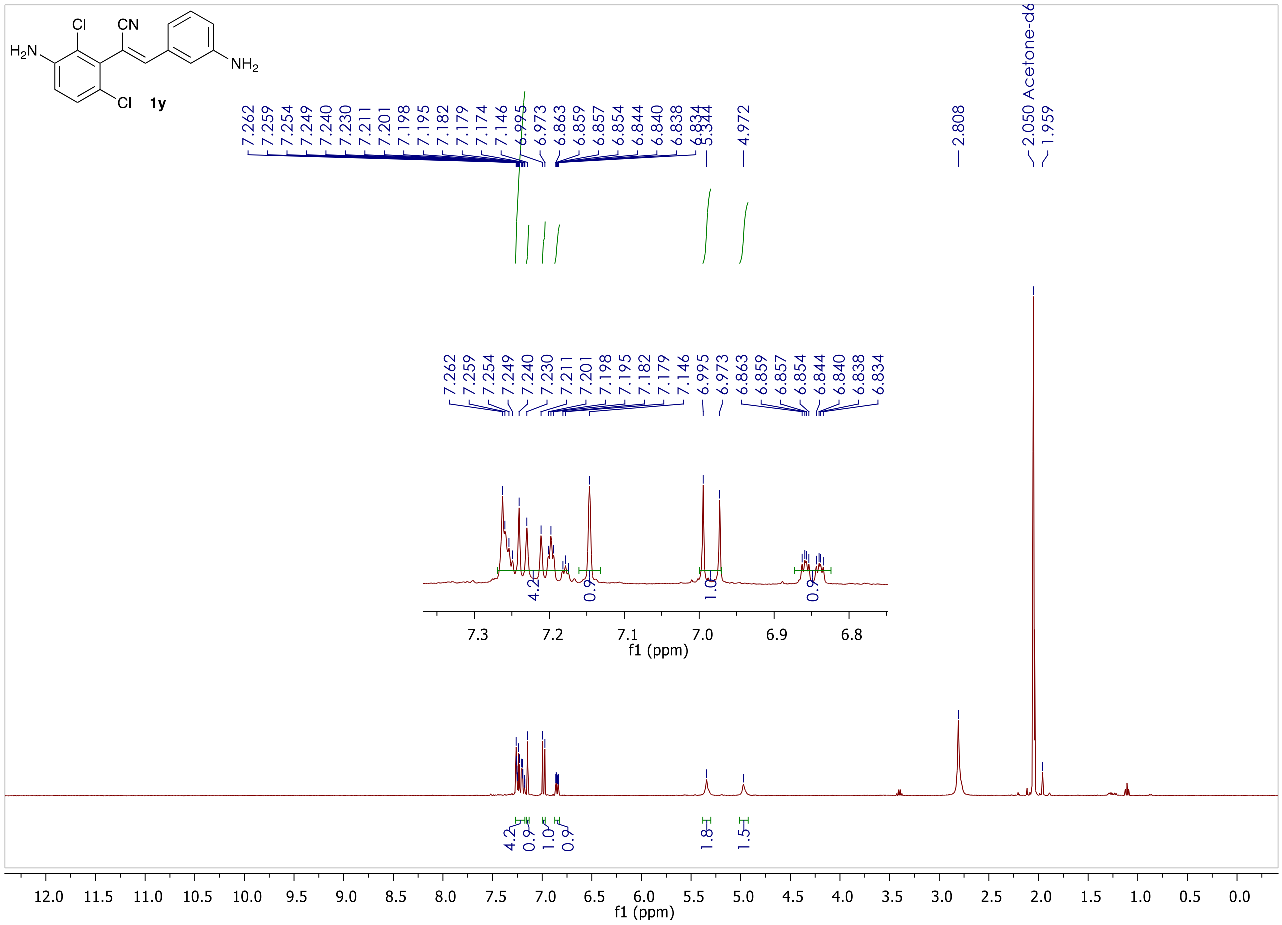

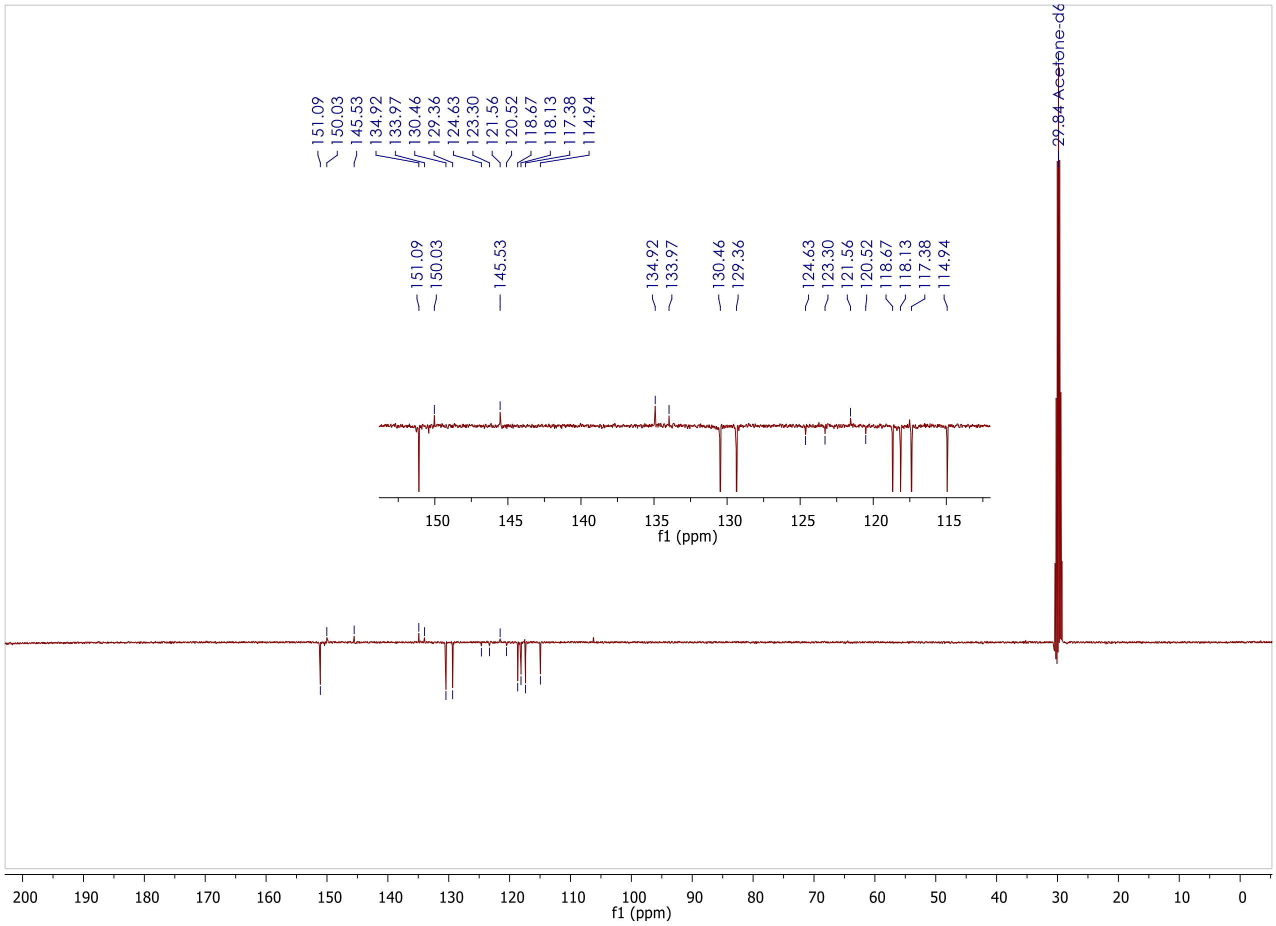

(*Z*)-2-(3,4-dichlorophenyl)-3-(4-(oxiran-2-ylmethoxy)phenyl)acrylonitrile (**6**)

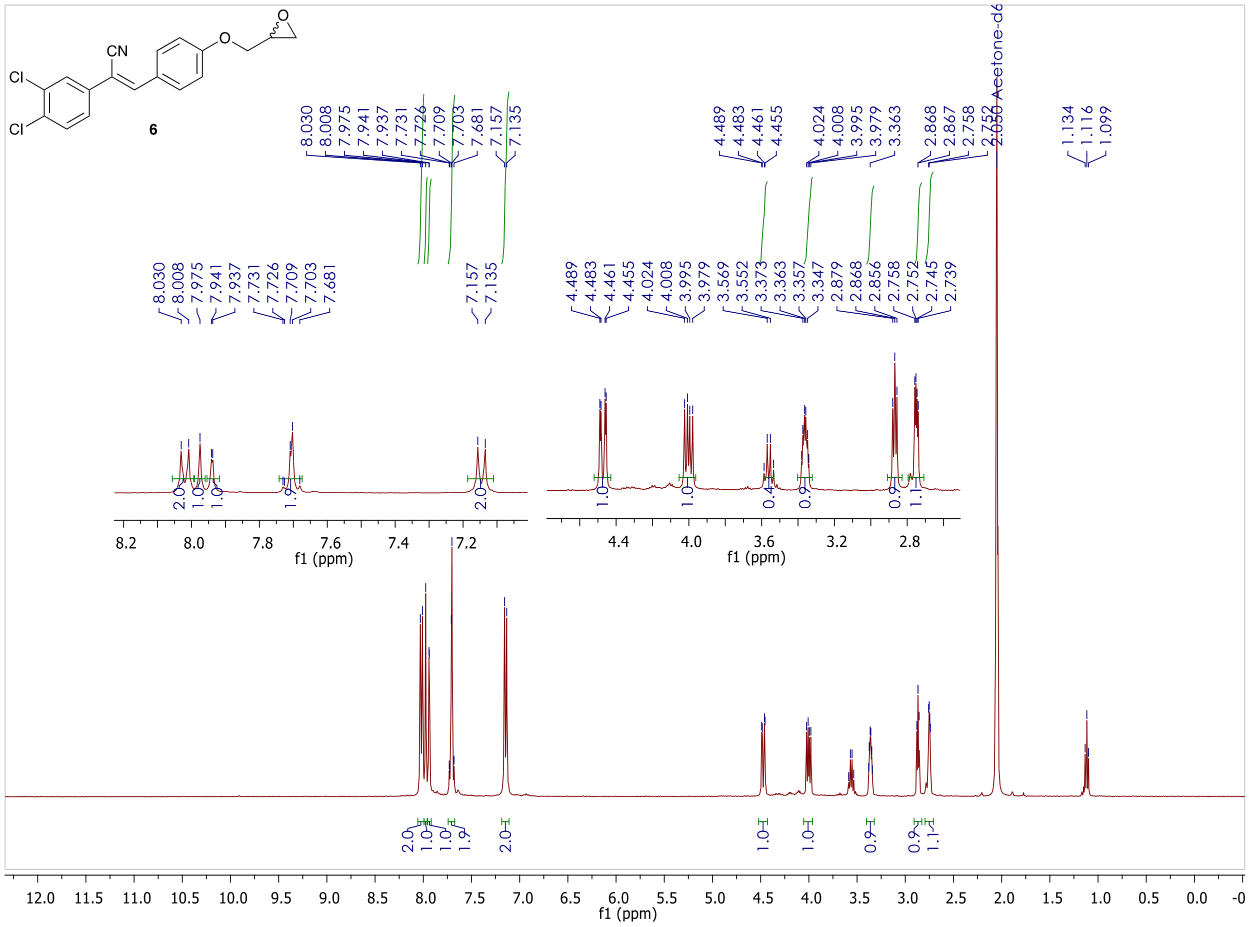

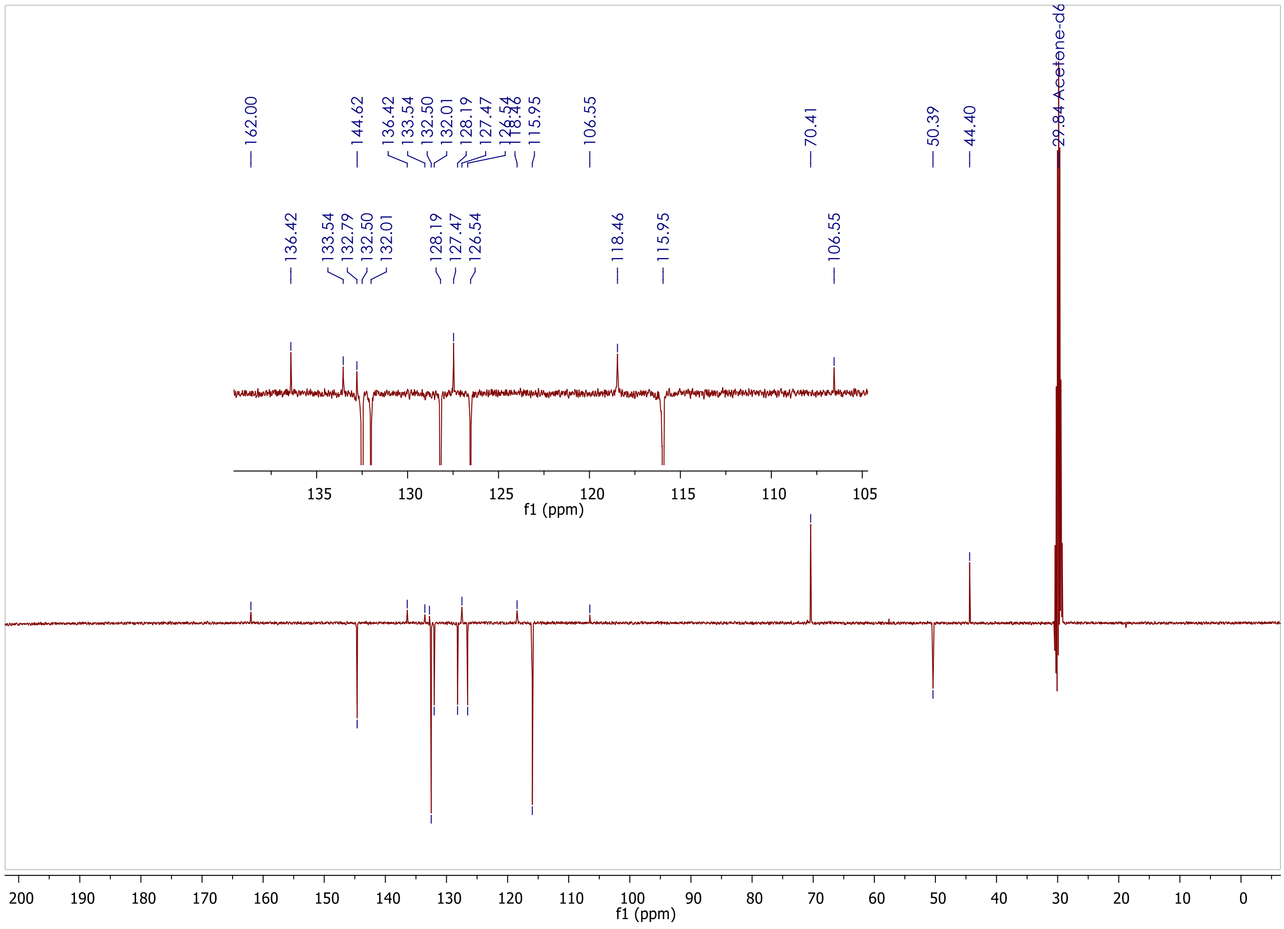

(*Z*)-2-(3,4-dichlorophenyl)-3-(4-(2-hydroxy-3-(phenylamino)propoxy)phenyl)acrylonitrile (**8a**)

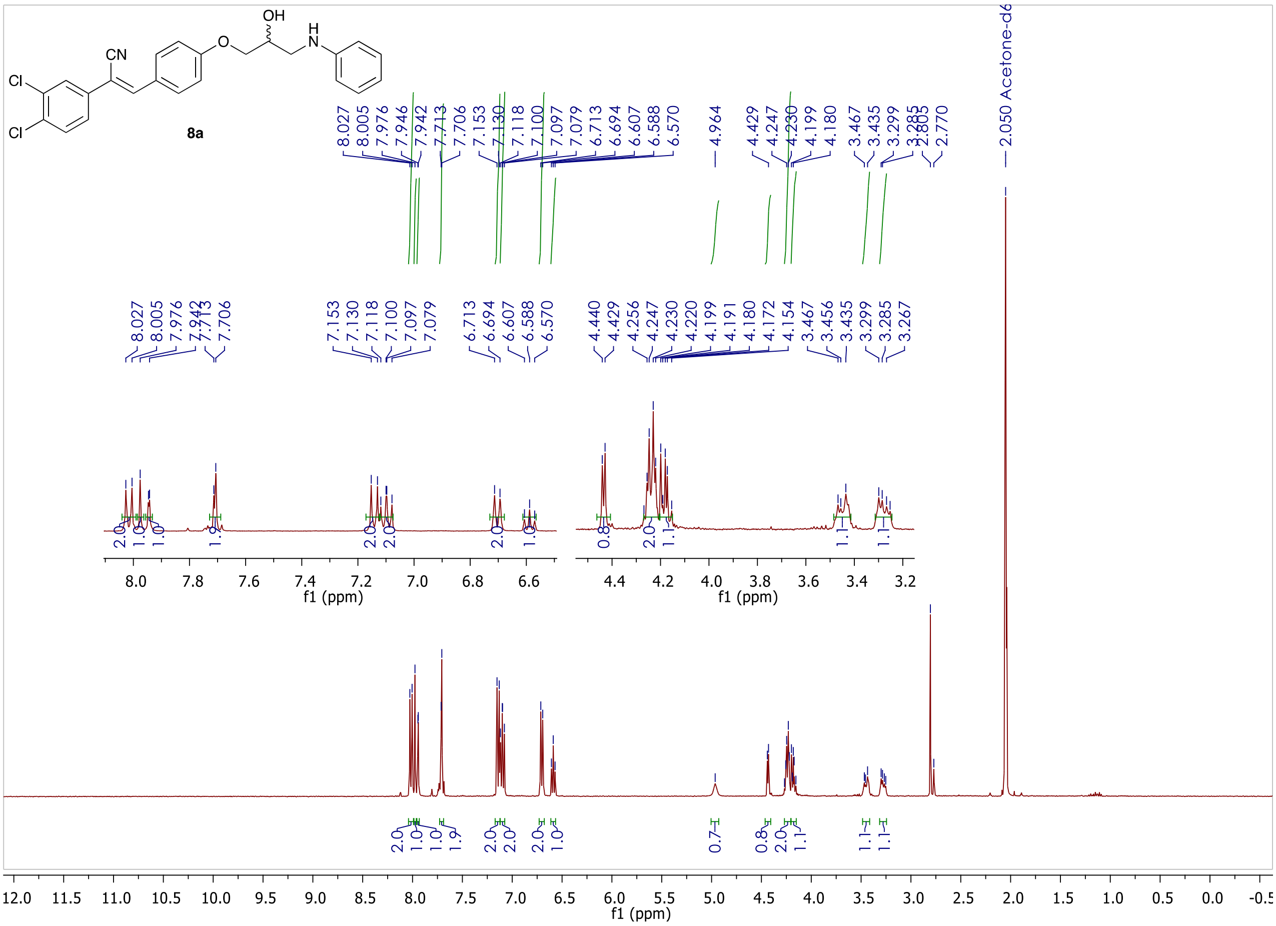

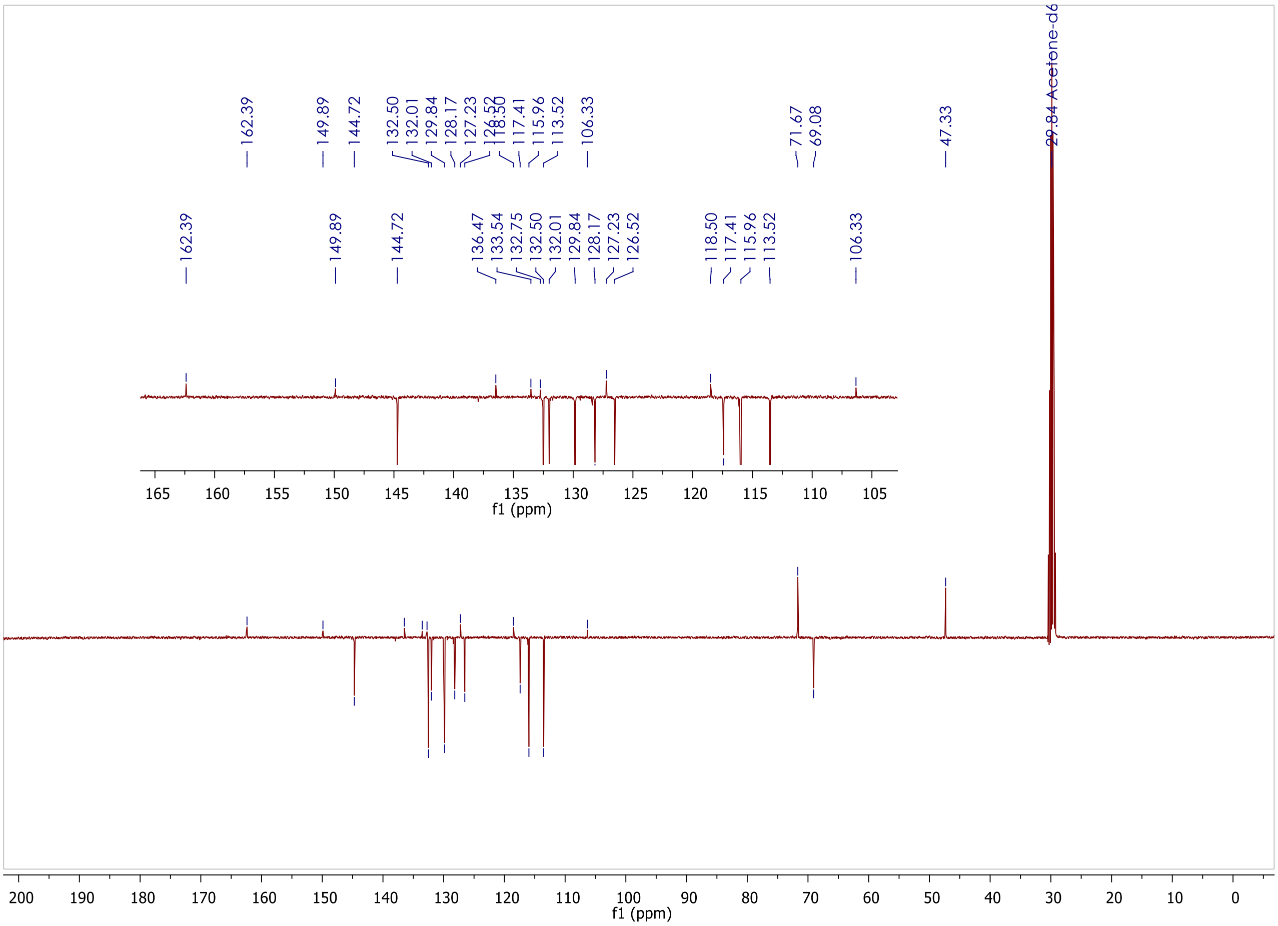

(*Z*)-3-(4-(3-((4-chlorophenyl)amino)-2-hydroxypropoxy)phenyl)-2-(3,4-dichlorophenyl)acrylonitrile (**8b**)

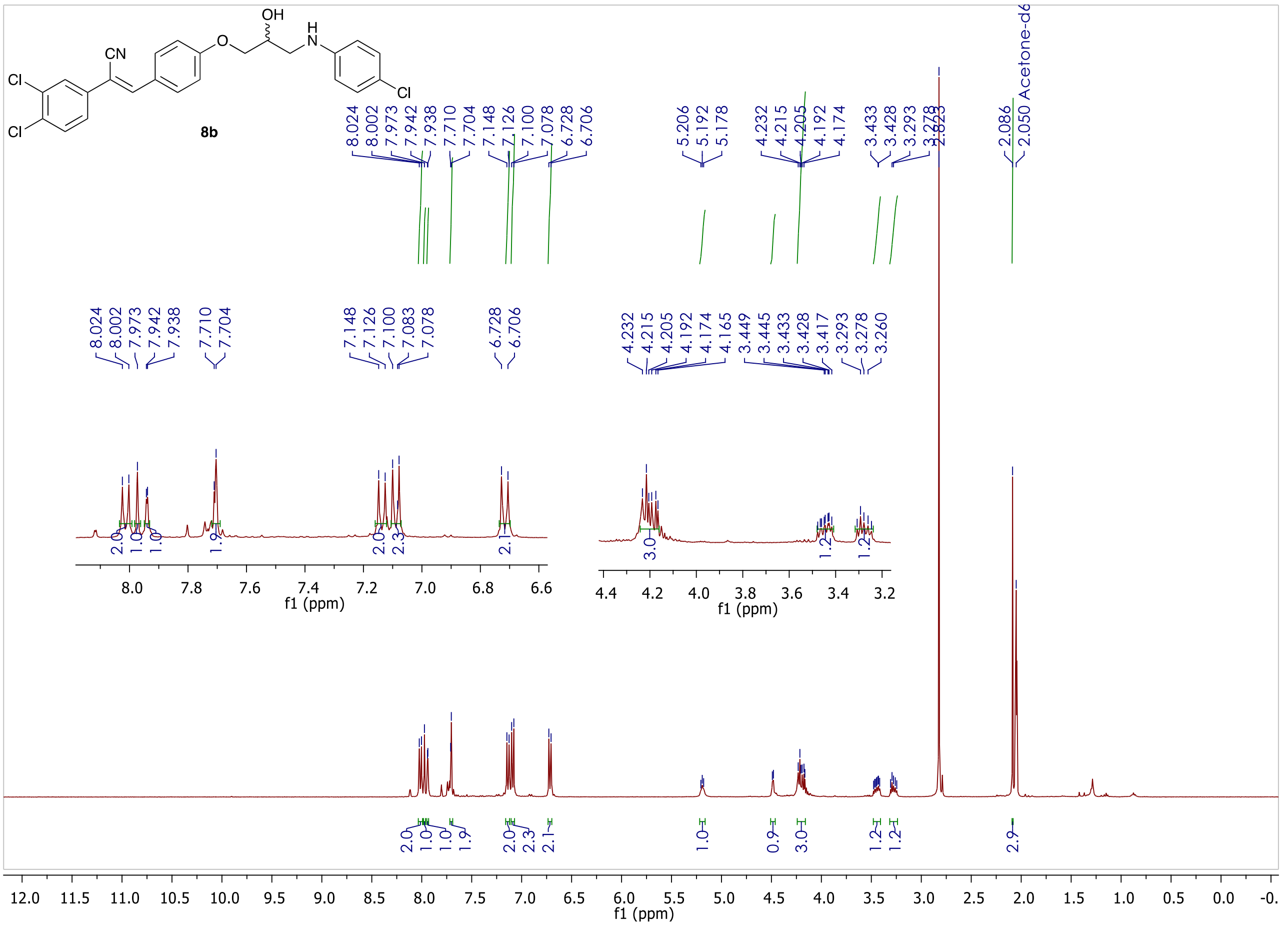

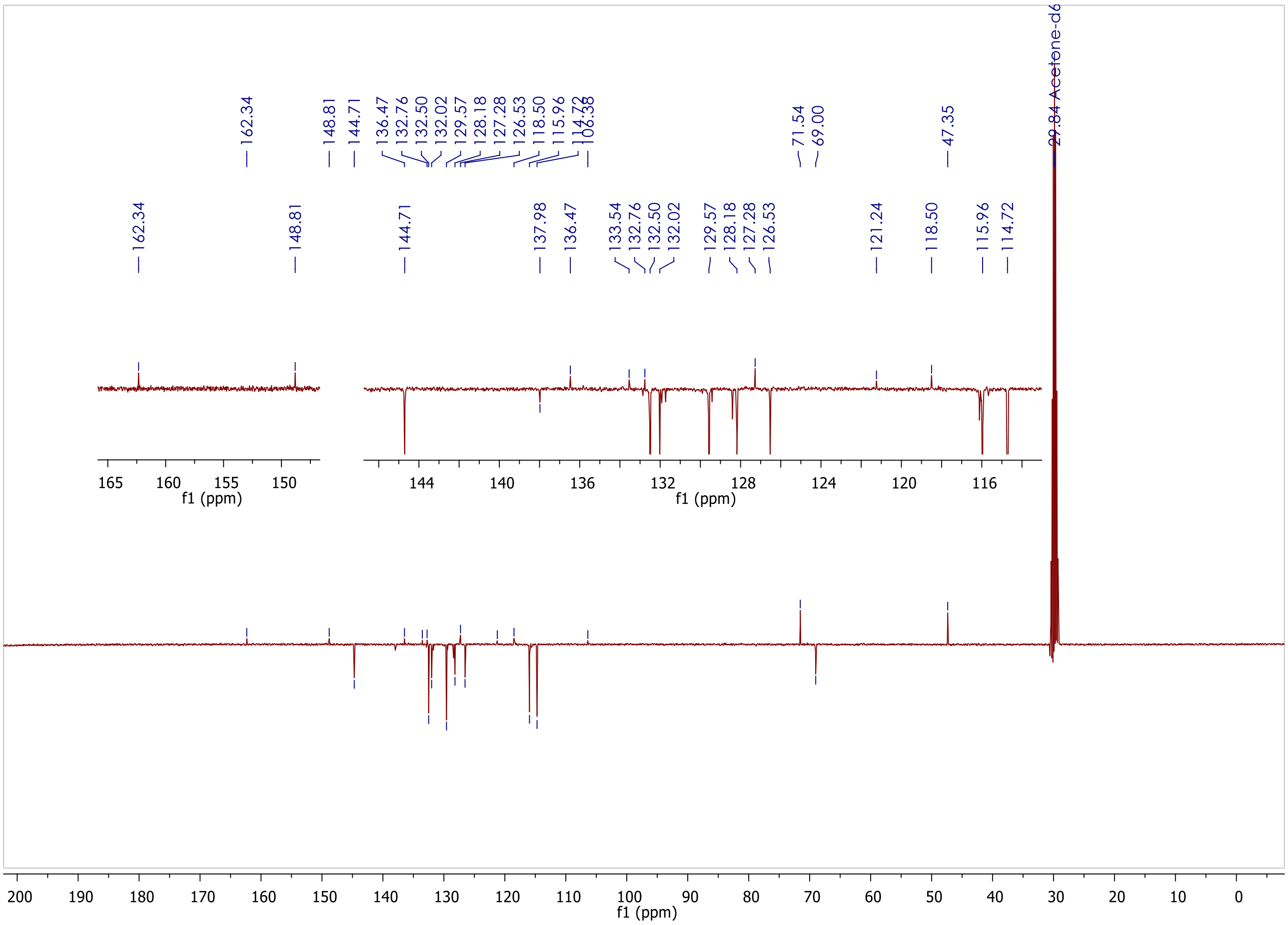

(*Z*)-3-(4-(3-((4-bromophenyl)amino)-2-hydroxypropoxy)phenyl)-2-(3,4-dichlorophenyl)acrylonitrile (**8c**)

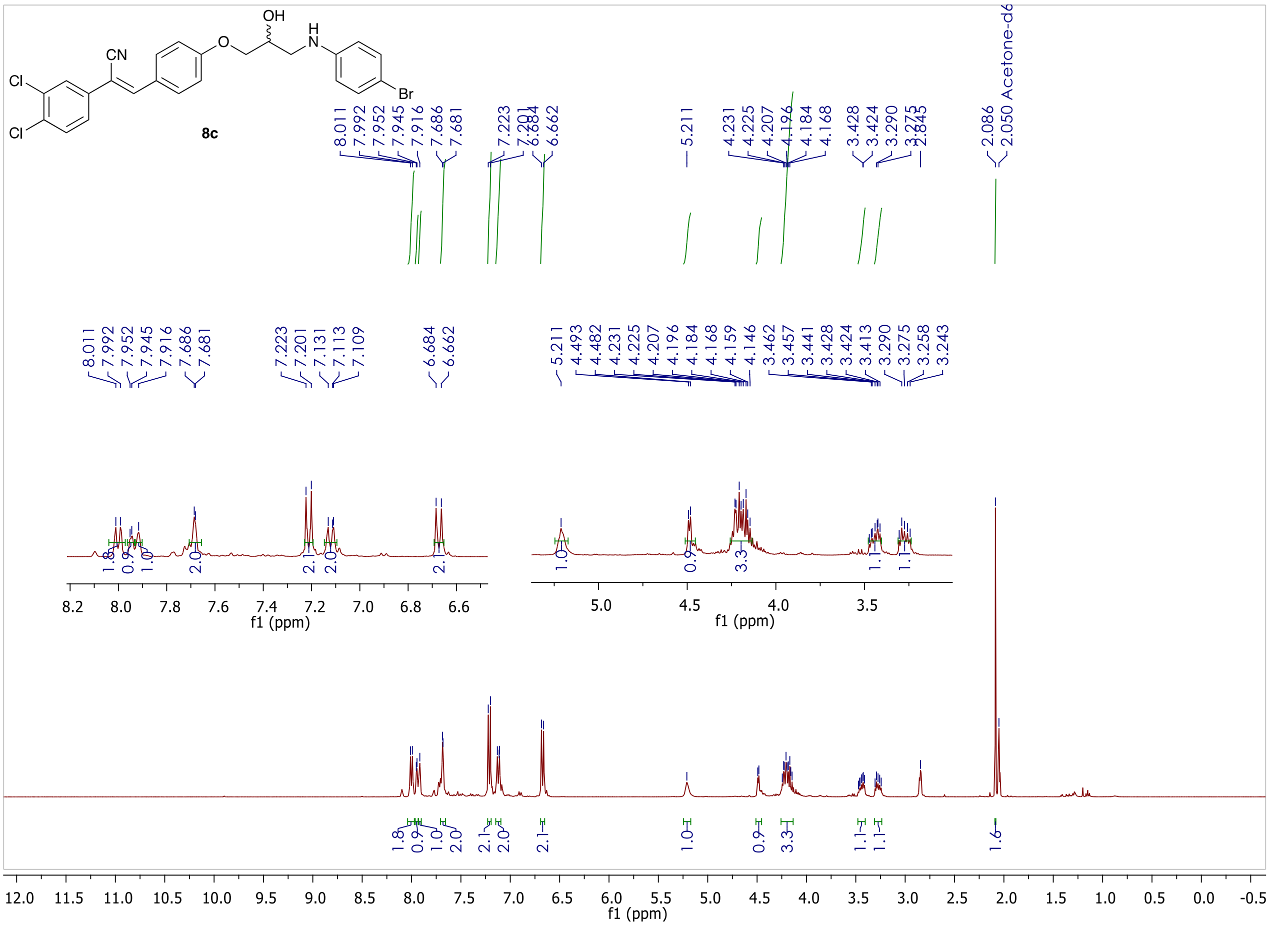

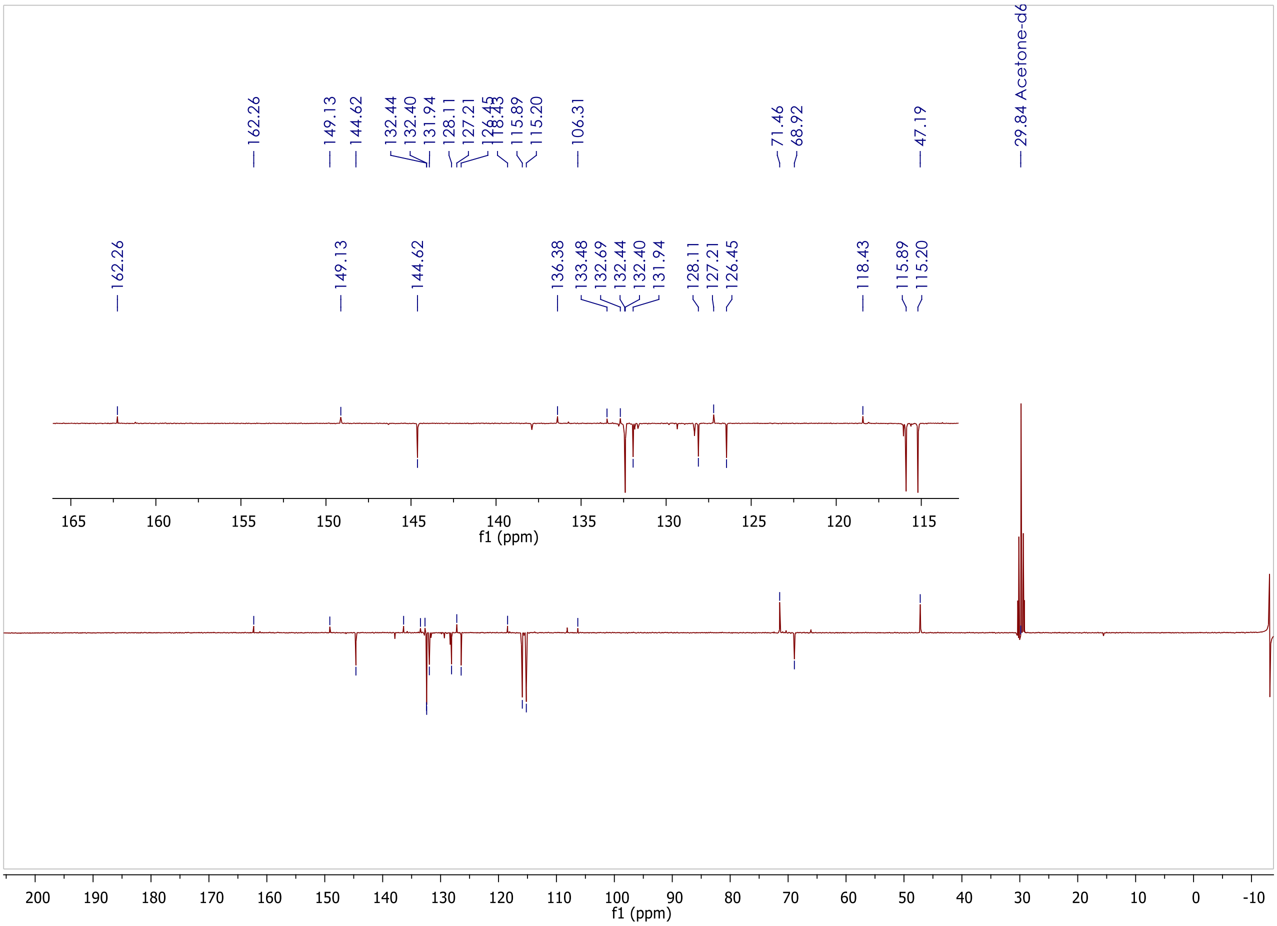

(*Z*)-2-(3,4-dichlorophenyl)-3-(4-(2-hydroxy-3-((4-methoxyphenyl)amino)propoxy)phenyl)acrylonitrile (**8d**)

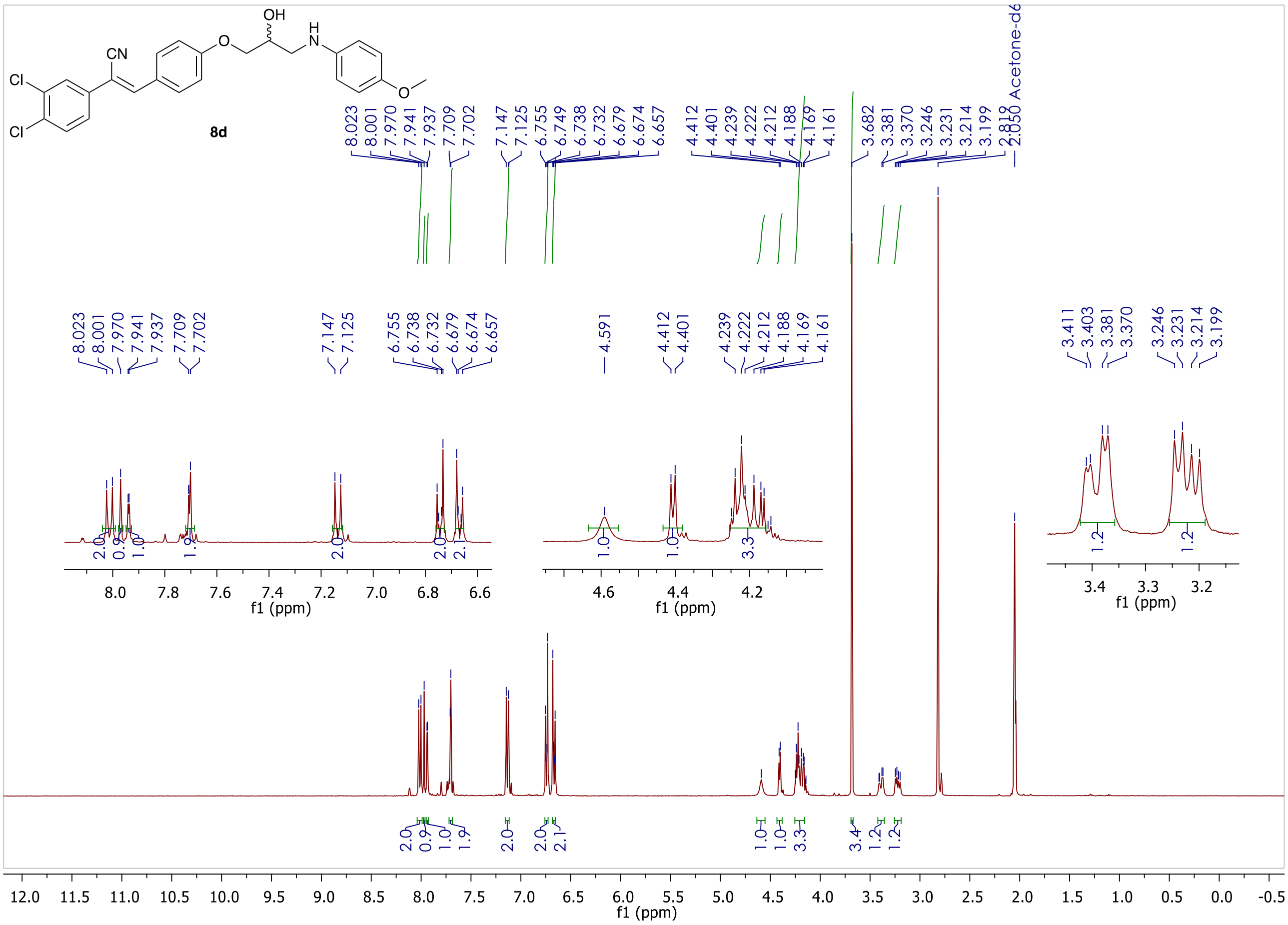

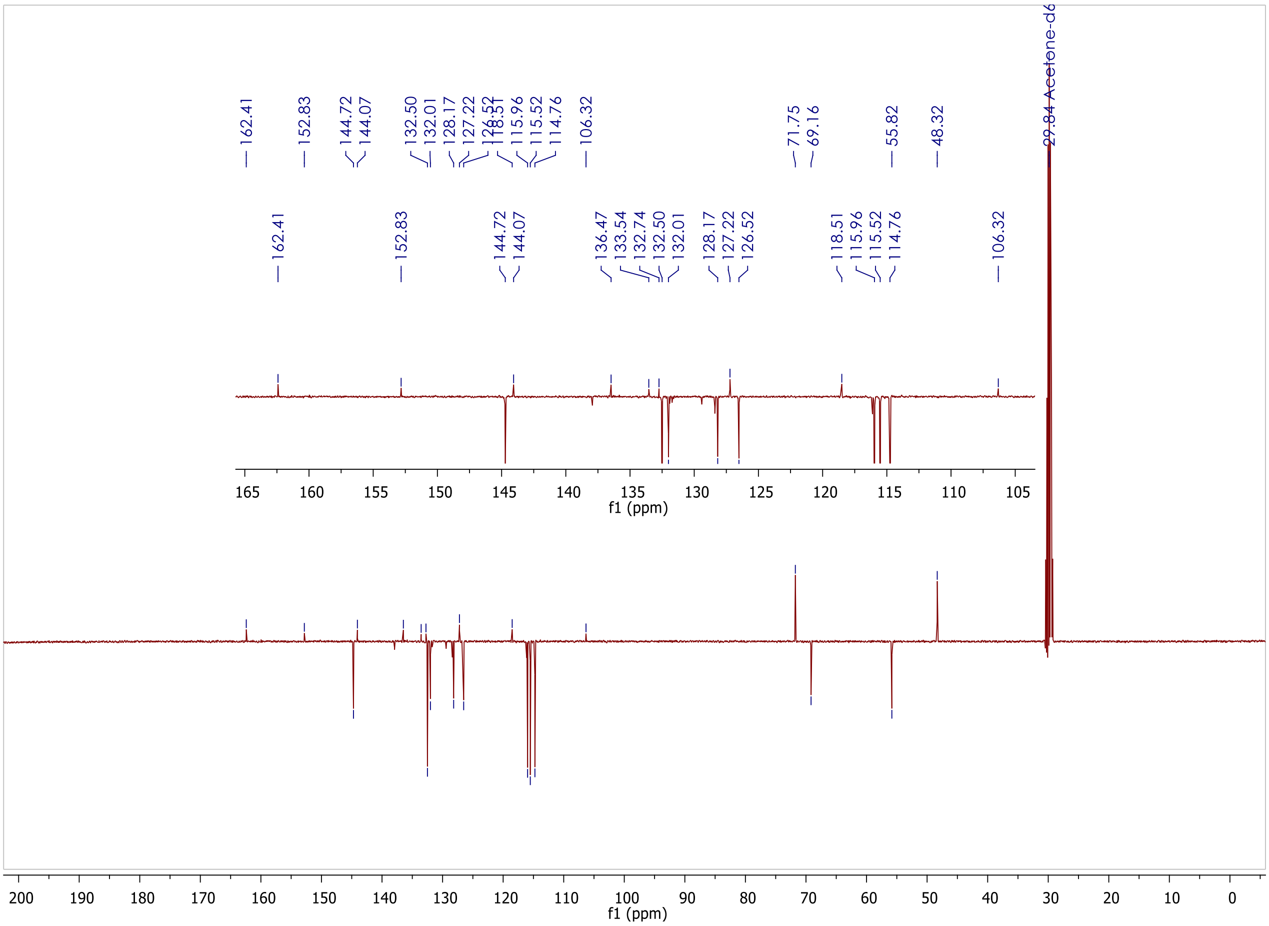

(*Z*)-3-(4-(3-(cyclohexylamino)-2-hydroxypropoxy)phenyl)-2-(3,4-dichlorophenyl)acrylonitrile (**8e**)

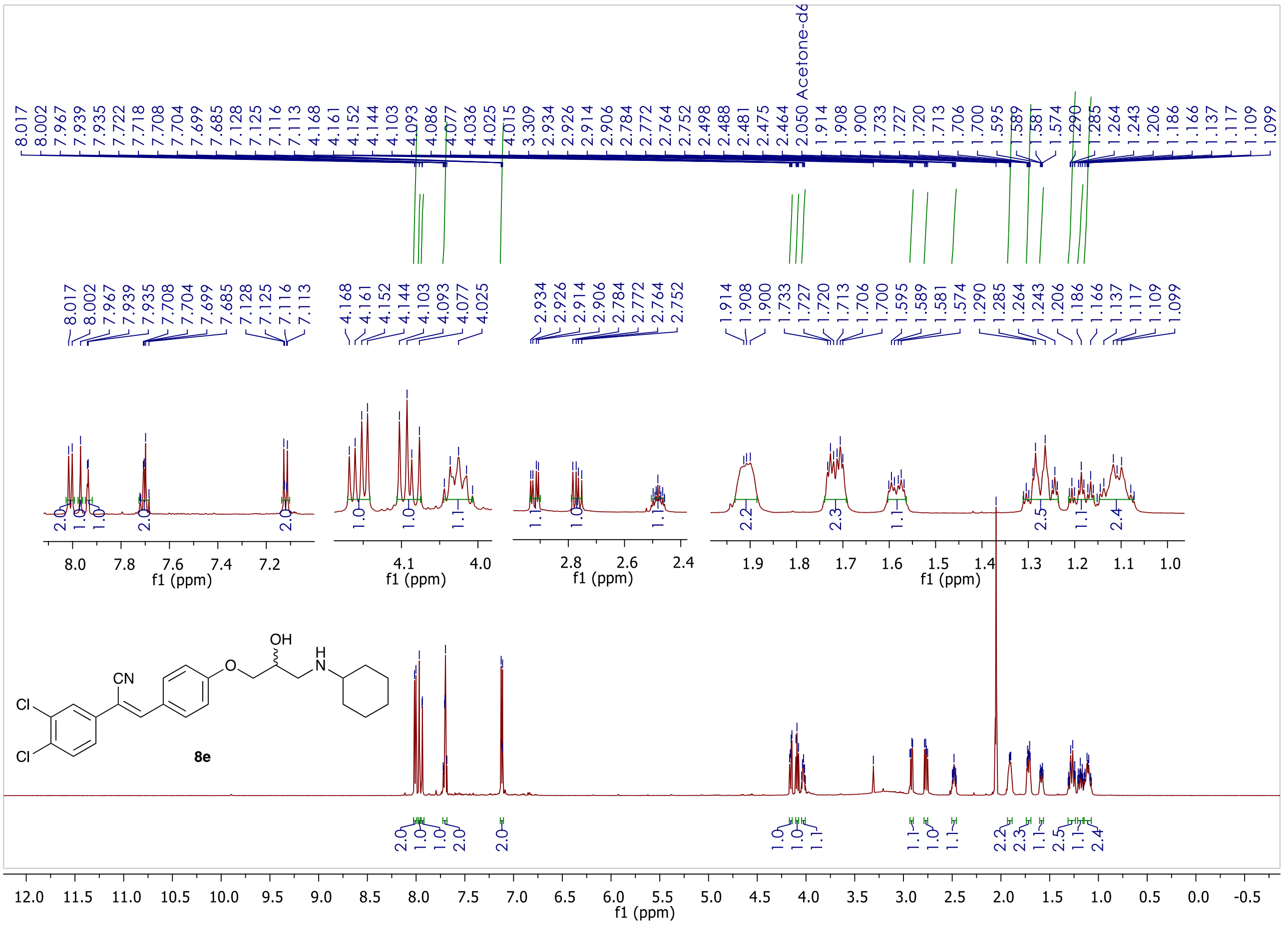

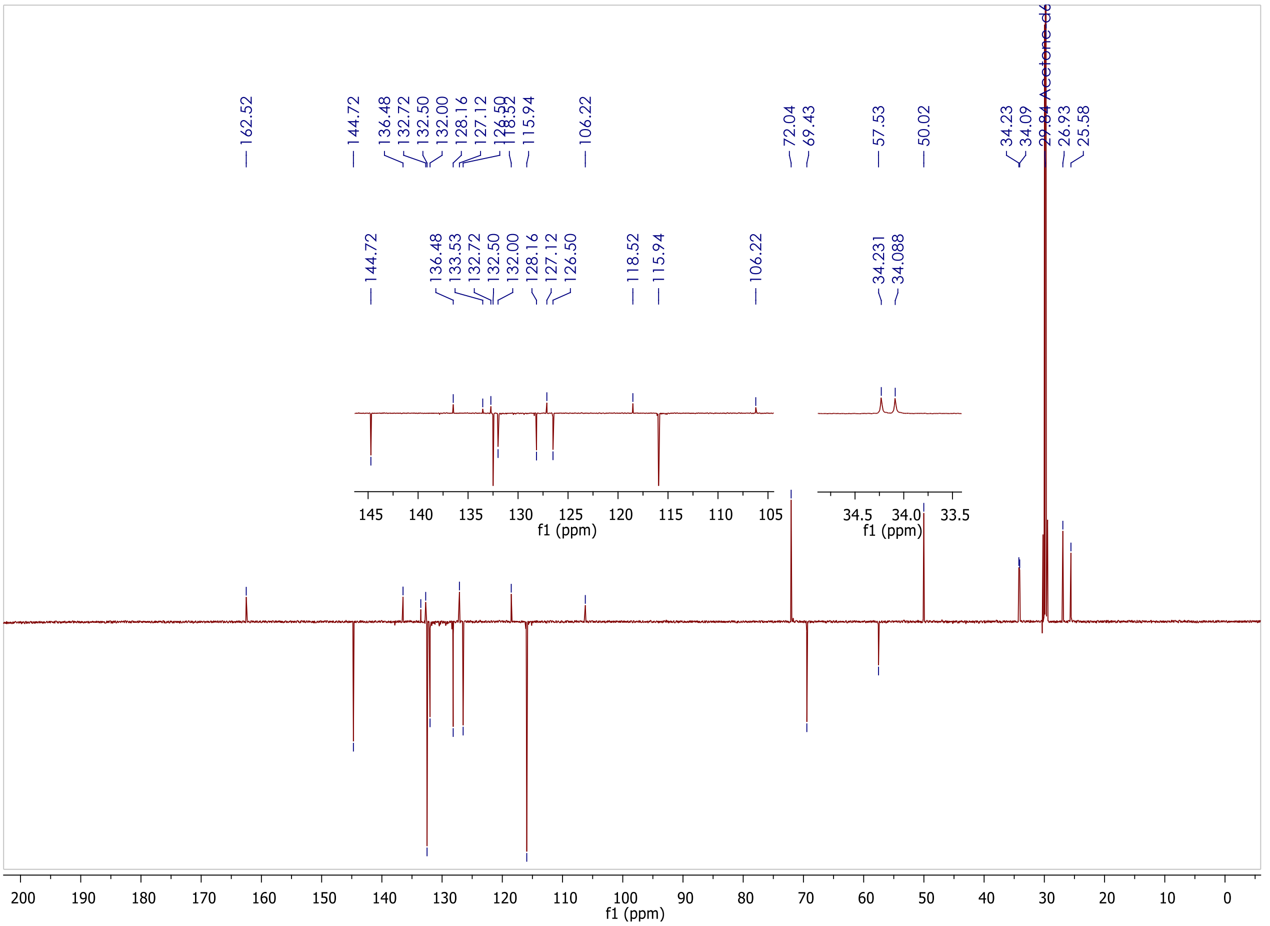

(*Z*)-3-(4-(3-(4-cyclohexylpiperazin-1-yl)-2-hydroxypropoxy)phenyl)-2-(3,4-dichlorophenyl)acrylonitrile (**8f**)

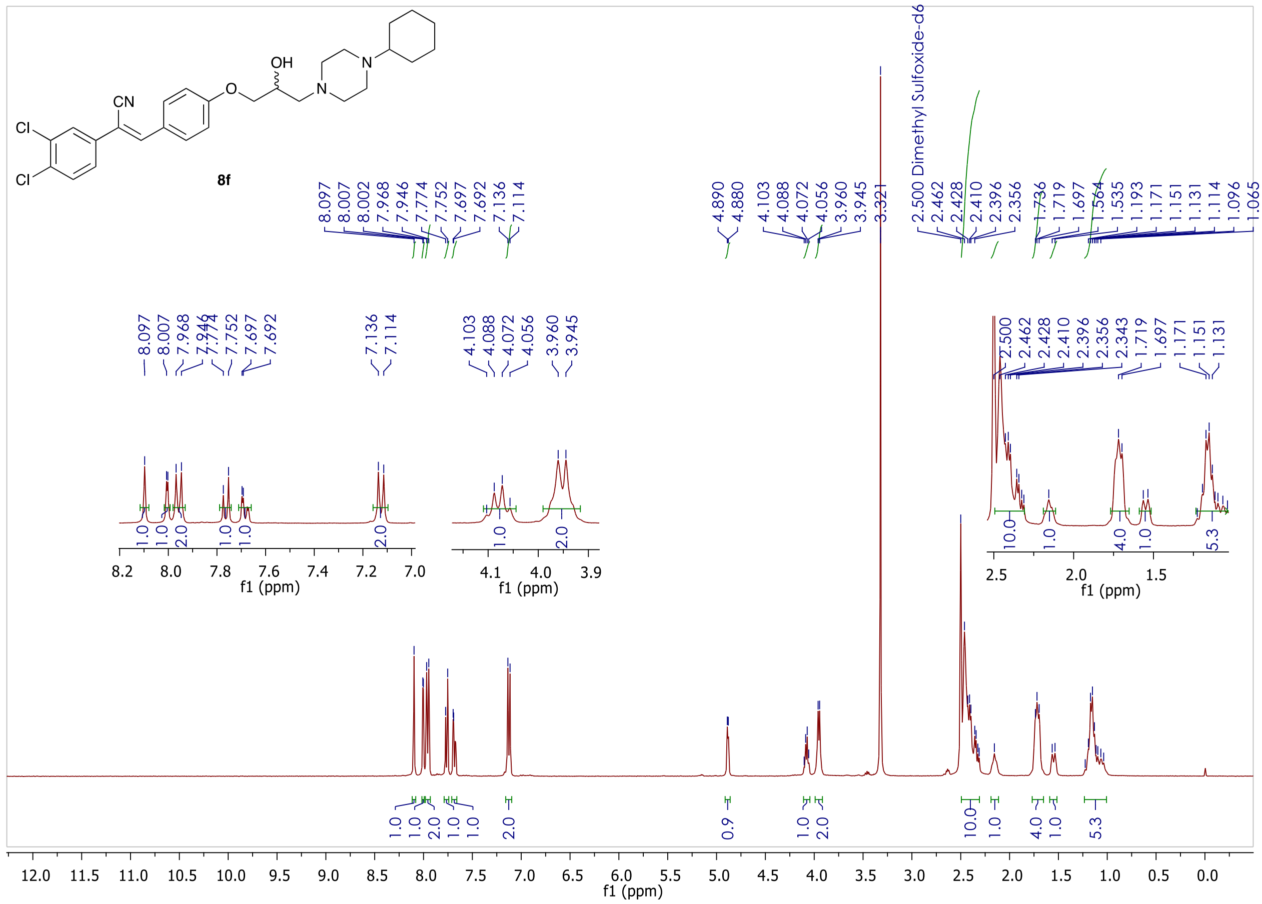

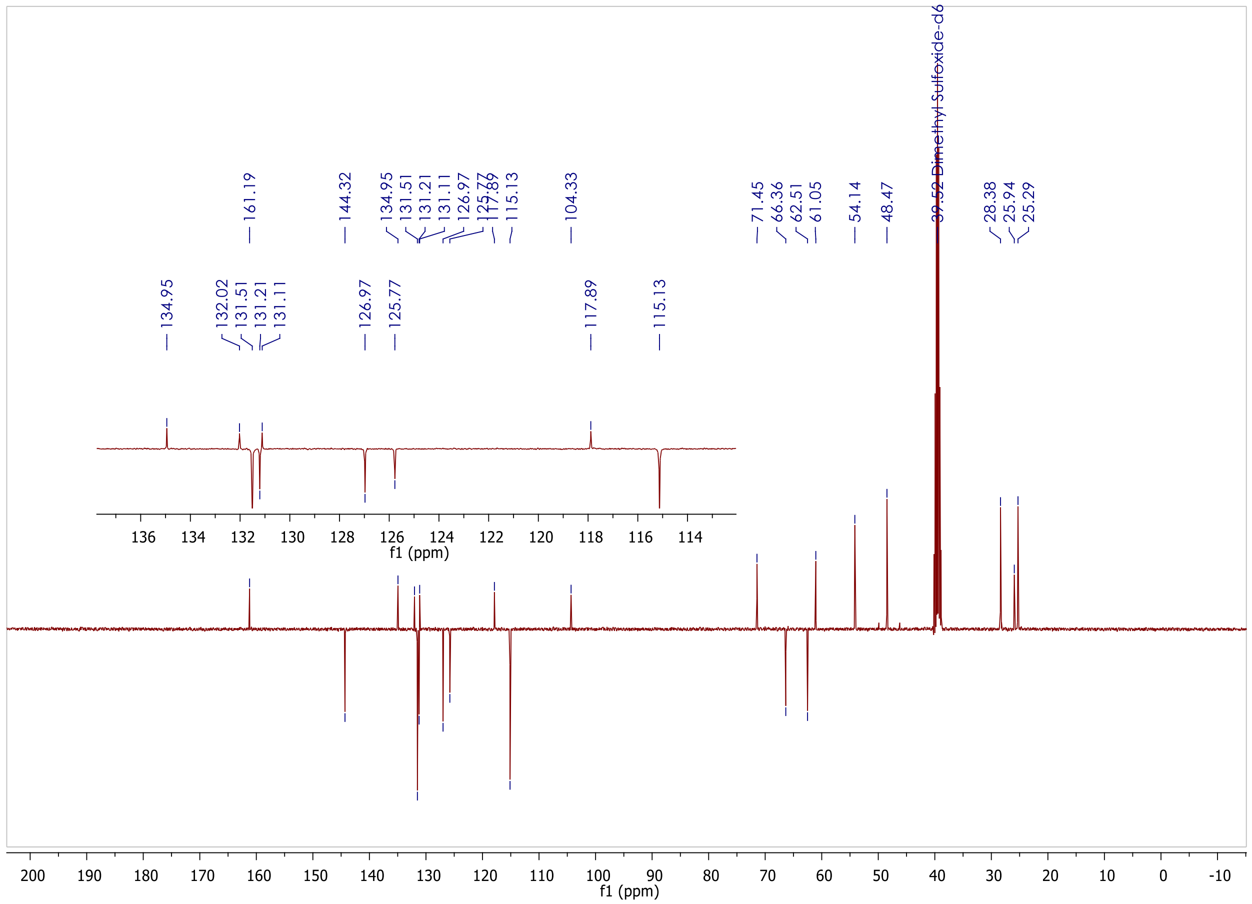

(*Z*)-2-(3,4-dichlorophenyl)-3-(4-(2-hydroxy-3-(piperidin-1-yl)propoxy)phenyl)acrylonitrile (**8g**)

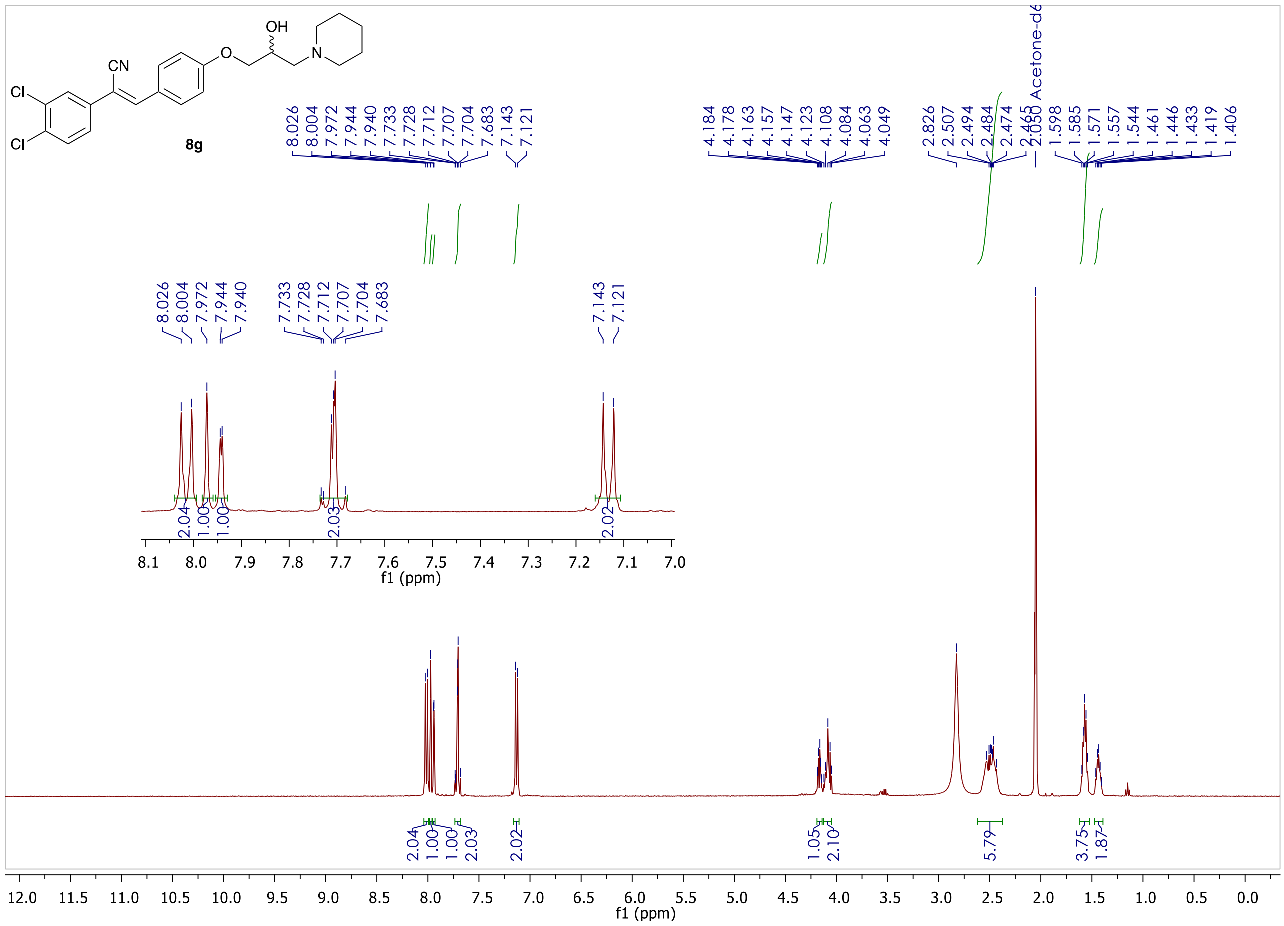

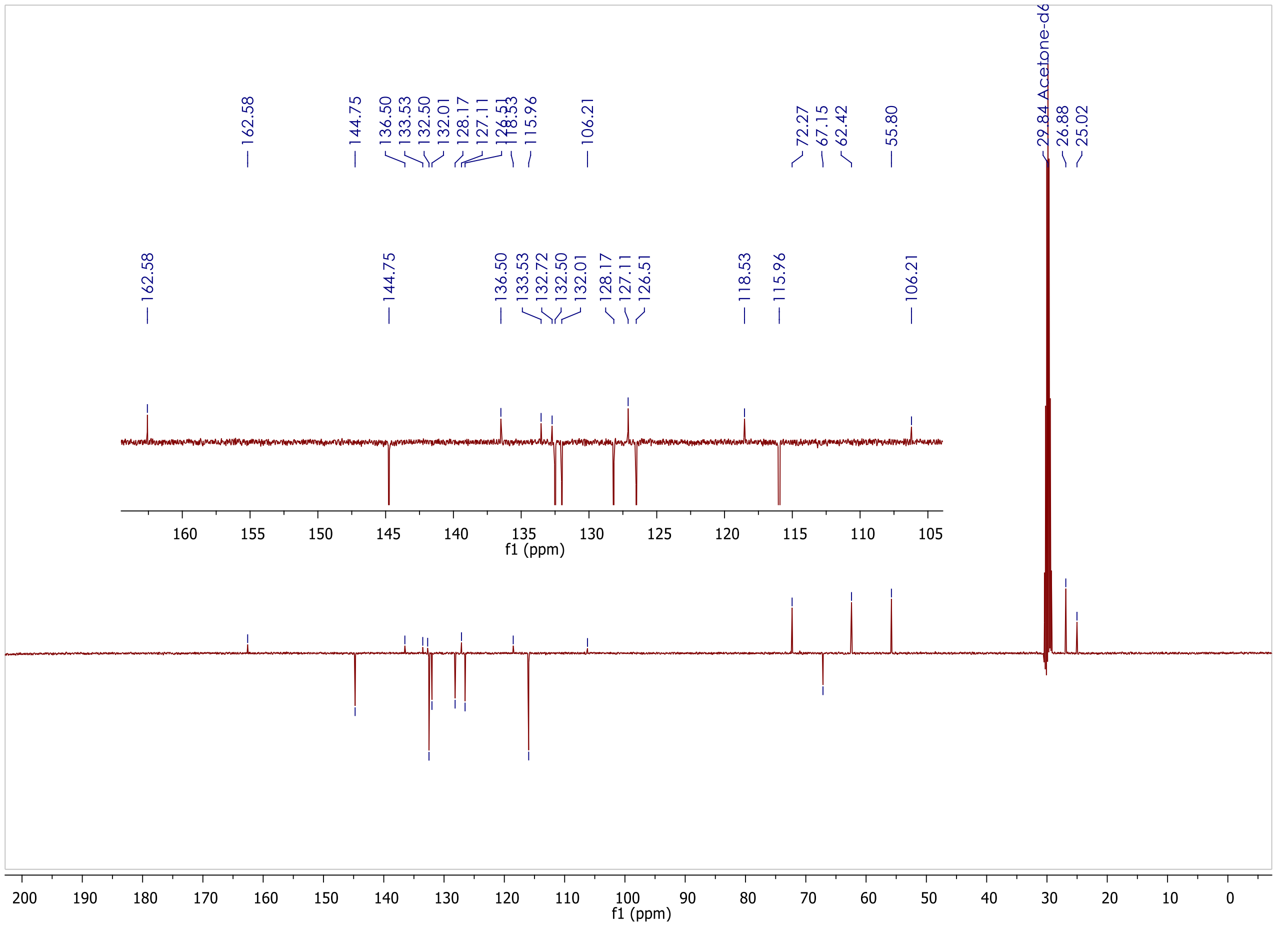

(*S*,*Z*)-2-(3,4-dichlorophenyl)-3-(4-(2-hydroxy-3-(piperidin-1-yl)propoxy)phenyl)acrylonitrile (**8h**)

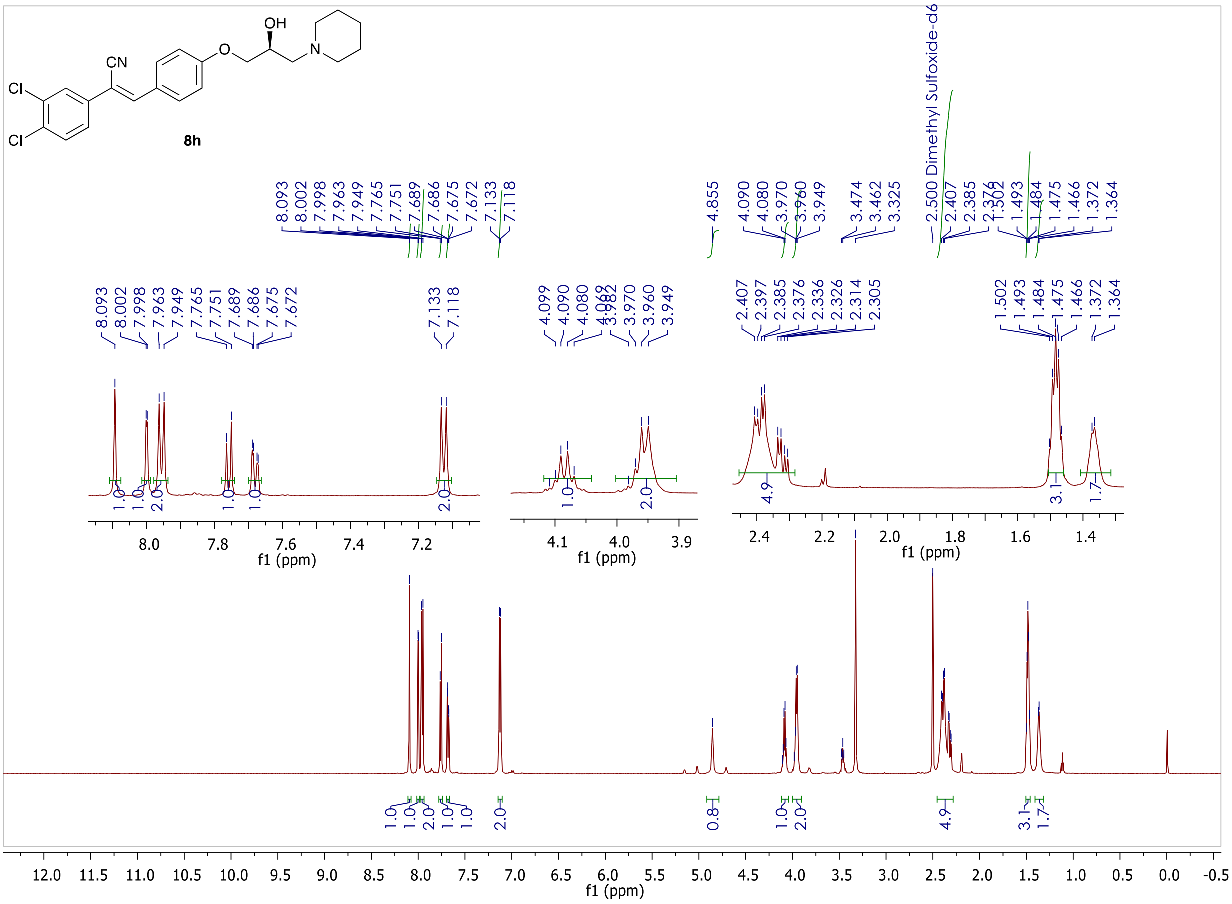

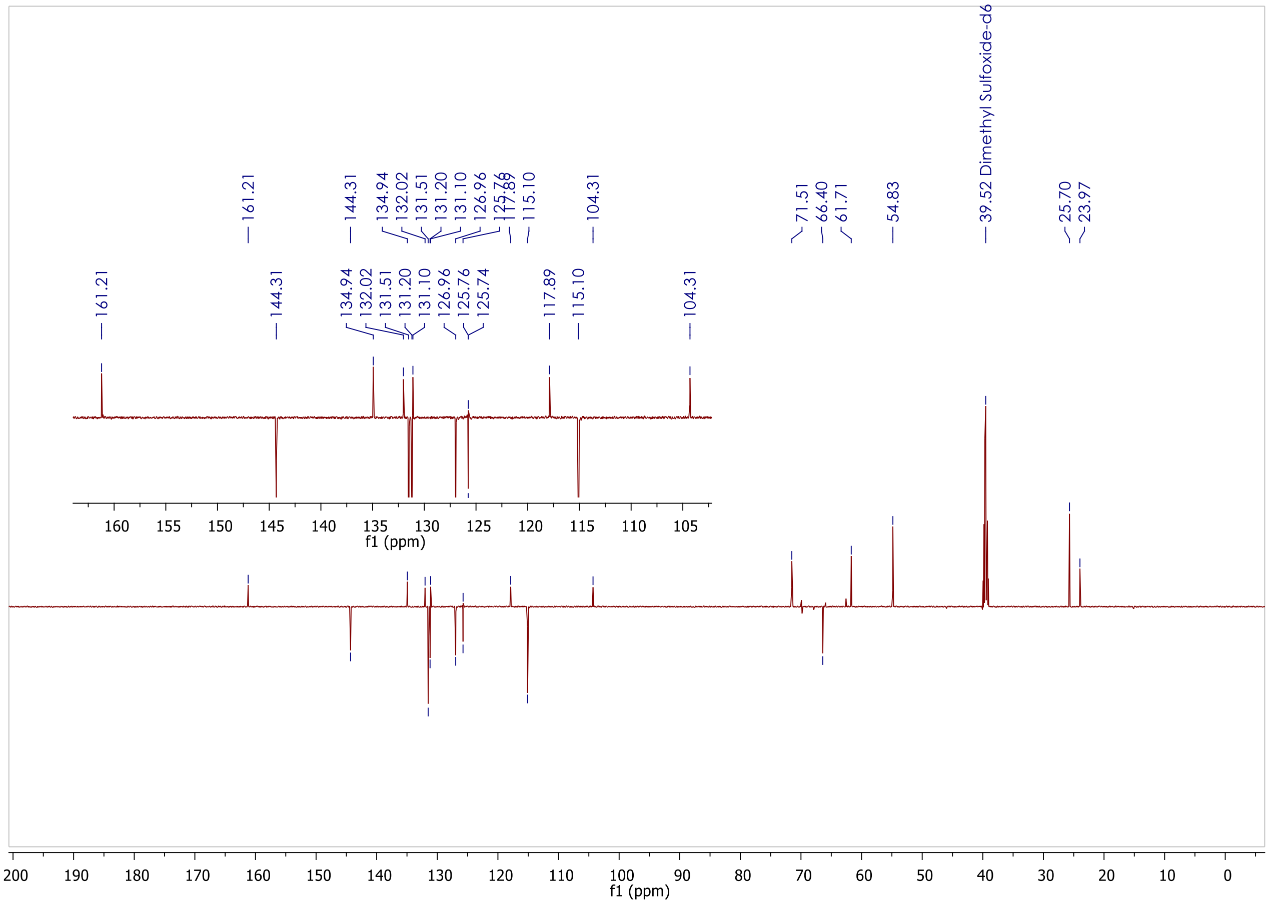

(*R*,*Z*)-2-(3,4-dichlorophenyl)-3-(4-(2-hydroxy-3-(piperidin-1-yl)propoxy)phenyl)acrylonitrile (**8i**)

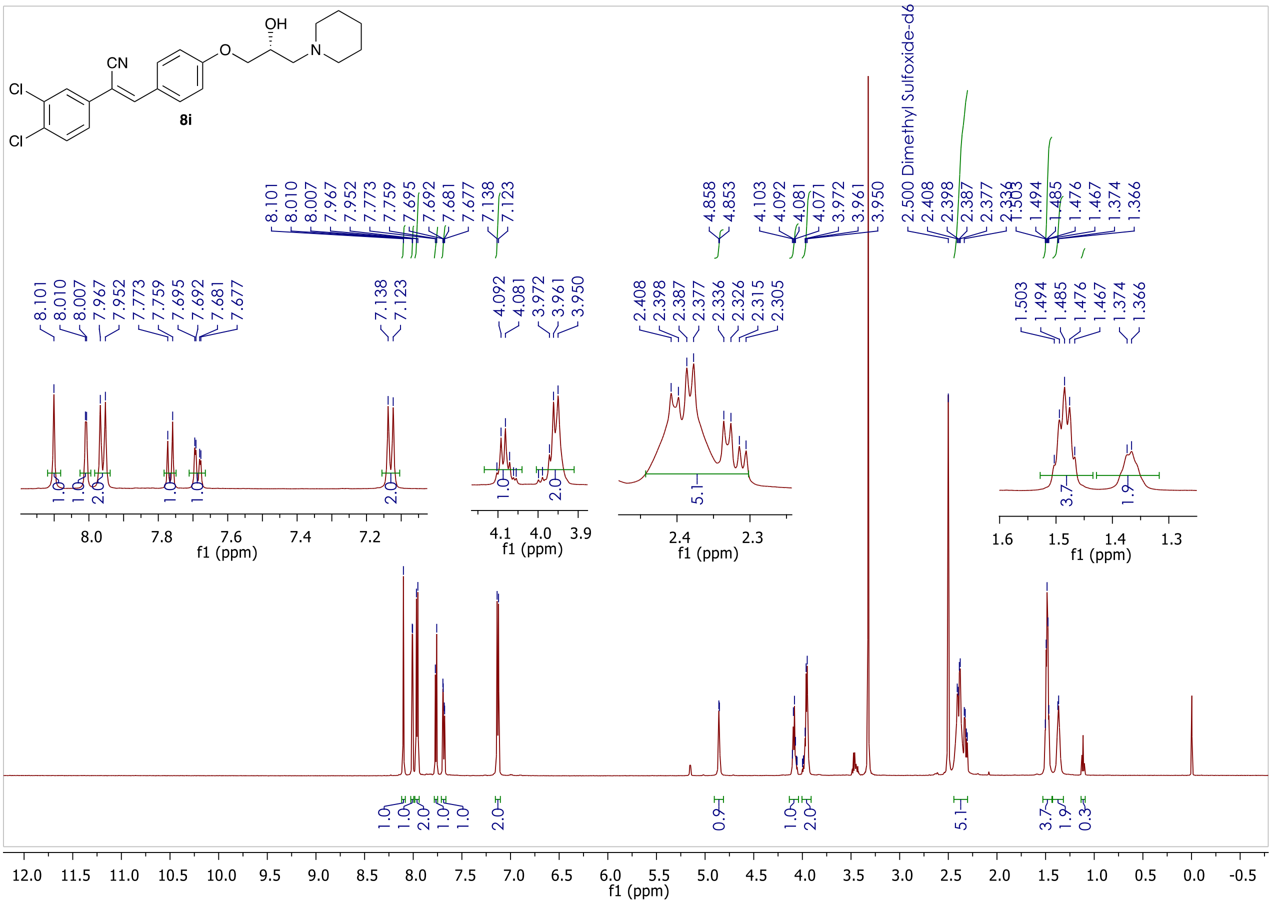

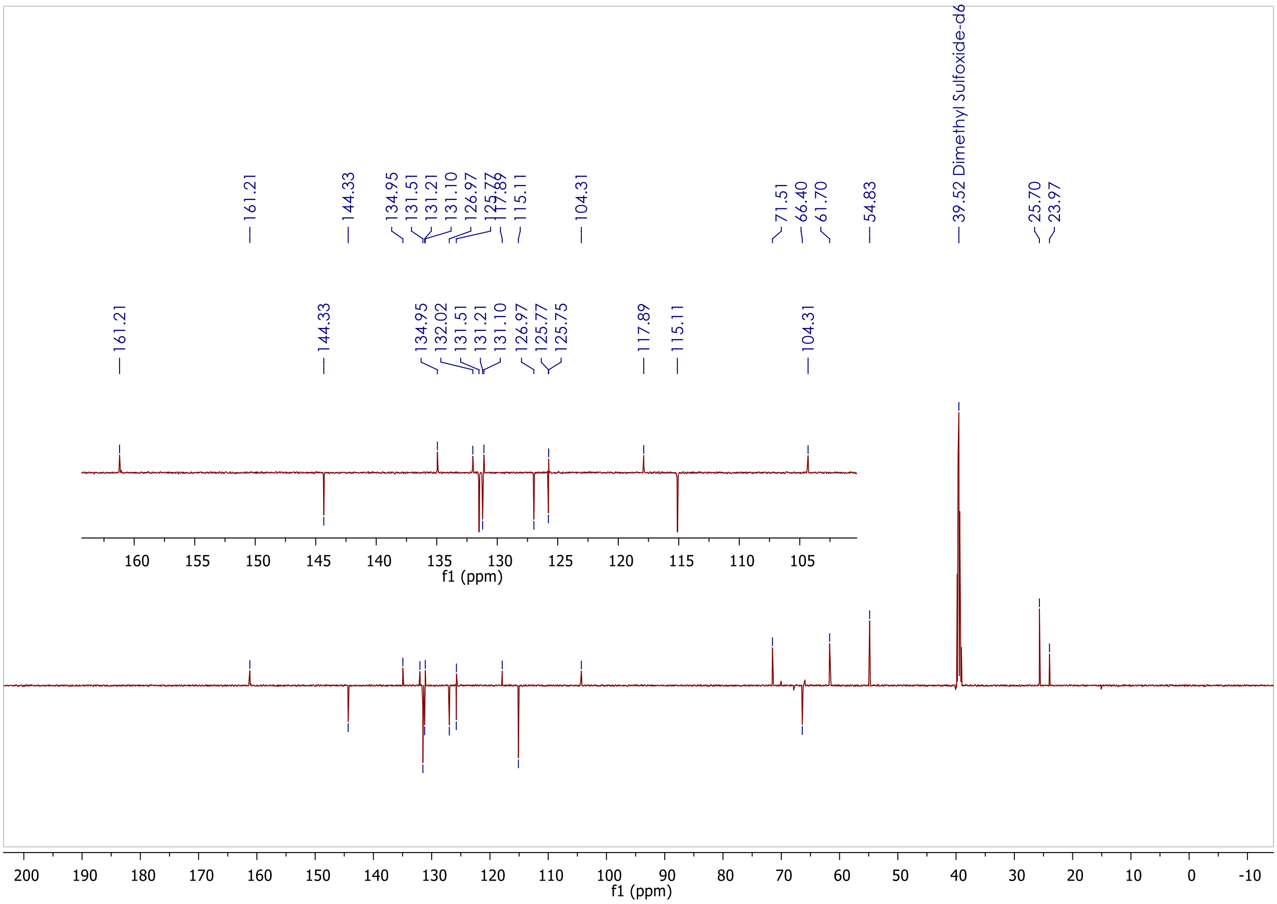

(*Z*)-2-(3,4-dichlorophenyl)-3-(4-(2-hydroxy-3-(d_{10}-piperidin-1-yl)propoxy)phenyl)acrylonitrile (**8j**)

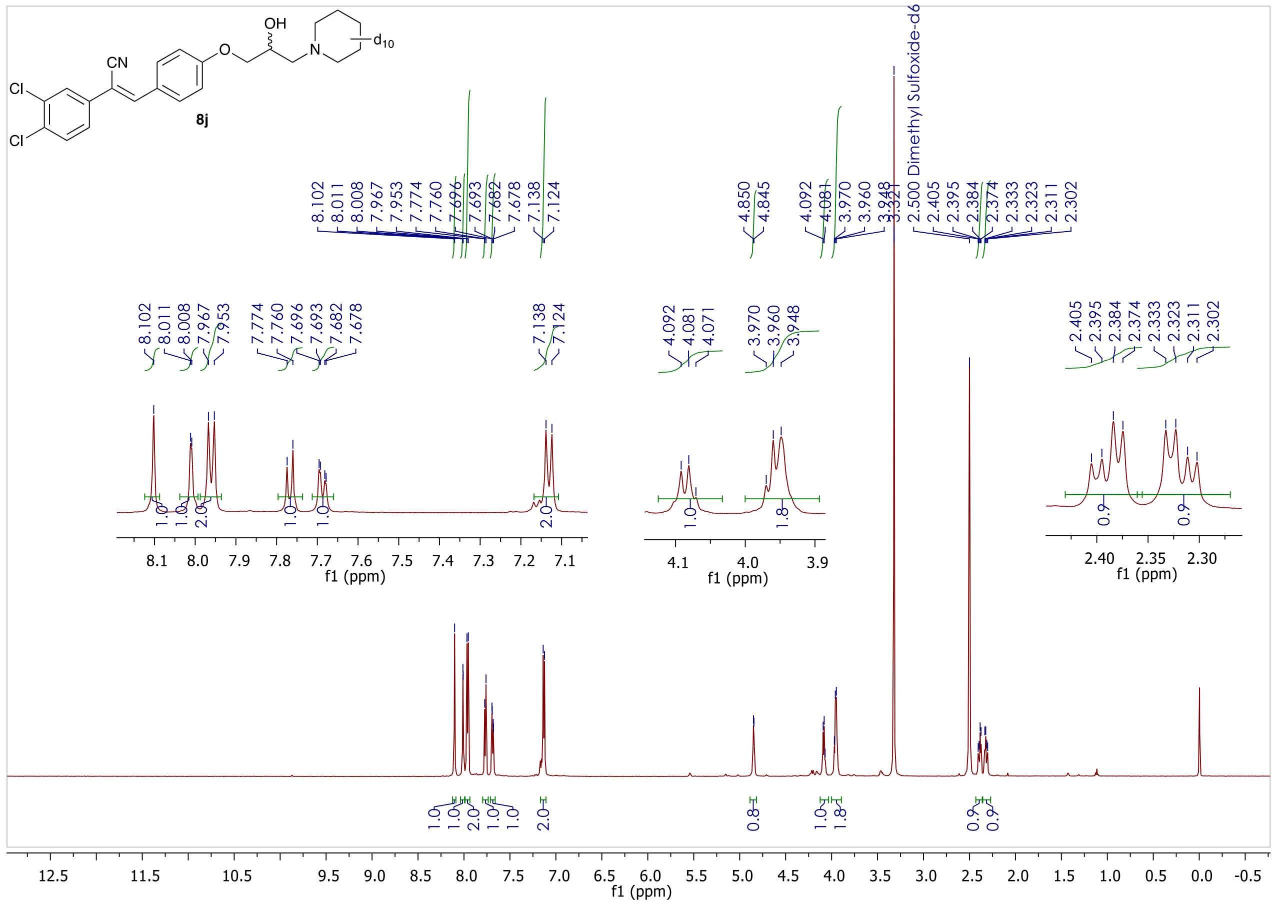

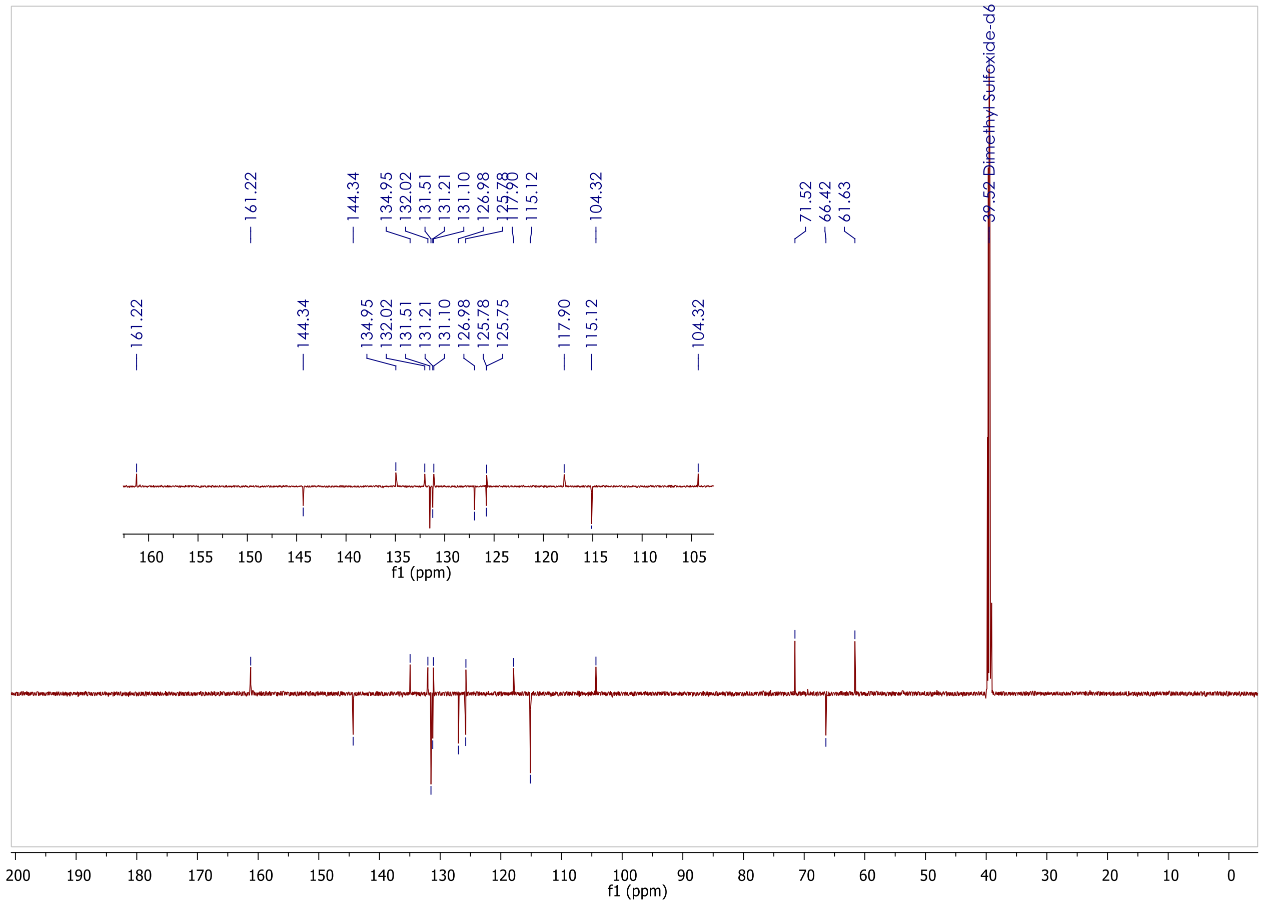

(*R*,*Z*)-3-(4-(3-(4-acetylpiperazin-1-yl)-2-hydroxypropoxy)phenyl)-2-(3,4-dichlorophenyl)acrylonitrilex (**8k**)

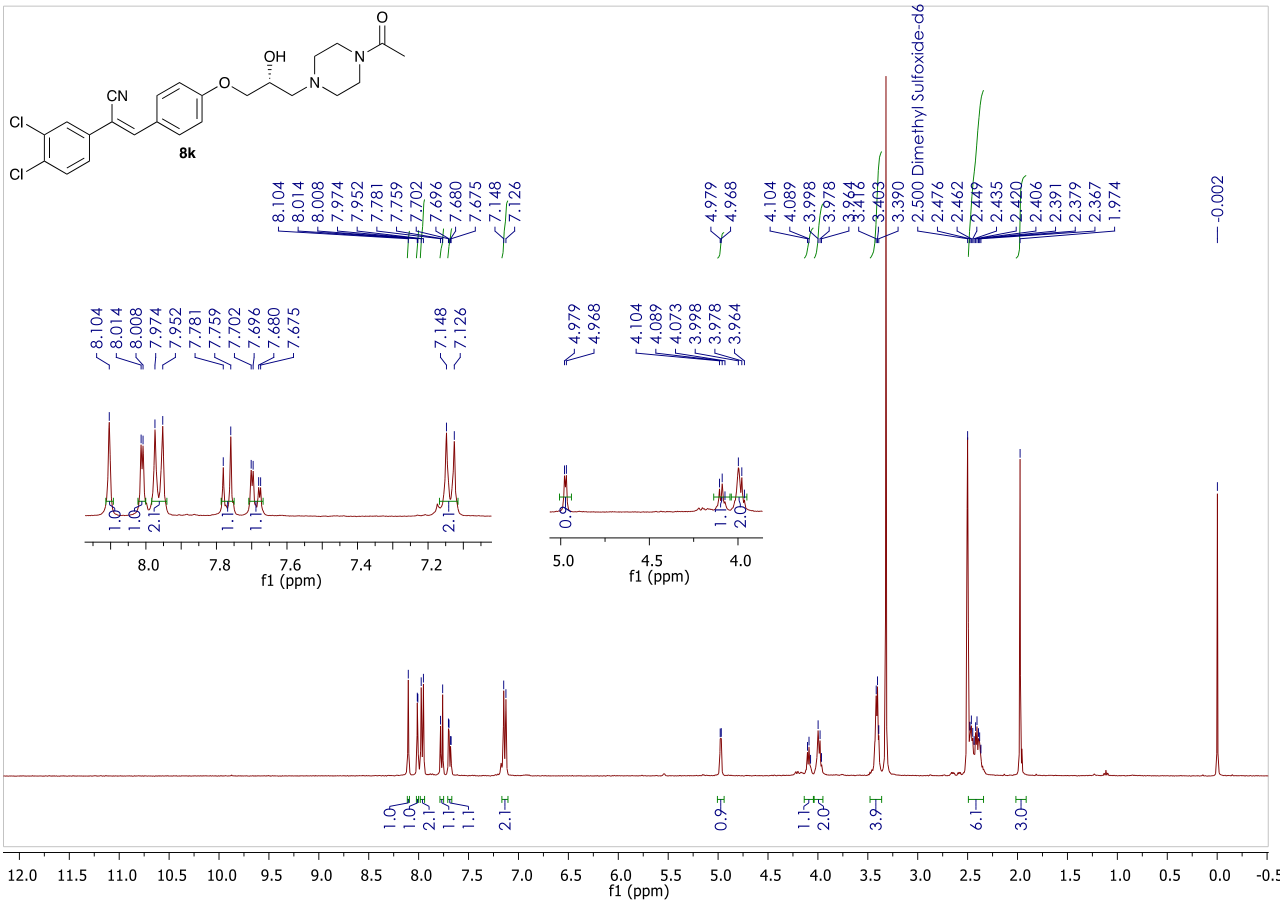

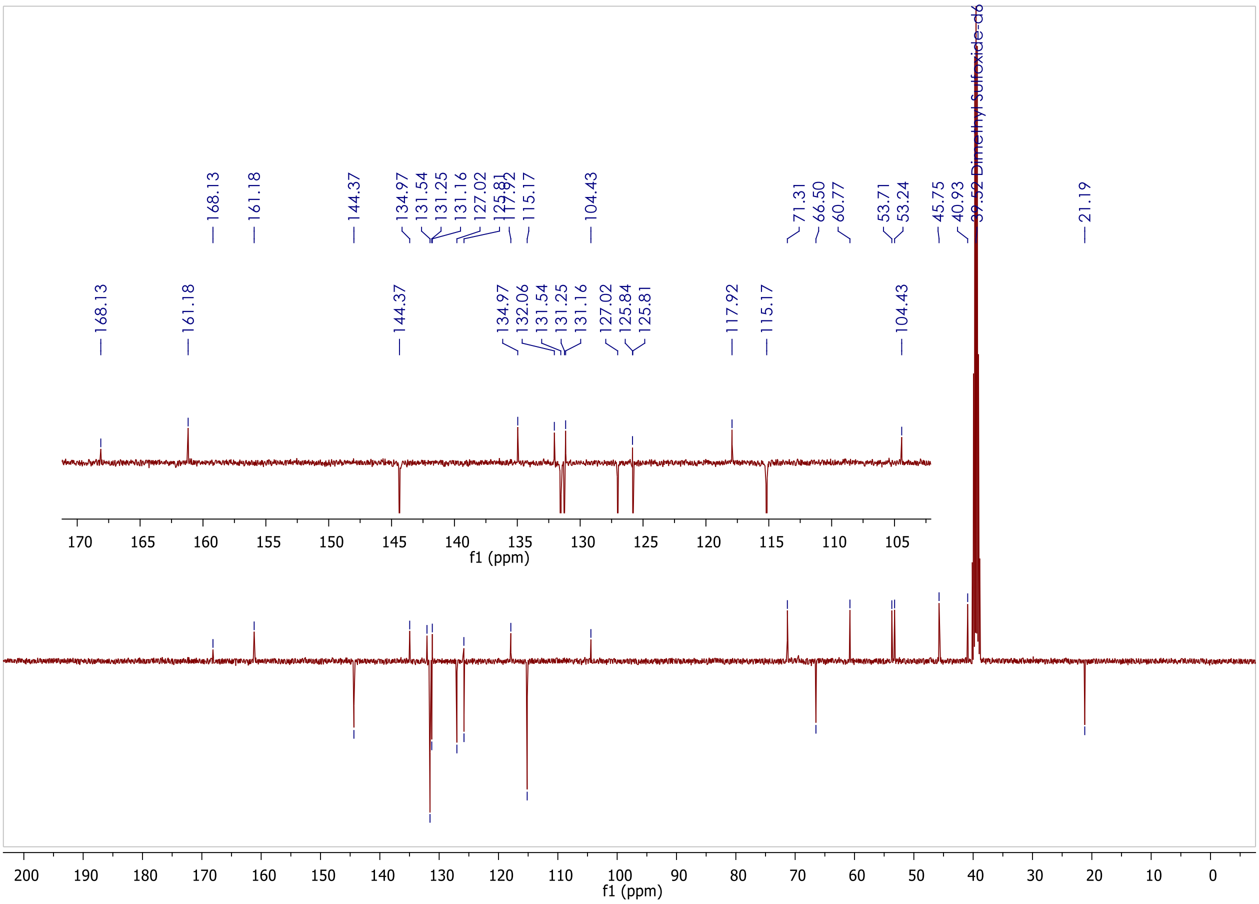

(*Z*)-2-(3,4-dichlorophenyl)-3-(4-(2-hydroxy-3-morpholinopropoxy)phenyl)acrylonitrile (**8l**)

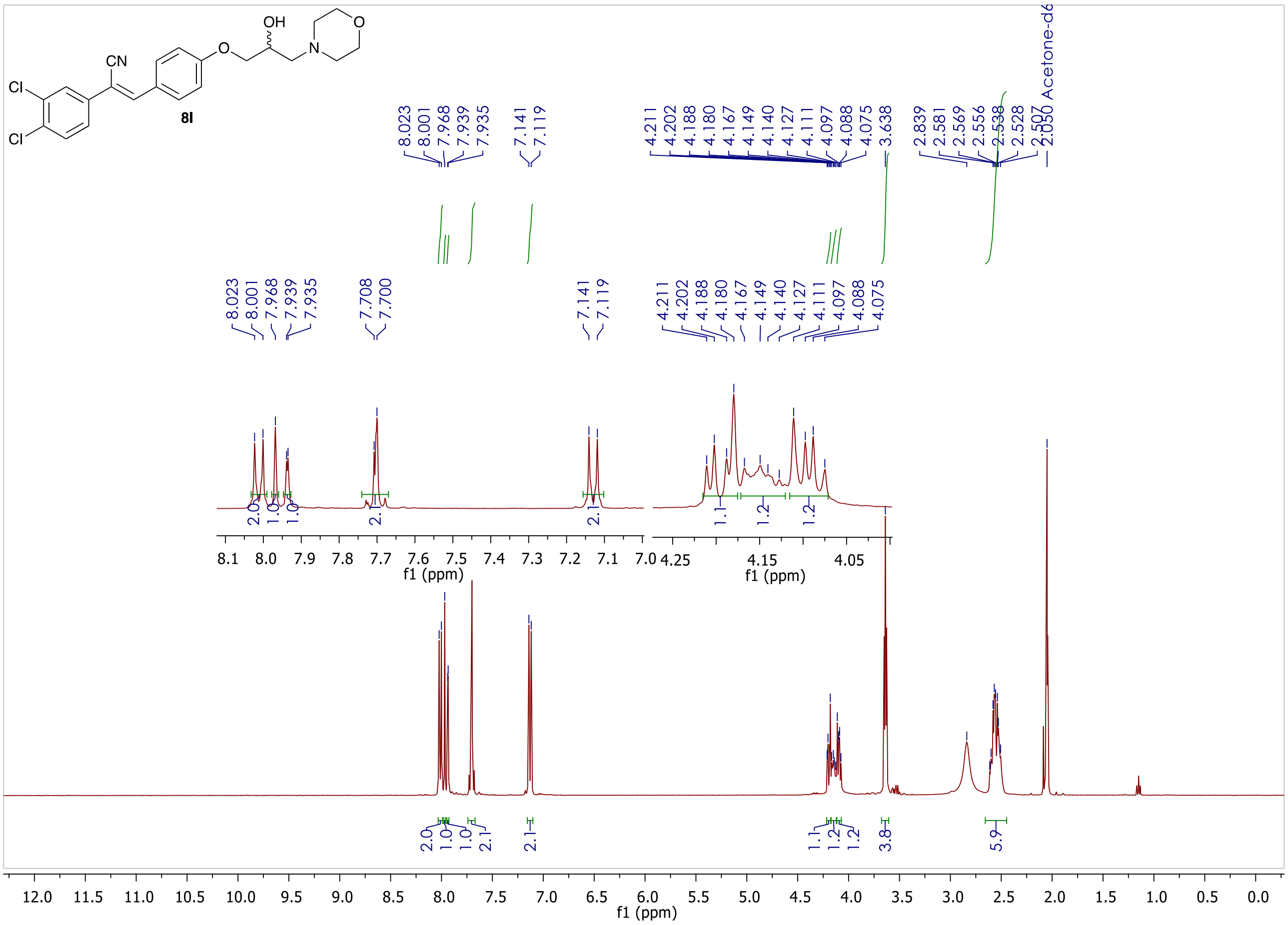

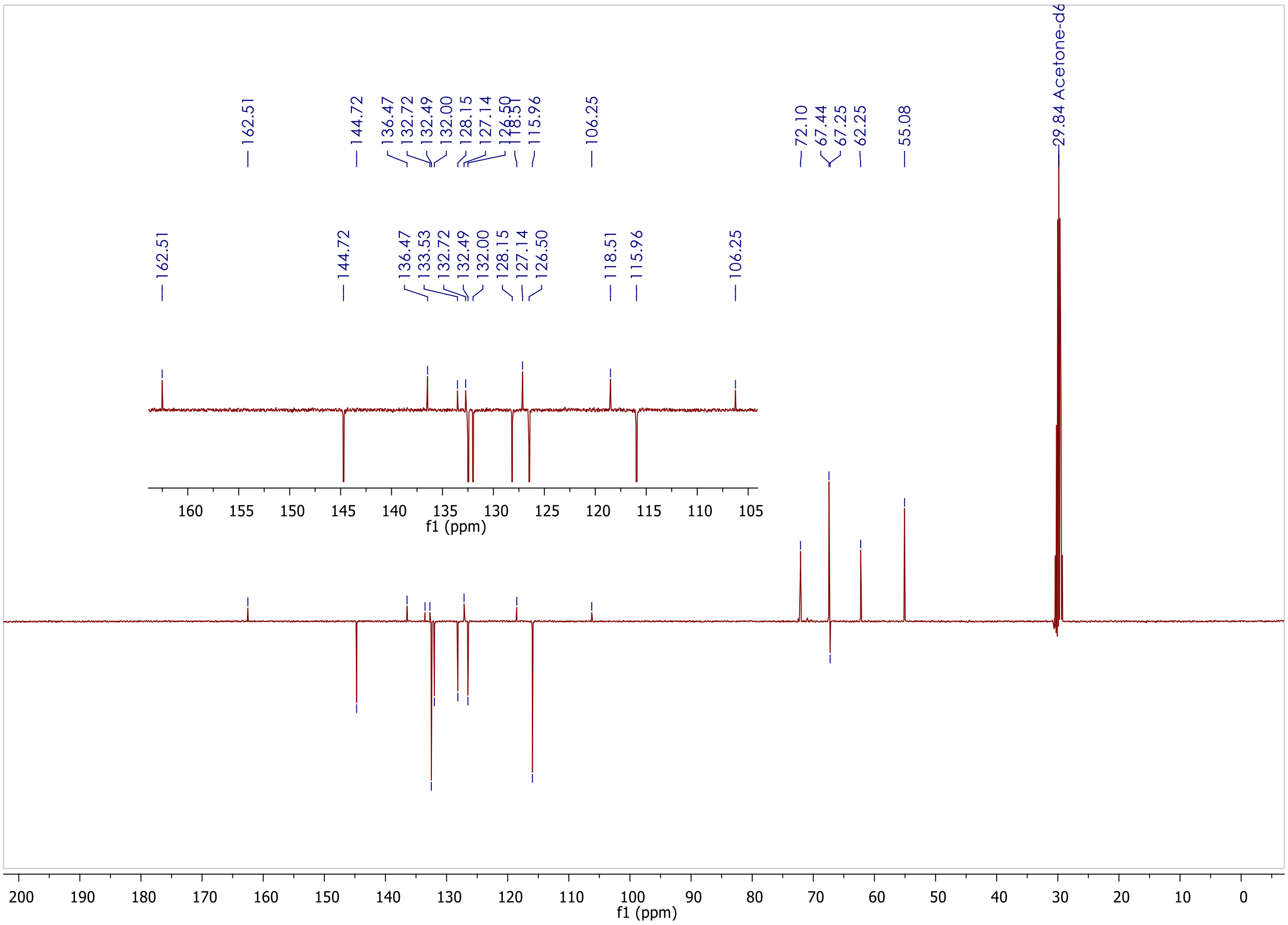

(*Z*)-2-(3,4-dichlorophenyl)-3-(2-(2-hydroxy-3-(4-phenylpiperazin-1-yl)propoxy)phenyl)acrylonitrile (**8m**)

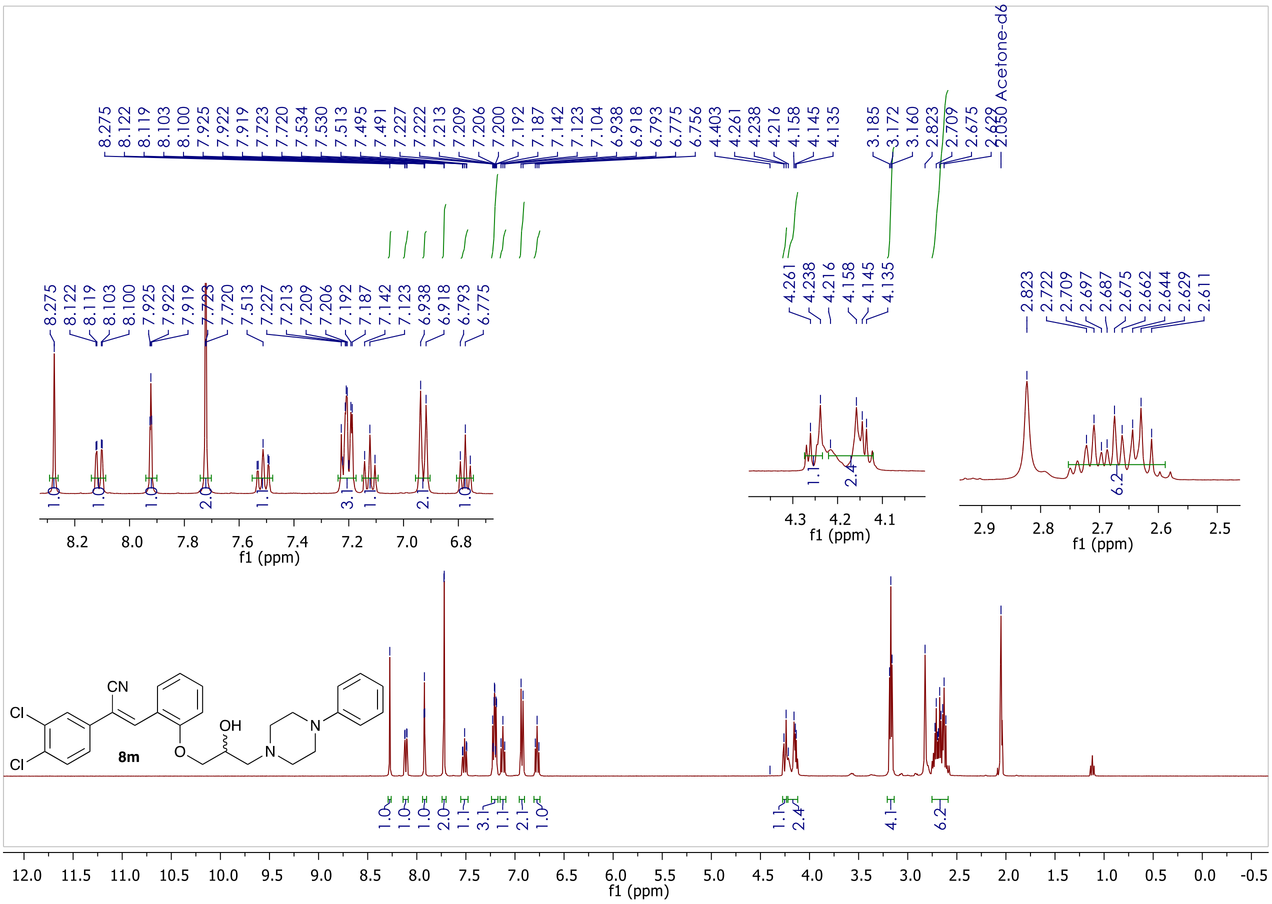

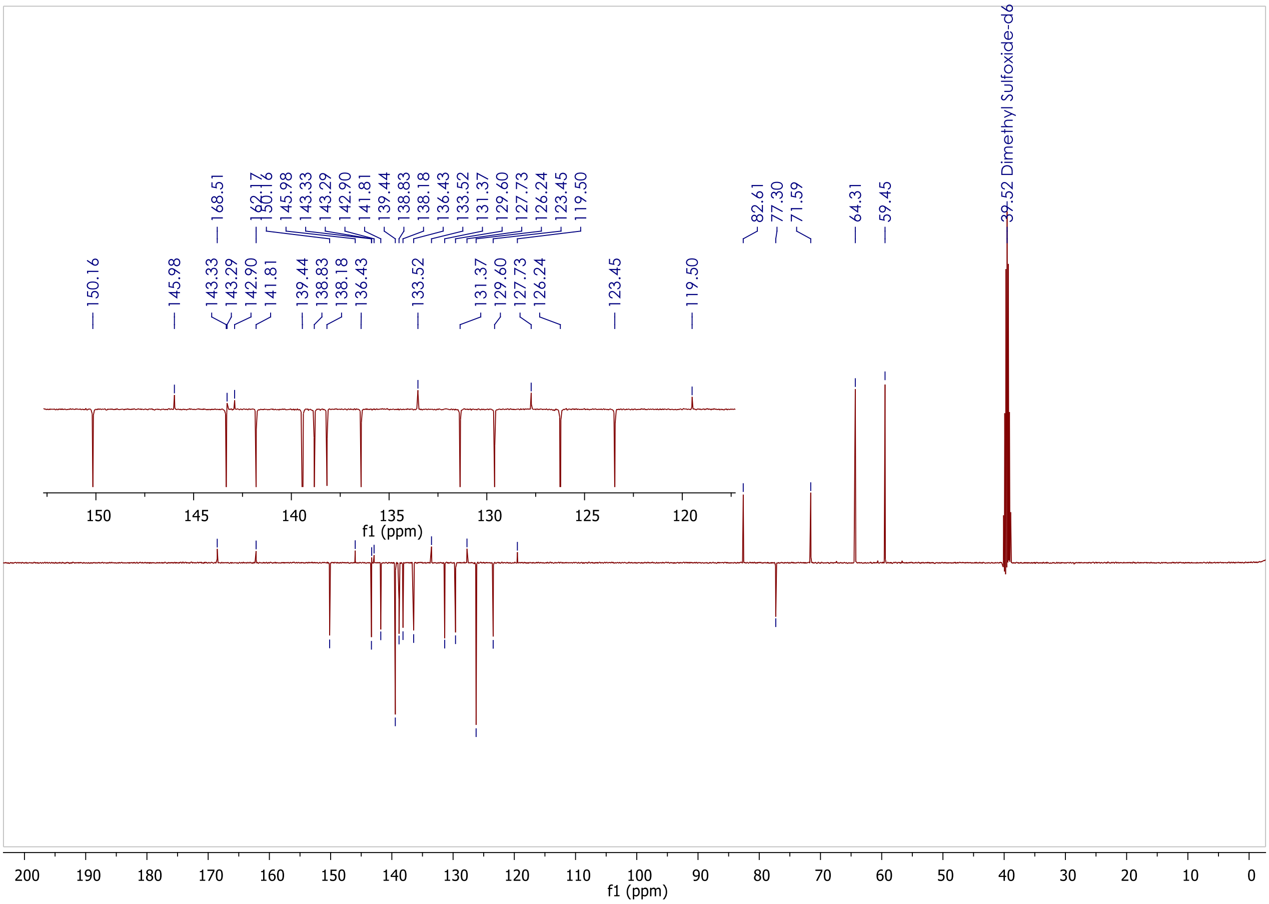

(*Z*)-2-(3,4-dichlorophenyl)-3-(3-(2-hydroxy-3-(4-phenylpiperazin-1-yl)propoxy)phenyl)acrylonitrile (**8n**)

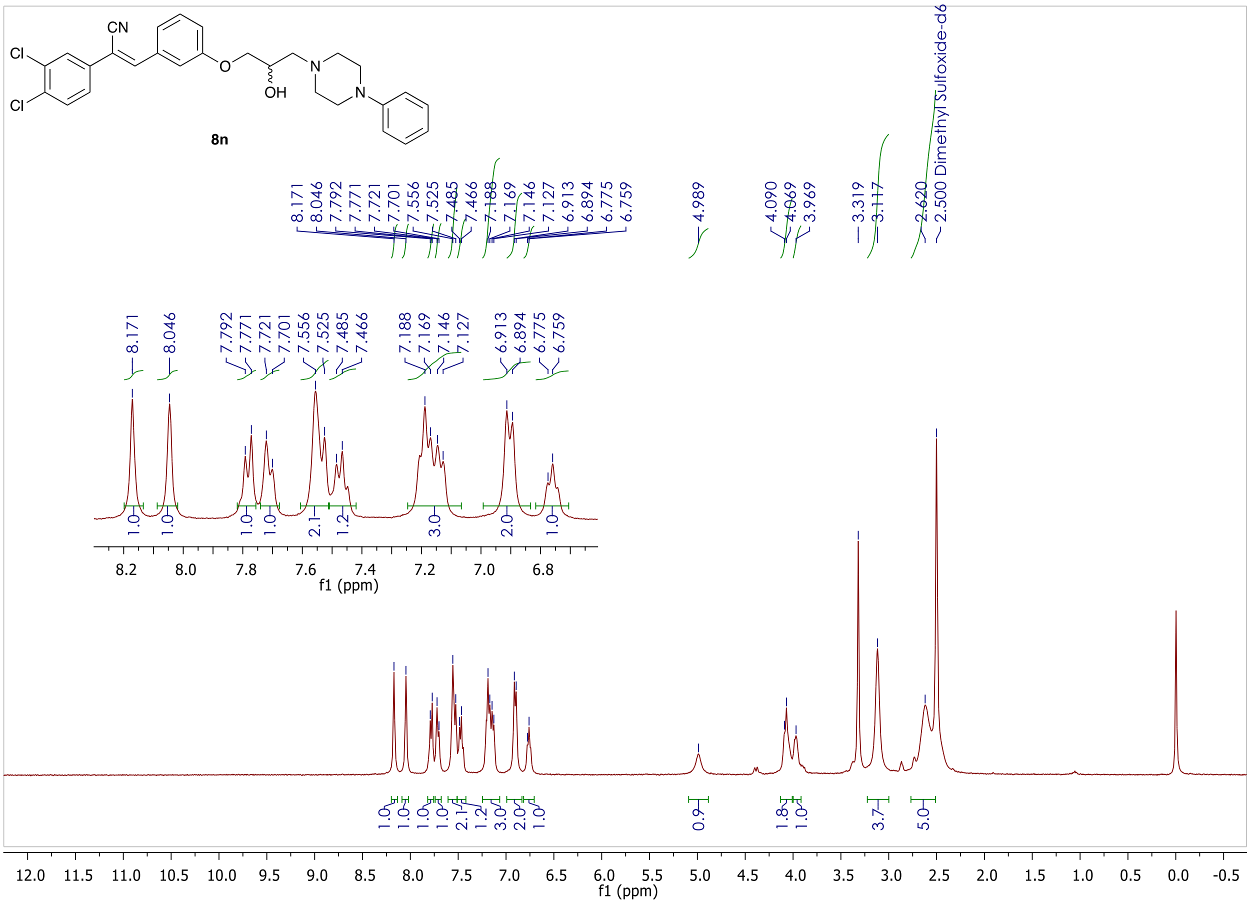

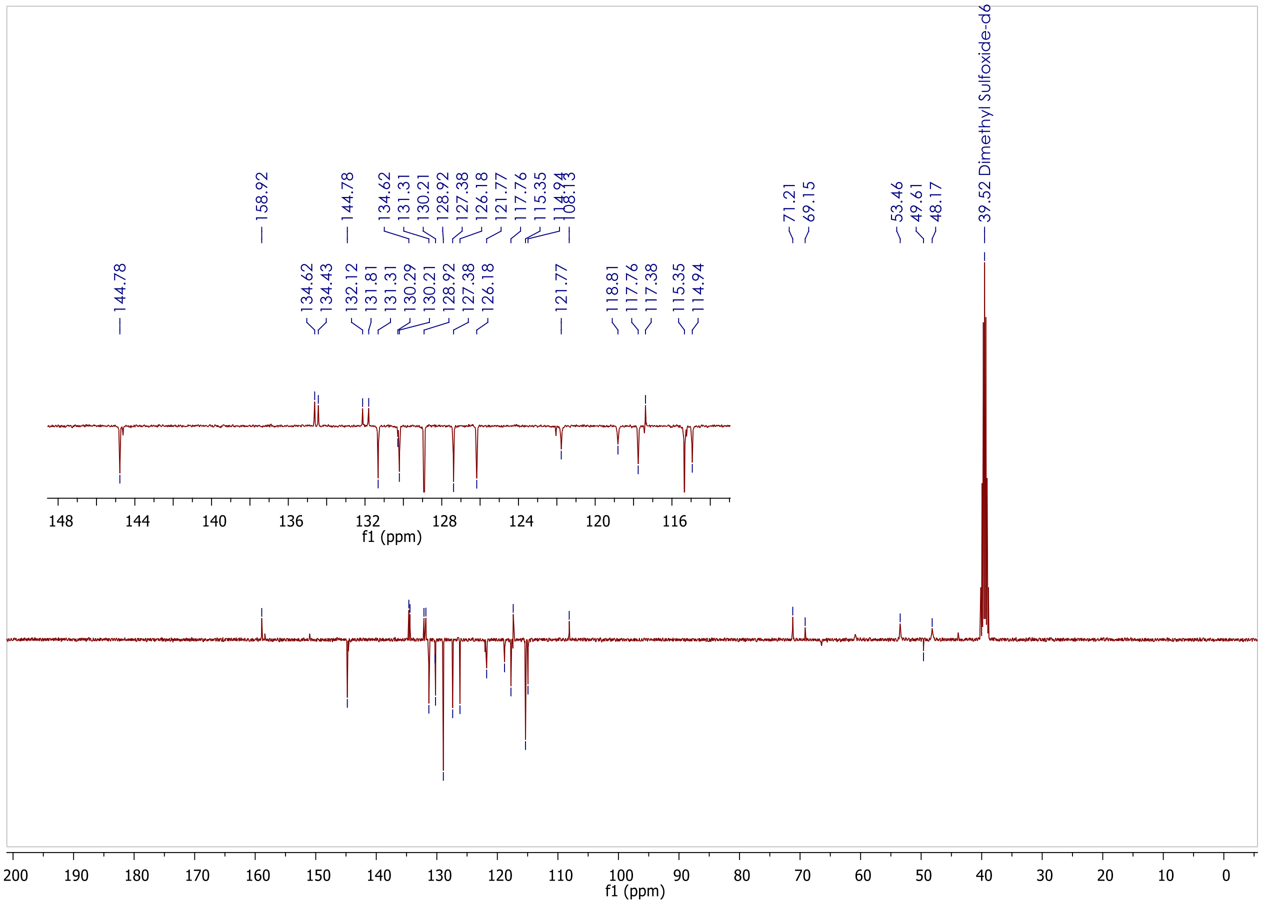

(*Z*)-2-(3,4-dichlorophenyl)-3-(4-(2-hydroxy-3-(4-phenylpiperazin-1-yl)propoxy)phenyl)acrylonitrile (**8o**)

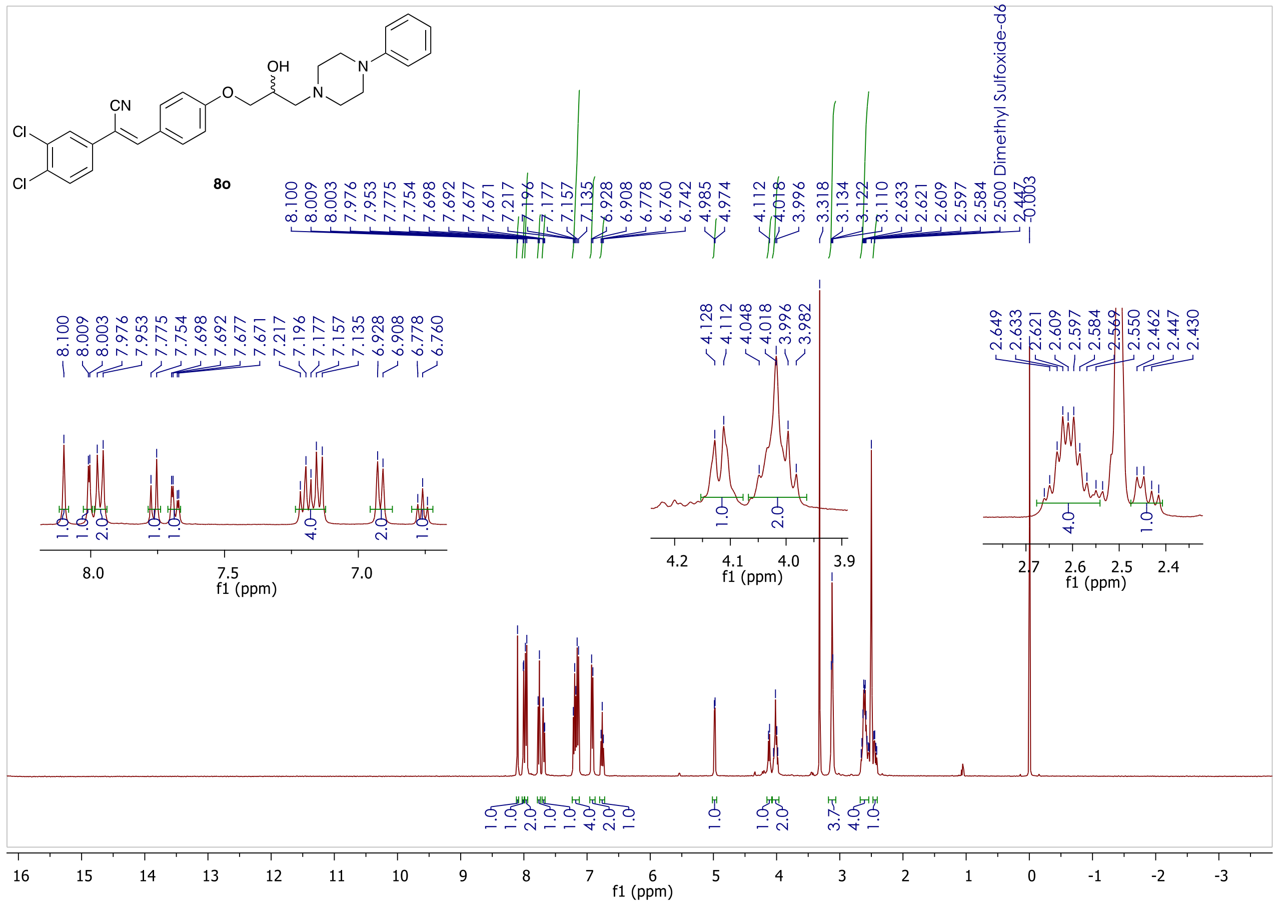

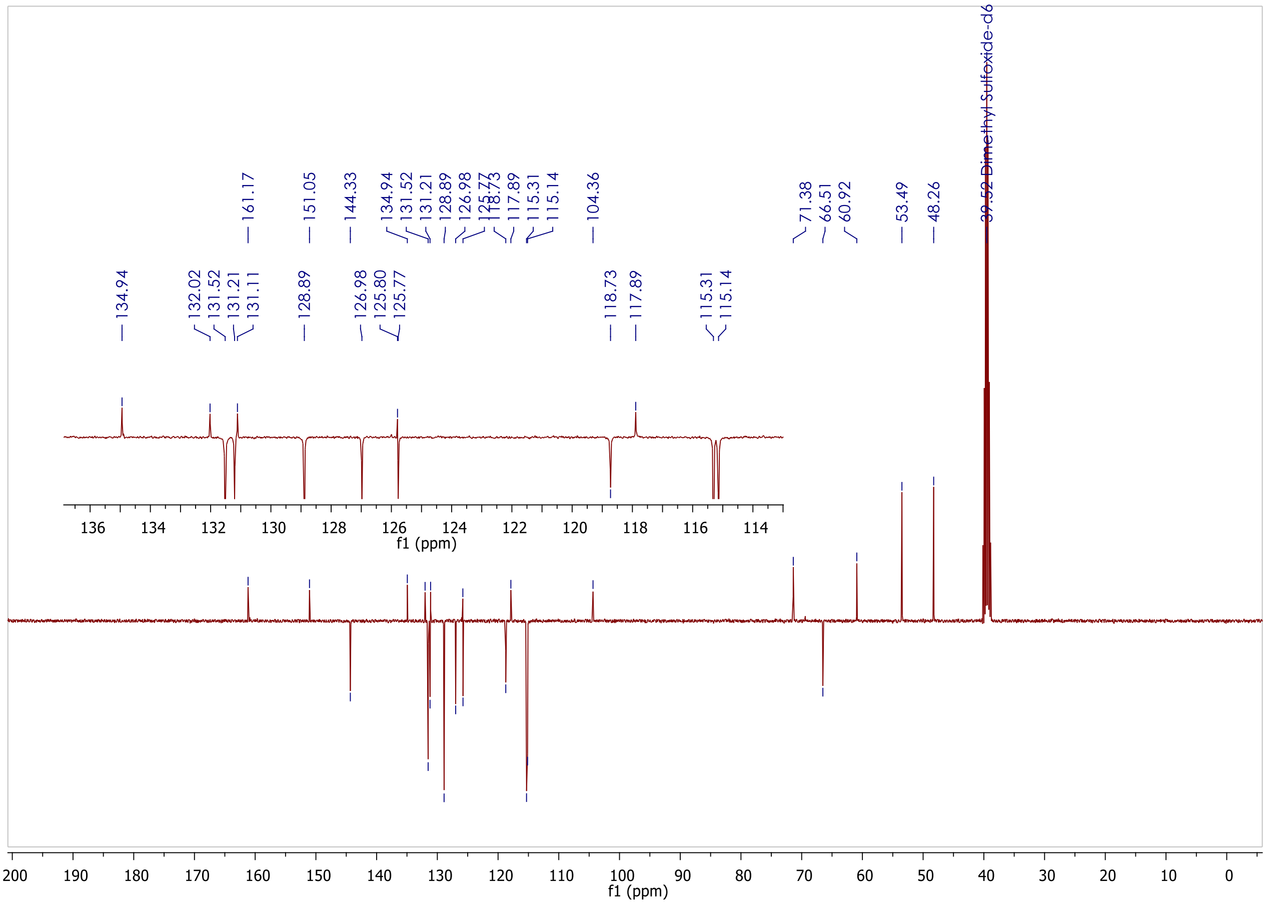

(*Z*)-2-(3,4-dichlorophenyl)-3-(4-(2-hydroxy-3-(4-(*p*-tolyl)piperazin-1-yl)propoxy)phenyl)acrylonitrile (**8p**)

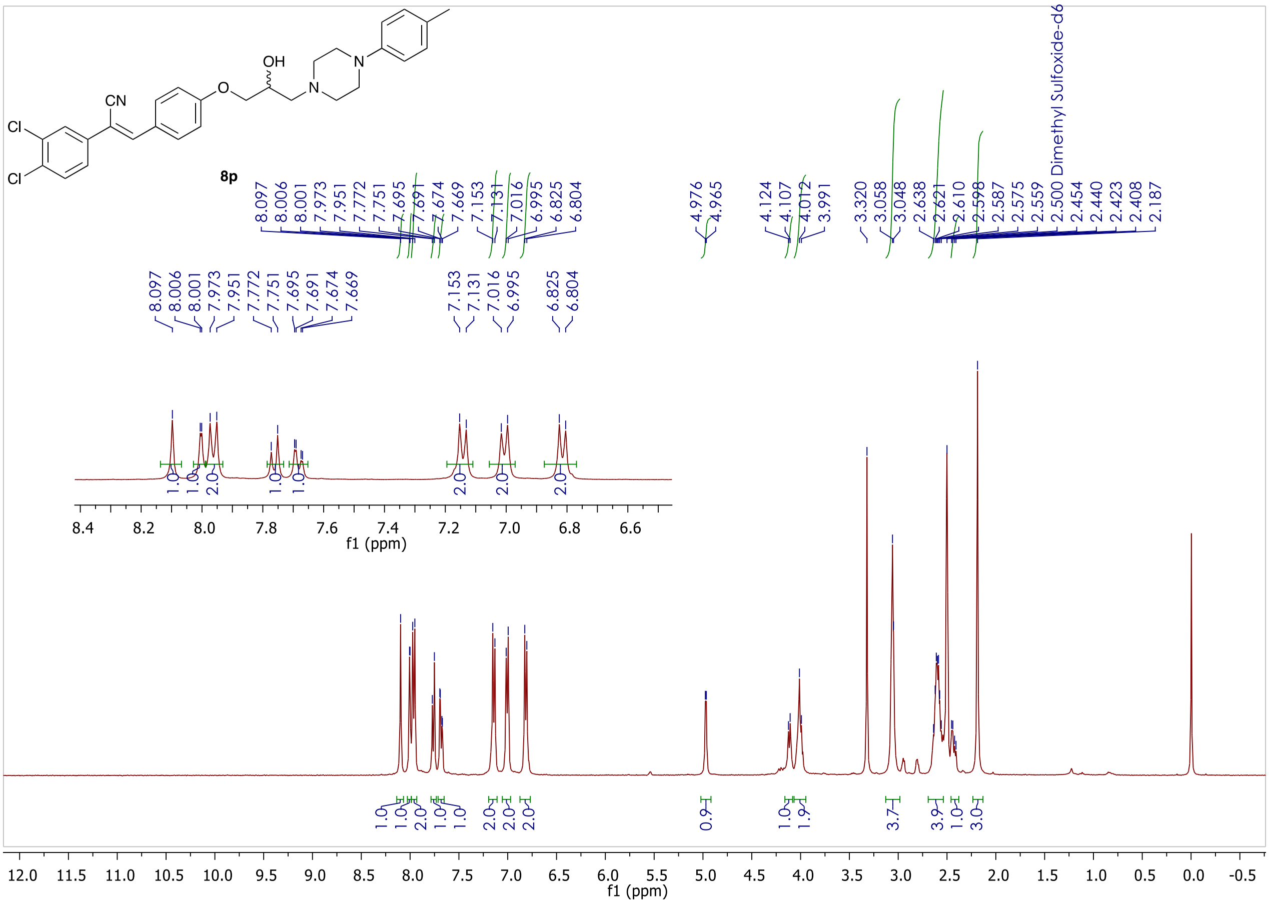

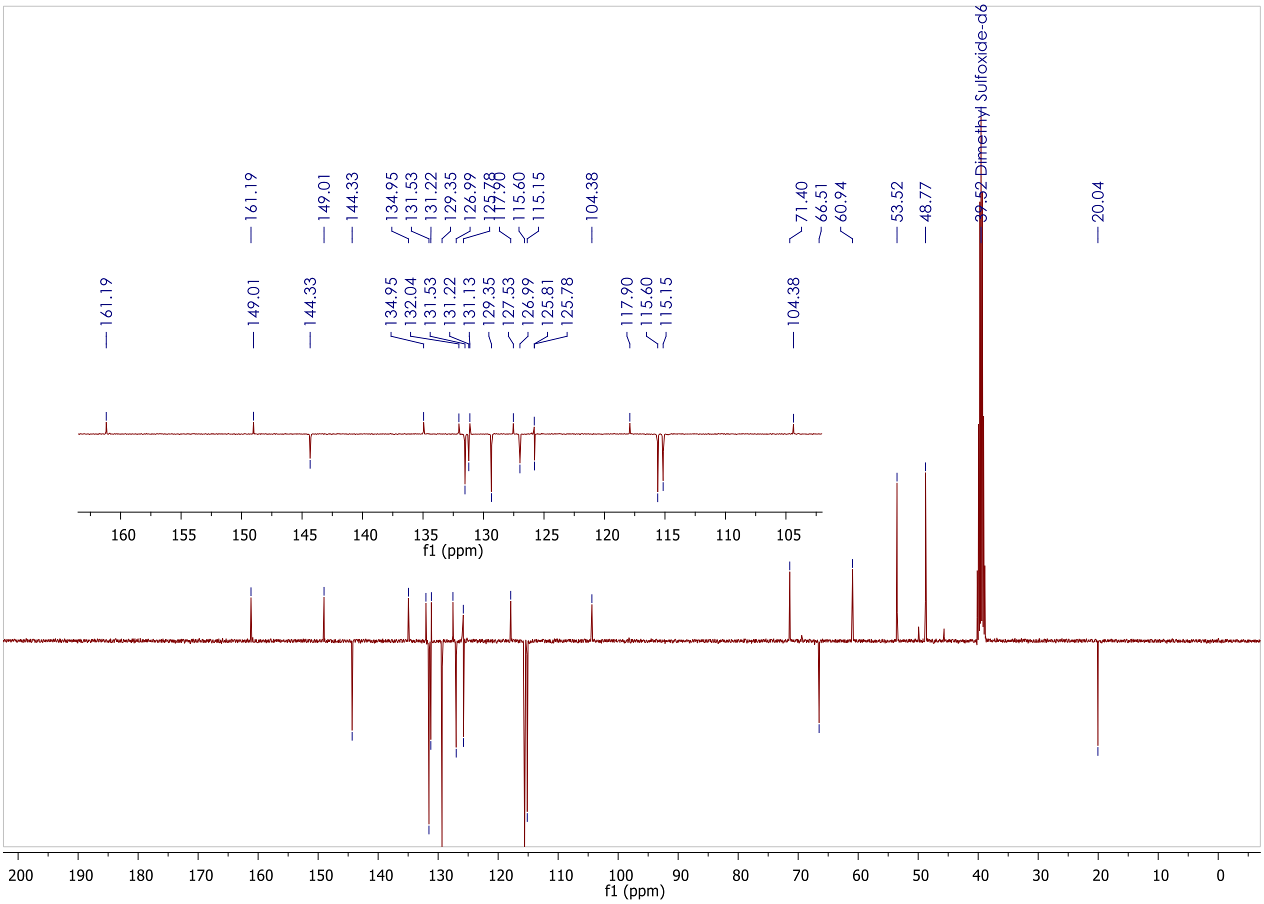

(*Z*)-3-(4-(3-(4-(4-chlorophenyl)piperazin-1-yl)-2-hydroxypropoxy)phenyl)-2-(3,4-dichlorophenyl)acrylonitrile (**8q**)

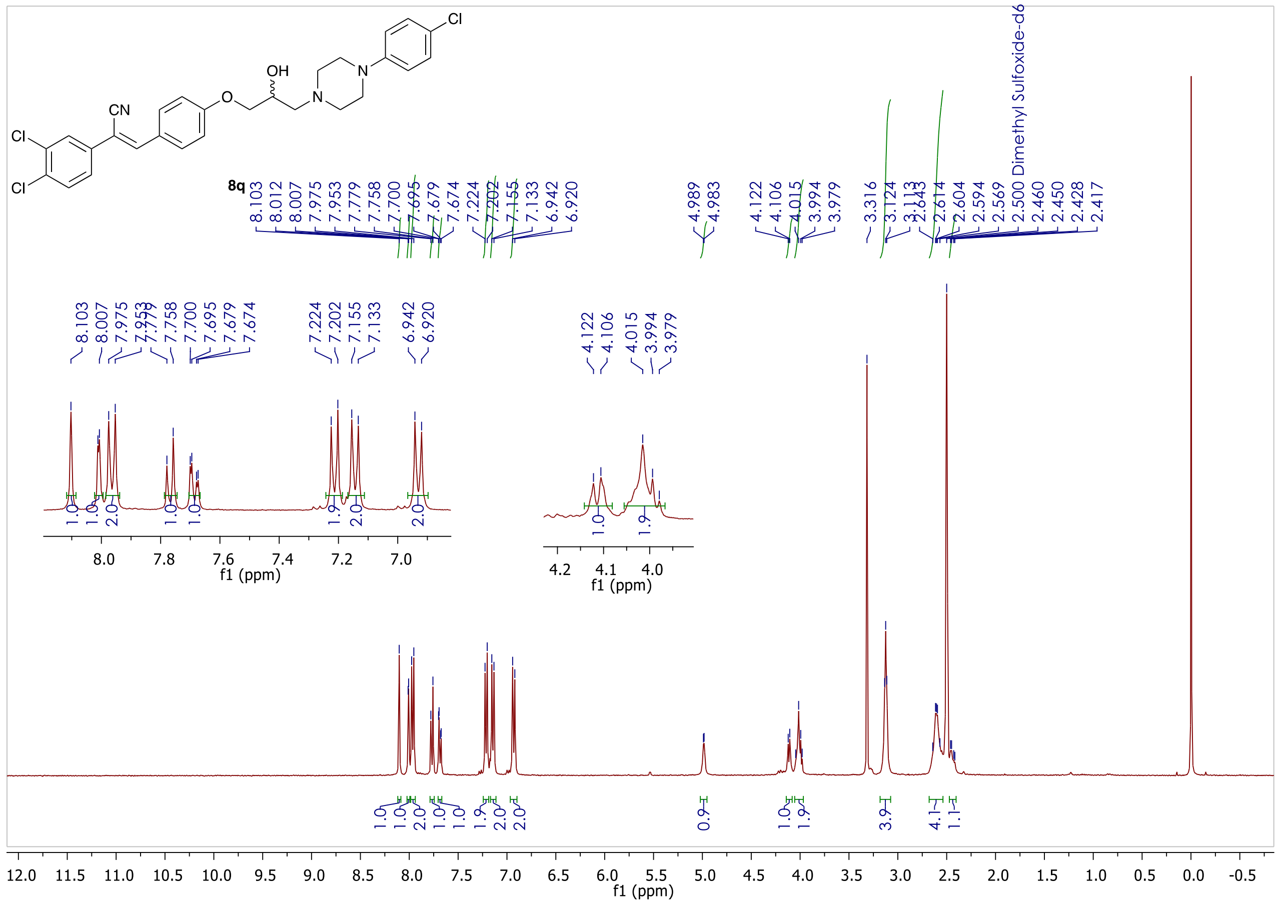

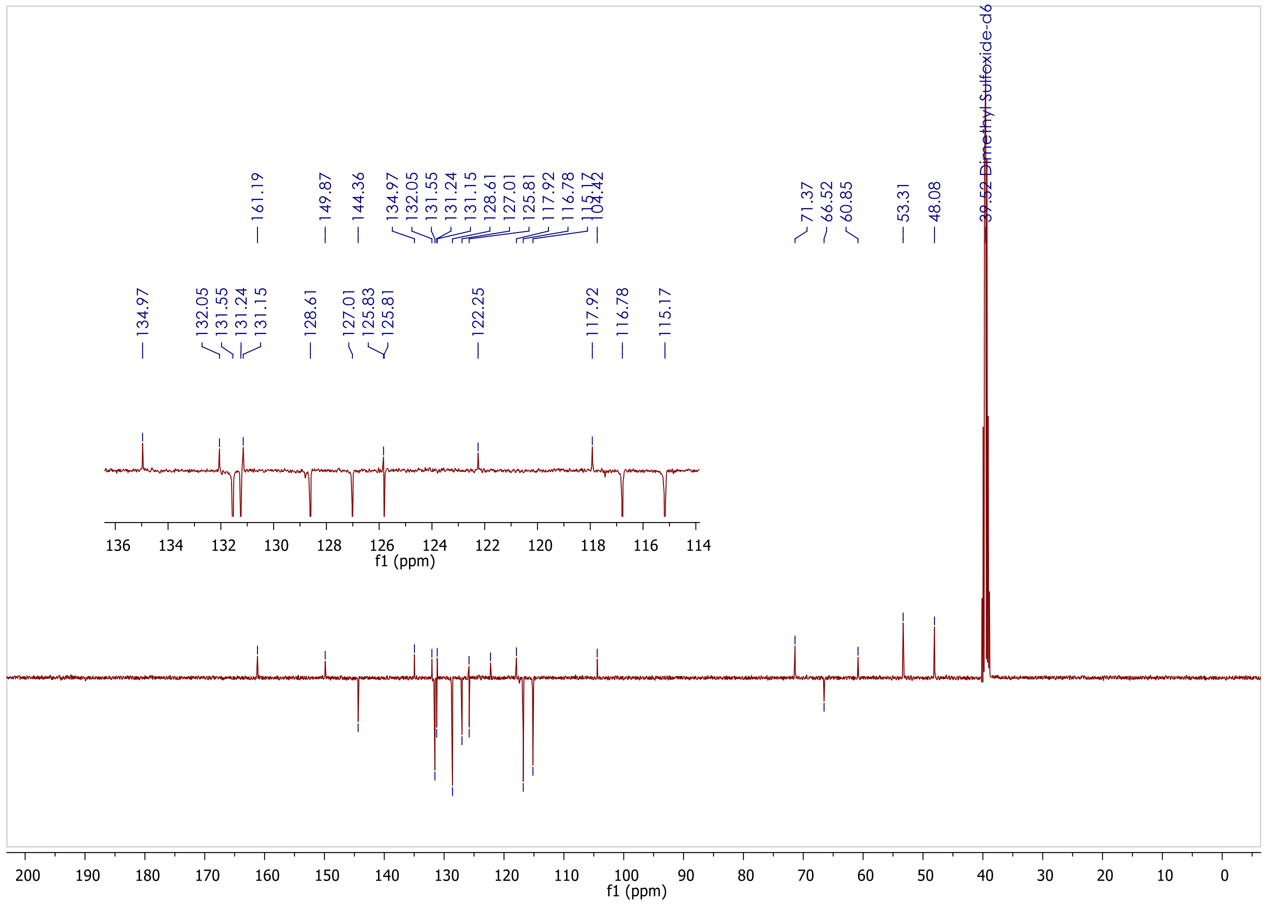

(*Z*)-3-(4-(3-(4-(4-bromophenyl)piperazin-1-yl)-2-hydroxypropoxy)phenyl)-2-(3,4-dichlorophenyl)acrylonitrile (**8r**)

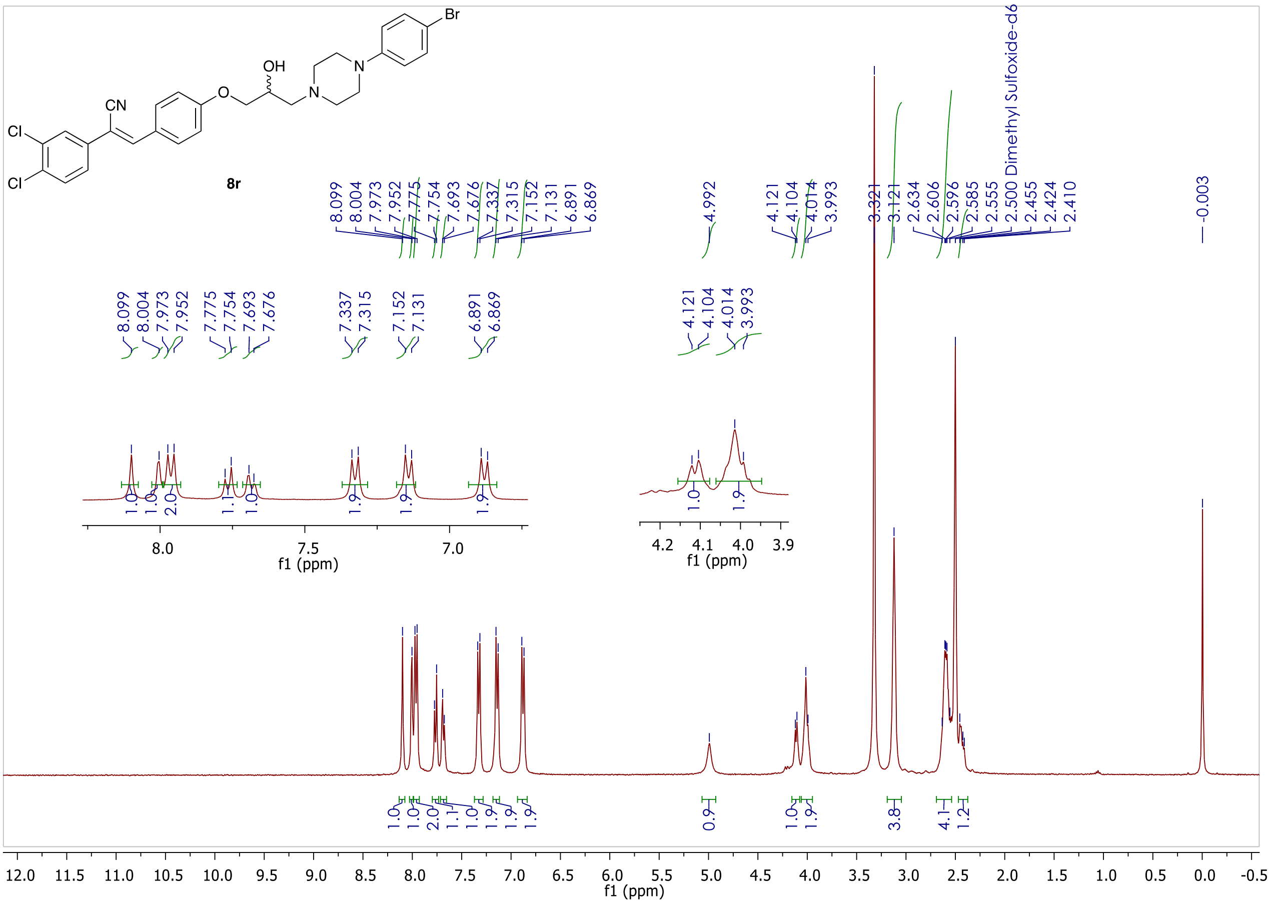

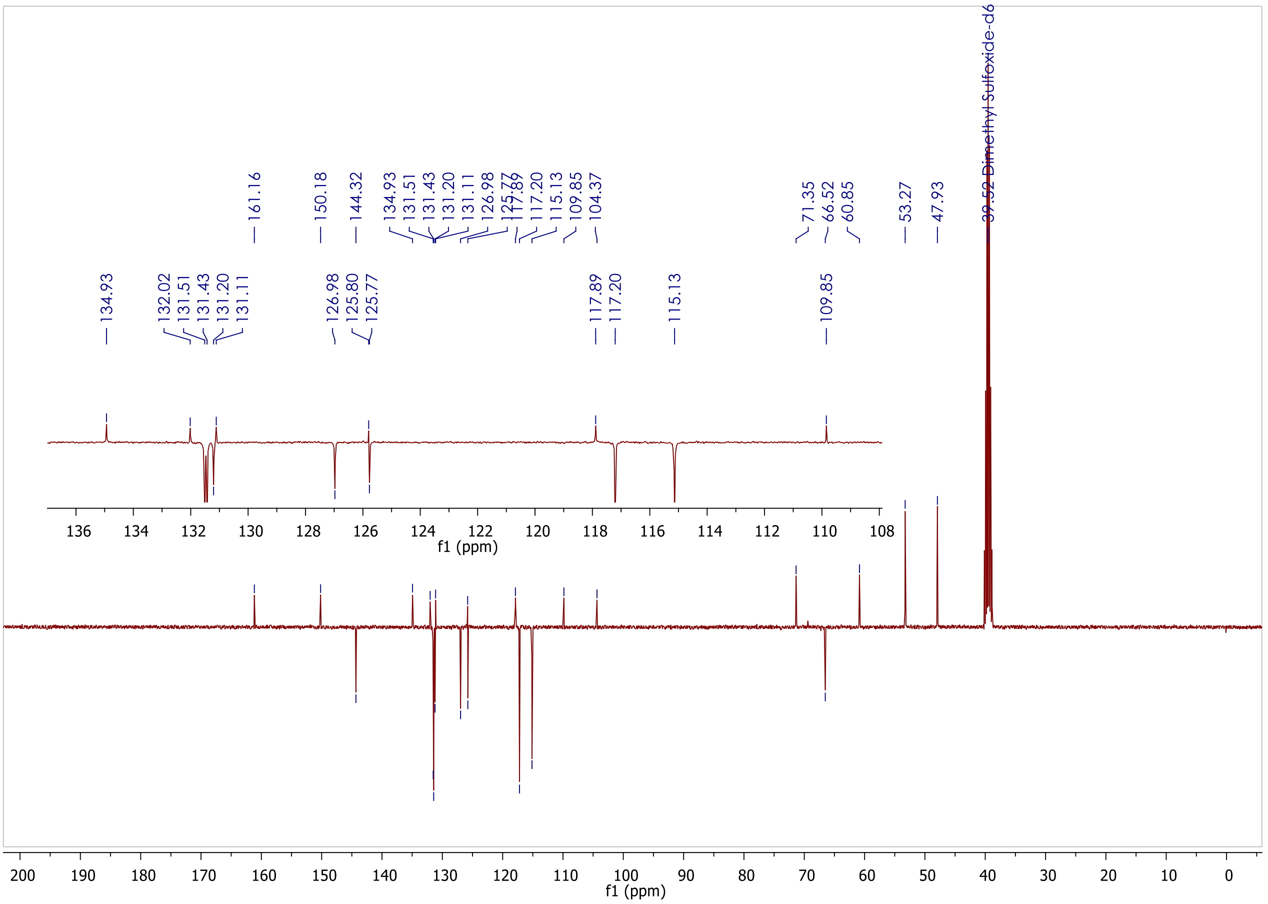

(*Z*)-2-(3,4-dichlorophenyl)-3-(4-(2-hydroxy-3-(4-(4-hydroxyphenyl)piperazin-1-yl)propoxy)phenyl)acrylonitrile (**8s**)

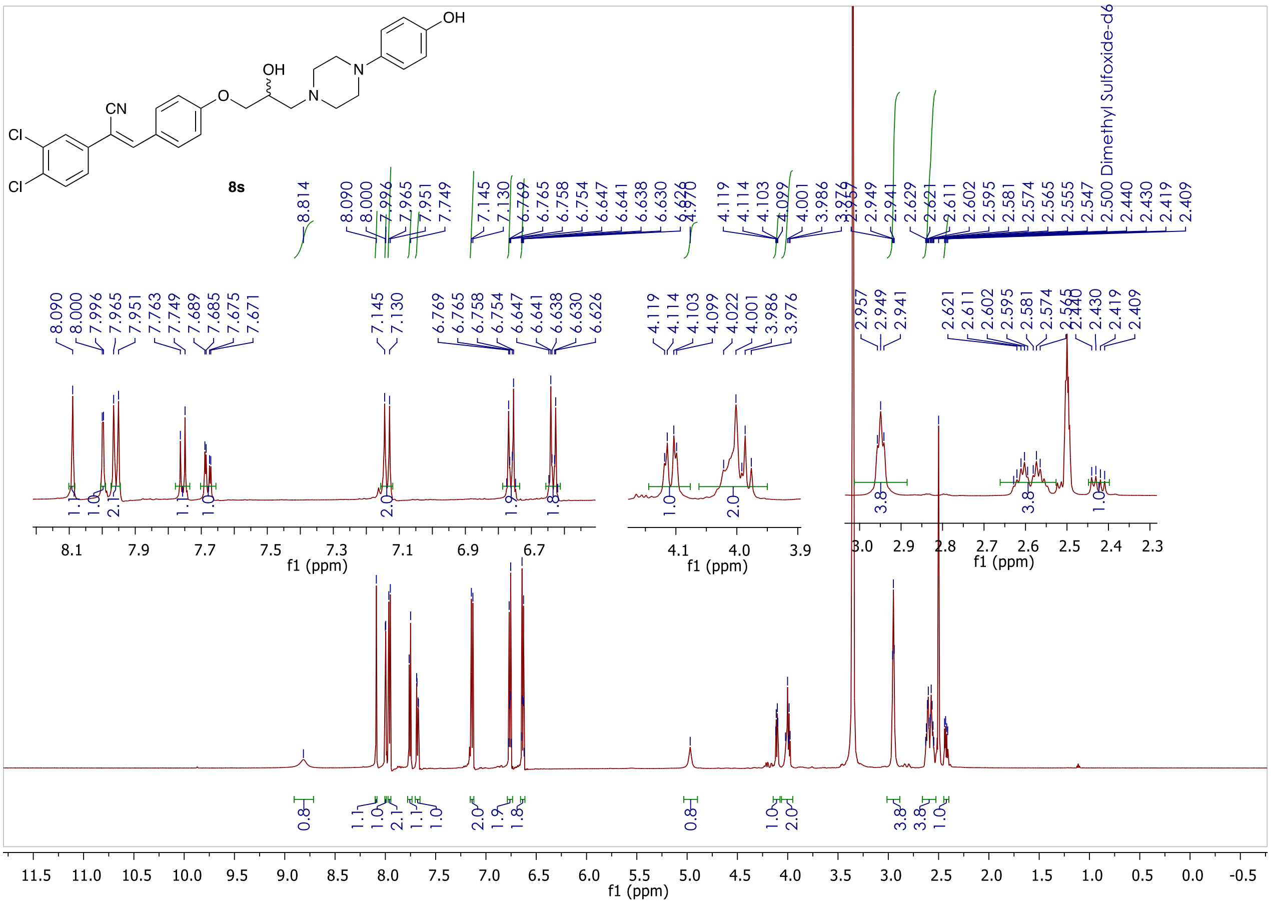

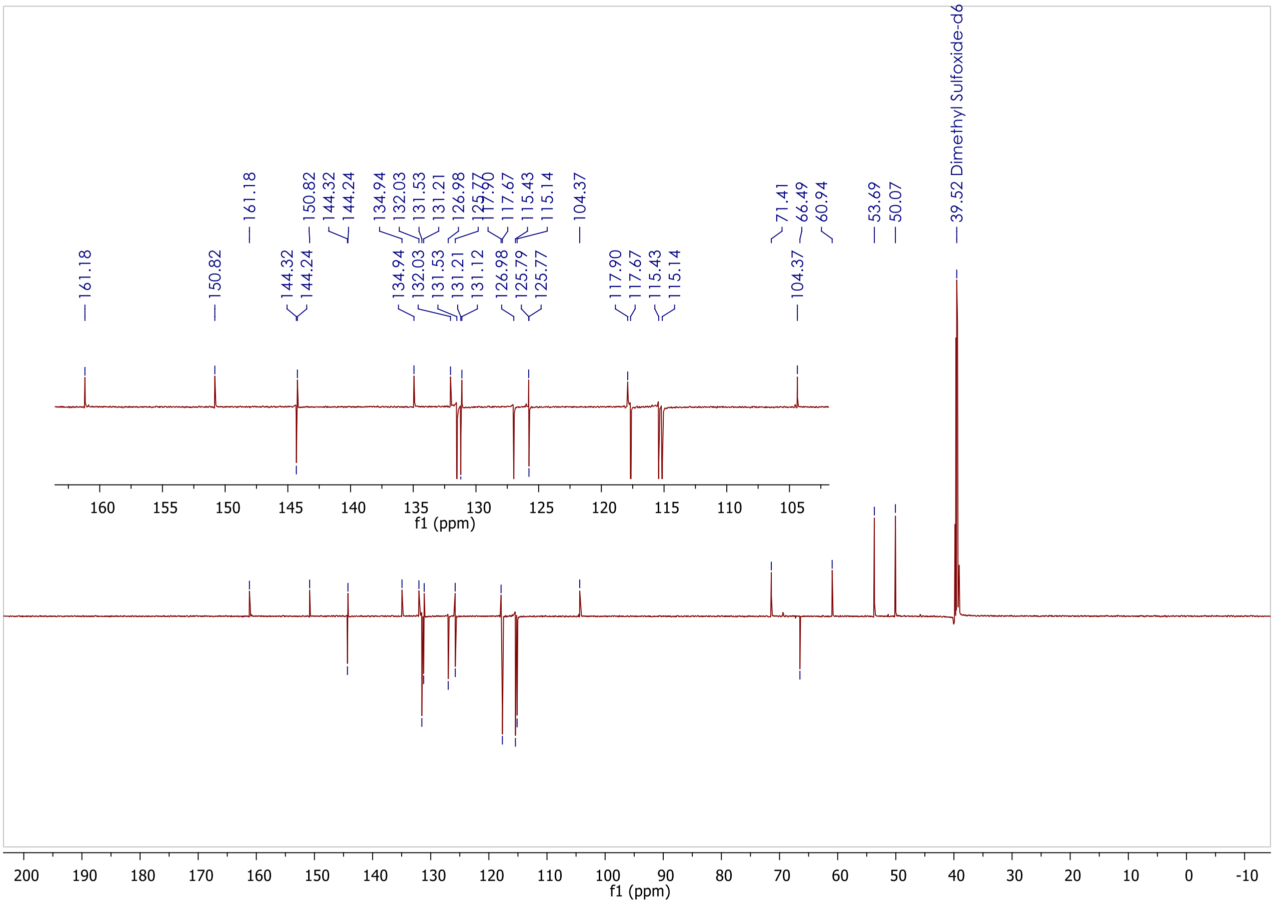

(*Z*)-2-(3,4-dichlorophenyl)-3-(4-(2-hydroxy-3-(4-(4-(trifluoromethyl)phenyl)piperazin-1-yl)propoxy)phenyl)acrylonitrile (**8t**)

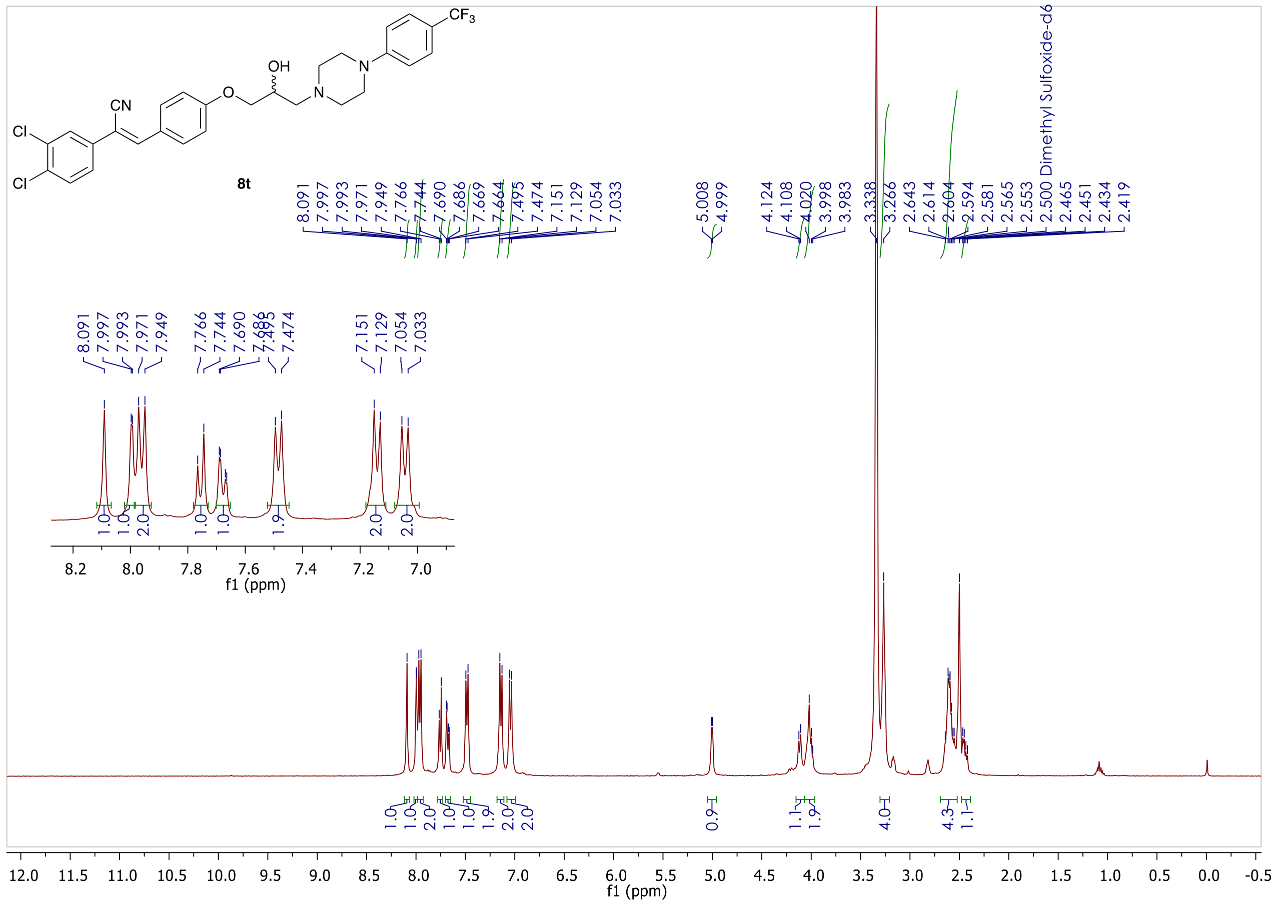

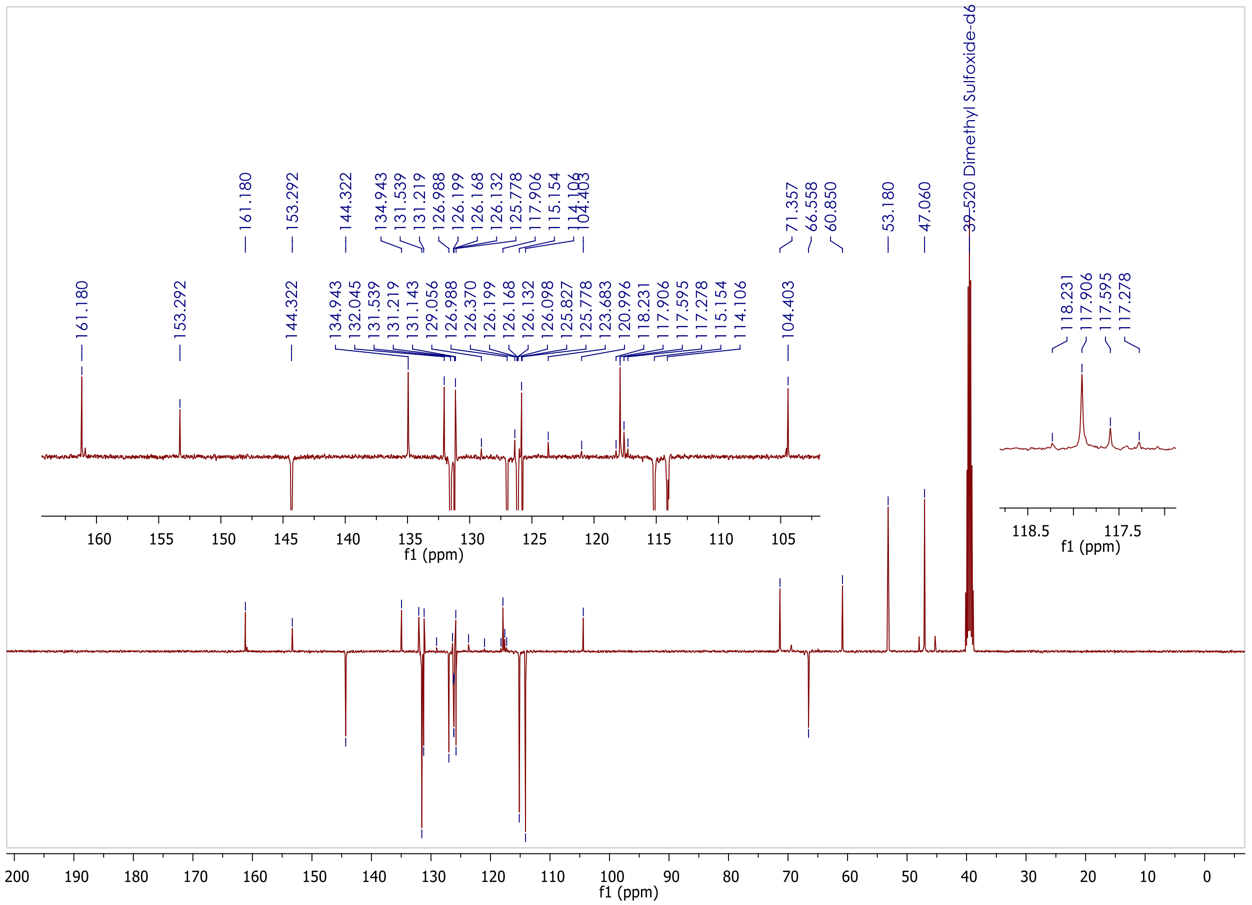

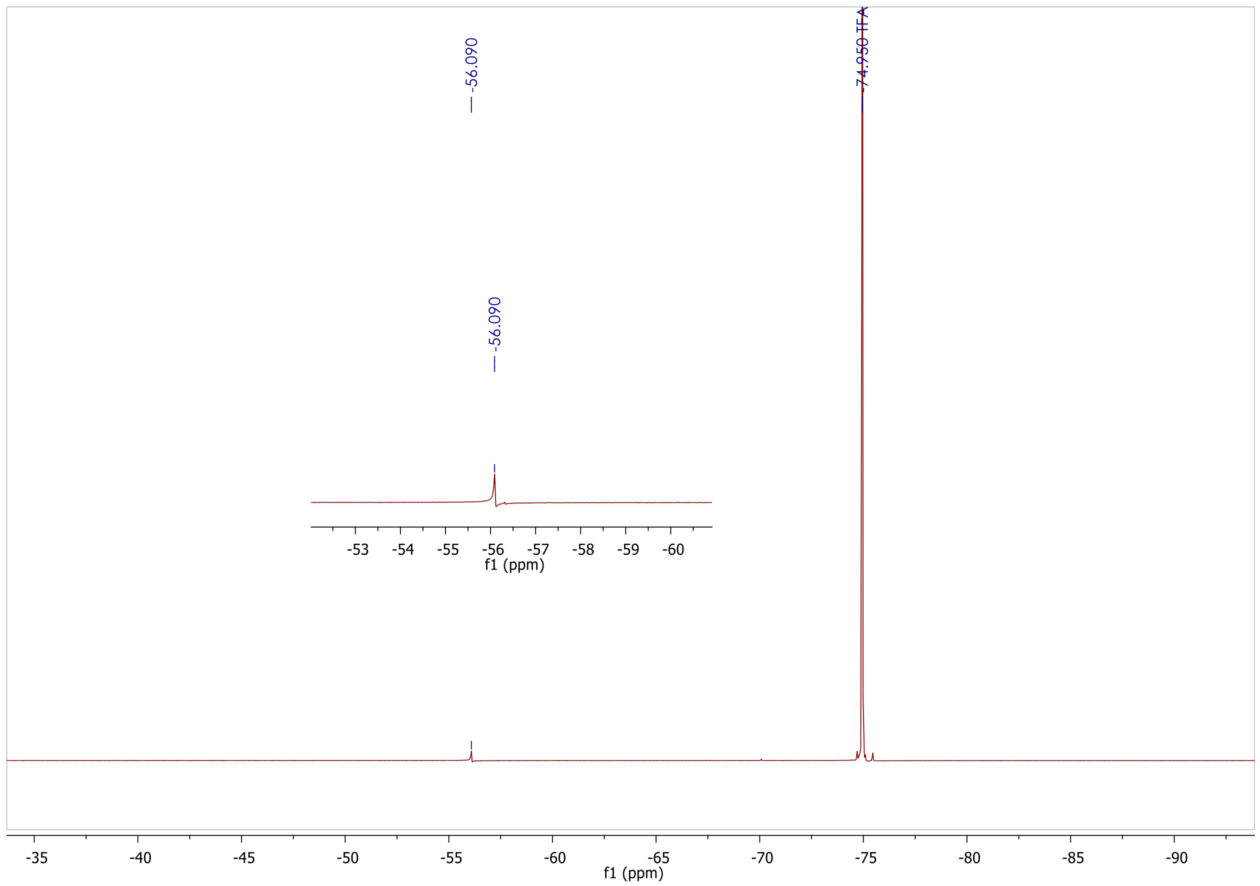
-56.090
-74.950 TFA
-56.090
-53
-54
-55
-56
-57
-58
-59
-60
f1 (ppm)
-35
-40
-45
-50
-55
-60
-65
-70
-75
-80
-85
-90
f1 (ppm)

(*Z*)-3-(4-(3-(4-(4-acetylphenyl)piperazin-1-yl)-2-hydroxypropoxy)phenyl)-2-(3,4-dichlorophenyl)acrylonitrile (**8u**)

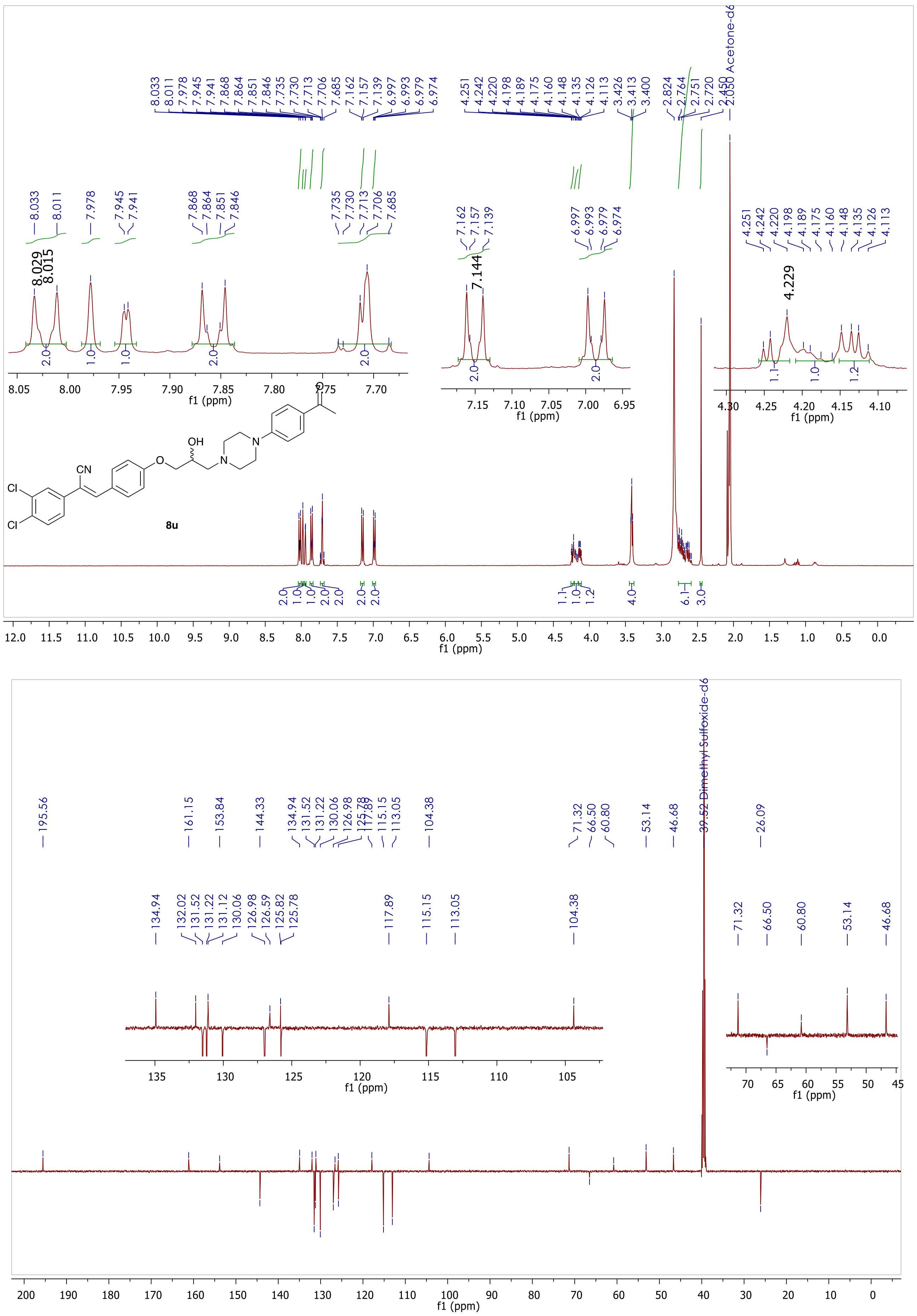

(*Z*)-2-(3,4-dichlorophenyl)-3-(4-(2-hydroxy-3-(4-(4-nitrophenyl)piperazin-1-yl)propoxy)phenyl)acrylonitrile (**8v**)

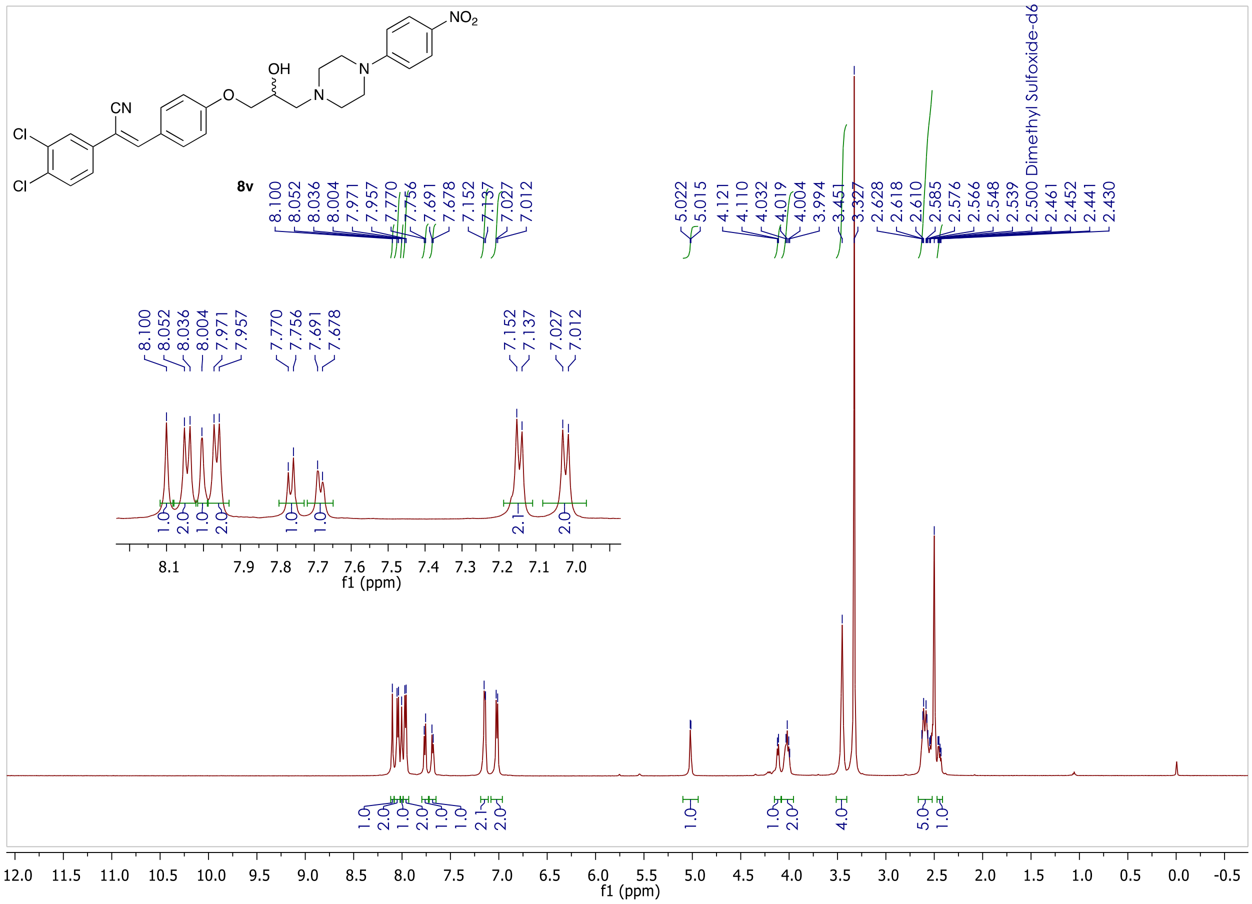

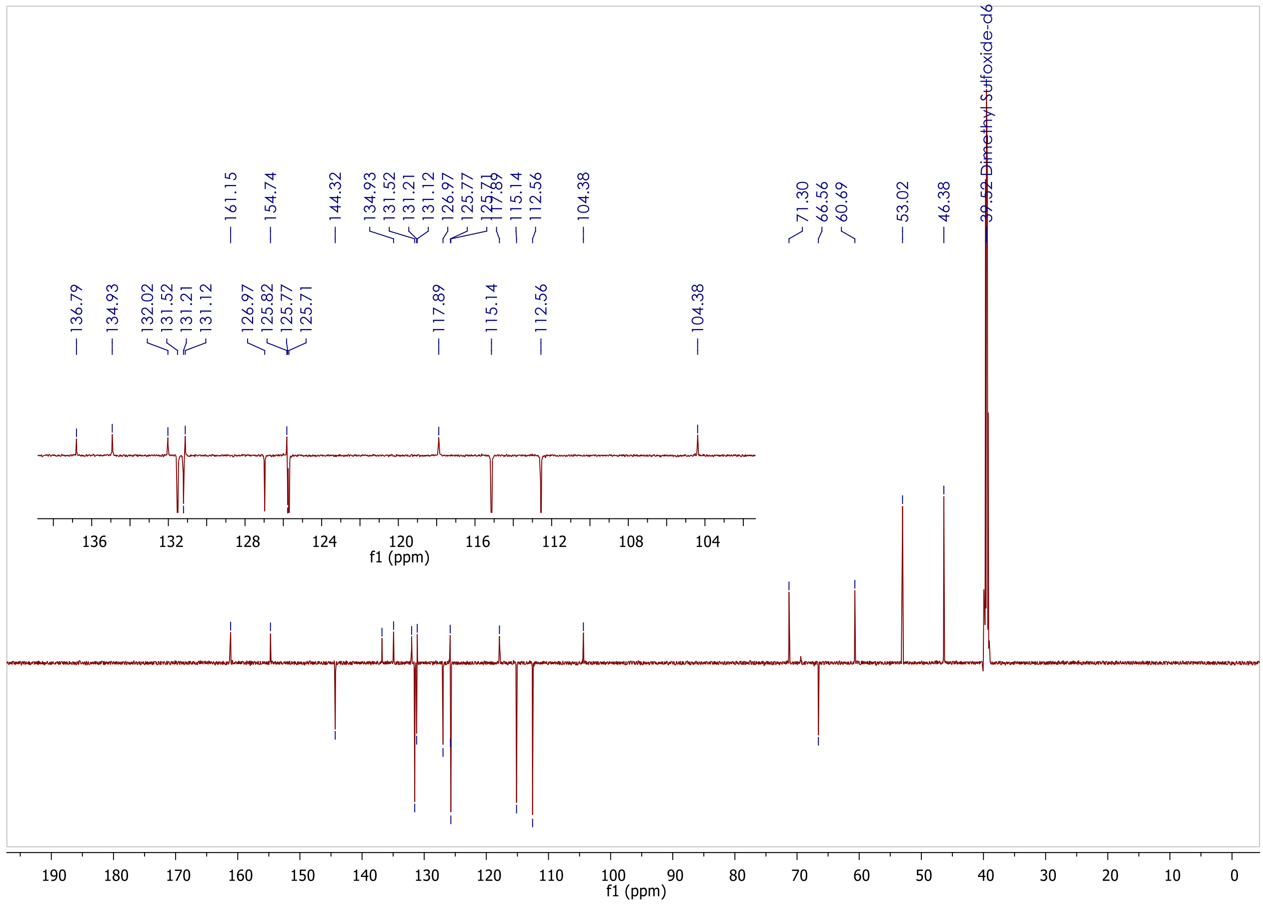

(*Z*)-2-(3,4-dichlorophenyl)-3-(4-(3-(4-(2-fluorophenyl)piperazin-1-yl)-2-hydroxypropoxy)phenyl)acrylonitrile (**8w**)

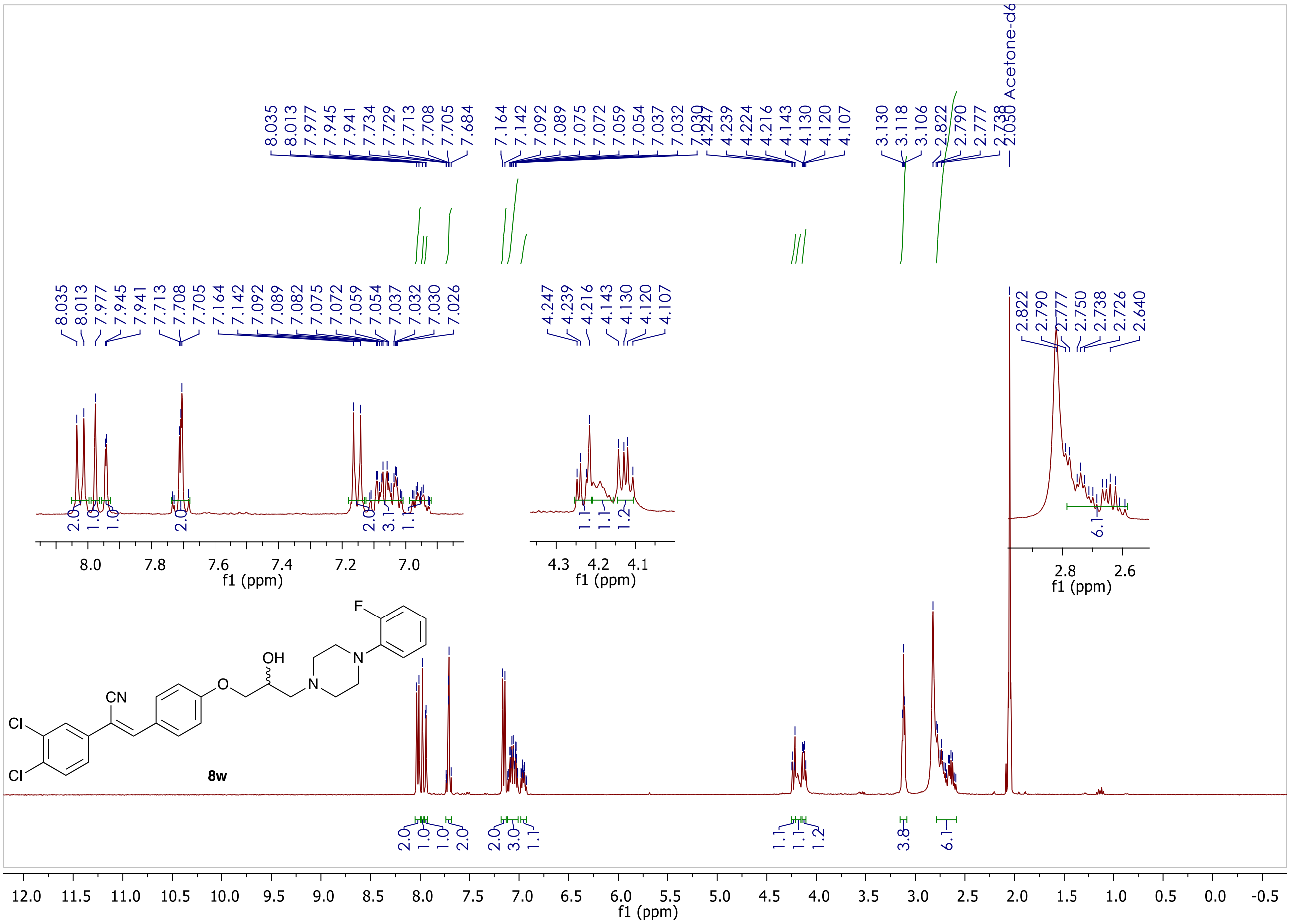

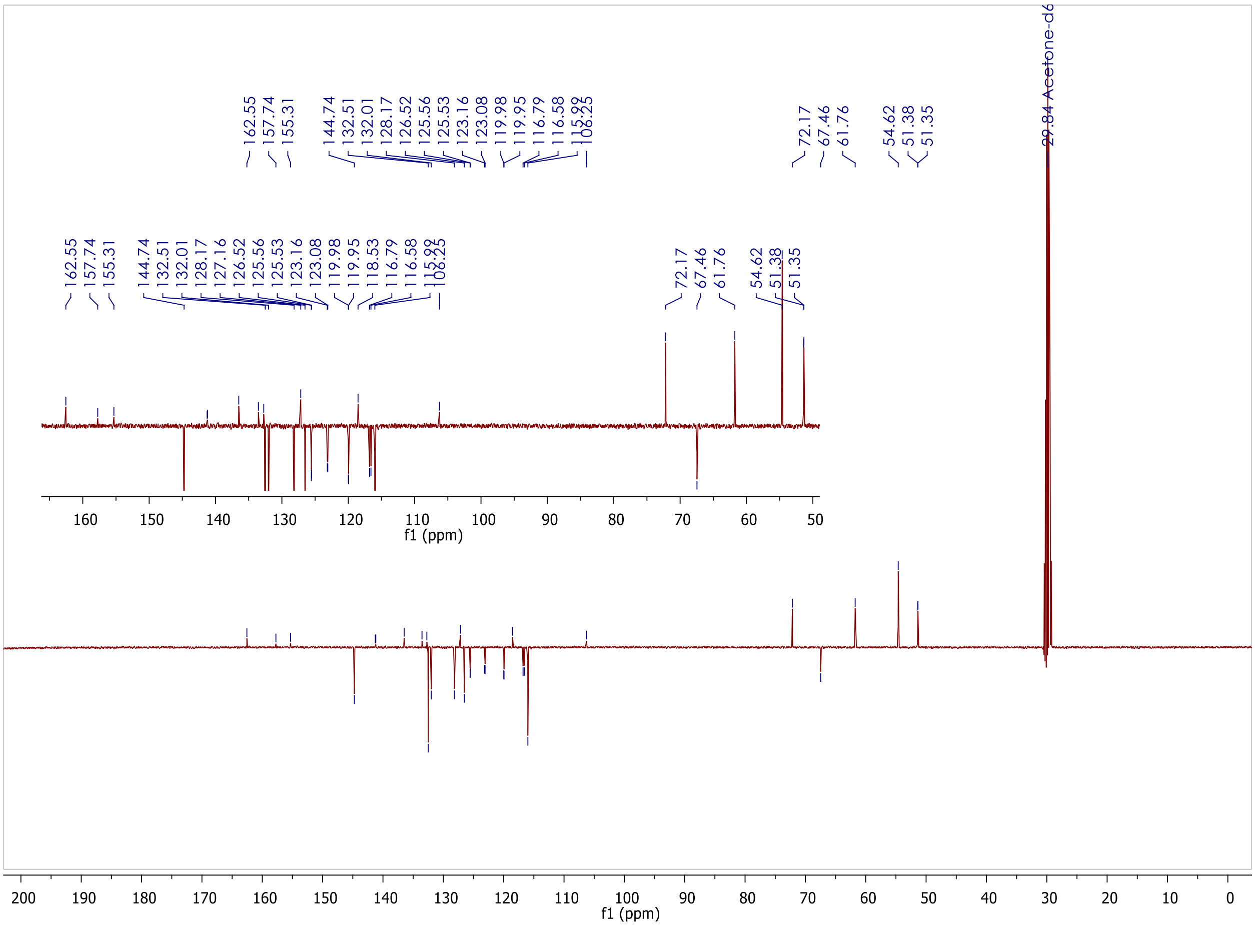

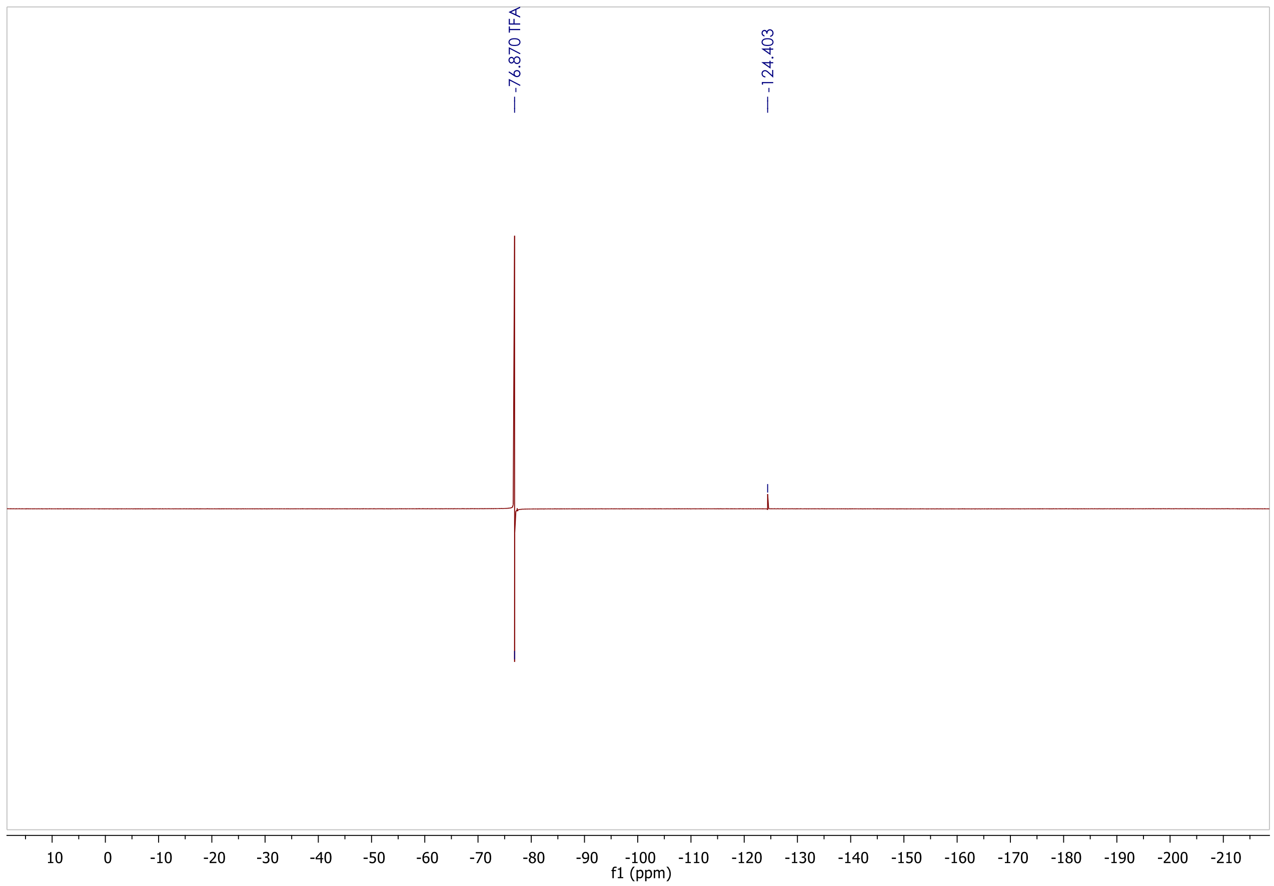
-76.870 TFA
-124.403
10
0
-10
-20
-30
-40
-50
-60
-70
-80
-90
-100
-110
-120
-130
-140
-150
-160
-170
-180
-190
-200
-210
f1 (ppm)

(*Z*)-2-(3,4-dichlorophenyl)-3-(4-(2-hydroxy-3-(4-phenylpiperidin-1-yl)propoxy)phenyl)acrylonitrile (**8x**)

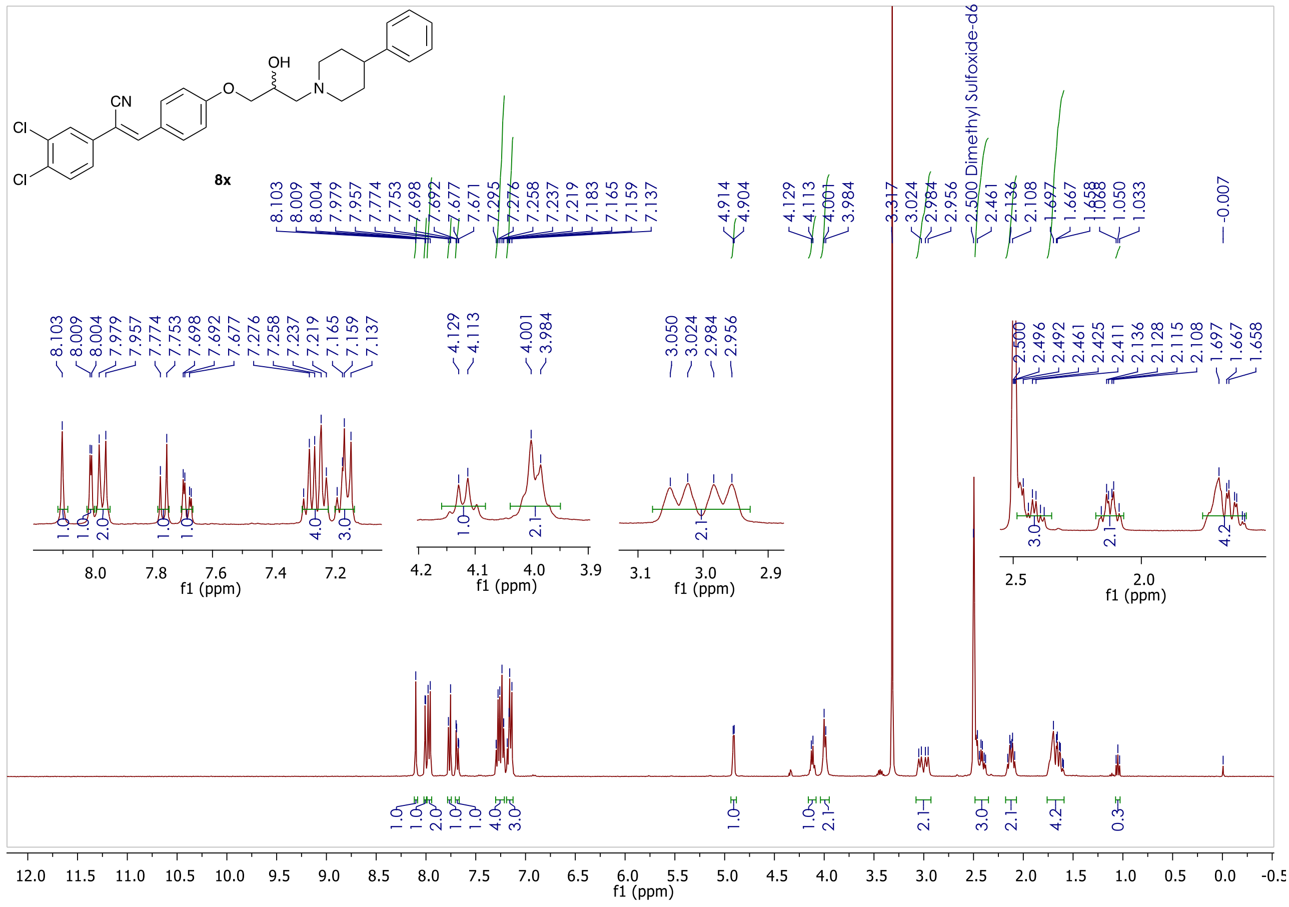

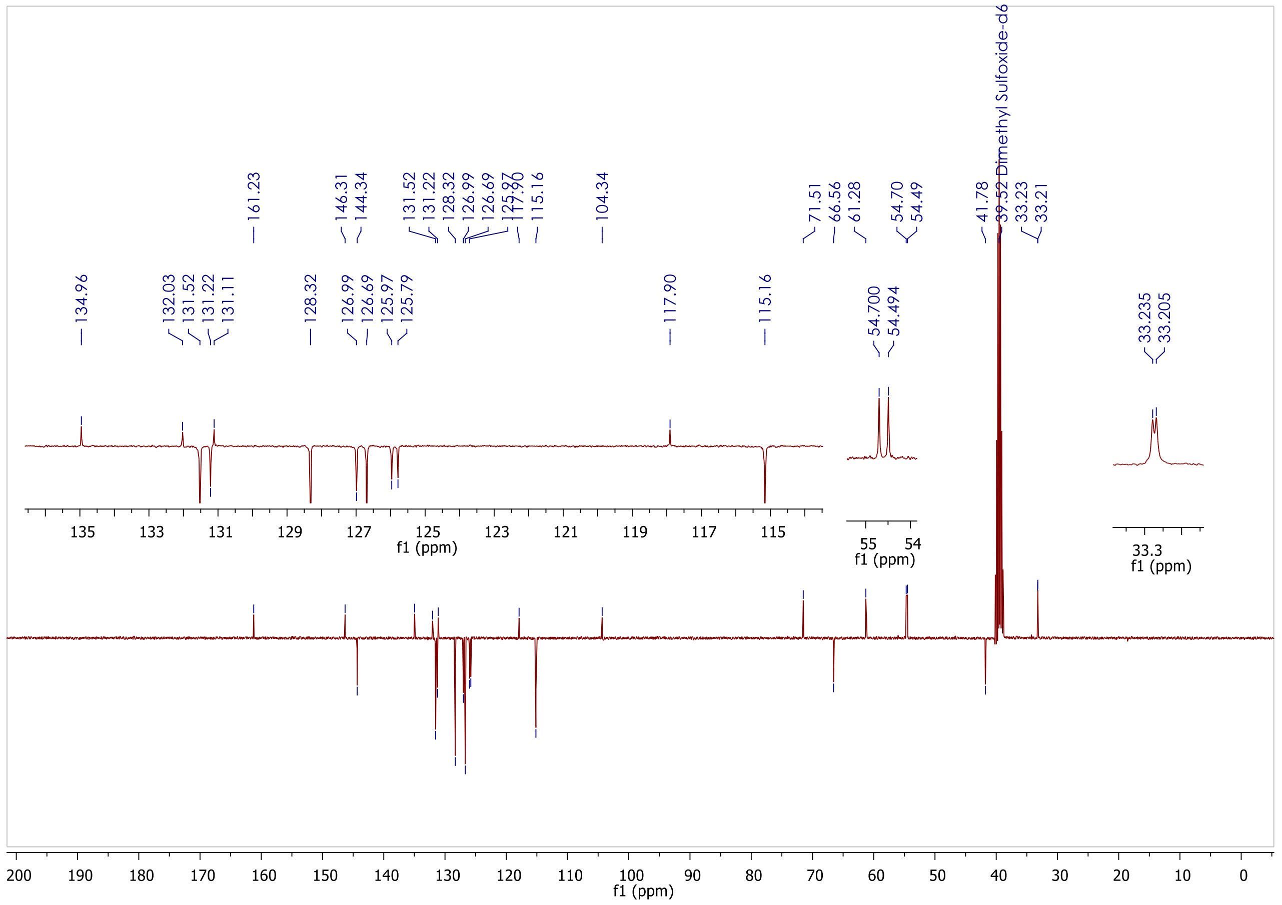

References

1. Cossar, P. J. Ma, C.; Gordon, C. P.; Ambrus, J. I.; Lewis, P. J.; McCluskey, A. (2017). Identification and validation of small molecule modulators of the NusB-NusE interaction. Bioorganic & Medicinal Chemistry Letters, 27(2), 162-167.
2. McCluskey, A.; Daniel, J.A.; Hadzic, G.; Chau, N.; Clayton, E.L.; Mariana, A.; Whiting, A.; Gorgani, N.N.; Lloyd, J.R.; Quan, A.; et al. Building a Better Dynasore: The Dyngo Compounds Potently Inhibit Dynamin and Endocytosis. *Traffic* **2013**, *14*, 1272–1289, https://doi.org/10.1111/tra.12119.
3. BakerJ.R.; GilbertJ.; PaulaS.; ZhuX.; SakoffJ.A.; McCluskeyA. Dichlorophenylacrylonitriles as AhR Ligands That Display Selective Breast Cancer Cytotoxicity in vitro. *ChemMedChem* **2018**, *13*, 1447–1458, doi:10.1002/cmdc.201800256.
4. TarletonM.; GilbertJ.; RobertsonM.J.; McCluskeyA.; SakoffJ.A. Library synthesis and cytotoxicity of a family of 2-phenylacrylonitriles and discovery of an estrogen dependent breast cancer lead compound. *MedChemComm* **2011**, *2*, 31–37, doi:10.1039/c0md00147c.
5. BakerJ.R.; RussellC.C.; GilbertJ.; SakoffJ.A.; McCluskeyA. Amino Alcohol Acrylonitriles as Activators of the Aryl Hydrocarbon Receptor Pathway: An Unexpected MTT Phenotypic Screening Outcome. *ChemMedChem* **2020**, *15*, 490–505, doi:10.1002/cmdc.201900643.
